# Meta-analysis of genome-wide association studies provides insights into genetic control of tomato flavor

**DOI:** 10.1038/s41467-019-09462-w

**Published:** 2019-04-04

**Authors:** Jiantao Zhao, Christopher Sauvage, Jinghua Zhao, Frédérique Bitton, Guillaume Bauchet, Dan Liu, Sanwen Huang, Denise M. Tieman, Harry J. Klee, Mathilde Causse

**Affiliations:** 1 INRA, UR1052, Génétique et Amélioration des Fruits et Légumes, Domaine Saint Maurice, 67 Allée des Chênes CS 60094, 84143 Montfavet Cedex, France; 2 MRC Epidemiology Unit & Institute of Metabolic Science, University of Cambridge, Addrenbrooke’s Hospital, Box 285, Hills Road, Cambridge, CB2 0QQ UK; 3 Genome Analysis Laboratory of the Ministry of Agriculture, Agricultural Genomics Institute at Shenzhen, Chinese Academy of Agricultural Sciences, 518124 Shenzhen, Guangdong China; 4 Key Laboratory of Biology and Genetic Improvement of Horticultural Crops of the Ministry of Agriculture, Sino-Dutch Joint Laboratory of Horticultural Genomics, Institute of Vegetables and Flowers, Chinese Academy of Agricultural Sciences, 100081 Beijing, China; 5 Horticultural Sciences, Plant Innovation Center, University of Florida, Post Office Box 110690, Gainesville, FL 32611 USA; 6 Present Address: Syngenta, 12 Chemin de l’Hobit, Saint Sauveur, 31790 France; 7 Present Address: Cardiovascular Epidemiology Unit, Department of Public Health and Primary Care, Strangeways Research Laboratory, University of Cambridge, Wort’s Causeway, Cambridge, CB1 8RN UK; 8 Present Address: Boyce Thompson Institute, Cornell University, 533 Tower Rd, Ithaca, NY 14853 USA

## Abstract

Tomato flavor has changed over the course of long-term domestication and intensive breeding. To understand the genetic control of flavor, we report the meta-analysis of genome-wide association studies (GWAS) using 775 tomato accessions and 2,316,117 SNPs from three GWAS panels. We discover 305 significant associations for the contents of sugars, acids, amino acids, and flavor-related volatiles. We demonstrate that fruit citrate and malate contents have been impacted by selection during domestication and improvement, while sugar content has undergone less stringent selection. We suggest that it may be possible to significantly increase volatiles that positively contribute to consumer preferences while reducing unpleasant volatiles, by selection of the relevant allele combinations. Our results provide genetic insights into the influence of human selection on tomato flavor and demonstrate the benefits obtained from meta-analysis.

## Abstract (web summary)

Flavor is one of the most important traits for improving tomato sensory quality and consumer acceptability. Here, the authors report meta-analysis of genome-wide association studies of flavor related traits and show genetic insights into the influence of human selection during domestication and improvement.

## Introduction

The deterioration of tomato flavor has been a source of complaint from consumers for decades^1^. During long-term domestication and breeding history, flavor has not been a priority, in contrast to yield, disease resistance, and postharvest shelf life^1,2^. However, flavor is one of the most important traits for improving tomato sensory quality and consumer acceptability^3^. Flavor is centrally influenced by sugars, acids, amino acids and a diverse set of volatiles^4–6^. Most of these compounds are quantitatively inherited as shown by many QTL studies but only a few QTLs have been positionally cloned^7^. Genome-wide association studies (GWAS) have detected many significant associated loci for tomato flavor related traits^6,8–12^ However, reducing a QTL to a causative gene is difficult and only a few candidate genes have been functionally validated^7^. The underlying genetic control of tomato flavor is still incomplete and remains an important breeding target.

Meta-analysis of genome-wide associations is powerful in dissecting complex human diseases^13,14^. A recent meta-analysis in cattle stature also demonstrated its power in non-human species^15^. However, to the best of our knowledge, no GWAS meta-analysis has been reported in major crops, despite the increasing number of GWAS studies in major crops, such as rice. To date, the genomes of over 500 tomato accessions have been fully sequenced^6,12,16–19^, making it possible to perform genotype imputation^20,21^ and subsequent meta-analysis of GWAS using summary data^14^ to decipher the polygenic architecture of agronomic traits. In this study, we perform a meta-GWAS on 775 tomato accessions and 2,316,117 SNPs and discover 305 significant associations for diverse flavor-related traits. Our results provide genetic insights into tomato flavor.

## Results

### Meta-analysis

**Fig. 1:**
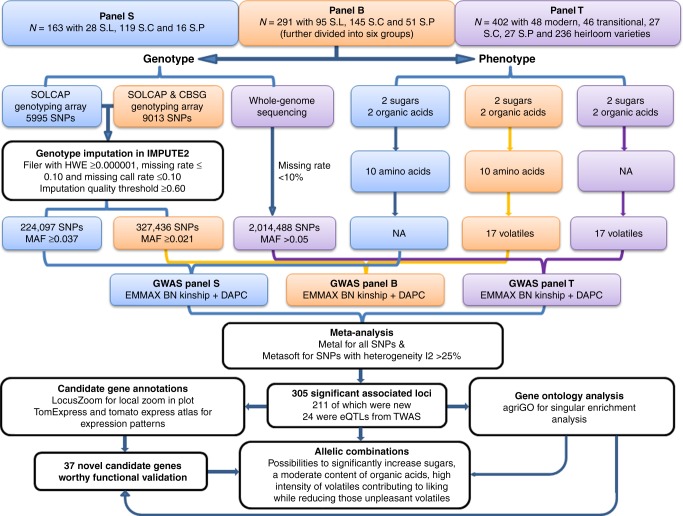
Overview of study design. N, the number of individuals; S.L, *S. lycopersicum*; S.C, *S. lycopersicum* var *cerasiforme*; S.P, *S. pimpinellifolium*; Genotyping arrays: SOLCAP, Solanaceae Coordinated Agricultural Project; CBSG, Centre of Biosystems Genomics consortium; HWE, Hardy–Weinberg equilibrium; MAF, minor allele frequency; GWAS, genome-wide association study; EMMAX, Efficient Mixed-Model Association eXpedited; DAPC, Discriminant Analysis of Principal Components; eQTL, expression quantitative trait locus; TWAS, transcriptome-wide association study

Here we report the first meta-analysis of GWAS in tomato using results of three publicly available GWAS panels: 163 tomato accessions from panel S^8^, 291 accessions from panel B^11^, and 402 accessions from panel T^6^ (Fig. 1). We analyzed a large set of tomato flavor-related quality chemicals, including sugars, organic acids, amino acids, and volatiles measured in each of these panels.

First, we used IMPUTE2 software^22^ to increase the genome-wide SNP densities of panel S^8^ and panel B^11^, which were genotyped using SNP arrays (Online methods). After quality control (Supplementary Figs. 1–3, Supplementary Tables 1 and 2, Supplementary Data 1–3), a total of 209,152 and 252,414 SNPs was retained for panel S and B, respectively. Imputation greatly increased the density of genomic coverage (Supplementary Fig. 4) and revealed a similar genetic population structure compared with genotyped SNPs for both panels (Supplementary Figs. 5–12 and Supplementary Data 4–5). We used the Efficient Mixed-Model Association eXpedited (EMMAX) software for association tests for panel S and B^23^, as reported for panel T^6^ (Online methods, Supplementary Fig. 13). After imputation, we observed a similar or slight statistical increase in terms of the significance and the number of associated loci compared with MLMM^24^ (Supplementary Figs. 14–44) and no genomic inflation (*λ* < 1) was detected for most (83.3%) of the traits (Supplementary Data 6). For panel T, which was characterized by 2,040,403 SNPs, the association tests had also been performed using EMMAX^6^.

By combining the three separate studies, a total of 775 unique tomato accessions were used for the final meta-analysis of 31 flavor-related traits (2 sugars, 2 organic acids, 10 amino acids, and 17 flavor-related volatiles). We performed the meta-analysis with two software: METAL^25^ using a fixed effect model and METASOFT^26^ for those SNPs where heterogeneity occurred (*I*^*2*^ > 25) using a random effect model. Manhattan plots and quantile–quantile (Q-Q) plots for all traits are shown in Supplementary Figs. 45–75. Meta-analysis identified a total of 305 significant loci (*P* < 4 × 10^−7^ for sugars, acids, and volatiles; *P* < 2.99 × 10^−6^ for amino acids), among which 211 were new (Supplementary Data 7). A total of 87 strong effect meta-QTLs were identified with high probability (*P* < 10^−9^). Most of these loci passed the suggestive thresholds in at least one panel (Supplementary Figs. 14–75). Among the identified loci, 35 had a moderate to strong heterogeneity (*I*^*2*^ > 25). We generated a local SQLite dataset for tomato (Online methods) and provided the LocusZoom plots for all the genome-wide significant associated loci (Supplementary Figs. 76–123). Among the 305 loci, 24 loci exhibited cis-eQTLs in a previous transcriptome-wide association study^12^ in fruit tissue (Supplementary Data 7). Among the 211 associated loci, we identified 37 promising candidate genes (7 with significant cis-eQTLs^12^) with functional annotations related to the pathways of flavor chemicals (Table 1).

**Table 1:** Summary of 37 candidate genes associated with main flavor-related traits in tomato fruit^a^

Trait	Chr	BP	Ref	Alt	*P*	*I* ^*2*^	Locus name	Candidate gene
Citrate	1	1749084	c	g	3.62 × 10^−13^	0	Solyc01g007090	Aluminum-activated malate transporter
Citrate	2	47904426	a	g	4.30 × 10^−13^	97.9	Solyc02g084820	Glycosyl transferase group 1
Citrate	3	52998165	a	c	1.84 × 10^−15^	0	Solyc03g083090	Glycogen synthase
Citrate	6	44955568	a	c	7.46 × 10^−27^	98.4	Solyc06g072920	Aluminum-activated malate transporter
Citrate	7	63601724	t	g	4.70 × 10^−12^	0	Solyc07g055840	Citrate synthase
Fructose	1	3327330	a	g	6.37 × 10^−11^	0	Solyc01g009150	Glycosyl hydrolase
Fructose	5	63485334	c	g	4.68 × 10^−10^	0	Solyc05g053400^a^	Glucosyltransferase
Fructose	7	63757414	a	c	4.28 × 10^−09^	0	Solyc07g055840	Citrate synthase
Fructose	8	64470216	a	g	2.33 × 10^−10^	96.2	Solyc08g081420	Glycosyltransferase-like protein
Fructose	10	422707	a	t	6.27 × 10^−10^	0	Solyc10g005510^a^	Glyceraldehyde-3-phosphate dehydrogenase
Fructose	10	65465775	t	c	6.84 × 10^−09^	0	Solyc10g086720	Fructose-1 6-bisphosphatase class 1
Glucose	1	1998383	a	g	2.36 × 10^−10^	0	Solyc01g007910	Succinyl-CoA ligase
Glucose	2	43844073	t	c	2.87 × 10^−09^	96.7	Solyc02g079220	Solute carrier family facilitated glucose transporter member 8
Glucose	4	911809	a	g	6.62 × 10^−09^	0	Solyc04g007160	Alpha-glucosidase
Glucose	8	58158082	a	g	4.99 × 10^−08^	0	Solyc08g069060	Beta-1 3-galactosyltransferase 6
Glucose	10	332069	t	g	1.20 × 10^−09^	0	Solyc10g005510^a^	Glyceraldehyde-3-phosphate dehydrogenase
Malate	1	2650772	t	c	2.08 × 10^−15^	0	Solyc01g008550	Cinnamoyl CoA reductase-like protein
Malate	9	72364359	a	t	1.34 × 10^−15^	0	Solyc09g098590	Sucrose synthase
Malate	11	55879120	a	c	7.14 × 10^−16^	0	Solyc11g072700	Glycosyltransferase-like protein
Malate	12	1824226	t	g	1.75 × 10^−19^	0	Solyc12g008430	Malic enzyme
Asparagine	2	54365596	a	g	3.72 × 10^−10^	94	Solyc02g093550^a^	Methyltransferase type 11
Asparagine	5	62468569	a	g	8.92 × 10^−09^	0	Solyc05g052170	Acetyltransferase GNAT family protein
Asparagine	12	64463407	t	c	1.13 × 10^−09^	0	Solyc12g089350	GDSL esterase/lipase
Aspartate	8	60307917	t	c	6.35 × 10^−09^	0	Solyc08g076350	Abhydrolase domain-containing protein
Aspartate	11	4008385	t	g	7.24 × 10^−11^	0	Solyc11g010960	Alcohol dehydrogenase
Aspartate	12	37536492	a	t	9.16 × 10^−08^	0	Solyc12g044940^a^	Short-chain dehydrogenase/reductase
Phenylalanine	11	4002767	t	c	9.57 × 10^−09^	0	Solyc11g010960	Alcohol dehydrogenase
Proline	3	66798980	t	g	2.39 × 10^−09^	0	Solyc03g117770^a^	Serine incorporator 1
Serine	3	69913055	a	g	3.06 × 10^−14^	0	Solyc03g121910	Threonine synthase
Geranyl acetone	2	40883244	a	g	6.00 × 10^−15^	0	Solyc02g081330	Phytoene synthase 2
Hexenal	1	1083181	c	g	1.45 × 10^−10^	0	Solyc01g006540	Lipoxygenase
Methyl salicylate	9	69293875	a	g	2.34 × 10^−19^	0	Solyc09g089580	1-aminocyclopropane-1-carboxylate oxidase-like protein
1-penten-3-one	5	3036212	a	g	7.07 × 10^−09^	0	Solyc05g008800^b^	Lipid phosphate phosphatase 3
2-methyl-1-butanol	6	37782796	a	g	5.50 × 10^−09^	0	Solyc06g059850	3-methyl-2-oxobutanoate dehydrogenase
6-methyl-5-hepten-2-one	3	3212583	t	c	6.76 × 10^−26^	0	Solyc03g025720	Long-chain-fatty-acid--CoA ligase
6-methyl-5-hepten-2-one	4	60345897	a	t	3.00 × 10^−11^	0	Solyc04g074360	UDP-glucuronosyltransferase
6-methyl-5-hepten-2-one	10	61007386	a	g	9.28 × 10^−09^	0	Solyc10g079470	L-galactono--lactone dehydrogenase

^a^A total of 305 loci for main tomato flavor-related quality traits were identified by meta-analysis of 775 tomato accessions and 2,316,117 SNPs. For each association, associated traits, chromosome (Chr), reference allele (Ref), alternative allele (Alt), the marker-trait association *P* value (*P*), heterogeneity I square (*I*^*2*^), locus name (International Tomato Annotation Group 2.4) and candidate genes are shown. All SNP positions were aligned on the tomato reference genome version 2.50. The *P-value* is reported from the random-effect model performed using the inverse variance-weighted fixed-effect model in METAL^25^. For those SNPs where heterogeneity occurs (*I*^*2*^ > 25, indicating moderate heterogeneity), we used the Han and Eskin random-effects model (RE2) implemented in METASOFT^26^. We also treated those candidate genes as new if previous GWAS did not report them though the association might be significant

^b^Significant cis expression quantitative trait loci (cis-eQTLs) from a previous transcriptome-wide association study (TWAS)^12^ mainly based on panel T

We performed a singular enrichment analysis for all associations using agriGO^27^ (http://bioinfo.cau.edu.cn/agriGO/index.php). Up to 10 biological processes were significantly enriched (*P* < 0.005) (Supplementary Data 8). All these enriched processes or groups were closely involved in flavor-related metabolites (in terms of sugars, organic acids, amino acids, and volatiles), such as UDP-glycosyltransferase activity, transferase activity, oxidoreductase activity, and carbohydrate metabolic processes.

Previously reported flavor-related loci in the three panels were all strongly associated in the meta-analysis at a higher significance level, such as *Lin5* (Solyc09g010080, fructose, *P* = 6.16 × 10^−10^; glucose, *P* = 4.30 × 10^−10^), *TFM6* (Solyc06g072920, malate, *P* = 2.26 × 10^−37^), and *Phytoene synthase 1* (Solyc03g031860, geranyl acetone, *P* = 6.73 × 10^−26^)^6,28^. In meta-analysis of GWAS, heterogeneity represents the genetic variations observed across combined studies^13^. In this study, strong heterogeneity occurred even for those loci with major effects, such as *Lin5* (fructose, *I*^*2*^ = 95.6, *P* = 1.05 × 10^−10^; glucose, *I*^*2*^ = 95.3, *P* = 5.85 × 10^−10^). This could be due to population structure, linkage disequilibrium, phenotyping platforms, G × E interactions, etc.^13^. We then focused on loci in regions showing low LD, where one or a few candidate genes could be identified and regions with medium LD but with candidate genes near the peak SNPs.

### Meta-analysis for sugar content

**Fig. 2:**
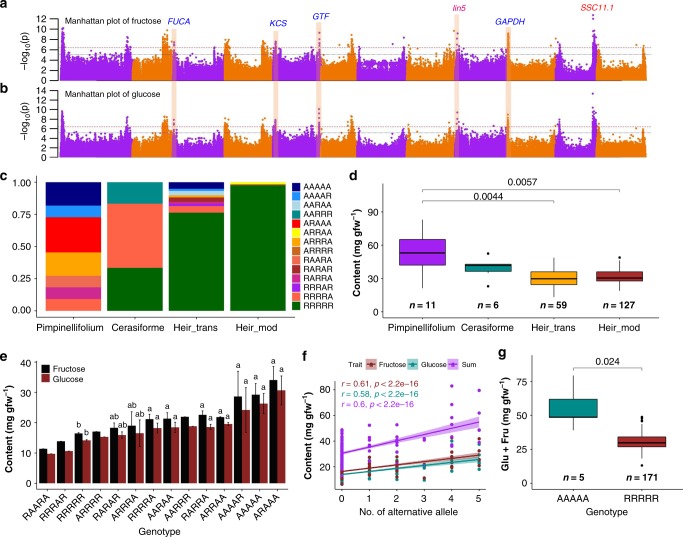
Combinations of fructose and glucose alleles for the improvement of tomato sugar content. Only alleles that were significantly associated both with fructose and glucose were analyzed. **a**, **b** Manhattan plot for meta-analysis of genome-wide association analysis of fructose (**a**) and glucose (**b**) content. Candidates and previously identified genes were labeled in blue and red, respectively. FUCA, *alpha-L-fucosidase 1*; KCS, *fatty acid elongase 3-ketoacyl-CoA synthase*; GTF, *glucosyltransferase*; GADPH, *glyceraldehyde-3-phosphate dehydrogenase*. **c** Allele distribution of fructose/glucose content at positions: chr3:1,506,106, chr5:3,403,706, chr5:63,485,334, chr9:3,477,979, and chr10:422,707 that were both significantly associated with fructose and glucose in *S. lycopersicum* var *cerasiforme* (cerasiforme), heirloom + transitional (heir_trans), heir + modern (heir_mod), and the closest wild species *S. pimpinellifolium* (pimpinellifolium) tomato accessions (see detailed information about groups in online methods). **d** Comparison of sugar content (fructose + glucose) between different tomato types in cerasiforme, heir_trans, heir_mod, and pimpinellifolium tomato accessions. **e** Mean (±SE) content of fructose (black) and glucose (brown) at different allele combinations in cerasiforme, heir_trans, heir_mod, and pimpinellifolium tomato accessions. Significant *t*-test *P* values are also provided. **f** Correlation between the number of alternative alleles and sugar content. Fructose, glucose, and the sum of fructose + glucose were colored in brown4, cyan4, and purple. **g** Comparison of sugar content (fructose + glucose) between all alternative and reference allele combinations at position chr3: 1,506,106, chr5: 3,403,706, chr5: 63,485,334, chr9: 3,477,979, and chr10: 422,707. Center line and limits of box were the mean and interquartile ranges. Error bars represent the maximum and minimum values. Whiskers indicate variability outside the upper and lower quartiles. Significant *t*-test *P* values are also provided. Source data of Fig. 2c–g are provided in a Source Data file

We looked into six candidate genes that were significantly associated both with fructose and glucose. In addition to *Lin5* and *SSC11.1*, we found four loci from the meta-analysis that were significantly associated both with fructose (Fig. 2a) and glucose content (Fig. 2b). These associations are in strong linkage disequilibrium with four candidate genes: *alpha-L-fucosidase 1* (*FUCA*; chr3: 1,506,106; fructose, *P* = 3.39 × 10^−8^; glucose, *P* = 1.46 × 10^−8^), *fatty acid elongase 3-ketoacyl-CoA synthase* (*KCS*; chr5: 3,403,706, fructose, *P* = 2.57 × 10^−8^; chr5: 3,406,424, glucose, *P* = 1.49 × 10^−8^), *glucosyltransferase* (*GTF*; chr5: 63,485,334; fructose, *P* = 4.68 × 10^−10^; glucose, *P* = 8.36 × 10^−10^), and *glyceraldehyde-3-phosphate dehydrogenase* (*GAPDH*; chr10:422,707, fructose, *P* = 6.27 × 10^−10^; chr10:332,069, glucose, *P* = 1.20 × 10^−9^). Notably, near the region of *FUCA* (up to ten genes), there are two candidate genes (Solyc03g006870, *phosphoglucomutase* and Solyc03g006860, *fructokinase*), which are also promising candidate genes for association with fructose and glucose content. Notably, *GTF* (*P* = 7.55 × 10^−34^) and GAPDH (*P* = 7.84 × 10^−17^) also showed significant cis-eQTL in a related transcriptome-wide association study^12^.

Interestingly, all these loci, except *Lin5* (which falls in the domestication sweep DW149^19^), were not associated with any domestication or improvement sweep^19^. We compared the frequencies of different combinations of alleles of these candidate genes in relation to sugar content in wild, transitional, heirloom and modern accessions (more detailed explanations about group definition in Online Methods). All modern, heirloom, and transitional accessions lost most of the diversity of allele combinations that is present in the wild species group (Fig. 2c). The sugar content of heirloom + transitional (heir_trans) and heirloom + modern (heir_mod) groups were both significantly lower than that of the wild species (Fig. 2d). Fruit sugar content increased gradually as the number of alternative alleles increased (Fig. 2e). We observed significant positive correlations between the number of alternative alleles within allele combinations and sugar content (Fig. 2f). In addition, total sugar content (glucose + fructose) of all alternative allele combinations was significantly higher (*P* = 0.024) than that of all reference allele combinations (Fig. 2g). Together, these results provide insights into possibilities for tomato sugar improvement.

### Meta-analysis for organic acids

The meta-analysis also provided several candidate genes for tomato fruit acid content. A strong association (*P* = 2.26 × 10^−37^ was detected for malate at an aluminum-activated malate transporter-like gene on chromosome 6, which has been reported to have a major effect on malate content^6,8,11^, and was further validated as *Al-Activated Malate Transporter 9* (*Sl-ALMT9*)^28^. We found a strong significant association for citrate (chr6: 44,955,568, *P* = 7.46 × 10^−27^), which was 1.54 kb away from *Sl-ALTM9* (Supplementary Fig. 45 and Table 1). We also identified a significant association with another aluminum-activated malate transporter on chromosome 1 (chr1:1,749,084, *P* = 3.62 × 10^−13^; Supplementary Fig. 45 and Table 1). The strong linkage with both citrate and malate indicated that *Al-Activated Malate Transporter* also plays an important role in regulating citrate content in tomato fruit.

**Fig. 3:**
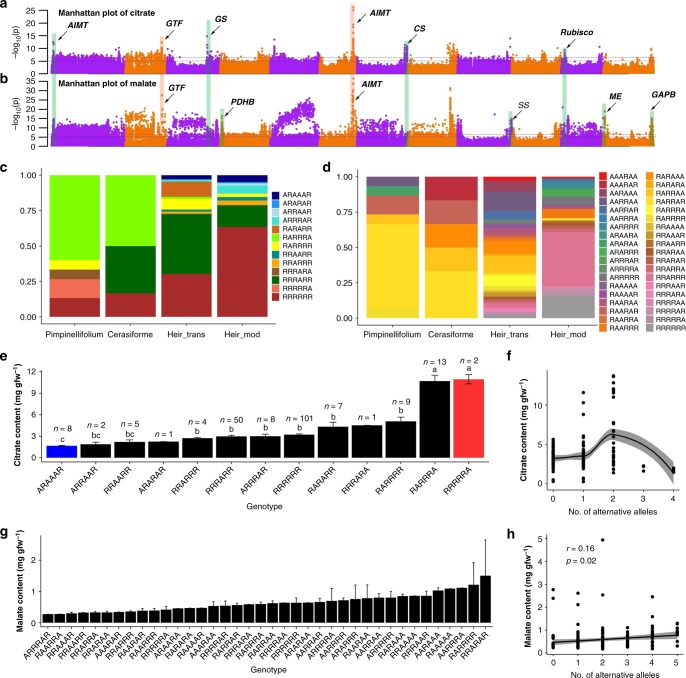
Combinations of citrate and malate alleles for the improvement of tomato organic acid content. **a**, **b** Manhattan plot for meta-analysis of genome-wide association analysis of citrate (**a**) and malate (**b**) content. AIMT, *Aluminum-activated malate transporter*; GTF, *Glycosyl transferase group 1*; GS, *Glycogen synthase*; AIMT, *Aluminum-activated malate transporter*; CS, *Citrate synthase*; Rubisco, *Ribulose-1 5-bisphosphate carboxylase/oxygenase activase 1*; PDHB, *Pyruvate dehydrogenase E1 component subunit beta*; SS, *Sucrose synthase*; ME, *Malic enzyme*; GAPB, *Glyceraldehyde-3-phosphate dehydrogenase B*. **c** Allele distribution of citrate content at positions: chr1:1749084, chr2: 47,904,426, chr3: 52,998,165, chr6: 44,955,568, chr7: 63,601,724, and chr10: 65,378,714 in cerasiforme, heir_trans, heir_mod, and pimpinellifolium tomato accessions. **d** Allele distribution of malate content at positions: chr2: 48,509,791, chr4: 2,156,747, chr6: 44,999,916, chr9: 72,364,359, chr12: 1,824,226, and chr12: 64,816,056 in cerasiforme, heir_trans, heir_mod, and pimpinellifolium tomato accessions. **e** Mean (±SE, standard error) content of citrate content at different allele combinations in cerasiforme, heir_trans, heir_mod, and pimpinellifolium tomato accessions. **f** Correlation between the number of alternative alleles and citrate content. **g** Mean (±SE) content of malate content at different allele combinations in cerasiforme, heir_trans, heir_mod, and pimpinellifolium tomato accessions. **h** Correlations between the number of alternative alleles and malate content. Source data of Fig. 3c–h are provided in a Source Data file

Candidate genes directly involved in the biosynthesis of citrate and malate were also identified. For example, we identified an association with citrate on chromosome 7, 150 kb away from a gene coding a citrate synthase (Solyc07g055840, *P* = 4.70 × 10^−12^). This candidate gene was also significantly associated with fructose (*P* = 4.28 × 10^−09^). For malate content, we found one association on chromosome 12 (chr12: 1,824,226, *P* = 1.75 × 10^−19^) close (36 kb) to a gene coding a malic enzyme (Solyc12g008430, four genes away from the peak SNP). We then took six candidate genes to analyze the relationships between different allele combinations and citrate and malate content, respectively (Fig. 3). The six candidate genes for citrate were *AIMT* (*Aluminum-activated malate transporter*, chr1: 1,749,084, *P* = 3.62 × 10^−13^), *GTF* (*Glycosyl transferase group 1*, chr2: 47,904,426, *P* = 4.30 × 10^−13^), *GS* (*Glycogen synthase*, chr3: 52,998,165, *P* = 1.84 × 10^−15^), *AIMT* (*Aluminum-activated malate transporter*, chr6: 44,955,568, *P* = 7.46 × 10^−27^), *CS* (*Citrate synthase*, chr7: 63,601,724, *P* = 4.70 × 10^−12^), and *Rubisco* (*Ribulose-1 5-bisphosphate carboxylase/oxygenase activase 1*, chr10: 65,378,714, *P* = 5.35 × 10^−09^). The six candidate genes for malate were *GTF* (*UDP-glucosyltransferase*, chr2: 48,509,791, *P* = 3.47 × 10^−28^), *PDHB* (*Pyruvate dehydrogenase E1 component subunit beta*, chr4: 2,156,747, *P* = 4.45 × 10^−17^), *AIMT* (*Aluminum-activated malate transporter*, chr6: 44,999,916, *P* = 2.26 × 10^−37^), *SS* (*Sucrose synthase*, chr9: 72,364,359, *P* = 1.34 × 10^−15^), *ME* (*Malic enzyme*, chr12: 1,824,226, *P* = 1.75 × 10^−19^), and *GAPB* (*Glyceraldehyde-3-phosphate dehydrogenase B*, chr12: 64,816,056, *P* = 5.99 × 10^−16^).

Among the selected candidates, *GTF* on chromosome 2 and *AIMT* on chromosome 6 were associated with both citrate and malate (Fig. 3a, b). Both *GTF* and *GS* are located within improvement sweeps (IS031 and IS044, respectively)^19^ and domestication sweeps (DS050 and DS175)^19^ were observed for malate on *PDHB* and *ME*. For citrate and malate, the modern tomato accessions presented very different allele combinations than those in wild species and cherry tomatoes (Fig. 3c, d). In comparison, the total number of allele combinations for malate was approximately three times that of citrate. The citrate content was significantly different between some allele combinations (Fig. 3e). With the increase in the total number of alternative alleles in different allele combinations, the citrate content first increased gradually, with a peak at *n* = 2, and then steadily decreased (Fig. 3f). The malate content also showed a wide range of variation among alleles (Fig. 3g and Supplementary Data 9). We observed a weak but significant (*P* = 0.02) positive linear correlation (*r* = 0.16) between the number of alternative alleles and malate content (Fig. 3h).

These results demonstrated that citrate content was more influenced by improvement sweeps while malate was more influenced by domestication sweeps in the long-term breeding history. In addition, citrate has much less allele diversity than malate and a distinct pattern of relationships between the number of alternative alleles and its content.

### Meta-analysis for amino acids and volatiles

Many candidate genes associated with amino acid and volatile contents were identified. For example, we found a significant association for serine on chromosome 3 (*P* = 3.06 × 10^−14^) (Supplementary Fig. 57 and Table 1), which was only significant in panel B (*P* = 2.13 × 10^−9^) (Supplementary Fig. 26). The candidate gene is annotated as a threonine synthase, an enzyme involved in the serine biosynthesis pathway. For proline, we found one associated locus (Solyc03g117770, *P* = 2.39 × 10^−9^), which was also reported as a significant eQTL (*P* = 1.04 × 10^−35^)^12^. This gene is a serine incorporator, and directly regulates serine content. One locus corresponding to GDSL esterase/lipase (Solyc12g089350) was also significantly associated with four amino acids (asparagine, GABA, glutamine and threonine). For hexanal, we found the strongest association corresponding to the lipoxygenase gene *LoxC* (Solyc01g006540, *P* = 1.45 × 10^−10^), which encodes an enzyme that is essential for synthesis of C6 and C5 fatty acid-derived volatiles^29,30^. This candidate gene was also significantly associated with (Z)-3-hexen-1-ol (*P* = 3.94 × 10^−07^). For 2-methyl-1-butanol, the strongest association corresponded to a 3-methyl-2-oxobutanoate dehydrogenase gene (Solyc06g059850, *P* = 5.50 × 10^−09^), an enzyme associated with branched chain amino acid metabolism.

**Fig. 4:**
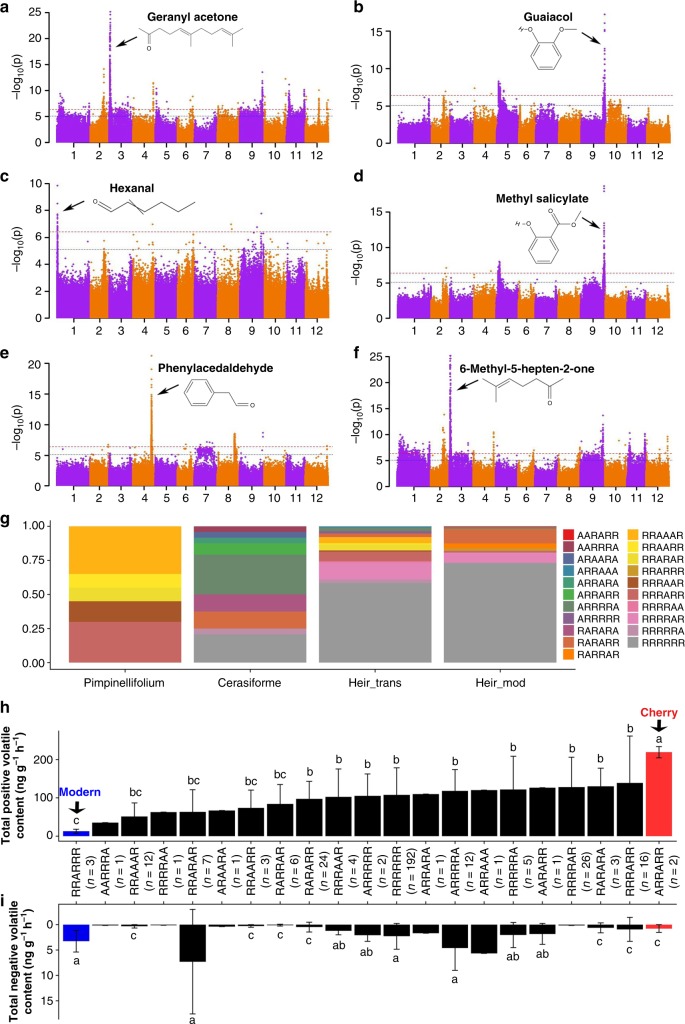
Combinations of six volatile alleles for the improvement of tomato volatile content. **a**–**f** Manhattan plot for meta-analysis of genome-wide association analysis of geranyl acetone (**a**), guaiacol (**b**), hexanal (**c**), methyl salicylate (**d**), phenylacetaldehyde (**e**), and 6-methyl-5-hepten-2-one (**f**) content. **g** Allele distribution of six volatiles content at positions: chr3: 4,328,514 (geranyl acetone), chr9: 69,299,940 (guaiacol), chr1: 1,083,181 (hexanal), chr9: 69,293,875 (methyl salicylate), chr4: 55,635,636 (phenylacetaldehyde), and chr3: 3,212,583 (6-methyl-5-hepten-2-one) in cerasiforme, heir_trans, heir_mod, and pimpinellifolium tomato accessions. **h**, **i** Mean (±SE, standard error) content of total content of the four positive volatiles (geranyl acetone, hexanal, phenylacetaldehyde and 6-methyl-5-hepten-2-one) (**h**) and two unpleasant volatiles (lower panel, guaiacol and methyl salicylate) (**i**) at different allele combinations in cerasiforme, heir_trans, heir_mod and pimpinellifolium tomato accessions. Source data of Fig. 4g–i are provided in a Source Data file

We then looked at the possibility that significantly increasing the overall intensity of volatiles contributed to consumer liking as well as significantly reducing the overall content of unpleasant volatiles by combining the strongest loci associated with the contents of six volatiles (Fig. 4). The four volatiles positively contributing to liking included geranyl acetone (chr3: 4,328,514, *P* = 6.73 × 10^−26^), hexanal (chr1: 1,083,181, *P* = 1.45 × 10^−10^), phenylacetaldehyde (chr4: 55,635,636, *P* = 5.59 × 10^−22^), and 6-methyl-5-hepten-2-one (chr3: 3,212,583, *P* = 6.76 × 10^−26^). The two unpleasant (or negative) volatiles were guaiacol (chr9: 69,299,940, *P* = 5.90 × 10^−18^) and methyl salicylate (chr9: 69,293,875, *P* = 2.34 × 10^−19^) (Fig. 4a–f). Modern and heirloom + transitional accessions had the lowest allele diversity, especially compared with *S. pimpinellifolium* and cherry tomato accessions (*S. l. cerasiforme*). Interestingly, we also found that cherry tomatoes had the greatest diversity of allele combinations and some of them only appeared in this group (Fig. 4g). The highest total content of the four positive volatiles was observed in allele combinations of cherry tomato accessions, which were significantly higher than the allele combinations of all modern tomato accessions (Fig. 4h). In contrast, modern accessions have, on average, a significantly higher content of unpleasant volatiles, compared with the cherry accessions (Fig. 4i). These results revealed the combinations of alleles that have the potential to significantly enhance the total contents of volatiles associated with consumer liking.

## Discussion

With the development of next-generation sequencing technology, GWAS has become a classical genetic approach to identify QTLs and causal genes in crops^31^. We herein demonstrate the potential of meta-analysis of GWAS following the detailed protocols first proposed in human genetics^32,33^, which can be easily applied in other crops. Meta-analysis of GWAS is used when pooling raw data of separate panels (mega-analysis) is not possible. It has been shown both theoretically and numerically that meta-analysis is statistically as efficient as mega-analysis^34,35^. Even when possible, it is thus not necessary to re-analyze the raw data to perform meta-analysis. Only summary data (beta, standard error and p-values of associations at each SNP) from each panel is needed and should be provided with each GWAS result. For mega-analysis, genotypes and phenotypes from all panels should be first combined and then analyzed, which requires proper management of phenotypic structure (data coming from different studies with different plant growth conditions, different harvesting and sampling procedures, different metabolic analysis protocols etc.) and genotypic structure (such as population structure and kinship). Compared to mega-analysis, meta-analysis can assess the heterogeneity (consistency) of studies, which can be caused by many factors, such as phenotypic structure, genetic structure, linkage disequilibrium, imputation accuracies or G × E interactions^13,34^.

Flavor remains a major breeding challenge in tomato^1,6^. Here, we used imputation-driven meta-analysis of genome-wide association studies to greatly increase the number of SNPs linked to chemicals associated with flavor. Among the 305 significantly associated loci, 41% of the SNPs had a low frequency (MAF < 0.1). Very low-frequency (0.01 < MAF < 0.05) SNPs were also detected (3 significant associated loci) (Supplementary Fig. 124). These results demonstrated that a sufficiently large sample size is needed to uncover these low-frequency and less common variants and to account for missing heritability^36–38^. Although hundreds of tomato genome sequences have been published^6,12,16–19^, a high sequence depth reference panel is needed, such as the 1000 Genomes Project^39^ in humans or the 1135 Arabidopsis genomes^40^ in Arabidopsis, to perform genotype imputation^20,21^, heritability estimation^36,41–43^ and meta-analysis^13,14^ with higher accuracy. Also, an imputation server could greatly enhance the integration of genetic resources^44^.

In this study, we identified 37 promising candidate genes with functional annotations consistent with their involvement in biosynthesis of flavor chemicals. With the advancement of genome editing technologies, their functional analysis could greatly promote our knowledge of the genetic architecture of tomato flavor, provide fully linked markers for breeding and ensure consumer satisfaction^45–48^. It is also possible now to introduce desirable traits into wild stress-tolerant tomato accessions by genome editing^49,50^. However, tomato flavor can only be significantly improved when multiple genes are modified.

Many consumers are more attracted by small and medium size tomatoes with superior taste^51^, as higher sugar content is usually associated with smaller fruit size^6^. In the meta-analysis, we found that modern cultivars have lost the majority of high-sugar alleles that were present in transitional, cherry tomato varieties and wild species. All these loci did not seem to have been influenced by any domestication or improvement sweeps, with the exception of *Lin5*, but some were loosely linked to fruit weight QTLs due to large LD in tomato. These results reflect the fact that sugar content has not been a breeding priority, in contrast to fruit size, yield, biotic, and abiotic resistances^1,6^. Strong positive correlations between the number of alternative alleles and sugar content provide clues on how to select higher sugar content tomato cultivars. However, sugar content can only be significantly improved when almost all the alternative alleles are selected, and will probably be accompanied by reduced fruit size^6^ except if precise recombination or genetic modifications limits the linkage drag effect.

Malate and citrate are the main organic acids in most ripe fruits^52^. In tomato, citrate has a stronger impact on consumer preferences. In this study, candidate genes potentially impacting both citrate and malate contents were identified. We also demonstrated that citrate has been more influenced by improvement sweeps and malate by domestication sweeps. These results show that citrate was probably selected for improving tomato flavor.

Flavor-related volatiles are strongly influenced by the environment^53,54^. Nevertheless this meta-analysis illustrates that it should be possible to significantly enhance the content of favorable aromas via replacement of undesirable alleles. However, unlike sugars, the undesirable alleles should be carefully chosen^6^. Cherry tomato varieties have been introduced to the market since the 1990s. Their genomes are an admixture of those of big-fruited tomatoes and *S. pimpinellifolium* species^19,55^ and may still contain a large number of favorable alleles. Thus they may serve as the most promising allele reservoir for breeding of high-flavor tomatoes.

In conclusion, we performed the first meta-analysis of genome-wide association analyses in a major vegetable and identified numerous loci involved in tomato flavor that were not identified in the three independent studies. A strong positive correlation between allele combinations and sugar content provides clues for breeding for higher sugar content. Modern cultivars have lost most of the allelic diversity for sugars, acids, and volatiles that is present within the species. Significant improvements should be achieved by replacing undesirable alleles. Taken together, our meta-analysis provides genetic insights into the genetic control of tomato flavor and gives a roadmap for flavor improvement.

## Methods

### Three GWAS panels

The meta-GWASs approach is based on three different GWAS panels already published and genotyped using different technologies. Our approach consisted in imputing SNP data for panels S^8^ and B^11^ from a reference panel, then conducting separate GWAS using the same mixed linear model (MLM) as described in^6^ and collecting the summary statistics to run a meta-GWAS.

Panel S consists of 163 accessions^8^, including 28*S. lycopersicum* (large tomato), 119*S. lycopersicum* var *cerasiforme* (cherry tomato), and 16*S. pimpinellifolium* (closest wild species). This panel was genotyped using the Solanaceae Coordinated Agricultural Project (SOLCAP) genotyping array^56,57^, generating 5995 high quality SNPs. The minimal success genotyping rate per accession was fixed at 90%. The minor allele frequency of SNPs ranged from 0.037 to 0.45. Tomato accessions in Panel S were grown in Avignon, France, following a randomized complete block design, in a greenhouse during the summers of 2007 and 2008^8,58^.

Panel B consists of 300 accessions with 62*S. pimpinellifolium*, 48*S. lycopersicum*, and 190*S*. l. *cerasiforme* accessions^11^. This panel was genotyped both with the SOLCAP^56,57^ and CBSG arrays^59^. After quality control, 9013 SNPs (minor allele frequency, MAF > 0.1) and 291 accessions were kept. Accessions in Panel B were grown in Agadir, Morocco, France, under passive greenhouse irrigated conditions in 2011 and 2012^11^. Each trial followed a randomized complete block design, with three and two blocks, in 2011 and 2012, respectively.

Panel T consists of 402 tomato accessions from two separate panels^6^. Panel T was genotyped by whole genome resequencing technology, generating a number of 2,014,488 SNPs passing quality control (MAF > 0.05, missing rate < 10%). This panel includes five tomato types, including modern (51), transitional (50), cherry (27), heirloom (243), and wild species (27)^6^.

### Phenotypes

A total of 31 flavor-related quality traits in tomato were analyzed for meta-analysis, including two sugars (fructose and glucose), two organic acids (citrate and malate), 10 amino acids, and 17 flavor-related volatiles. The 10 amino acids were asparagine, aspartate, GABA, glutamine, lysine, methionine, phenylalanine, proline, serine, and threonine. The 17 volatiles were (E)-2-heptenal (E2HEP), (E)-2-hexenal (E2HEX), (E)-2-pentenal (E2PEN), (E,E)-2,4-decadienal (EE24D), (Z)-3-hexen-1-ol (Z3H1X), (Z)-3-hexenal (Z3HEX), 1-octen-3-one (X1O3ON), 1-penten-3-one (X1P3ON), 2-methyl-1-butanol (X2M1BU), 3-methyl-1-butanol (X3M1BU), 6-methyl-5-hepten-2-one (X6MHON), beta-ionone (BIONO), geranylacetone (GRACE), guaiacol (GUAIA), hexanal (XEXAN), phenylacetaldehyde (PHEAC), and methylsalicylate (METHY).

Sugars and organic acids were measured in all three panels. Amino acids were measured both in panel S and B, while flavor-related volatiles were measured both in panel B and T. Briefly, fructose and glucose in panel S were measured using the micro-method. Citrate and malate were measured by gas chromatography-mass spectrometry (GC-MS)^8^. Data distribution was tested using the Shapiro–Wilk test and data with a non-normal distribution were Log_10_ transformed. In panel B, these metabolites were measured within the Product Metabolism and Analytical Sciences Endogenous Metabolite Profiling Platform at Syngenta Jealott’s Hill International Research Center, Bracknell, UK. Fructose and glucose were analyzed by high pH ion-exchange chromatography. Citrate and malate were analyzed using electrospray ionization-liquid chromatography (ESI-LC-MS/MS). Fructose and malate were transformed using the Boxcox method. Citrate was transformed using the Log_10_ method. In panel T, citrate and malate were measured using the citrate and malate analysis kits (R-Biopharm, Marshall, MI), according to the manufacturer’s instructions^60^. Measurements of amino acids and volatiles in panel S was measured using GC-MS by comparing with a database of authentic standards. Small organic acids and amino acids in panel B were analyzed using electrospray ionization-liquid chromatography (ESI-LC-MS/MS).Volatiles in panel T were first captured by headspace solid phase micro extraction (HS-SPME) coupled GC-MS.

### Reference panel for SNP imputation

A reference panel was selected from the 360 re-sequenced tomato accessions^19^ to perform SNP imputation in panels S and B. Among this panel, only accessions with genome coverage ≥90% and mean sequencing depth ≥4.0 were kept. Wild tomato species were also removed, generating a total reference set of 221 accessions genotyped with 3,809,156 SNPs (Supplementary Table 1).

### Recombination map

A high-density recombination map is required for imputation and computing genomic partitions. However, the available tomato genetic maps EXPIM 2012 and EXPEN 2012^57^ have a limited genomic coverage (~3500 mapped SNPs). In order to use a much denser genetic map, we developed a Python script to infer the corresponding genetic positions of the 3,809,156 SNPs in the reference panel. Before calculating the recombination rate, we first compared the physical vs genetic distribution patterns for each chromosome (Supplementary Fig. 1). Comparing with EXPIM 2012, this newly built genetic map had the same distribution pattern (Supplementary Fig. 1). This comparison indicated the inferred genetic positions were accurate and were then used for estimating the recombination rate, as required for imputation. Minor adjustments were also done for some SNPs in order to follow an overall increasing positional order. Extreme recombination rate values were also removed (>2000 cM/Mb).

### Genotype imputation

One unphased reference panel from IMPUTE2 (https://mathgen.stats.ox.ac.uk/impute/impute_v2.html#home)^22^ was adopted for imputation of panel S and B independently. The 221 filtered sequenced accessions passing quality control were used as the reference panel. The newly built recombination map was used instead of EXPIM 2012. The whole genome was then divided into genomic intervals of 5 Mb for imputation and the effective size of population (*Ne*) was set at 2000.

### Quality control

After imputation, the minimum MAF for panel S and B was set at 0.037 and 0.021, respectively, according to the formula: [Number of chromosomes/(2 × Number of individuals)]^61^. After combining all the imputed data, basic statistic summaries were obtained in QCTOOL v2 (http://www.well.ox.ac.uk/~gav/qctool_v2/) with the following command:./qctool -g GWAS.gen -snp-stats. We then filtered all imputed SNPs with Hardy-Weinberg equilibrium (HWE) ≥ 0.000001, MAF ≥ 0.037 (0.021 for panel B), missing rate ≤ 0.10 and missing call rate ≤ 0.10. After these primary control steps, a total of 224,097 and 327,436 SNPs were retained for panel S and B, respectively.

In order to determine the optimal threshold of imputation quality (Info criteria), we compared the imputed and sequenced genotype data of the nine overlapping accessions in panel S that have been genotyped by SNP arrays and whole-genome sequencing. If the maximum of the three probabilities at a locus was higher than 0.9, we treated it as a certainty. This was done by converting the imputed data to ped/map format via GTOOL (http://www.well.ox.ac.uk/~cfreeman/software/gwas/gtool.html). We then compared the imputed and genotyped values of the nine accessions (Supplementary Fig. 2). Total numbers of corrected SNPs at different MAF and Info thresholds were obtained to validate the optimal threshold of MAF and Info. The average value of Info was 0.882 (with no filtering of MAF). With the increase of Info, the number of correctly genotyped SNPs increased from less than 200 to about 50,000 for panel S (Supplementary Fig. 2a, Supplementary Table 2). On average, 51.45% of the SNPs have been correctly imputed for all Info values. There was no significant difference between the numbers of corrected imputed SNPs for different Info values of the three tomato groups (Supplementary Fig. 2b). The majority of imputed SNPs had a MAF value ranging from 0.037 to 0.25, with a mean value of 0.172 ± 0.103 (with no filtering of Info). The percentage of successfully genotyped SNPs averaged at 57.3% and a higher percentage of corrected imputed SNPs decreased gradually with the increase of MAF (Supplementary Fig. 2c). Similarly, no significant difference was found between the numbers of corrected imputed SNPs for different MAF values of three tomato genetic groups (Supplementary Fig. 2d). Details of the number and percentage of corrected imputed SNPs at different MAF bins among the nine accessions are listed in Supplementary Data 1. We than compared the relationship between MAF and Info. The average value of Info was 0.912 for all values of MAF (Supplementary Fig. 2e). We found that the lowest mean value of Info (0.622) was observed on less common SNPs (0.037 < MAF < 0.05) (Supplementary Fig. 2e, Supplementary Data 2). However, this value is still higher than the proper imputation quality threshold (0.4) in common quality control of meta-analysis of genome-wide association studies^33^. So, we decided to set the Info threshold at 0.60 as the threshold of high imputation quality.

After filtering with imputation quality threshold (Info) ≥ 0.60, total of 209,152 and 252,414 SNPs were retained for panel S and B, respectively. The mean Info value at different MAF values for panel S and B were 0.929 and 0.922, respectively (Supplementary Data 3). The lowest mean value of Info at different MAF value was 0.810 and 0.783, respectively (Supplementary Fig. 2f, Supplementary Fig. 3). These SNPs offered a much denser genomic coverage for both panel S and B (35-fold and 28-fold, respectively) (Supplementary Fig. 4). Only some large genomic gaps still remained where there were few genotyped SNPs over a long genomic region (Supplementary Fig. 4). These results indicated that all the retained SNPs had a high imputation quality and were used for further analyses.

### Linkage disequilibrium analysis

For population structure and kinship analyses, only independent SNPs (*r*^2^ < 0.2) were used. This was done in PLINK (https://www.cog-genomics.org/plink2) with: --indep-pairwise 50 5 0.2 (windows, step, r2) –maf 0.05, generating a total of 3,602 and 4,294 independent SNPs for panel S and B, respectively.

### Principal component analysis

In order to compare the genetic structure revealed before and after imputation, we performed a principal component analysis (PCA) for panels S and B, using all genotyped SNPs and independent imputed SNPs (*r*^2^ < 0.2) in PLINK: --pca. Principal component analysis showed that genotype imputation did not lead to significant differences in genetic group composition and pairwise individual distances, for all three accession classes of panel S (S.C., S.L., S.P.) (Supplementary Fig. 5a–c). For the first principal component (PC1), there were strong positive correlations (0.93, 0.82, and 0.93 for S.C, S.L., and S.P. respectively) between genotyped and imputed SNPs (only imputed SNPs) (Supplementary Fig. 5d). By combining genotyped and imputed SNPs together (hereafter called ‘All’ dataset), a similar strong positive correlation (0.94, 0.82, and 0.94 for S.C, S.L., and S.P. respectively) was also found (Supplementary Fig. 5e). Correlation between imputed and all SNPs was also strong for all tomato classes (Supplementary Fig. 5f). For the panel B, a previous study revealed a population structure composed of six groups^62^. After imputation, we found they had a similar distribution pattern (Supplementary Fig. 6). PC1 between genotyped SNPs and all (genotyped and imputed) SNPs had a strong positive correlation (higher than 0.7 for all six groups) (Supplementary Fig. 6c). In contrast, the second principal component (PC2) had strong negative correlations for all six groups (lower than −0.6 for all six groups) (Supplementary Fig. 6d).

### Population structure

In a previous study, the population structure of panel S was evaluated by Structure v2.3.4^63^ (https://web.stanford.edu/group/pritchardlab/structure_software/release_versions/v2.3.4/html/structure.html). So we first compared the structure following the same parameters, with 1 × 10^6^ burn-in period and 5 × 10^6^ MCMC steps. Based on the Evanno method^63^, the optimal number of ancestral populations was two. Only minor population assignment differences were found for both subpopulations, compared with structure from genotyped SNPs (Supplementary Fig. 7).

We further used discriminant analysis of principal components (DAPC)^64^ (http://adegenet.r-forge.r-project.org/files/tutorial-dapc.pdf) using the independent 3,602 and 4,294 SNPs (*r*^2^ < 0.2) to infer the optimal population structure for panels S and B. This method partitioned the variance within and among groups without assumptions on LD or Hardy–Weinberg equilibrium^65^, which has shown a better performance in clustering individuals^11^. The optimal number of clusters was determined by Bayesian Information Criteria (BIC) with a minor increase or decrease. All PCs and all discriminant functions were retained to find the optimal number of clusters. In the following DAPC analyses, all discriminant functions and the first 50 PCs were retained in order to achieve 80% of cumulative variance for both panel S and B.

For panel S, the optimal number of clusters was six (Supplementary Fig. 8) and DAPC revealed a clear structure of all the accessions (Supplementary Fig. 9). For panel B, the optimal number of cluster was six, which was the same as that revealed by using genotyped SNPs (Supplementary Fig. 10). Membership of each cluster was also quite similar (Supplementary Fig. 11), compared with that of genotyped SNPs (Supplementary Fig. 12). Detailed information of the membership of each cluster revealed by all independent SNPs for panels S and B is listed in Supplementary Data 4 and Supplementary Data 5, respectively. These results indicated that imputation did not cause significant differences in the genetic structure for both panels S and B. For panel T, the optimal number of clusters was five from DAPC with the first 20 PCs retained and a cross validation run of 100 times^6^.

### Genome-wide association analysis

Though SNPTEST v2.5.4 (https://mathgen.stats.ox.ac.uk/genetics_software/snptest/snptest.html#introduction) can use the imputed data from IMPUTE2 to detect associations directly, it cannot however handle too many cofactors in the model. For accessions from each panel used in this study, there is strong genetic structure. We first took one trait (malate) in panel S as an example to choose the optimal association software to perform the association tests.

In order to add kinship as a cofactor in SNPTEST, we performed a principal component analysis of the kinship calculated in SPAGeDi (http://ebe.ulb.ac.be/ebe/SPAGeDi.html) and structure in Structure v2.3.4. We then added the first 20 PCs as cofactors in the frequentist association test model in SNPTEST. In the next step, we used EMMAX (http://genetics.cs.ucla.edu/emmax/index.html) with the BN kinship matrix and DAPC results to conduct association analyses. For BN kinship calculation, the default command was used: emmax-kin -v -h -d 10. A uniform threshold (*P* = 1/*n*, *n* is the effective number of independent SNPs) was used as the genome-wide significance threshold for all three panels. The effective number of independent SNPs was calculated in Genetic type 1 Error Calculator (GEC)^66^ (http://grass.cgs.hku.hk/gec/download.php). The suggestive *p*-value for the 224,097 SNPs of panel S was 9.63 × 10^−5^ and the significant *p-value* was 4.82 × 10^−6^. For the 327,436 SNPs of panel B, the suggestive and significant p-value was 5.99 × 10^−5^ and 2.99 × 10^−6^, respectively.

After comparing the association results for malate of panel S, we found the strongest *p*-value in SNPTEST was still quite low, compared with other approaches (Supplementary Fig. 13). Results from MLMM (https://github.com/Gregor-Mendel-Institute/MultLocMixMod) and EMMAX were quite similar. So, in the following analyses, we only used SNPTEST to compute summary statistics, not for finding associations. For MLMM, this model adds the marker as co-factor using a window of 10. If too many markers are in full LD, the genetic variance calculation may be biased^24^. So, we used EMMAX for association analyses for all traits with the BN kinship matrix and DAPC results as covariance.

### Meta-analysis

A total of 788 tomato accessions and 2,316,117 SNPs from three GWAS panels were used for the final meta-analysis. Since each panel was stratified and a small number of individuals overlapped between panels (38 between panel B and S, 18 between panel S and T, 17 between panel B and T), genomic inflation factor (λ) was corrected before meta-analysis using GenABEL^61^ (http://www.genabel.org/packages/GenABEL) in R. Genomic inflation can be caused by population structure, cryptic relatedness, genotyping errors, sample size, LD, trait heritability, number of causal variants and other technical artefacts^67^. Though no adjustment is necessary when *λ* is lower or equal to one, we still corrected the standard errors of beta coefficients by applying the formula SE × $\sqrt \lambda$ in general for each individual studies to get the chi-squares to its optimal values^68^.

METAL^25^ (fixed-effect model) (https://genome.sph.umich.edu/wiki/METAL_Documentation) and METASOFT^26^ (random-effect model) (http://genetics.cs.ucla.edu/meta/) are two most commonly used meta-analysis software^13^. Meta-analysis was first performed using the inverse variance-weighted fixed-effect model in METAL^25^. The genome-wide significant *p*-value for meta-analysis was set as 4.0 × 10^−7^, except for SNPs that only appeared between panel S and B (the significant p-value was set at 2.99 × 10^−6^). For those SNPs where heterogeneity occurs (*I*^*2*^ > 25, indicating moderate heterogeneity), we used the Han and Eskin random-effects model (RE2) in METASOFT^26^. This model assumes no heterogeneity under the null hypothesis and offers greater power under heterogeneity, compared with conventional random-effect models^26^.

### Local SQLite database for LocusZoom

In order to obtain a regional zoom plot of the candidate SNPs in LocusZoom^69^ (https://genome.sph.umich.edu/wiki/LocusZoom_Standalone), a local SQLite database of tomato was required. We thus created a custom SQLite database in LocusZoom with the following steps. SNP positions in the 221 accessions of the reference panel were inserted by: dbmeister.py --db my_database.db --snp_pos my_snp_pos_file. For the gene information, we first downloaded the gene annotation file from Solgenomics (ftp://ftp.solgenomics.net/genomes/Solanum_lycopersicum/annotation/ITAG2.4_release/). We then converted it to genePred file format by gff3ToGenePred (http://hgdownload.cse.ucsc.edu/admin/exe/). Gene names were replaced with short codes instead of providing full names to avoid long names and overlapping. We then inserted the gene information by the following command line: dbmeister.py --db my_database.db --refflat my_refflat_file. For the recombination file, we used the recombination map previously inferred and inserted the data into our database by: dbmeister.py --db my_database.db --snp_set my_snpset_file. We used the 221 reference panel to calculate the linkage disequilibrium (LD) in PLINK by the following parameter: --ld-snp my.snp --ld-window-kb 100000 --ld-window 1000 --r2 --ld-window-r2 0 (windows, step, r2).

### LD in candidate gene regions

In order to define the window size of the candidate genes, we first calculated the LD around the significant associated SNP with the window size of 5 Mb in PLINK with the following command line: --ld-window-kb 500000 --ld-window 1000 --r2 --ld-window-r2 0 (windows, step, r2). We then chose LD higher than 0.5 as the threshold of LD decay for the candidate gene region sizes. Within the regions, we chose the candidate genes based on both the distance of the peak SNP as well as the closest genes with known functions related to the trait. If no gene fell in the candidate regions, we provided the closest gene. We further crosschecked the candidate gene expression patterns using the Tomato Expression Atlas^70^ (http://tea.solgenomics.net/expression_viewer/input).

### Group re-definition of panel T

The relationship between allele combinations and flavor-related metabolites (sugars, organic acids and volatiles) was only based on panel T. For the accessions in panel T, they were previously defined as five clusters, namely *S. lycopersicum* var *cerasiforme*, heirloom, transitional, modern and the closest wild species *S. pimpinellifolium* tomato accessions^6^. However, there were up to 11 accessions with duplicated individual IDs (Supplementary Data 10) and we cross-checked these duplicated lines and only kept one. In addition, some accessions in the group of heirloom, modern and transitional were labeled inappropriately based on the DAPC analysis. In order to correct for this, we generated the principal component analysis (PCA) based on independent SNPs (LD = 0.1) (Supplementary Fig. 125). Based on PCA, some heirloom accessions are mixed with modern accessions and were labeled as heir_mod (heirloom and modern). For the remaining heirloom accessions, they were combined with transitional accessions and labeled as heir_trans (heirloom and transitional) (Supplementary Fig. 126). The accessions of panel T were thus re-defined as four clusters, namely *S. lycopersicum* var *cerasiforme*, (cerasiforme, 26 members), heirloom and modern (heir_mod, 196 members), heirloom and transitional (heir_trans, 138 members), and *S. pimpinellifolium* (27 members) (Supplementary Data 10–11). These redefined groups were then used for allelic combination analyses. Statistical tests were only performed for those allele combinations with at least two observations (either labeled with letters or with *p*-values).

### Reporting summary

Further information on experimental design is available in the Nature Research Reporting Summary linked to this article.

## Supplementary information

Supplementary Information

Peer Review

Reporting Summary

Description of Additional Supplementary Files

Supplementary Data 1

Supplementary Data 2

Supplementary Data 3

Supplementary Data 4

Supplementary Data 5

Supplementary Data 6

Supplementary Data 7

Supplementary Data 8

Supplementary Data 9

Supplementary Data 10

Supplementary Data 11

Source Data

## Data Availability

Data supporting the findings of this work are available within the paper and its Supplementary Information files. All new meta-analysis data associated with the paper are available in a repository [10.15454/TWFDYW]. The source data underlying Figs. 2c–g, 3c–h, and 4g–i and Supplementary Figs. 5a–f, 6a–d, and 124–126 are provided as a Source Data file. Additional datasets generated and analyzed during the current study are available from the corresponding author on reasonable request.
