# Supplementary material for: Meta-analysis of genome-wide association studies provides insights into genetic control of tomato flavor
Source: Nat Commun. 2019 Apr 4;10:1534. doi: 10.1038/s41467-019-09462-w (PMC6449550; doi:10.1038/s41467-019-09462-w)
Supplement: Supplementary file 1 — Supplementary Information [file 41467_2019_9462_MOESM1_ESM.pdf]

Meta-analysis of genome-wide association studies provides new insights into genetic control of tomato flavor

Zhao *et al*.

Supplementary Table 1. Mean and standard error of imputation info.

MAF	No. of SNPs	Mean info	SD info
0.050	27724	0.6221	0.2628
0.075	17990	0.7525	0.1965
0.100	9608	0.8639	0.0853
0.125	17185	0.9197	0.0702
0.150	31414	0.9436	0.0536
0.175	29102	0.9418	0.0536
0.200	16478	0.9253	0.0645
0.225	11171	0.9289	0.0527
0.250	15136	0.9659	0.0424
0.275	6106	0.9248	0.0373
0.300	5640	0.9496	0.0358
0.325	11673	0.9628	0.0294
0.350	3152	0.9433	0.0366
0.375	5370	0.9560	0.0264
0.400	2213	0.9535	0.0242
0.425	1810	0.9543	0.0228
0.450	2265	0.9494	0.0247
0.475	1963	0.9401	0.0261
0.500	2108	0.9367	0.0260
Mean	11479.3684	0.9123	0.0616

This analysis was done based on the nine accessions appeared both in the reference and panel S across different MAF bins after filtering with MAF $\geqslant$ 0.037, HWE $\geqslant$ 0.000001, missing $\leqslant$ 0.1 and missing_call $\leqslant$ 0.1 and Info $\geqslant$ 0.60.

Supplementary Table 2. Mean and standard error of imputation info for Panel S and B.

	Panel S			Panel B		
MAF	No. of SNPs	Mean info	SD info	No. of SNPs	Mean info	SD info
0.050	15855	0.8103	0.1314	30874	0.7834	0.1360
0.075	15150	0.8242	0.0973	19803	0.8512	0.1048
0.100	9762	0.8742	0.0717	9754	0.8669	0.0717
0.125	17547	0.9224	0.0683	6462	0.8706	0.1006
0.150	31929	0.9447	0.0533	9183	0.9349	0.0468
0.175	29897	0.9434	0.0537	9329	0.9223	0.0723
0.200	17278	0.9288	0.0649	20899	0.9694	0.0439
0.225	11905	0.9333	0.0538	28911	0.9646	0.0358
0.250	15806	0.9673	0.0421	18438	0.9387	0.0555
0.275	6491	0.9293	0.0403	10637	0.9481	0.0492
0.300	5885	0.9517	0.0365	6291	0.9263	0.0552
0.325	11855	0.9633	0.0295	20592	0.9741	0.0351
0.350	3328	0.9463	0.0378	6208	0.9451	0.0400
0.375	5527	0.9573	0.0270	7087	0.9601	0.0333
0.400	2341	0.9558	0.0290	7912	0.9561	0.0281
0.425	1948	0.9576	0.0249	8019	0.9535	0.0313
0.450	2378	0.9518	0.0264	11151	0.9672	0.0281
0.475	2082	0.9435	0.0289	14826	0.9736	0.0263
0.500	2188	0.9390	0.0282	6038	0.9667	0.0281
Sum	209152	-	-	252414	-	
Mean	11008.000000	0.9286	0.0497	13284.947368	0.9218	0.0940
Min	1948	0.8103	0.0249	6038	0.7834	0.0263
Max	31929	0.9673	0.1314	30874	0.9741	0.1360

This analysis was done based on all accessions in panel S and B across different MAF bins after filtering with HWE ⩾ 0.000001, missing ⩽ 0.1 and missing_call ⩽ 0.1 and Info ⩾ 0.60. The MAF filtering threshold for Panel S and B was 0.037 and 0.021, respectively.

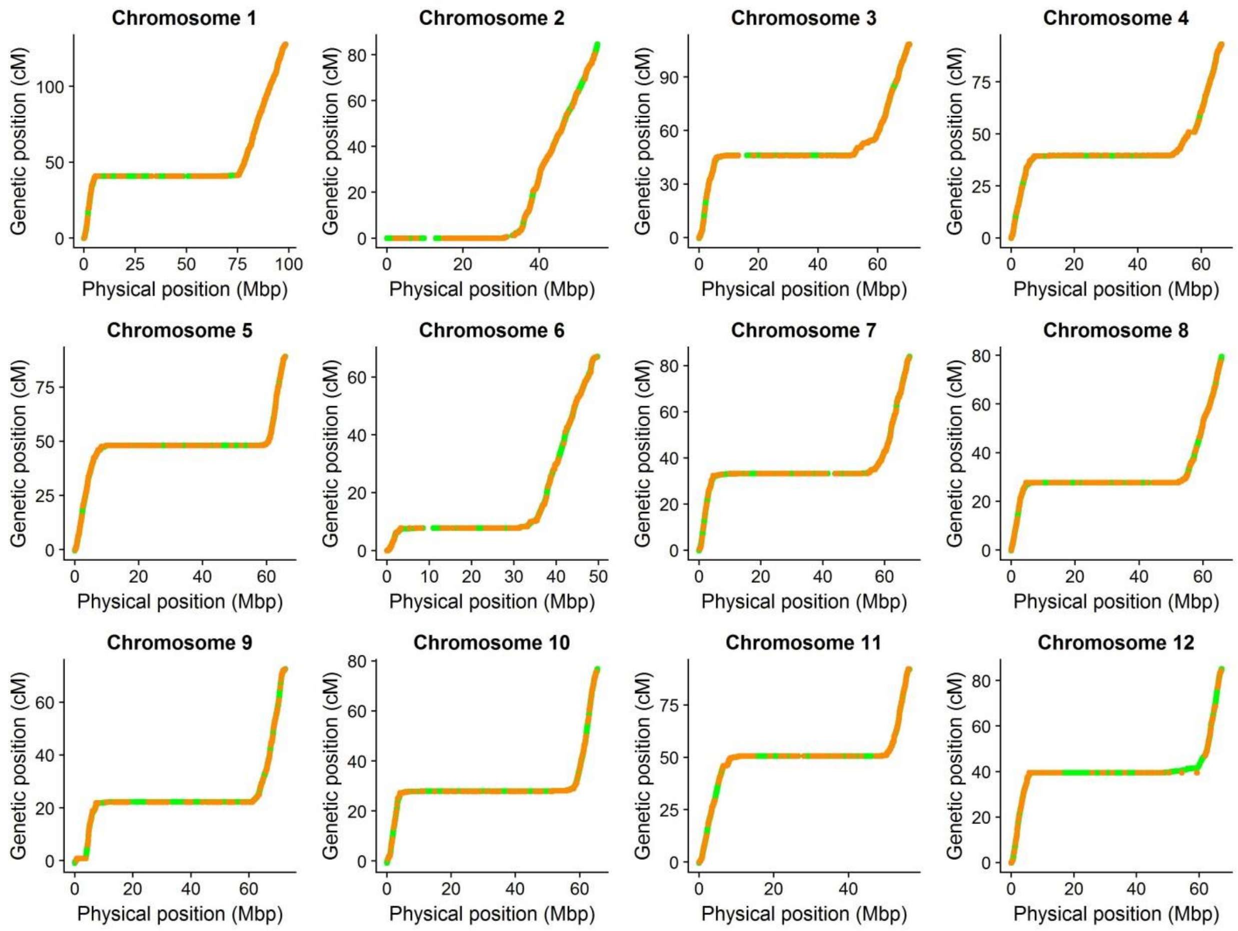

Supplementary Figure 1. Relationship between the inferred genetic and the physical positions on each chromosome. The genetic and physical relationships of SNPs in EXPIM 2012 are indicated by orange circles. The genetic positions of the 3,809,156 SNPs in the reference panel were inferred based on the relationship of the physical and genetic positions of EXPIM 2012 and the corresponding physical position on the reference. The physical position and inferred genetic positions of SNPs in the reference panel are indicated in green circles.

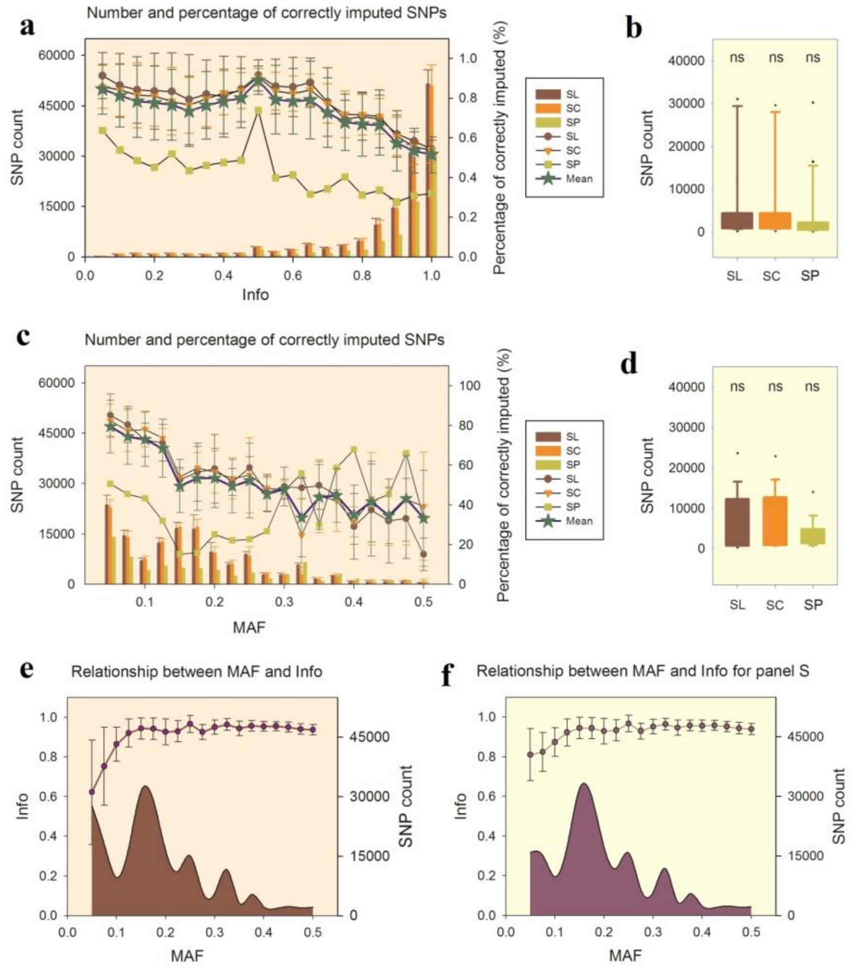

Supplementary Figure 2. Relationship between imputation quality Info and MAF. This relationship was evaluated among different tomato classes (2 *S. lycopersicum*, 6 *S.l.* var *cerasiforme* and 1 *S. pimpinellifolium*) that are in common both in the reference panel and GWAS panel S. Primary quality control steps were done before analysis with Hardy-Weinberg equilibrium (HWE) ≥ 0.000001, MAF ≥ 0.037, missing rate ≤ 0.10 and missing call rate ≤ 0.10. (**a**) The number and percentage of correctly imputed SNPs at different Info values. Each bar represents the number of correctly imputed SNPs. This was done by comparing the maximum of the three probabilities at a locus that was higher than 0.9 with the sequenced genotyping calls. (**b**) Significant t-test of the number of correctly imputed SNPs for three tomato classes at different Info values. (**c**) The number and percentage of correctly imputed SNPs at different MAF values. (**d**) Significant t-test of the number of correctly imputed SNPs for three tomato classes at different MAF values. (**e**) Relationship between MAF and Info for the nine accessions. Smooth line with stars represents the number of SNPs at different MAF values. Line plot with error bars represents the mean and standard error of Info values at different MAF values. (**f**) Relationship between MAF and Info values for all accessions of panel S after genotype imputation quality control. Error bars stand for the stand error.

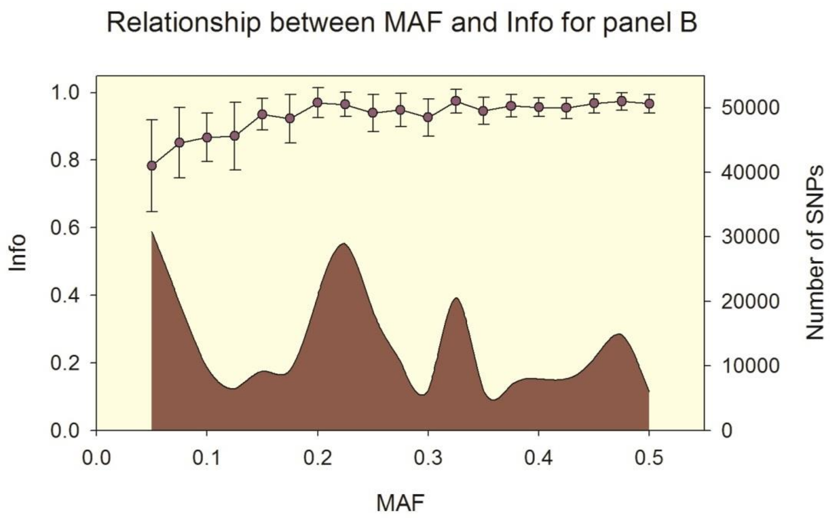

Supplementary Figure 3. Relationship between imputation quality Info criteria and MAF for panel B. Main quality control include Hardy-Weinberg equilibrium (HWE) ≥ 0.000001, MAF ≥ 0.021, missing rate ≤ 0.10 and missing call rate ≤ 0.10. Smooth line represents the number of SNPs at different MAF values. Line plot with error bars represents the mean and standard error of Info values at different MAF values. Error bars stand for the stand error.

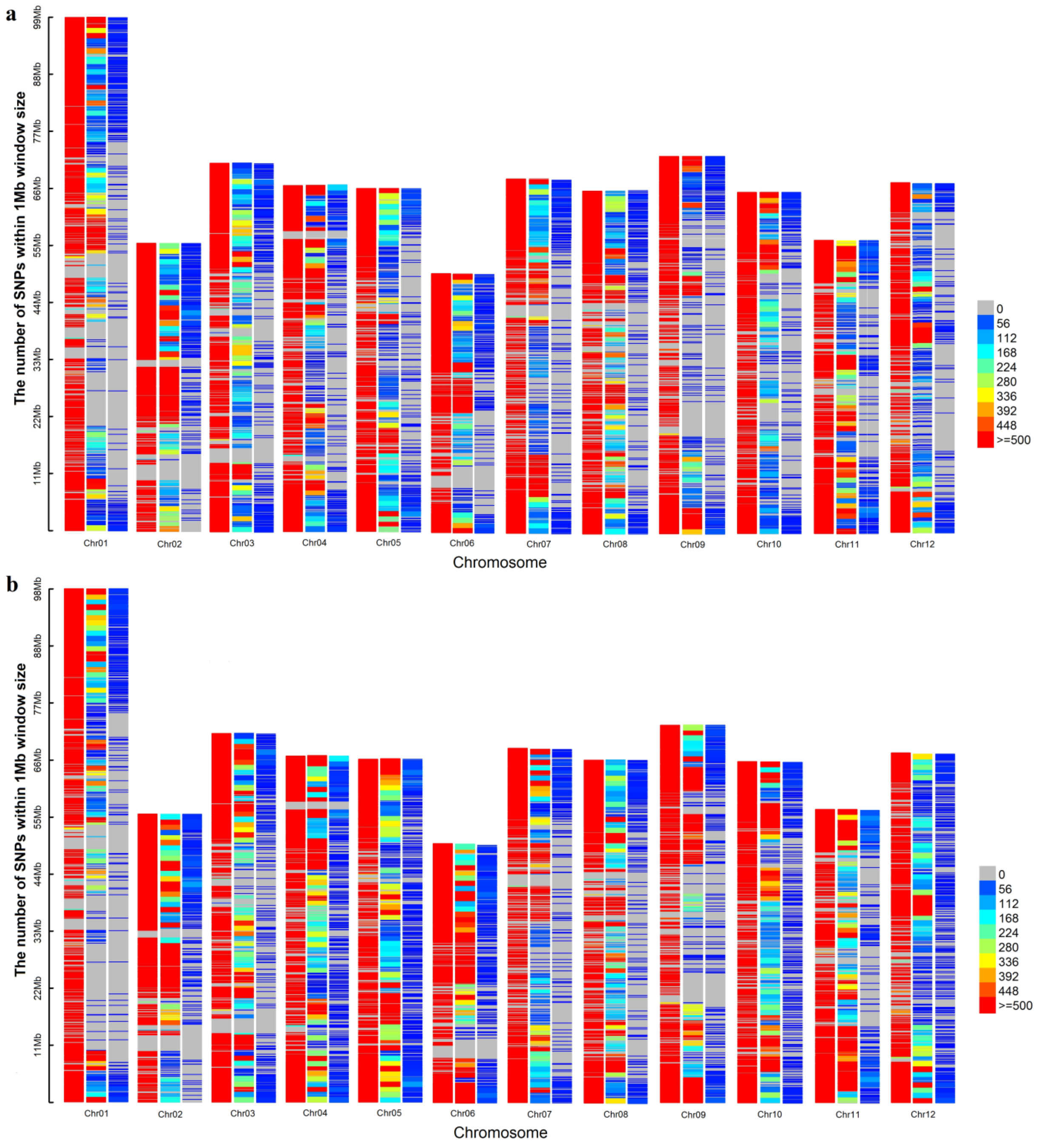

Supplementary Figure 4. Physical positions of SNPs on each. Color legend indicates the density of SNPs on 12 chromosomes. (**a**) Physical positions of SNPs on each chromosome before (right) and after imputation (middle) and the reference panel (left) for panel S. (**b**) Physical positions of SNPs on each chromosome before (right) and after imputation (middle) and the reference panel (left) for panel B.

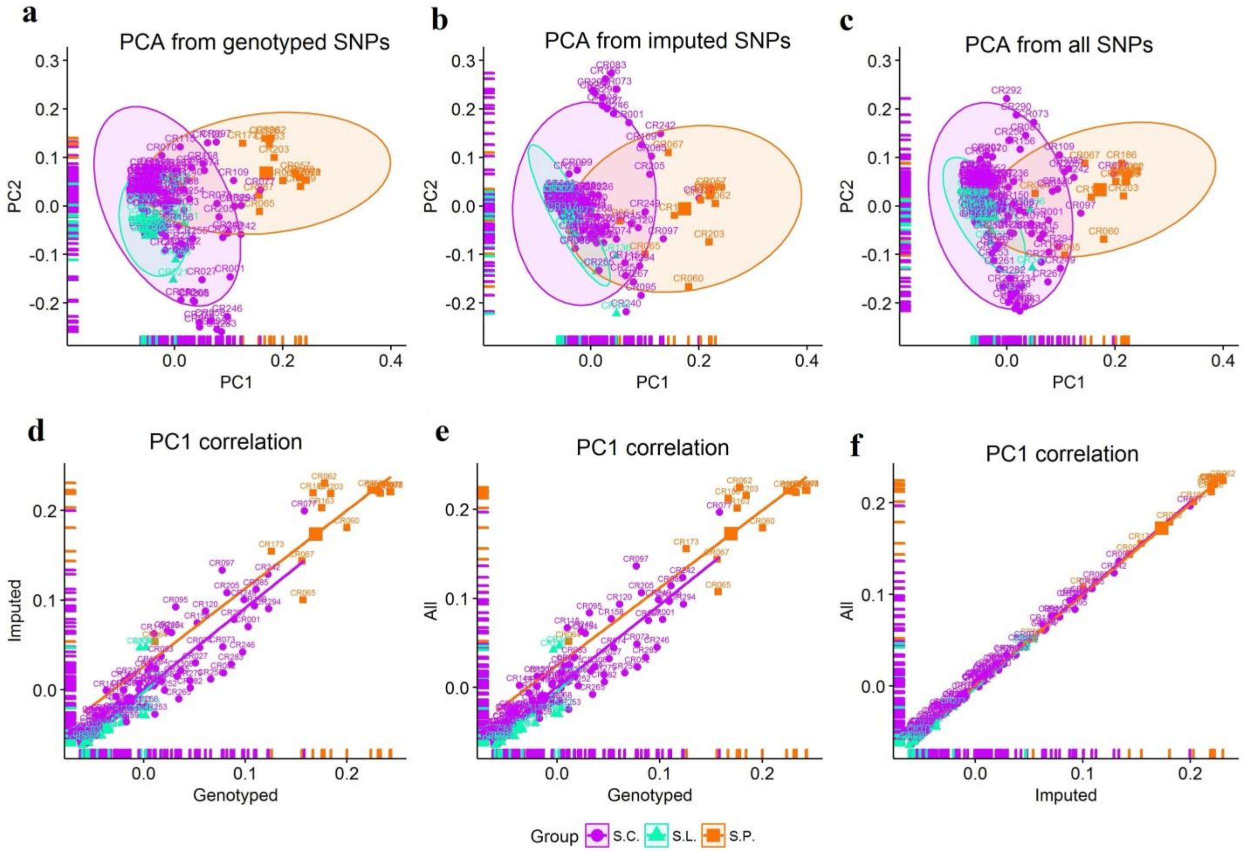

Supplementary Figure 5. Principal component analysis comparison before and after genotype imputation for panel S. (**a**) PCA revealed by genotyped SNPs. (**b**) PCA revealed by independent imputed SNPs ($r^2 \leq 0.2$). (**c**) PCA revealed by all independent genotyped plus imputed SNPs ($r^2 \leq 0.2$). (**d**) Correlation between the first principal component of genotyped and independent imputed SNPs. (**e**) Correlation between the first principal component of genotyped and all independent genotyped plus imputed SNPs. (**f**) Correlation between the first principal component of independent imputed and all independent genotyped plus imputed SNPs. S.C., *Solanum lycopersicum*; S.C., *S. lycopersicum* var *cerasiforme* and S.P., *S. pimpinellifolium*. Source data of Supplementary Figure 5a-f are provided as a Source Data file.

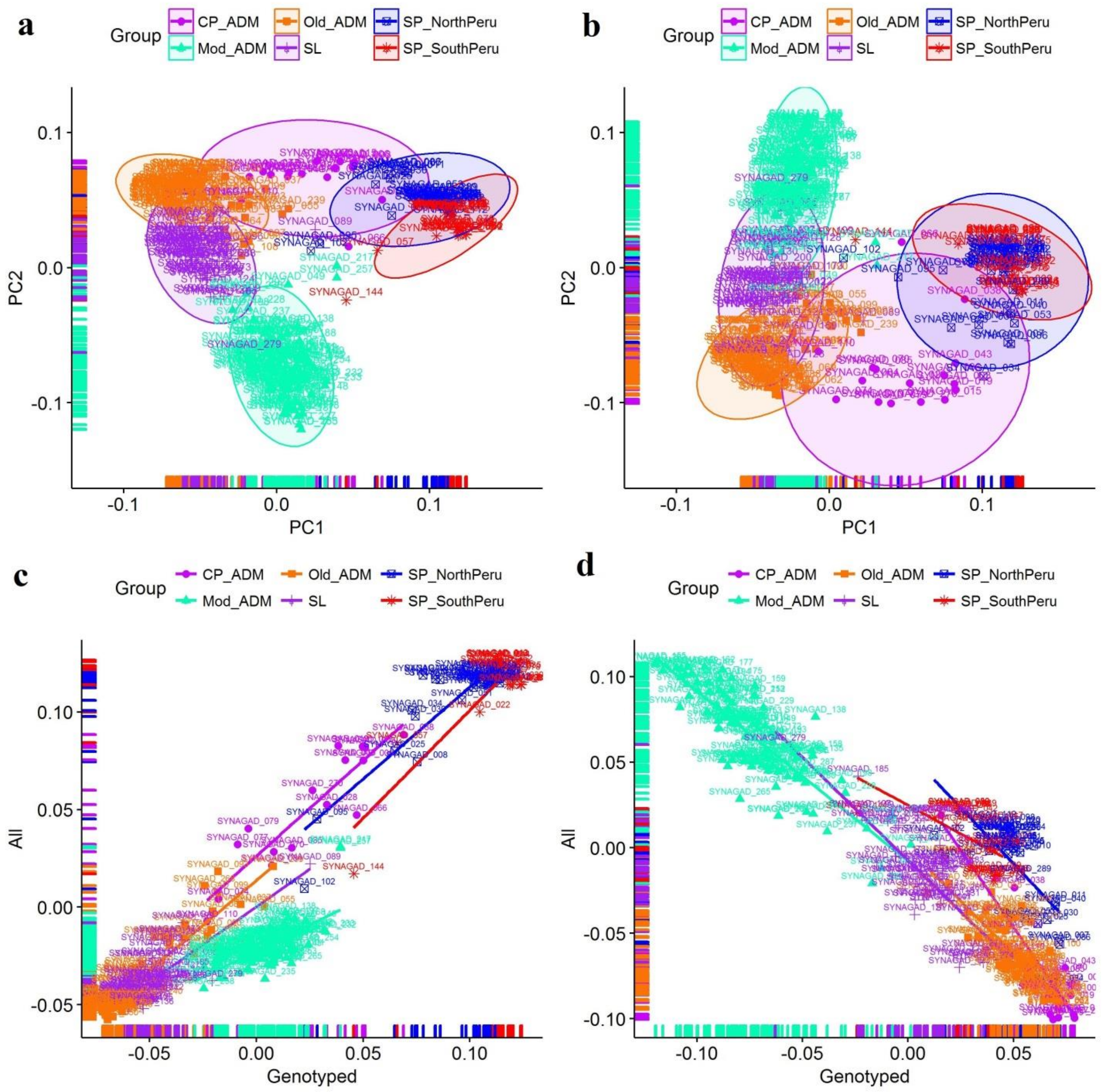

Supplementary Figure 6. Principal component analysis comparison before and after genotype imputation for the six groups previously defined for panel B. (**A**) PCA revealed by genotyped SNPs. (**B**) PCA revealed by all (genotyped and imputed) SNPs ($r^2 \leq 0.2$). (**C**) Correlation between the first principal component of genotyped and all SNPs. (**D**) Correlation between the second principal component of genotyped and all SNPs. Source data of Supplementary Figure 6a-d are provided as a Source Data file.

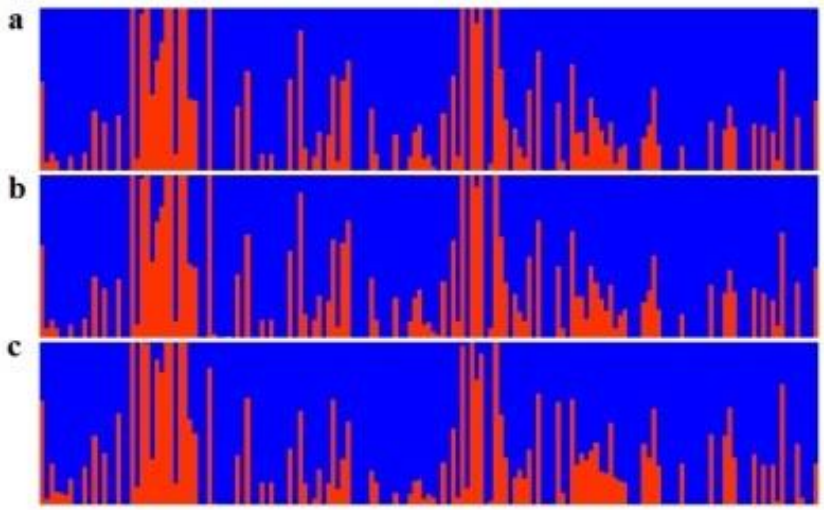

Supplementary Figure 7. Population structure of panel S. Each bar represents one individual. (**a**) Population structure revealed by independent imputed SNPs. (**b**) Population structure revealed by all independent (imputed and genotyped) SNPs. (**c**) Population structure revealed by genotyped SNPs.

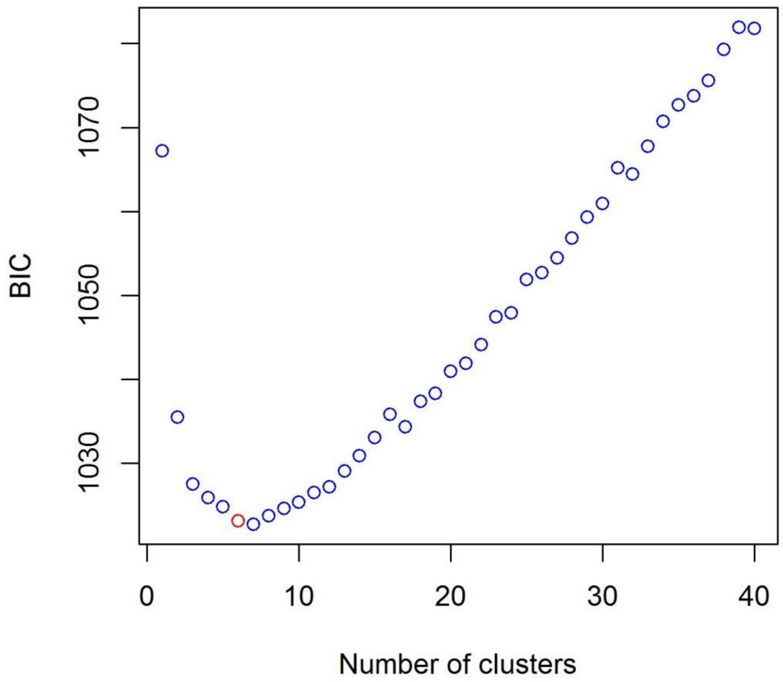

Supplementary Figure 8. Relationship between Bayesian Information Criteria and the number of clusters revealed by all independent SNPs for panel S. Red circle indicates the chosen number of clusters (6).

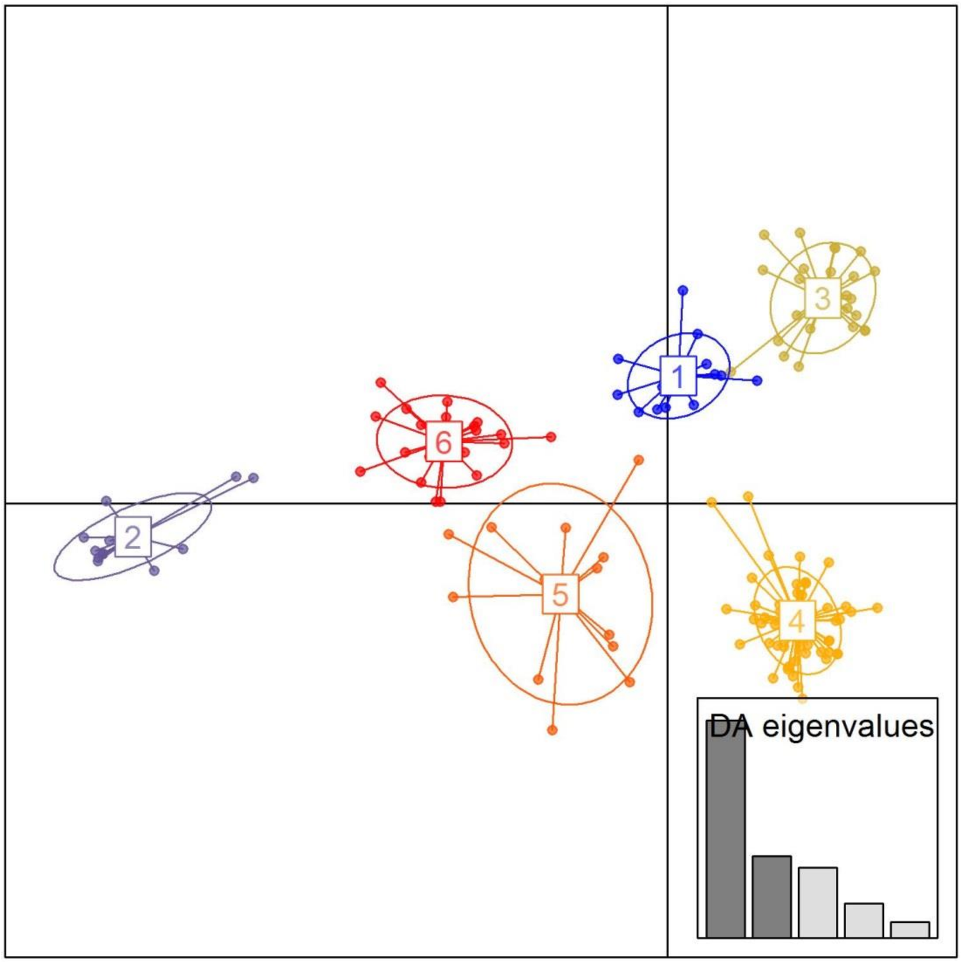

Supplementary Figure 9. Population structure revealed by discriminant analysis of principal components based on all independent SNPs of panel S.

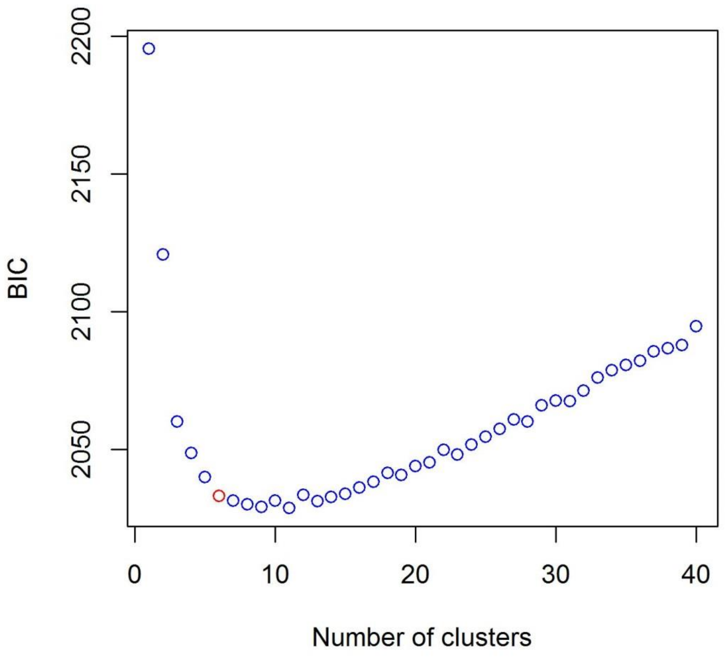

Supplementary Figure 10. Relationship between Bayesian Information Criteria and the number of clusters revealed by all independent SNPs for panel B. Red circle indicates the chosen number of clusters (6).

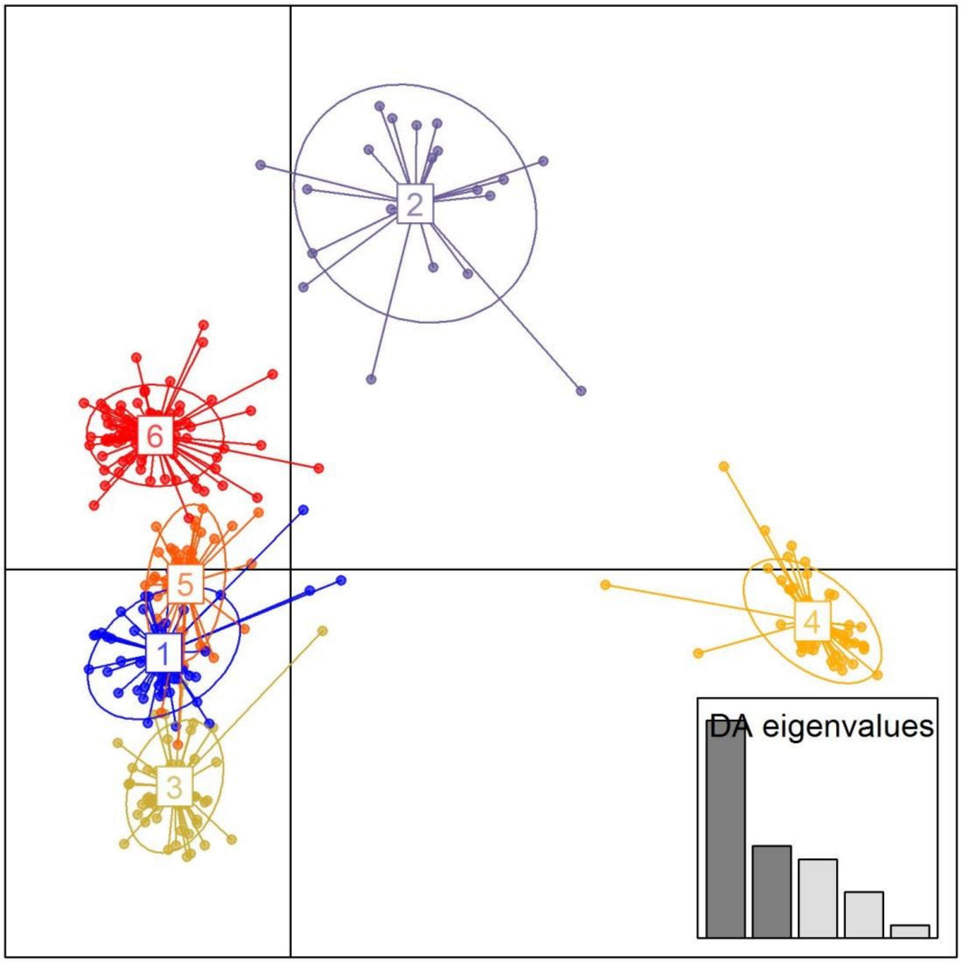

Supplementary Figure 11. Population structure revealed by discriminant analysis of principal components based on all independent SNPs of panel B.

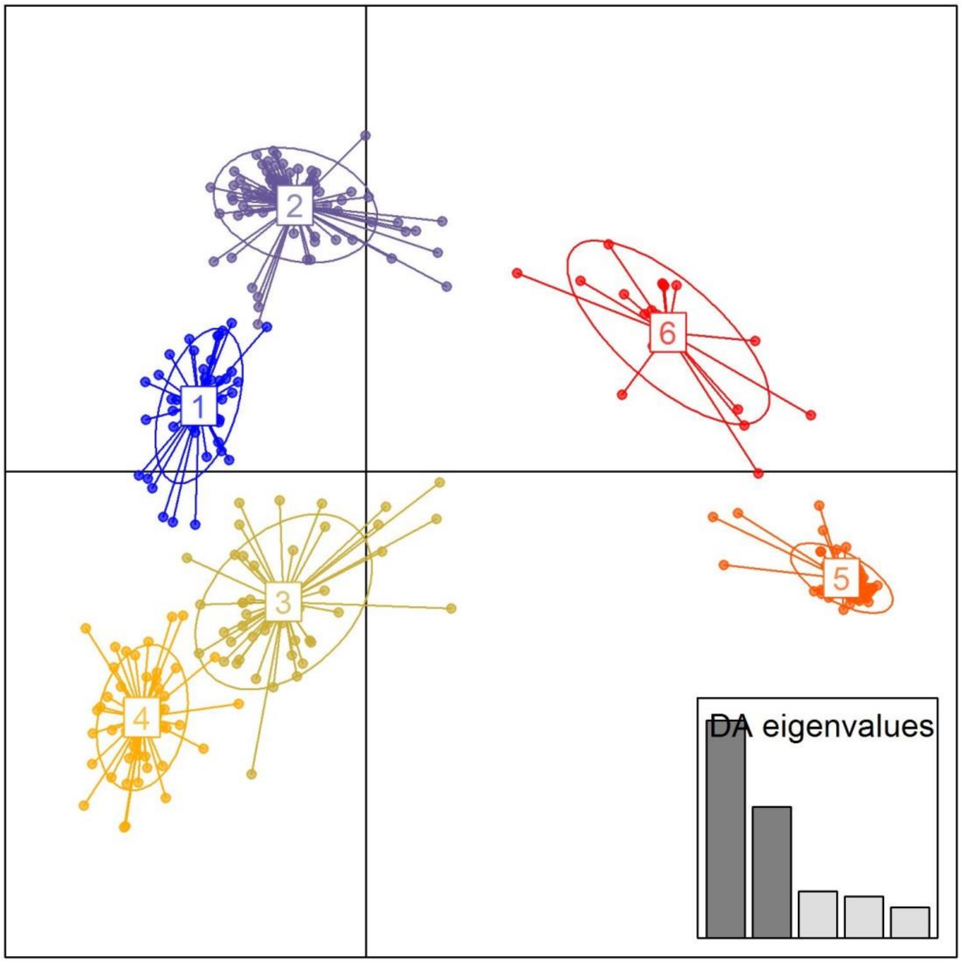

Supplementary Figure 12. Population structure revealed by discriminant analysis of principal components based on all genotyped SNPs of panel B.

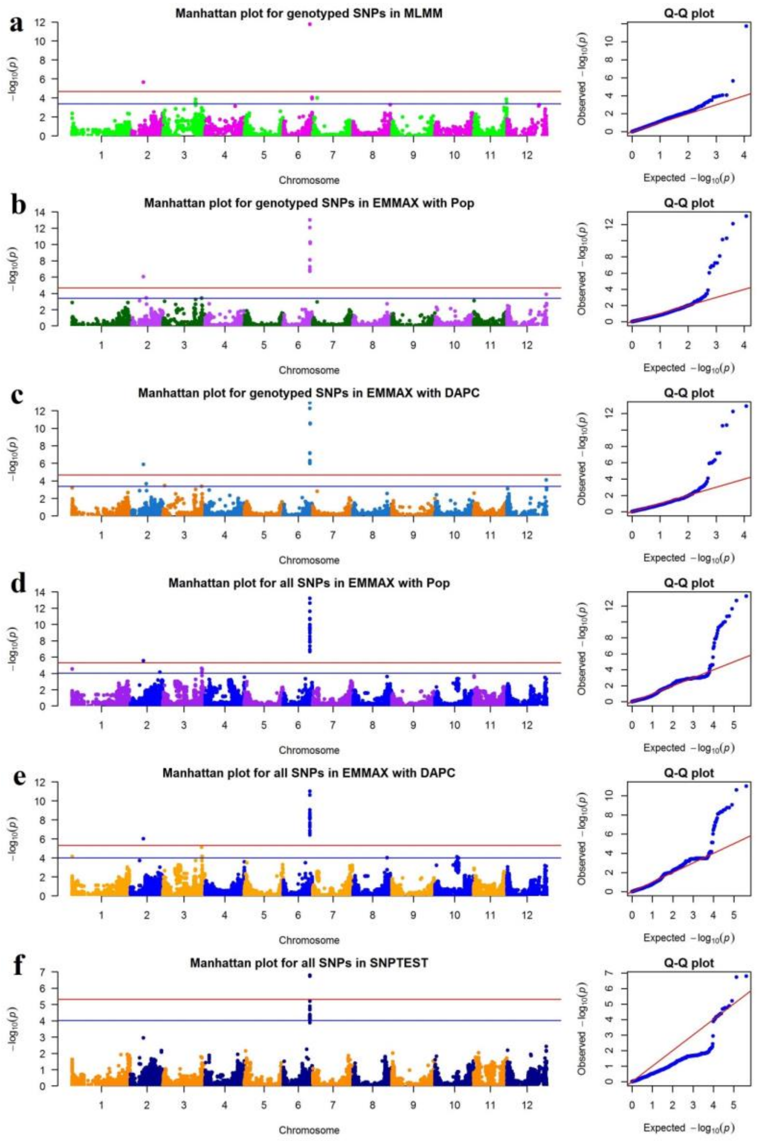

Supplementary Figure 13. Comparison of different GWAS approaches using malate from panel S as an example. (**a**) Manhattan and quantile-quantile (Q-Q) plot for GWAS of genotyped SNPs in multi-locus mixed model (MLMM). (**b**) Manhattan and Q-Q plot for GWAS of genotyped SNPs in efficient mixed-model association expedited (EMMAX) with the cofactor of structure revealed by Structure v2.3.4. (**c**) Manhattan and Q-Q plot for GWAS of genotyped SNPs in EMMAX with the cofactor of structure revealed by discriminant analysis of principal components (DAPC). (**d**) Manhattan and Q-Q plot for GWAS of all (imputed and genotyped) SNPs in EMMAX with the cofactor of structure revealed by Structure v2.3.4. (**e**) Manhattan and Q-Q plot for GWAS of all (imputed and genotyped) SNPs in EMMAX with the cofactor of structure revealed by DAPC. (**f**) Manhattan and Q-Q plot for GWAS of all (imputed and genotyped) SNPs in SNPTEST with the cofactor of the first 20 principal components of kinship and structure.

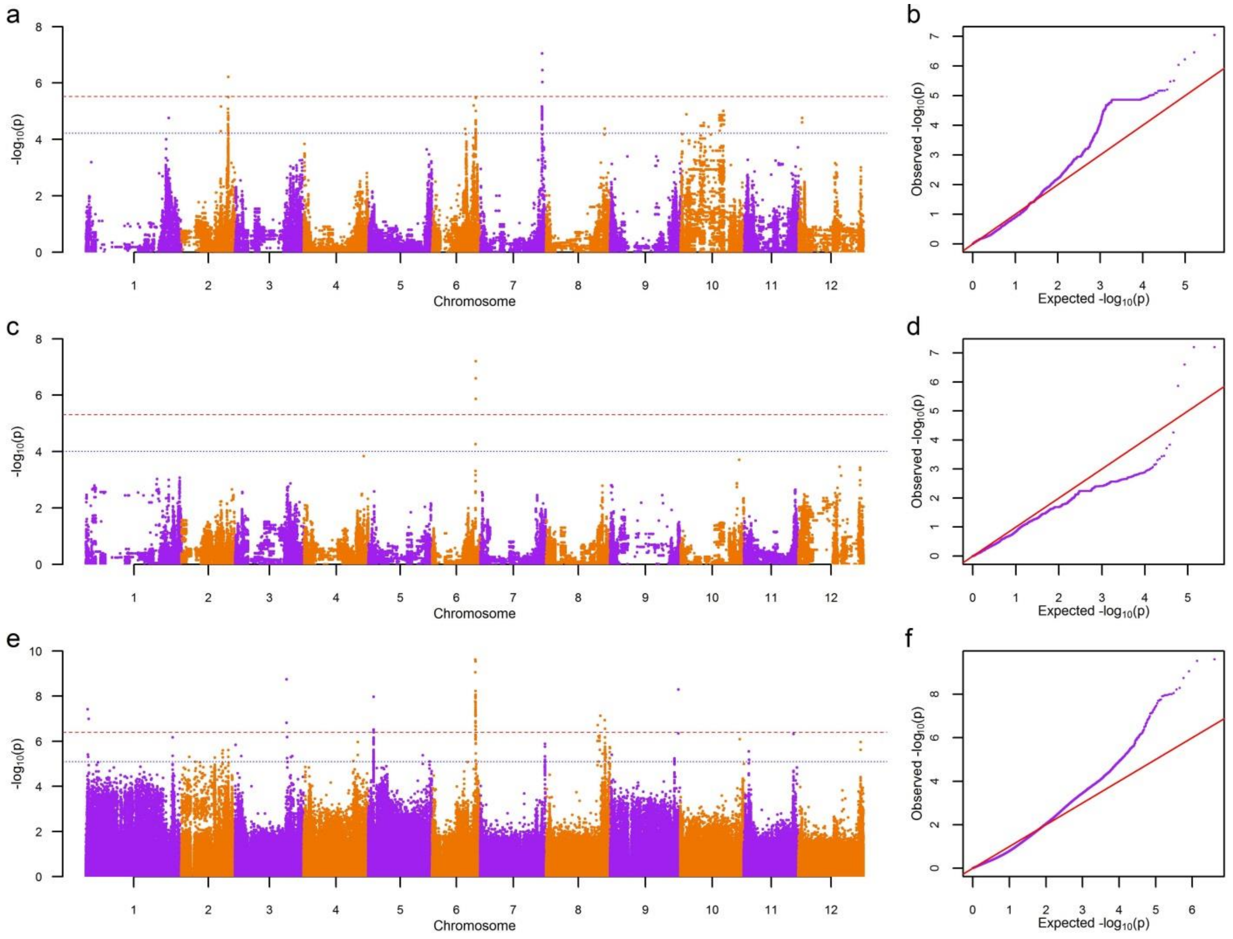

Supplementary Figure 14. Genome-wide association analysis of citrate from EMMAX. (**a**) Manhattan plot of panel B. (**b**) Quantile-quantile plot of panel B. (**c**) Manhattan plot of panel S. (**d**) Quantile-quantile plot of panel S. (**e**) Manhattan plot of panel T. (**f**) Quantile-quantile plot of panel T.

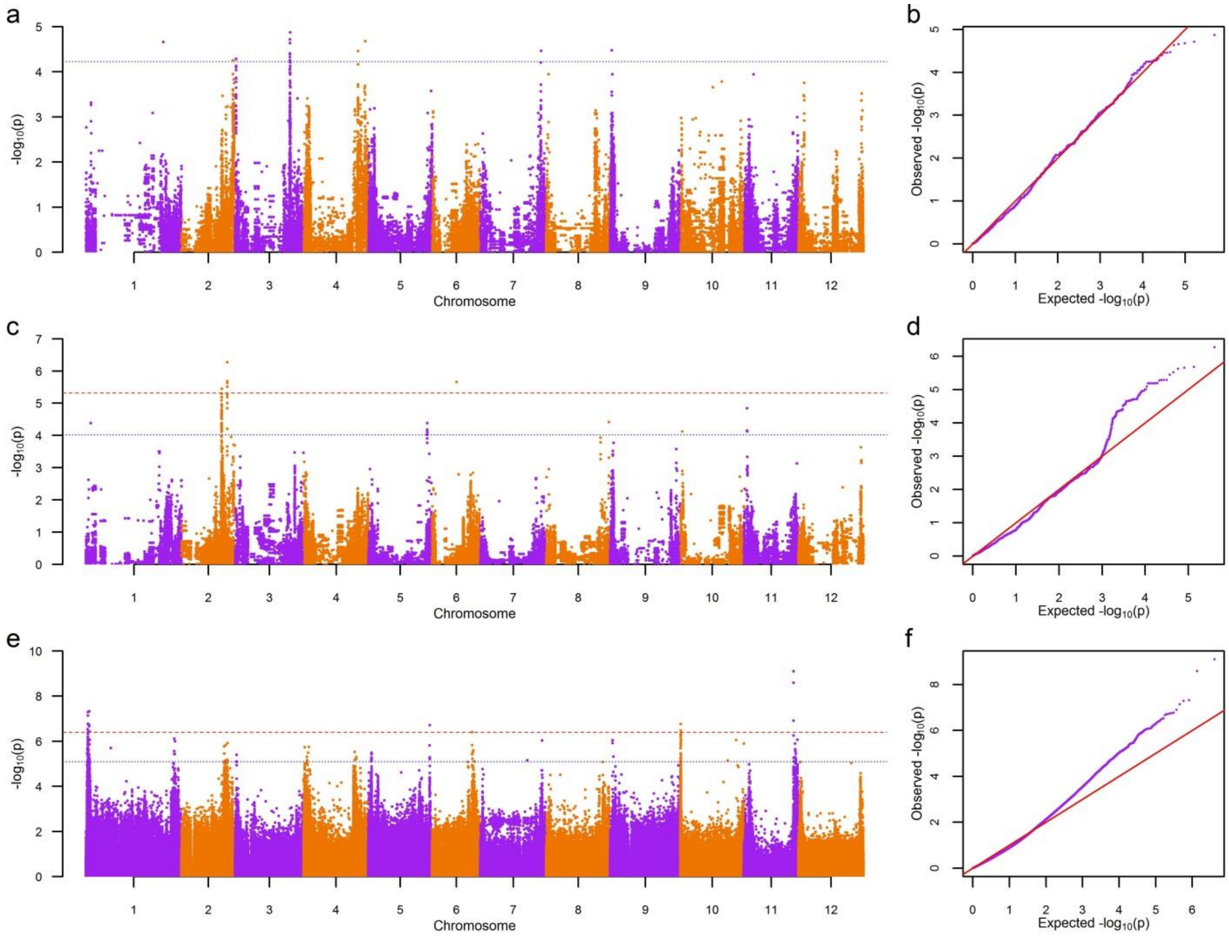

Supplementary Figure 15. Genome-wide association analysis of fructose from EMMAX. (**a**) Manhattan plot of panel B. (**b**) Quantile-quantile plot of panel B. (**c**) Manhattan plot of panel S. (**d**) Quantile-quantile plot of panel S . (**e**) Manhattan plot of panel T. (**f**) Quantile-quantile plot of panel T.

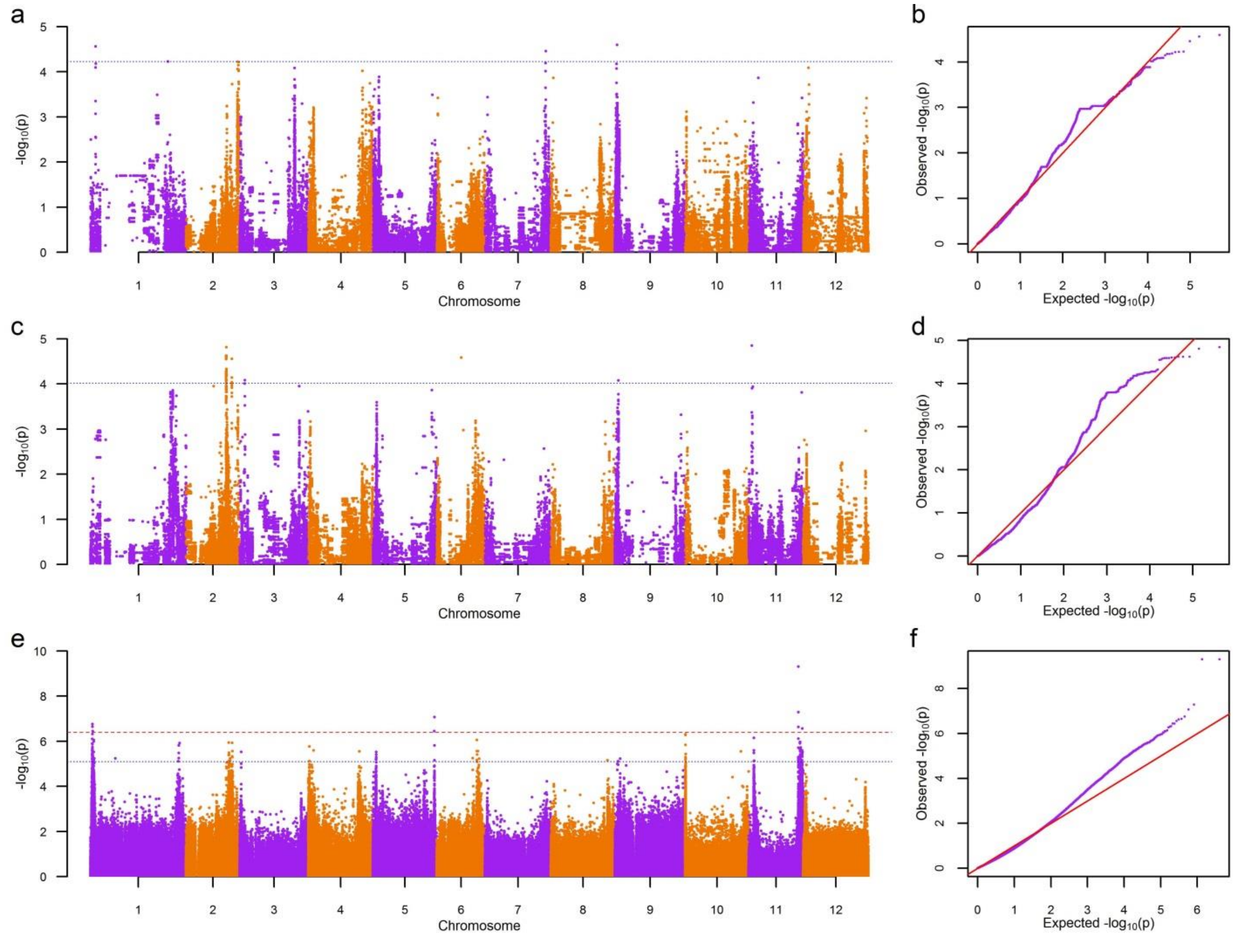

Supplementary Figure 16. Genome-wide association analysis of glucose from EMMAX. (**a**) Manhattan plot of panel B. (**b**) Quantile-quantile plot of panel B. (**c**) Manhattan plot of panel S. (**d**) Quantile-quantile plot of panel S. (**e**) Manhattan plot of panel T. (**f**) Quantile-quantile plot of panel T.

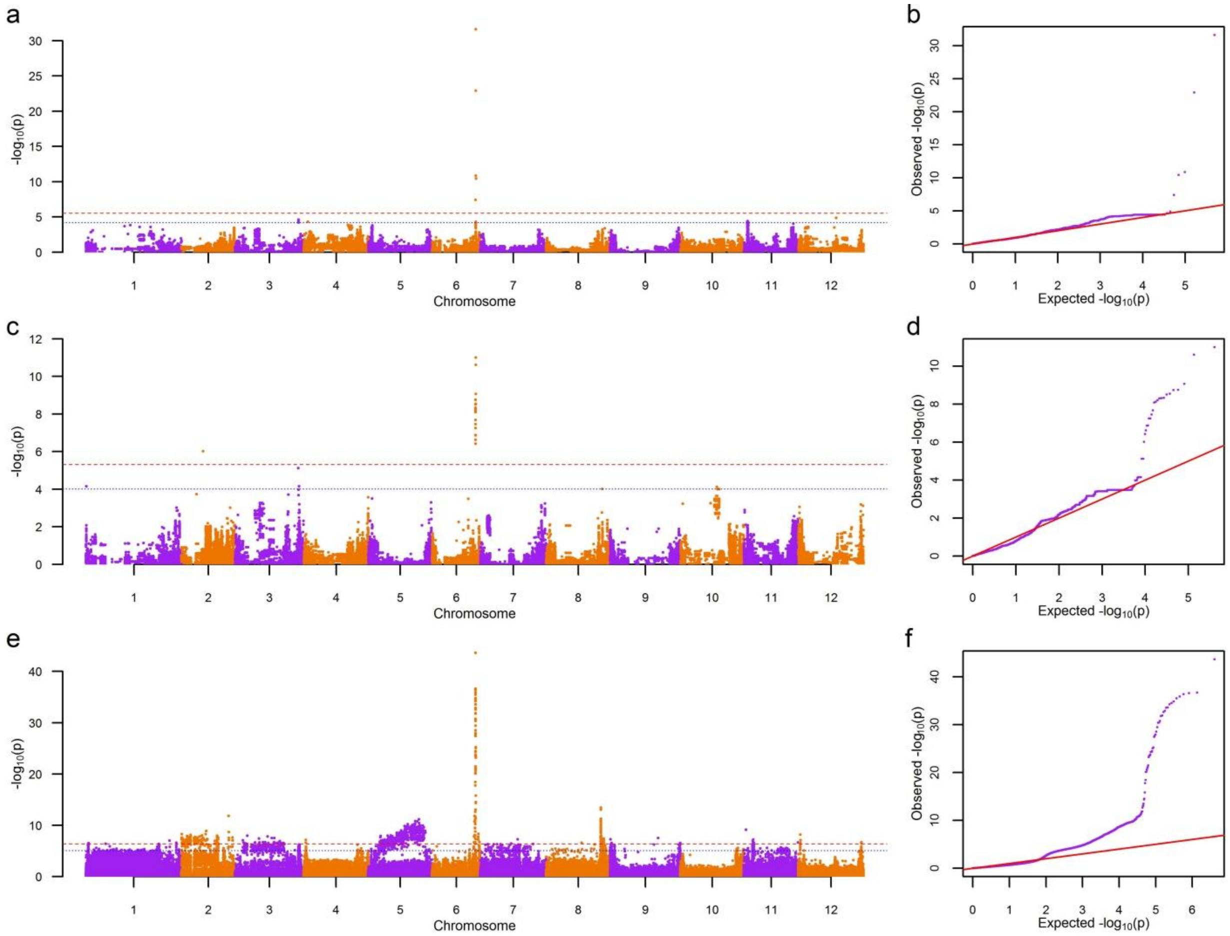

Supplementary Figure 17. Genome-wide association analysis of malate from EMMAX. (**a**) Manhattan plot of panel B. (**b**) Quantile-quantile plot of panel B. (**c**) Manhattan plot of panel S. (**d**) Quantile-quantile plot of panel S. (**e**) Manhattan plot of panel T. (**f**) Quantile-quantile plot of panel T.

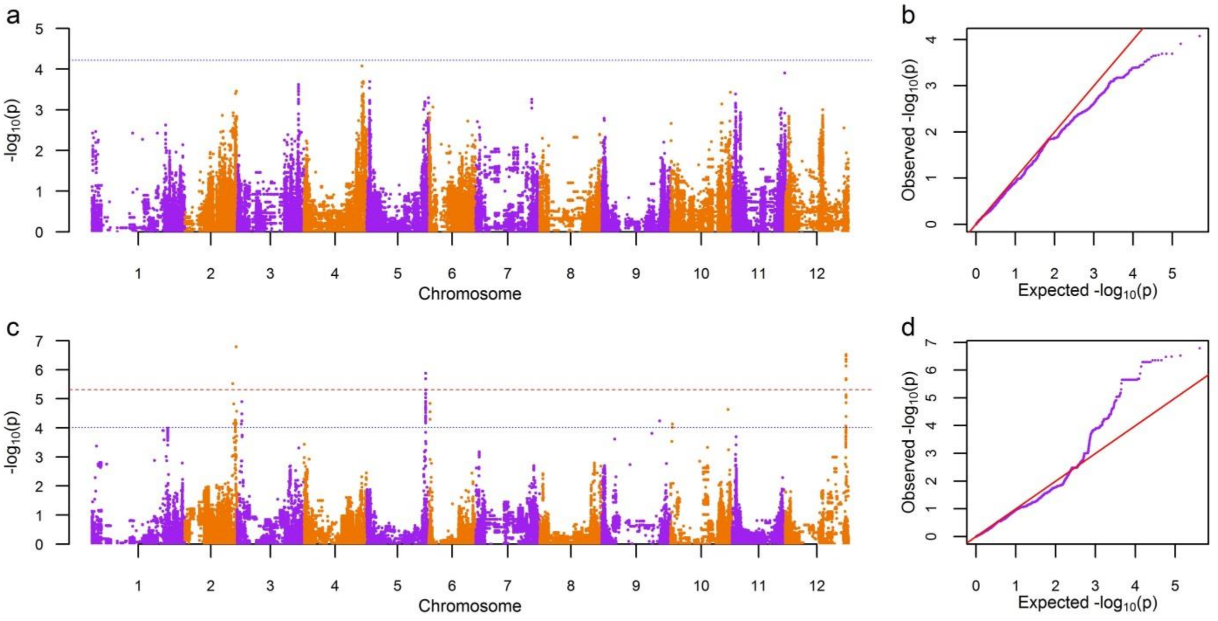

Supplementary Figure 18. Genome-wide association analysis of asparagine from EMMAX. (**a**) Manhattan plot of panel B. (**b**) Quantile-quantile plot of panel B. (**c**) Manhattan plot of panel S. (**d**) Quantile-quantile plot of panel S.

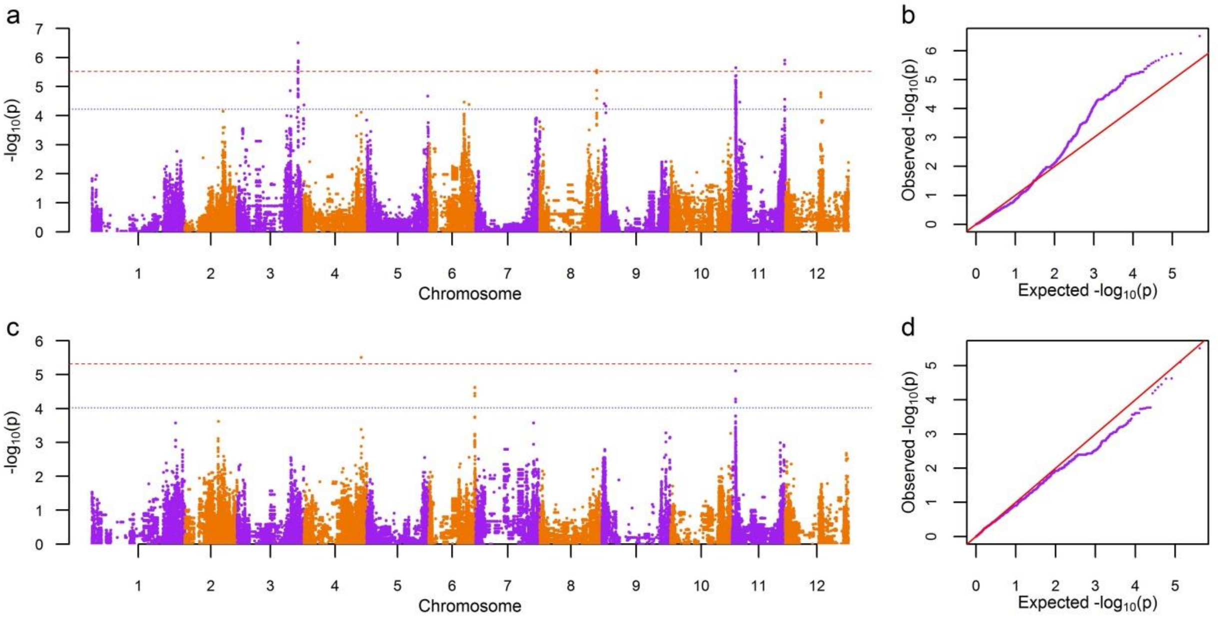

Supplementary Figure 19. Genome-wide association analysis of aspartate from EMMAX. (**a**) Manhattan plot of panel B. (**b**) Quantile-quantile plot of panel B. (**c**) Manhattan plot of panel S. (**d**) Quantile-quantile plot of panel S.

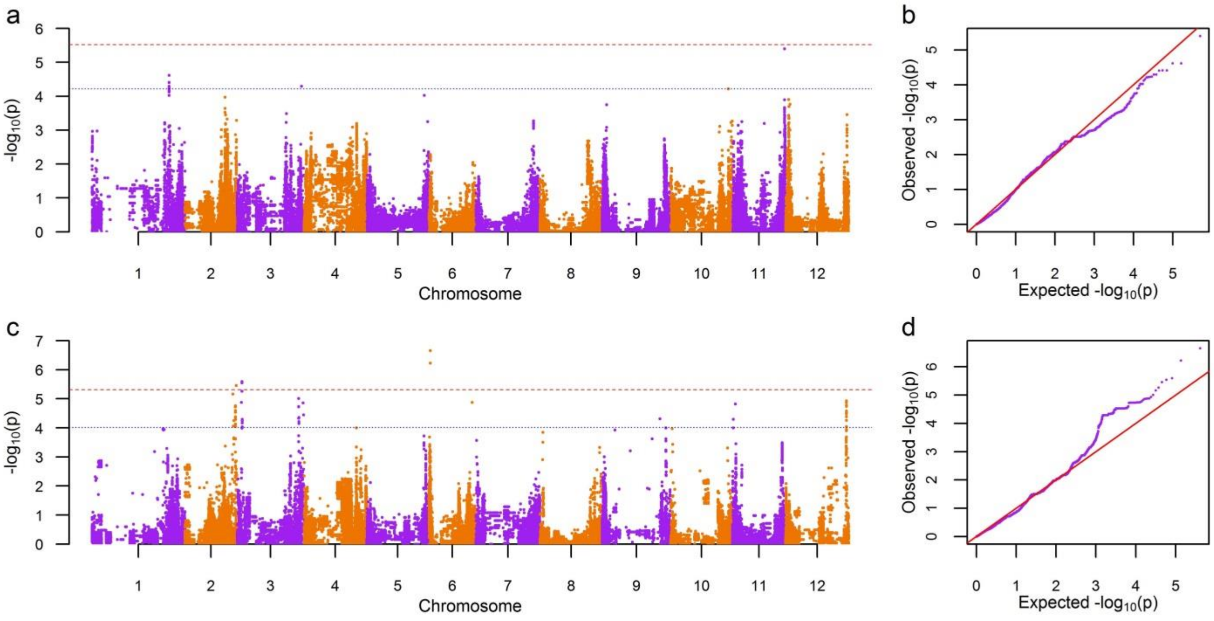

Supplementary Figure 20. Genome-wide association analysis of GABA from EMMAX. (**a**) Manhattan plot of panel B. (**b**) Quantile-quantile plot of panel B. (**c**) Manhattan plot of panel S. (**d**) Quantile-quantile plot of panel S.

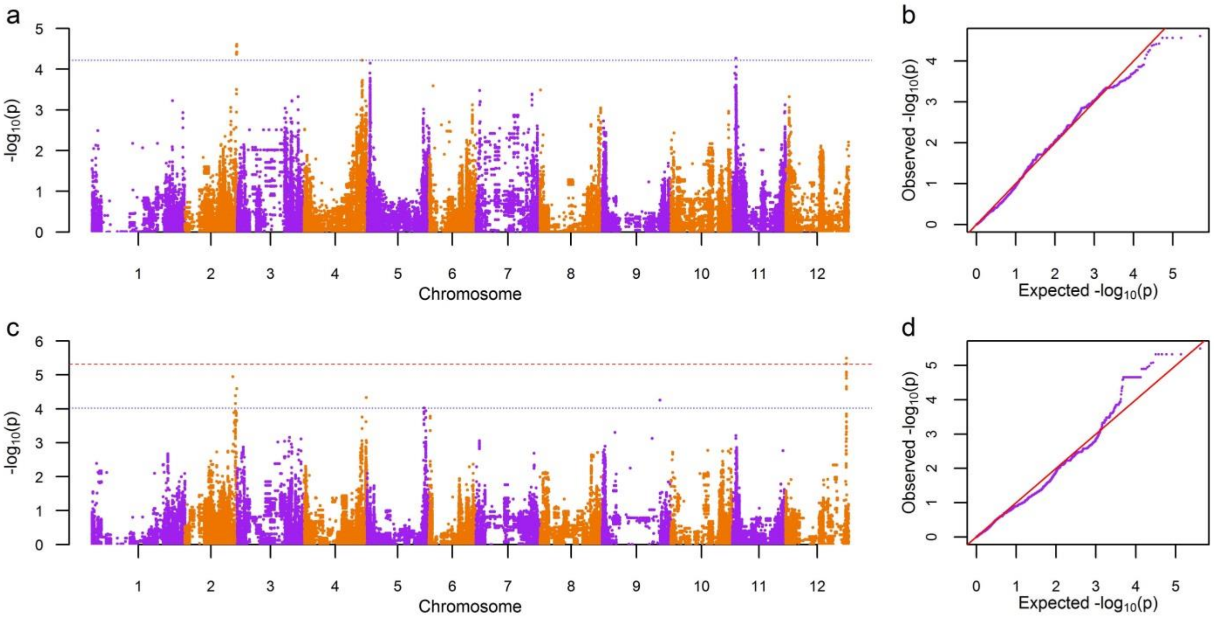

Supplementary Figure 21. Genome-wide association analysis of glutamine from EMMAX. (**a**) Manhattan plot of panel B. (**b**) Quantile-quantile plot of panel B. (**c**) Manhattan plot of panel S. (**d**) Quantile-quantile plot of panel S.

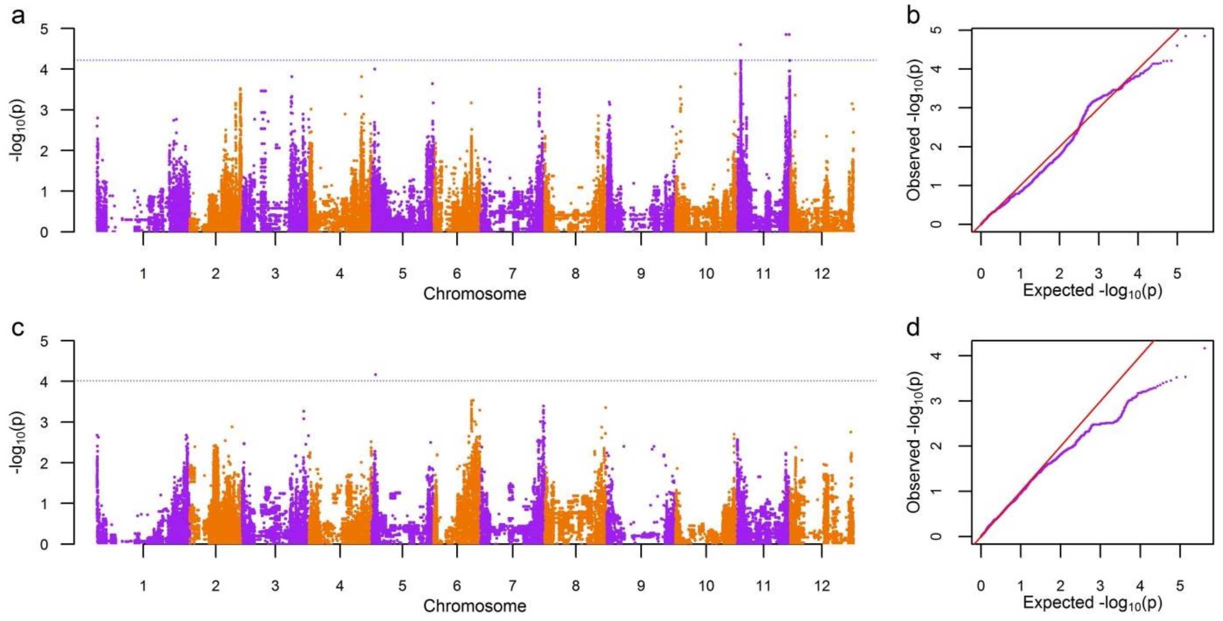

Supplementary Figure 22. Genome-wide association analysis of lysine from EMMAX. (**a**) Manhattan plot of panel B. (**b**) Quantile-quantile plot of panel B. (**c**) Manhattan plot of panel S. (**d**) Quantile-quantile plot of panel S.

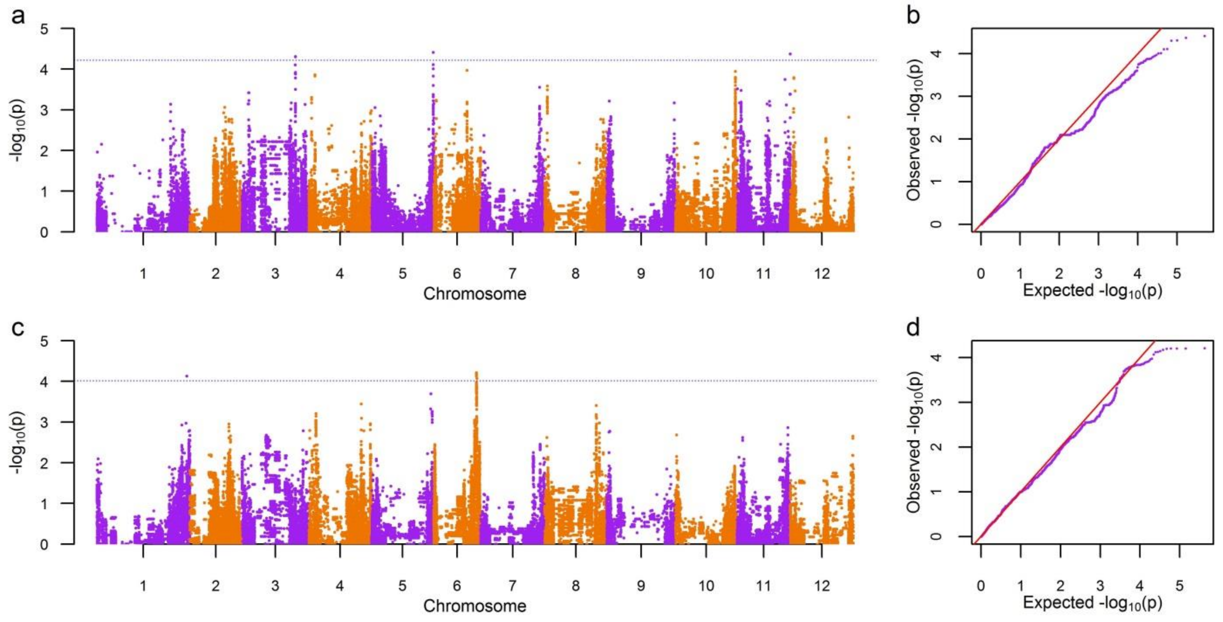

Supplementary Figure 23. Genome-wide association analysis of methionine from EMMAX. (**a**) Manhattan plot of panel B. (**b**) Quantile-quantile plot of panel B. (**c**) Manhattan plot of panel S. (**d**) Quantile-quantile plot of panel S.

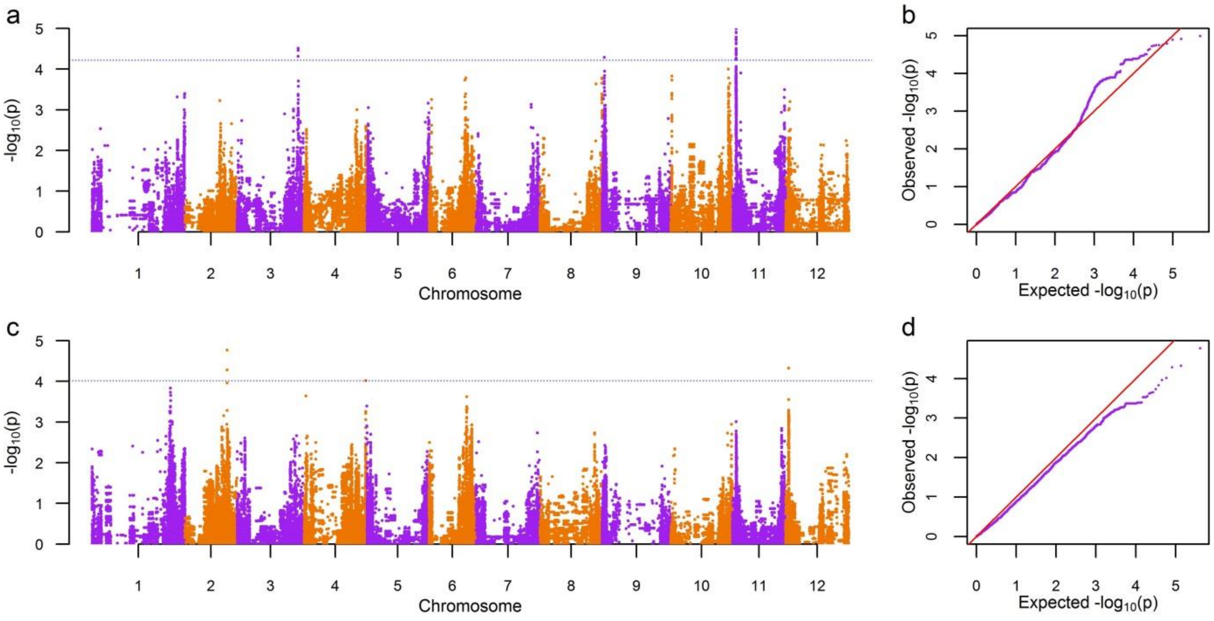

Supplementary Figure 24. Genome-wide association analysis of phenylalanine from EMMAX. (**a**) Manhattan plot of panel B. (**b**) Quantile-quantile plot of panel B. (**c**) Manhattan plot of panel S. (**d**) Quantile-quantile plot of panel S.

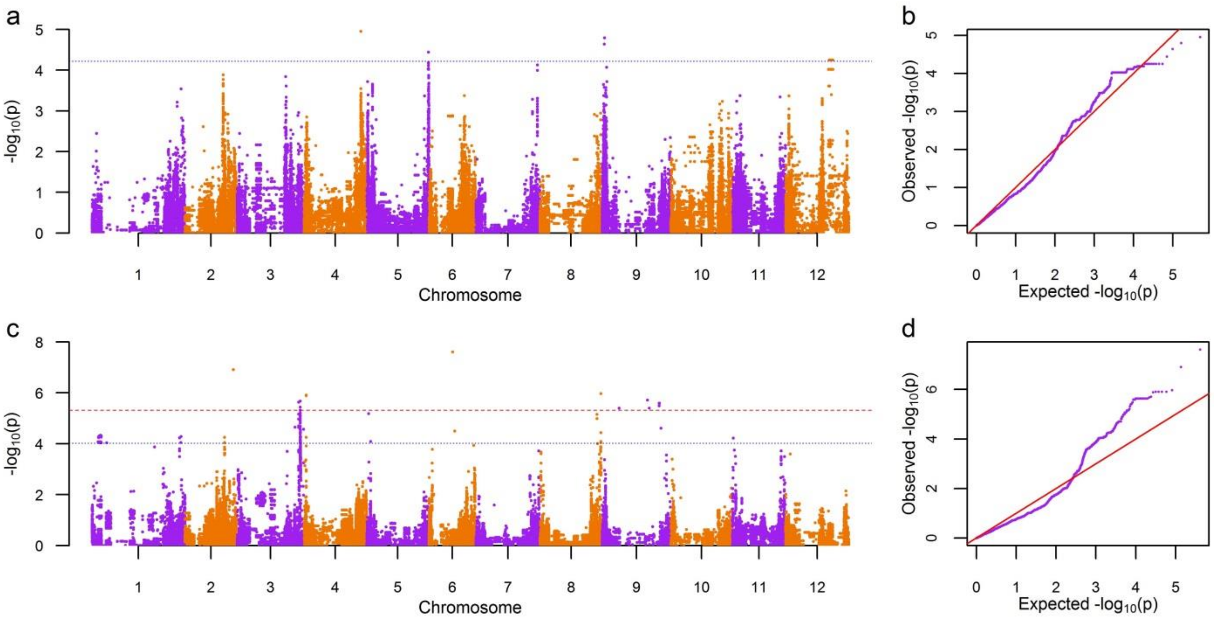

Supplementary Figure 25. Genome-wide association analysis of proline from EMMAX. (**a**) Manhattan plot of panel B. (**b**) Quantile-quantile plot of panel B. (**c**) Manhattan plot of panel S. (**d**) Quantile-quantile plot of panel S.

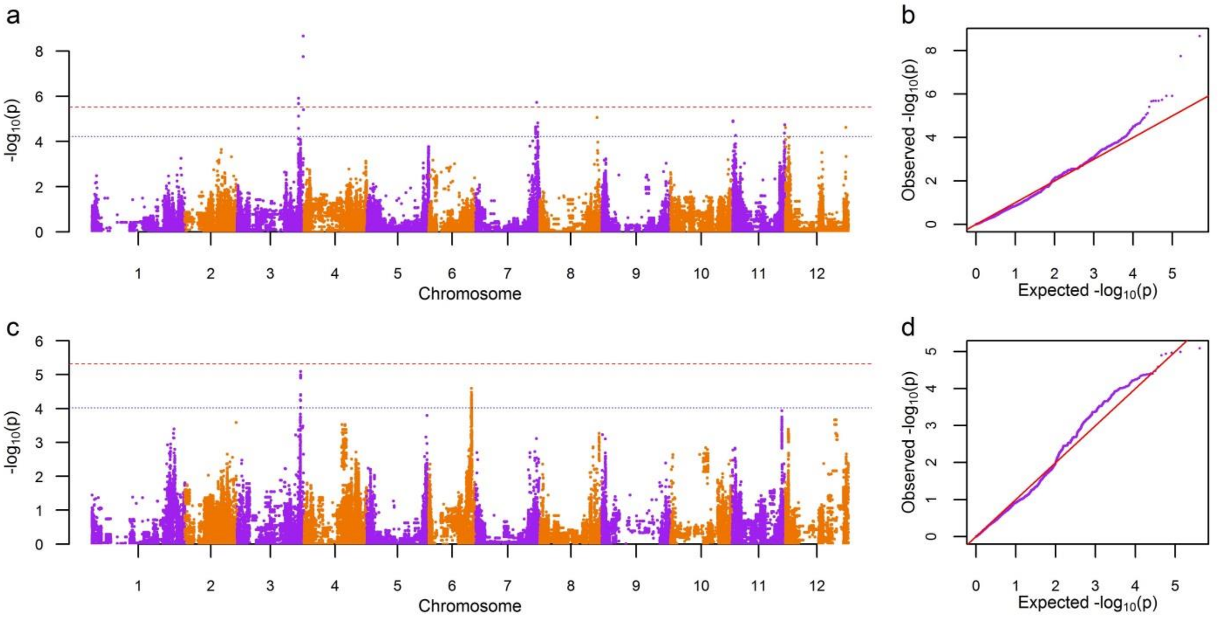

Supplementary Figure 26. Genome-wide association analysis of serine from EMMAX. (**a**) Manhattan plot of panel B. (**b**) Quantile-quantile plot of panel B. (**c**) Manhattan plot of panel S. (**d**) Quantile-quantile plot of panel S.

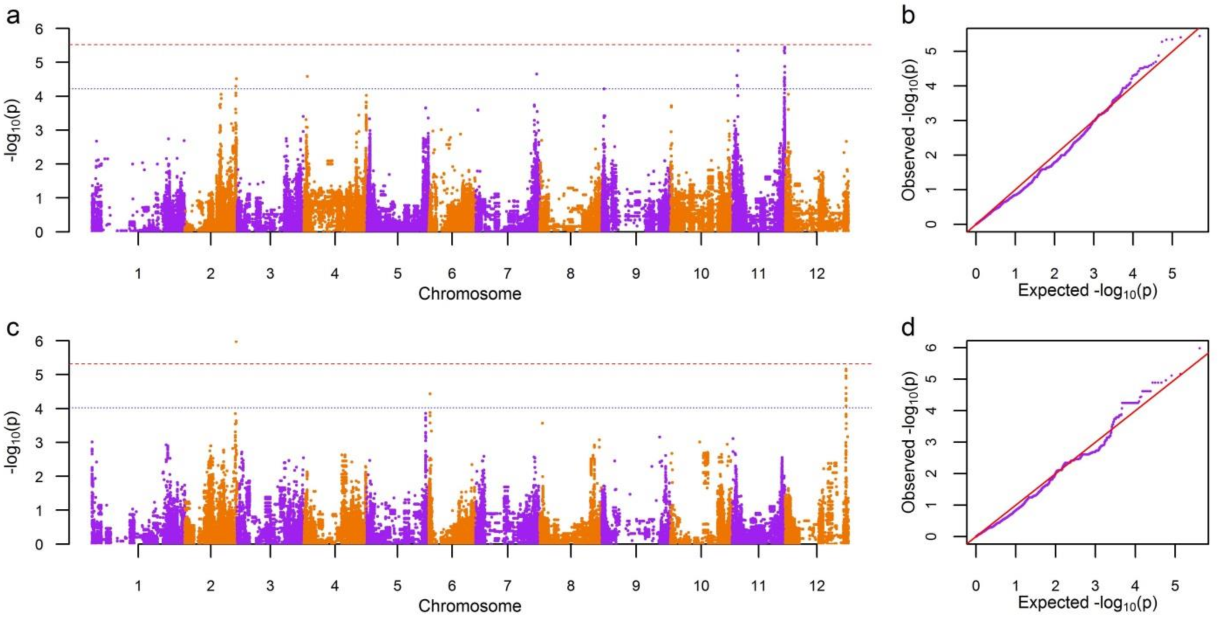

Supplementary Figure 27. Genome-wide association analysis of threonine from EMMAX. (**a**) Manhattan plot of panel B. (**b**) Quantile-quantile plot of panel B. (**c**) Manhattan plot of panel S. (**d**) Quantile-quantile plot of panel S.

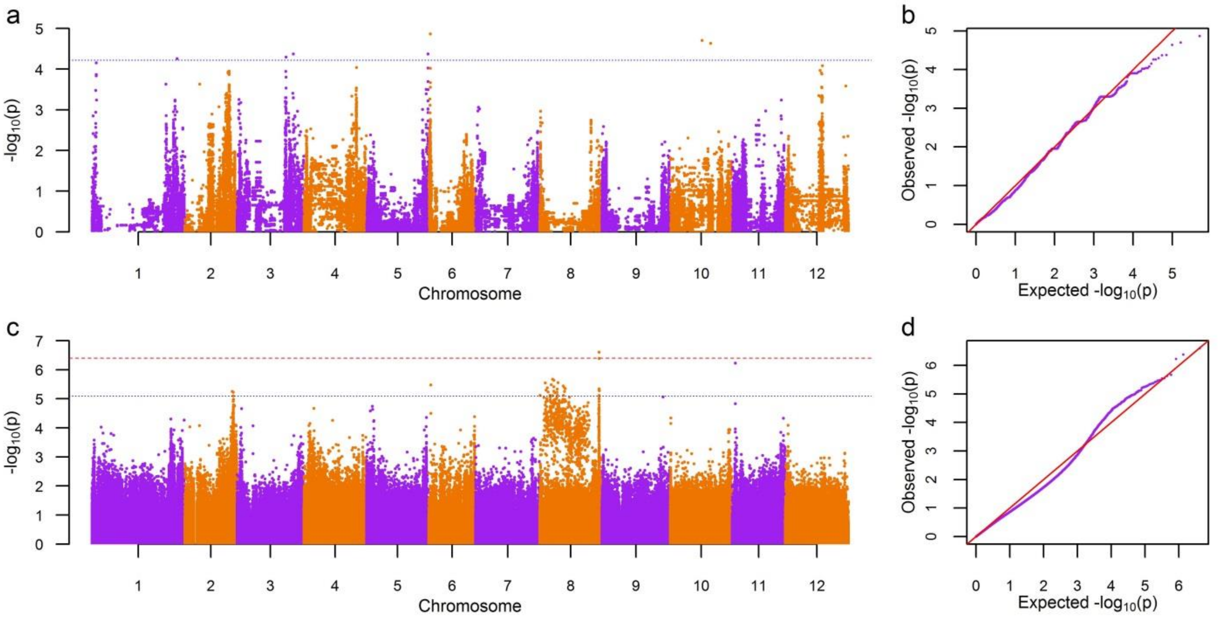

Supplementary Figure 28. Genome-wide association analysis of beta-ionone from EMMAX. (**a**) Manhattan plot of panel B. (**b**) Quantile-quantile plot of panel B. (**c**) Manhattan plot of panel T. (**d**) Quantile-quantile plot of panel T.

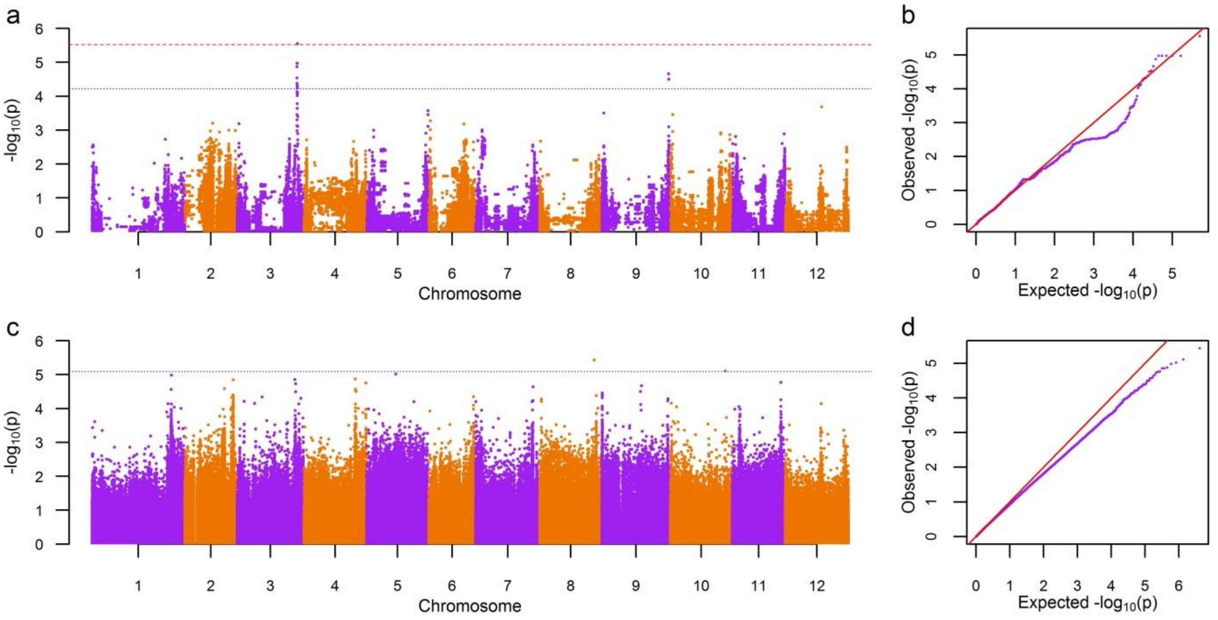

Supplementary Figure 29. Genome-wide association analysis of (E)-2-heptenal from EMMAX. (**a**) Manhattan plot of panel B. (**b**) Quantile-quantile plot of panel B. (**c**) Manhattan plot of panel T. (**d**) Quantile-quantile plot of panel T.

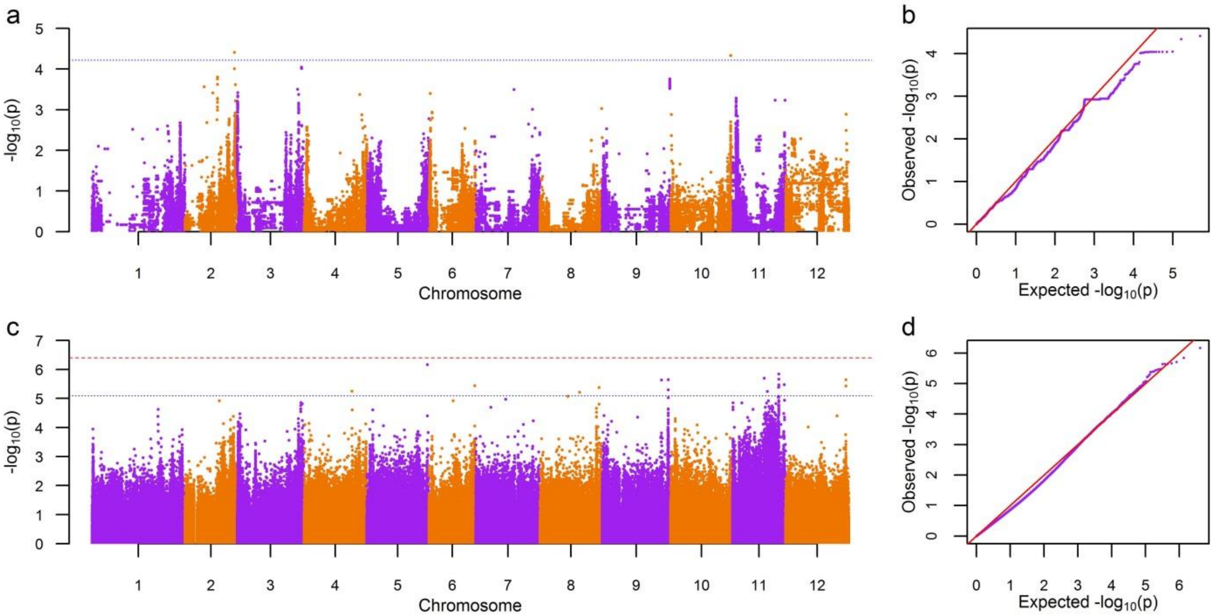

Supplementary Figure 30. Genome-wide association analysis of (E)-2-hexenal from EMMAX. (**a**) Manhattan plot of panel B. (**b**) Quantile-quantile plot of panel B. (**c**) Manhattan plot of panel T. (**d**) Quantile-quantile plot of panel T.

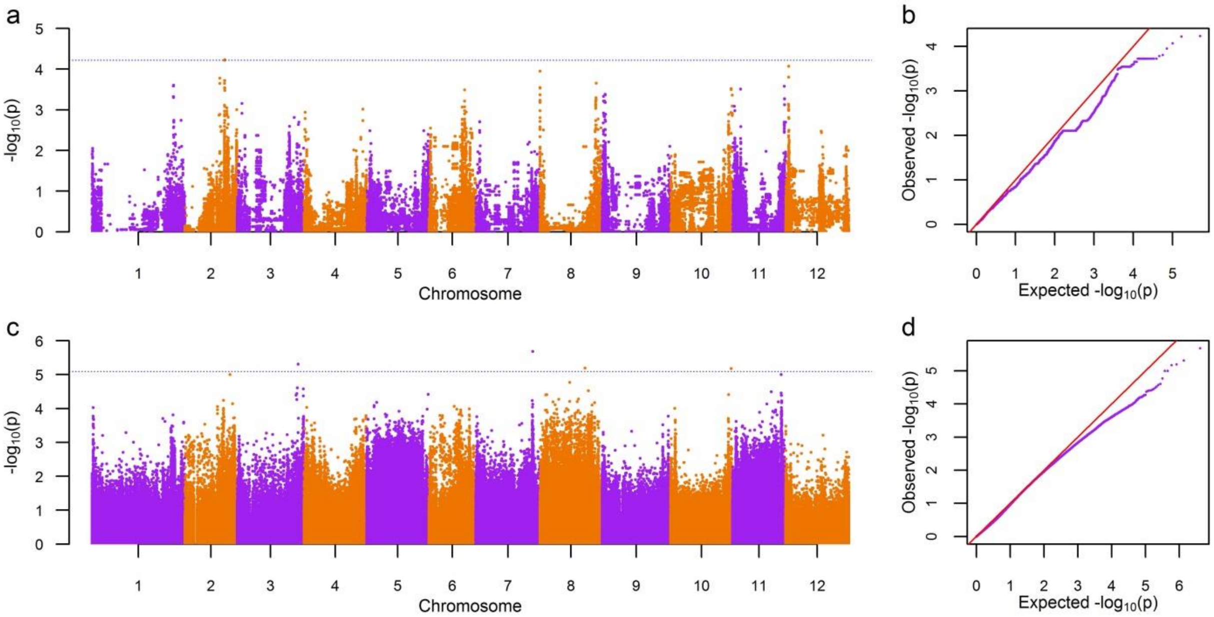

Supplementary Figure 31. Genome-wide association analysis of (E,E)-2,4-decadienal from EMMAX. (**a**) Manhattan plot of panel B. (**b**) Quantile-quantile plot of panel B. (**c**) Manhattan plot of panel T. (**d**) Quantile-quantile plot of panel T.

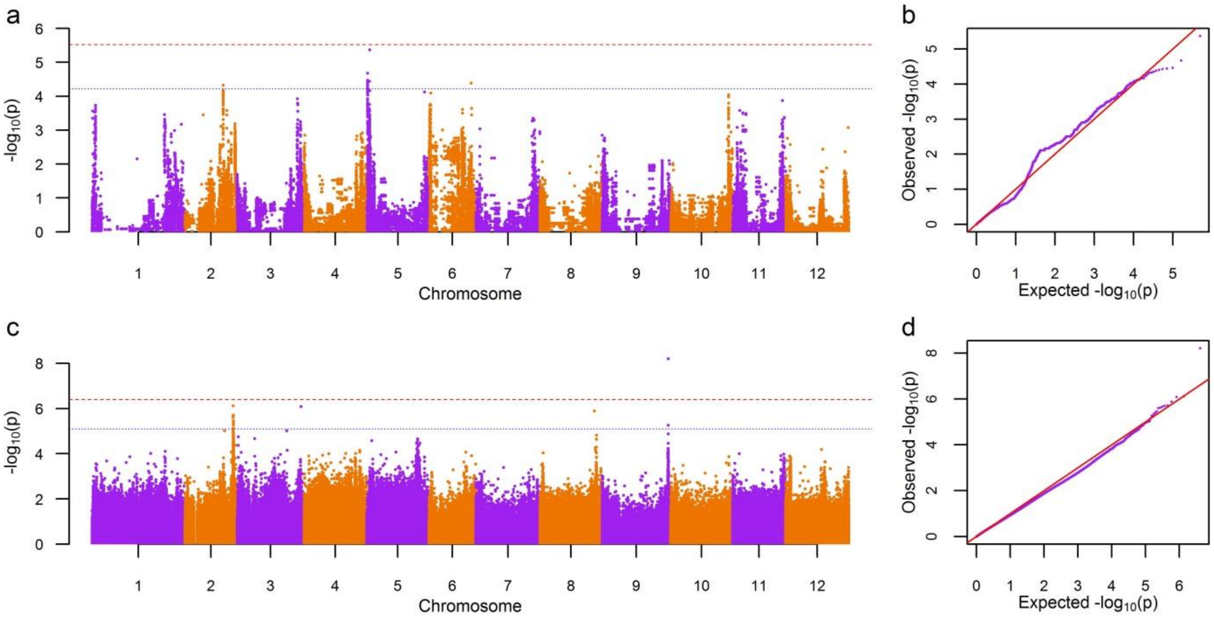

Supplementary Figure 32. Genome-wide association analysis of (E)-2-pentenal from EMMAX. (**a**) Manhattan plot of panel B. (**b**) Quantile-quantile plot of panel B. (**c**) Manhattan plot of panel T. (**d**) Quantile-quantile plot of panel T.

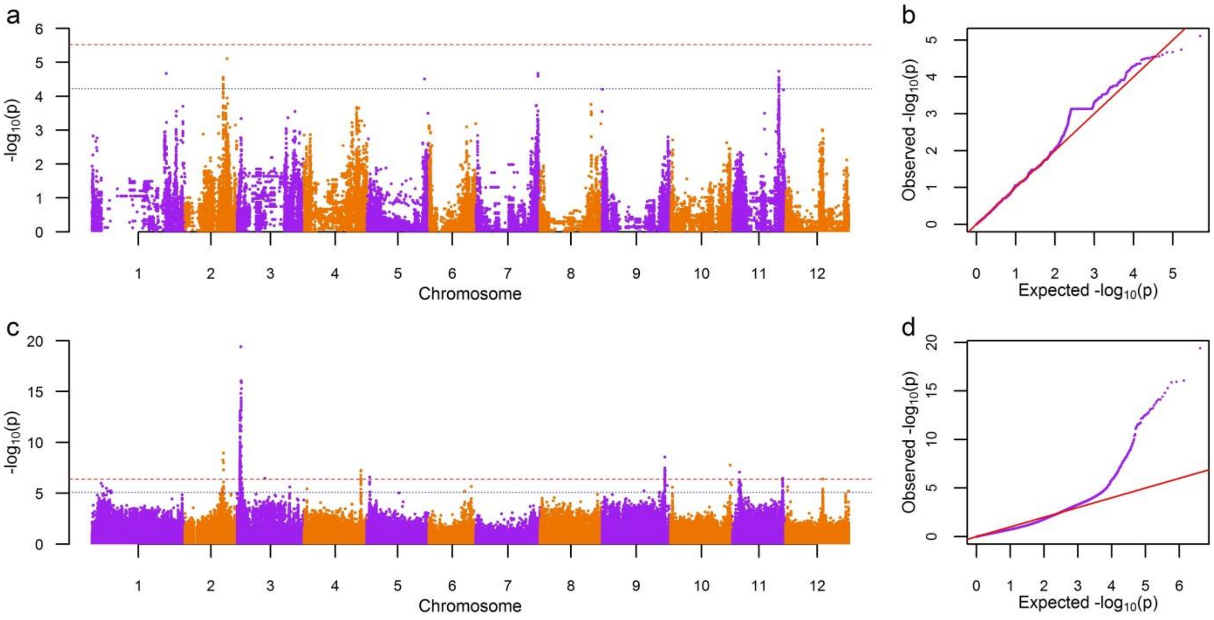

Supplementary Figure 33. Genome-wide association analysis of geranyl acetone from EMMAX. (**a**) Manhattan plot of panel B. (**b**) Quantile-quantile plot of panel B. (**c**) Manhattan plot of panel T. (**d**) Quantile-quantile plot of panel T.

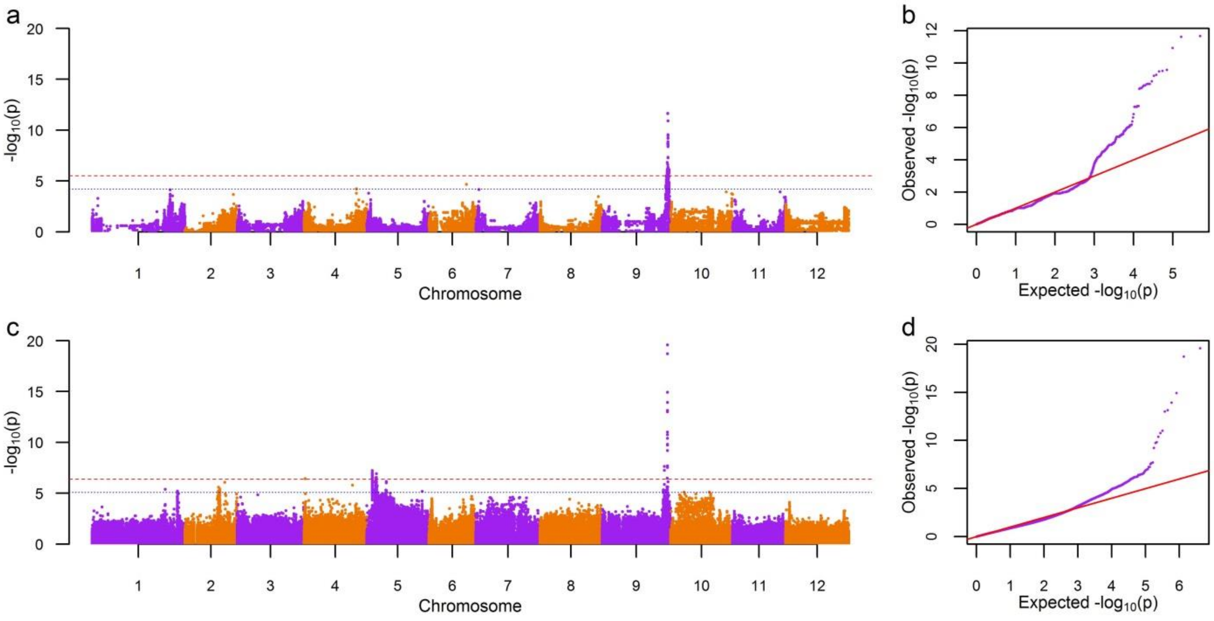

Supplementary Figure 34. Genome-wide association analysis of guaiacol from EMMAX. (**a**) Manhattan plot of panel B. (**b**) Quantile-quantile plot of panel B. (**c**) Manhattan plot of panel T. (**d**) Quantile-quantile plot of panel T.

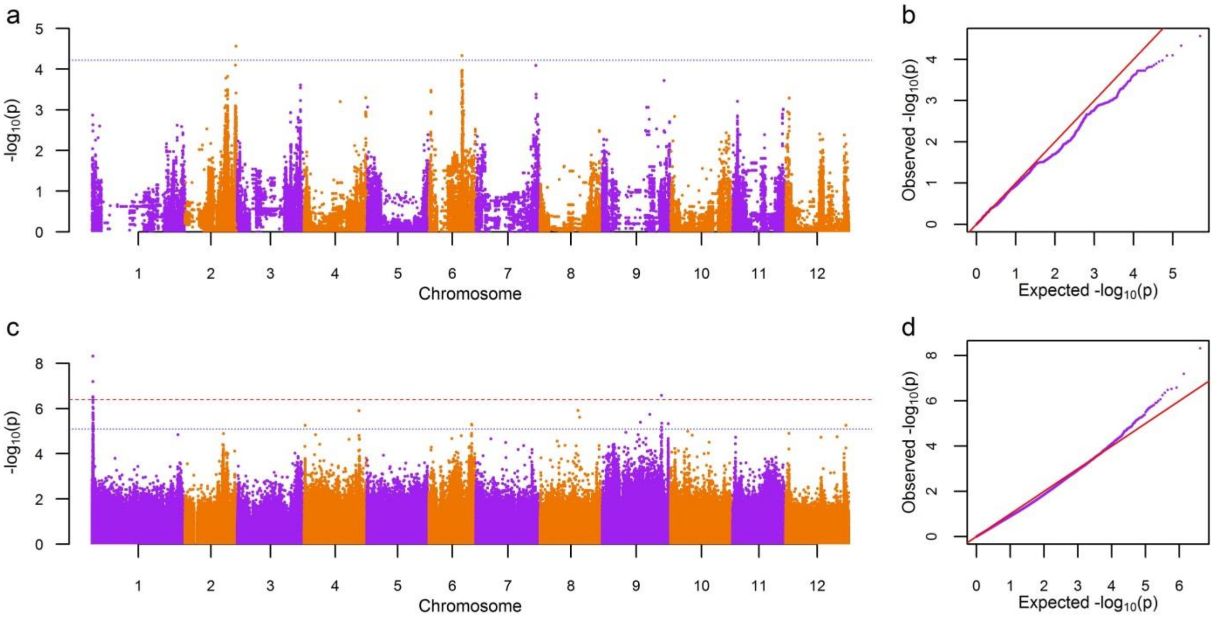

Supplementary Figure 35. Genome-wide association analysis of hexanal from EMMAX. (**a**) Manhattan plot of panel B. (**b**) Quantile-quantile plot of panel B. (**c**) Manhattan plot of panel T. (**d**) Quantile-quantile plot of panel T.

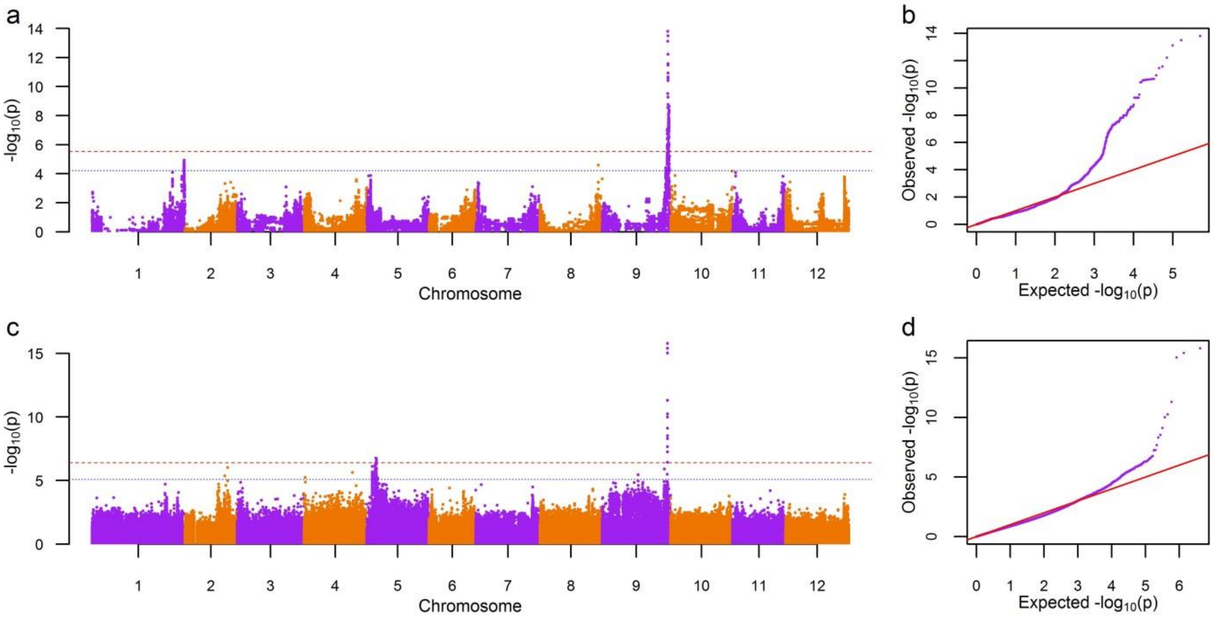

Supplementary Figure 36. Genome-wide association analysis of methyl salicylate from EMMAX. (**a**) Manhattan plot of panel B. (**b**) Quantile-quantile plot of panel B. (**c**) Manhattan plot of panel T. (**d**) Quantile-quantile plot of panel T.

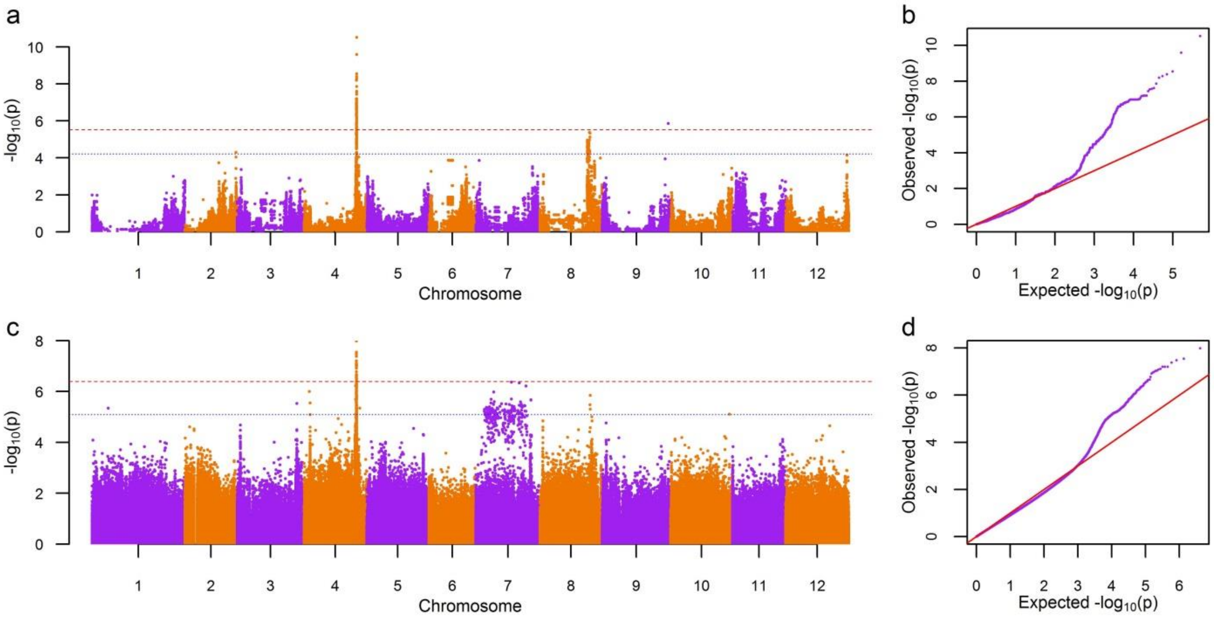

Supplementary Figure 37. Genome-wide association analysis of phenylacetaldehyde from EMMAX. (**a**) Manhattan plot of panel B. (**b**) Quantile-quantile plot of panel T. (**c**) Manhattan plot of panel S. (**d**) Quantile-quantile plot of panel T.

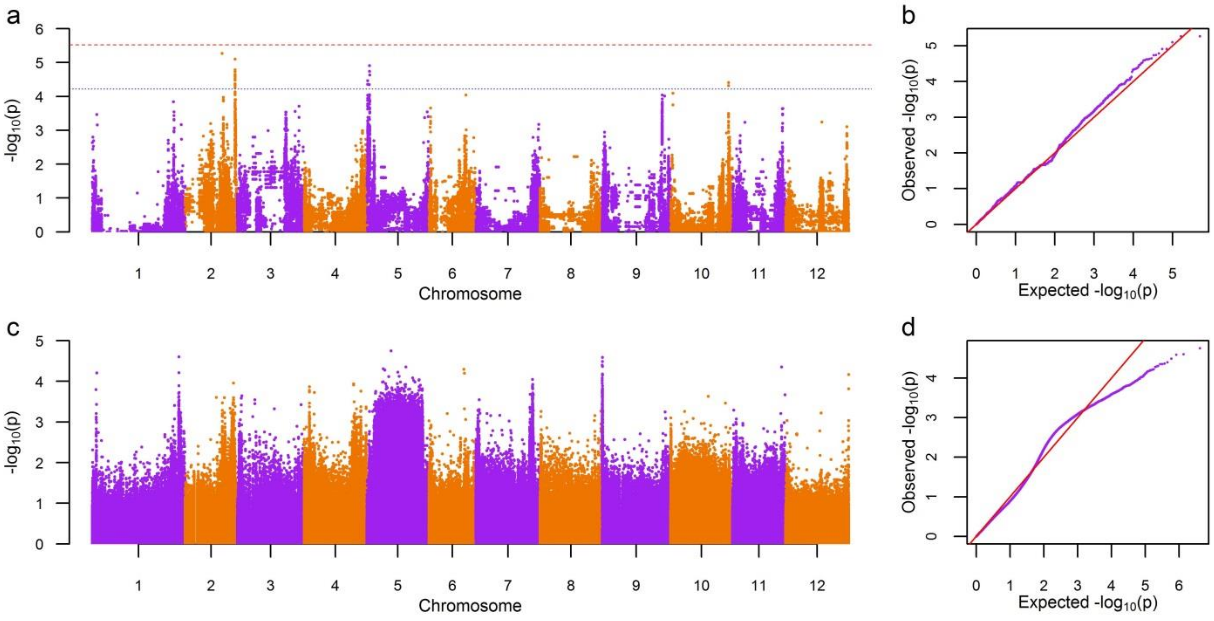

Supplementary Figure 38. Genome-wide association analysis of 1-octen-3-one from EMMAX. (**a**) Manhattan plot of panel B. (**b**) Quantile-quantile plot of panel B. (**c**) Manhattan plot of panel T. (**d**) Quantile-quantile plot of panel T.

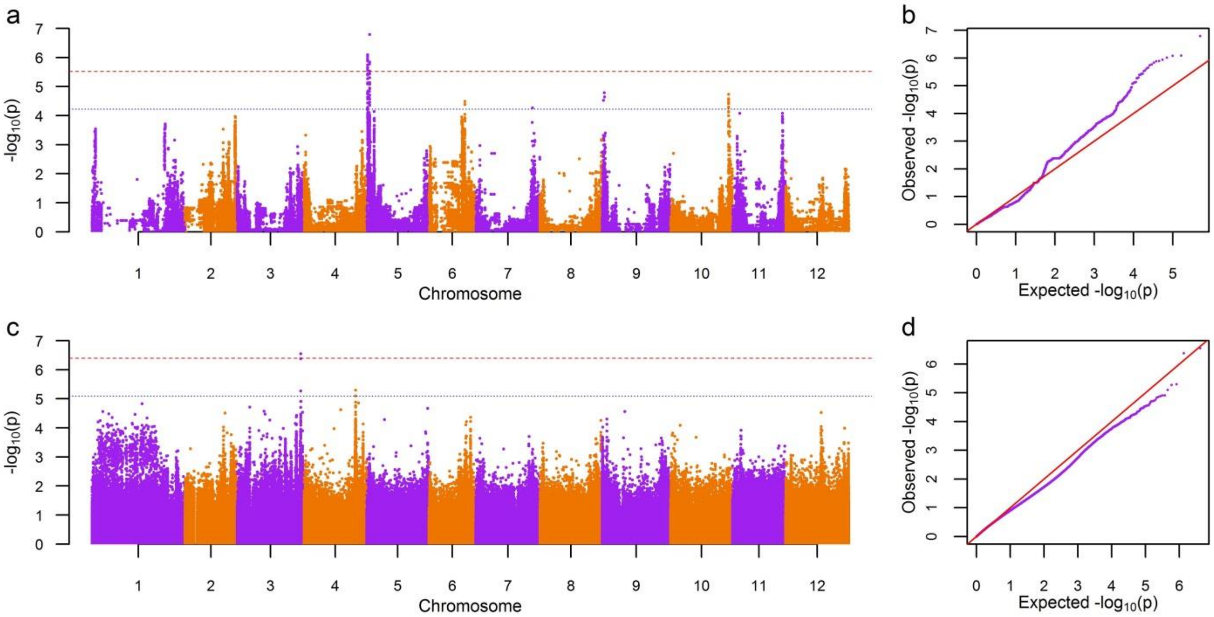

Supplementary Figure 39. Genome-wide association analysis of 1-penten-3-one from EMMAX. (**a**) Manhattan plot of panel B. (**b**) Quantile-quantile plot of panel B. (**c**) Manhattan plot of panel T. (**d**) Quantile-quantile plot of panel T.

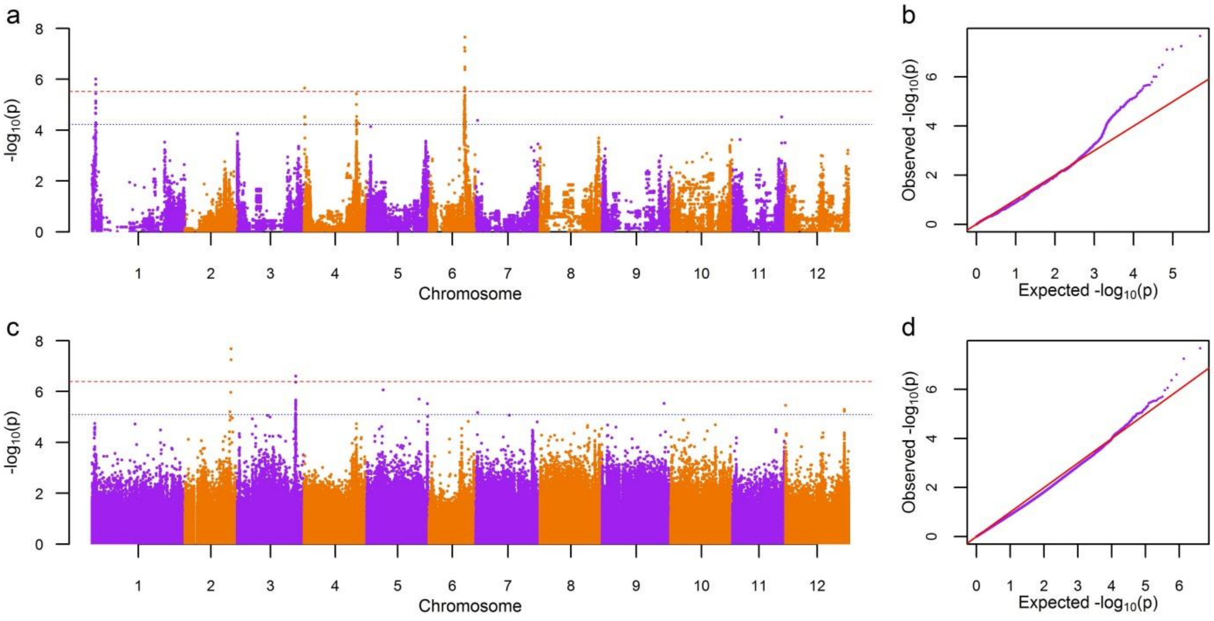

Supplementary Figure 40. Genome-wide association analysis of 2-methyl-1-butanol from EMMAX. (**a**) Manhattan plot of panel B. (**b**) Quantile-quantile plot of panel B. (**c**) Manhattan plot of panel T. (**d**) Quantile-quantile plot of panel T.

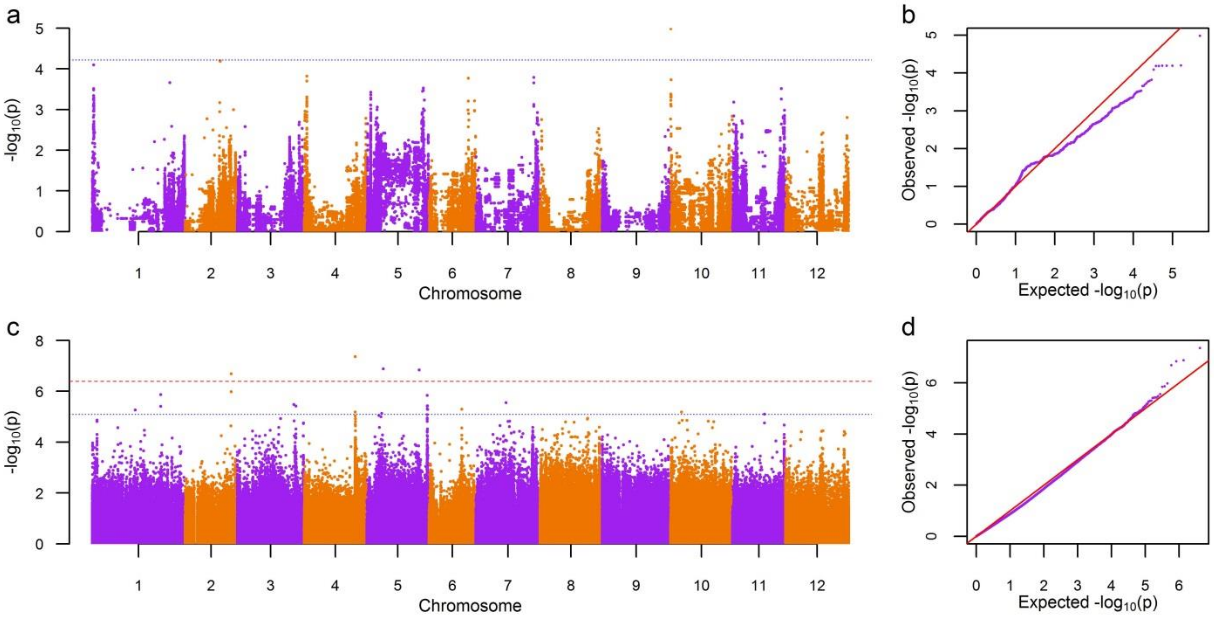

Supplementary Figure 41. Genome-wide association analysis of 3-methyl-1-butanol from EMMAX. (**a**) Manhattan plot of panel B. (**b**) Quantile-quantile plot of panel B. (**c**) Manhattan plot of panel T. (**d**) Quantile-quantile plot of panel T.

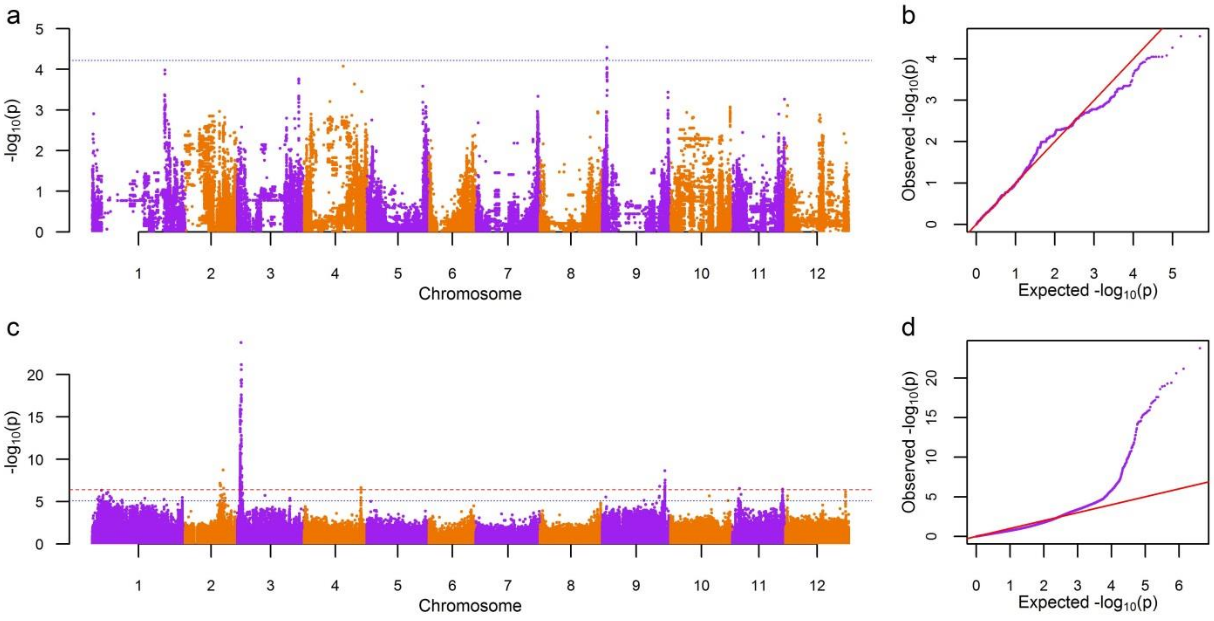

Supplementary Figure 42. Genome-wide association analysis of 6-methyl-5-hepten-2-one from EMMAX. (**a**) Manhattan plot of panel B. (**b**) Quantile-quantile plot of panel B. (**c**) Manhattan plot of panel T. (**d**) Quantile-quantile plot of panel T.

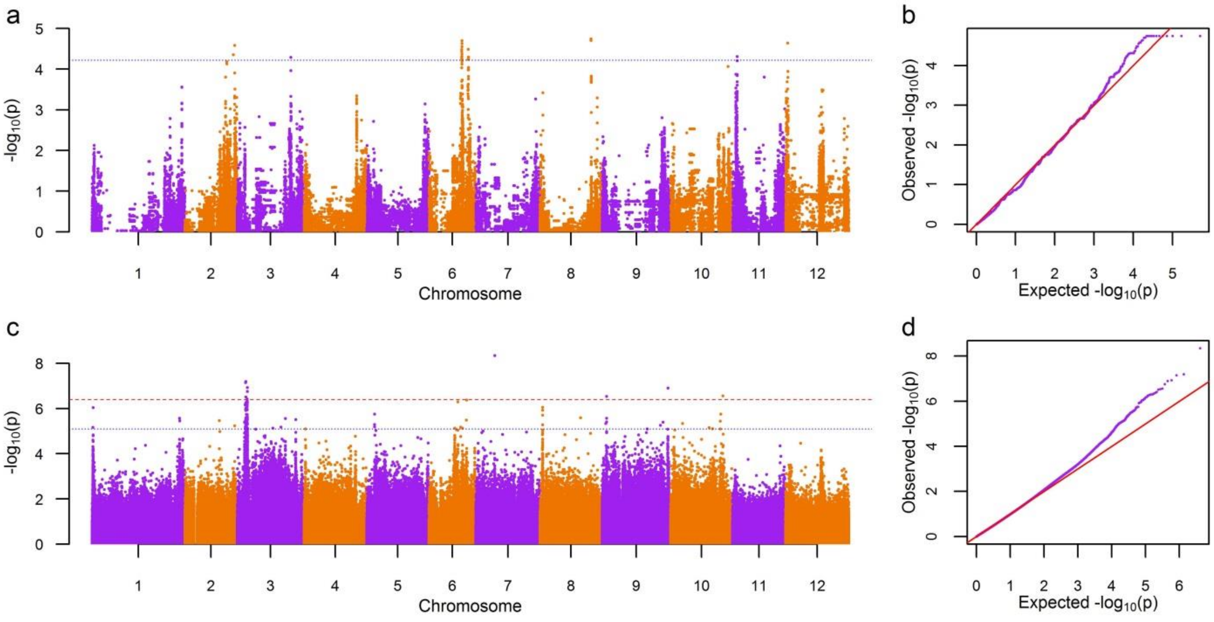

Supplementary Figure 43. Genome-wide association analysis of (Z)-3-hexen-1-ol from EMMAX. (**a**) Manhattan plot of panel B. (**b**) Quantile-quantile plot of panel B. (**c**) Manhattan plot of panel T. (**d**) Quantile-quantile plot of panel T.

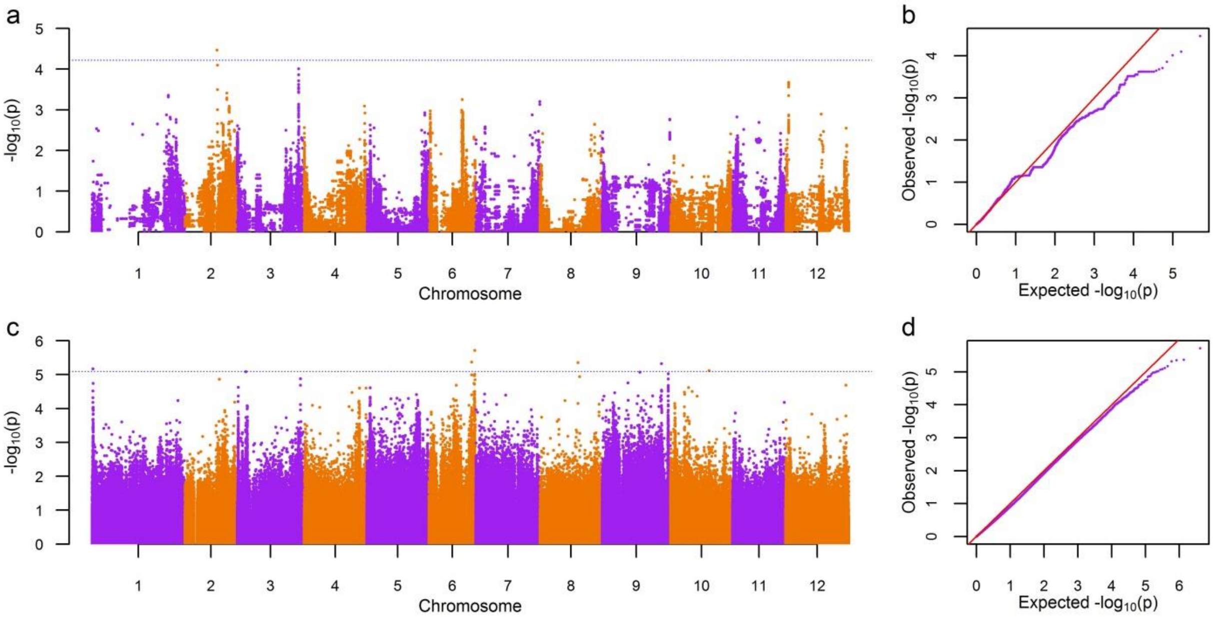

Supplementary Figure 44. Genome-wide association analysis of (Z)-3-hexenal from EMMAX. (**a**) Manhattan plot of panel B. (**b**) Quantile-quantile plot of panel B. (**c**) Manhattan plot of panel T. (**d**) Quantile-quantile plot of panel T.

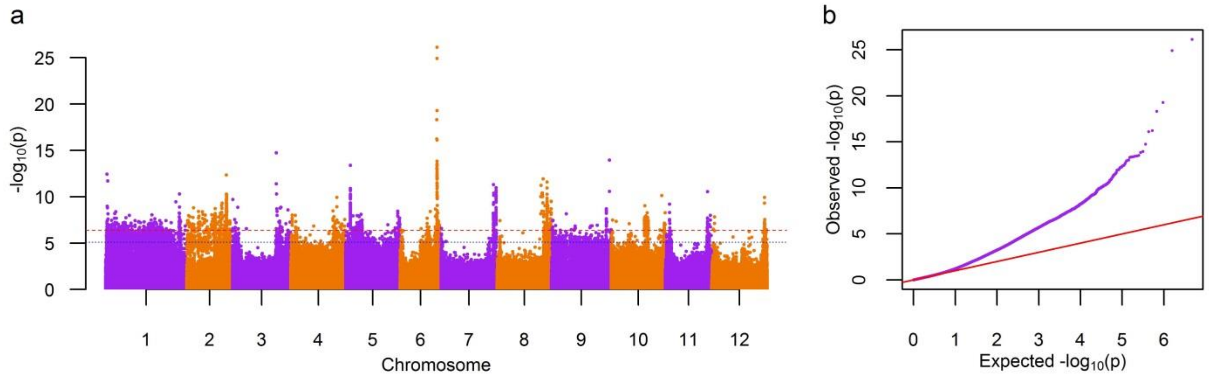

Supplementary Figure 45. Meta-analysis of genome-wide association of citrate. (**a**) Manhattan plot of meta-analysis. (**b**) Quantile-quantile plot of meta-analysis.

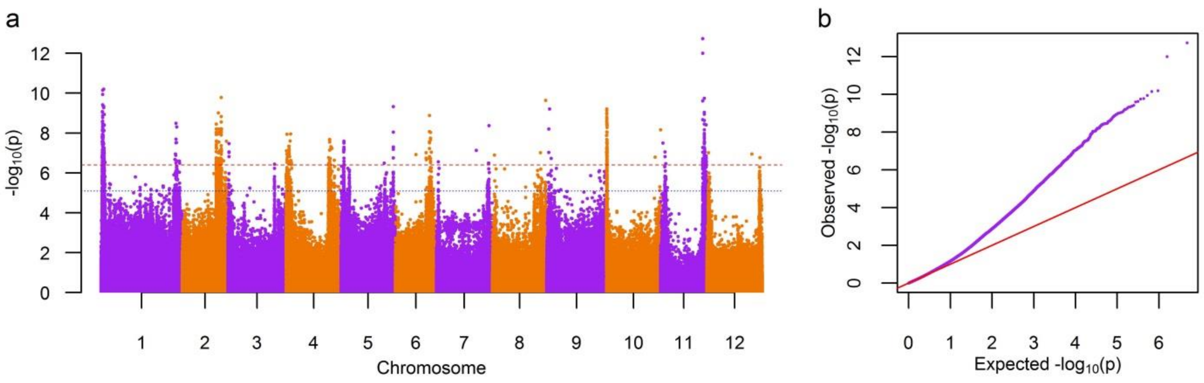

Supplementary Figure 46. Meta-analysis of genome-wide association of fructose. (**a**) Manhattan plot of meta-analysis. (**b**) Quantile-quantile plot of meta-analysis.

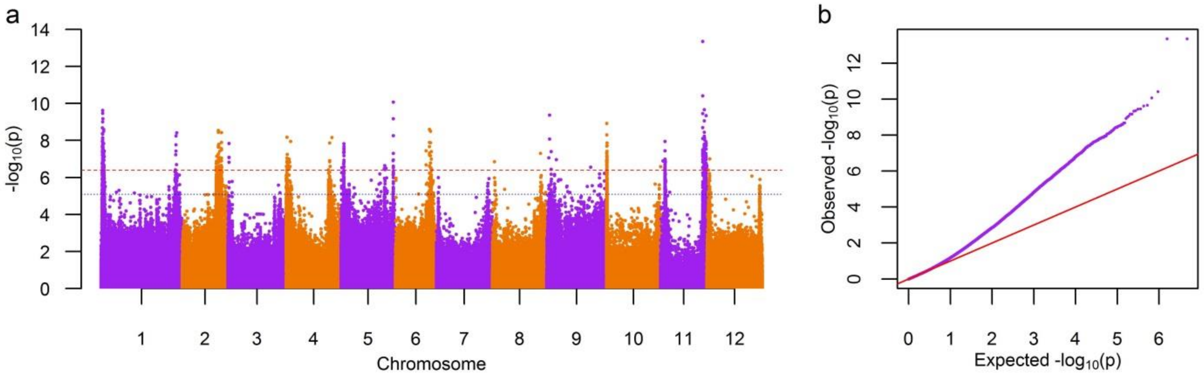

Supplementary Figure 47. Meta-analysis of genome-wide association of glucose. (**a**) Manhattan plot of meta-analysis. (**b**) Quantile-quantile plot of meta-analysis.

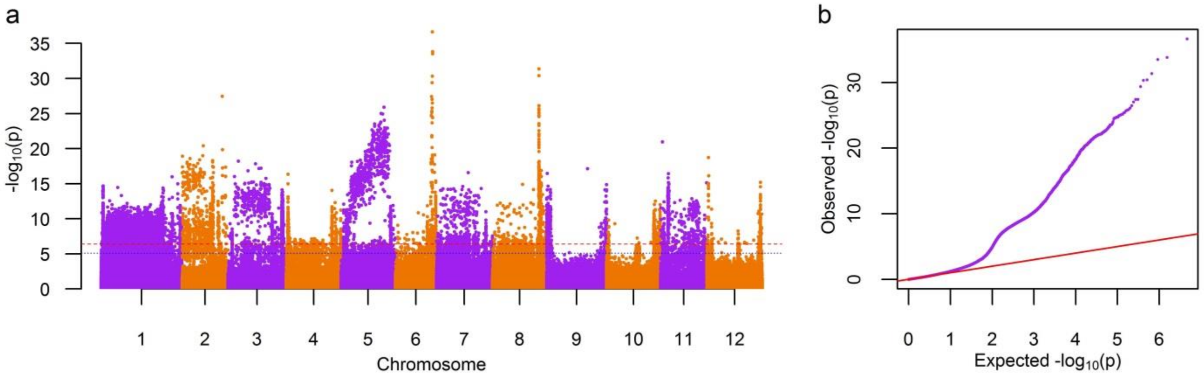

Supplementary Figure 48. Meta-analysis of genome-wide association of malate. (**a**) Manhattan plot of meta-analysis. (**b**) Quantile-quantile plot of meta-analysis.

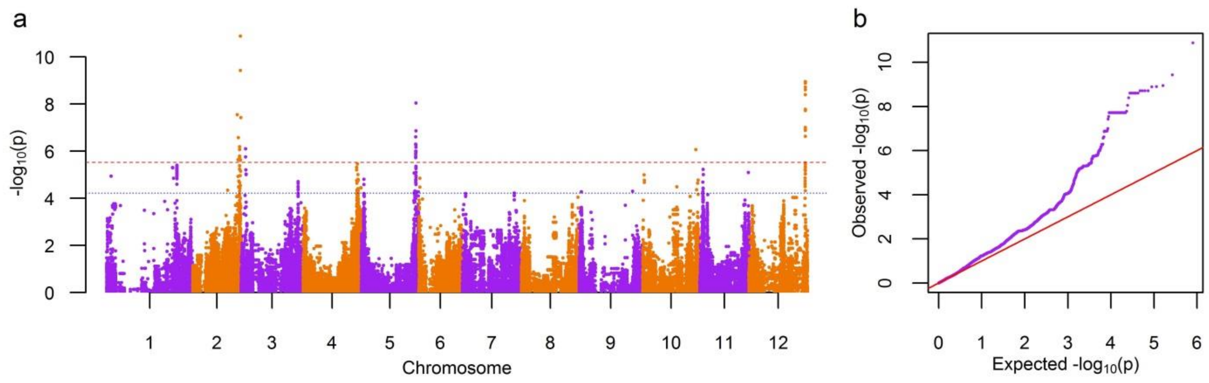

Supplementary Figure 49. Meta-analysis of genome-wide association of asparagine. (**a**) Manhattan plot of meta-analysis. (**b**) Quantile-quantile plot of meta-analysis.

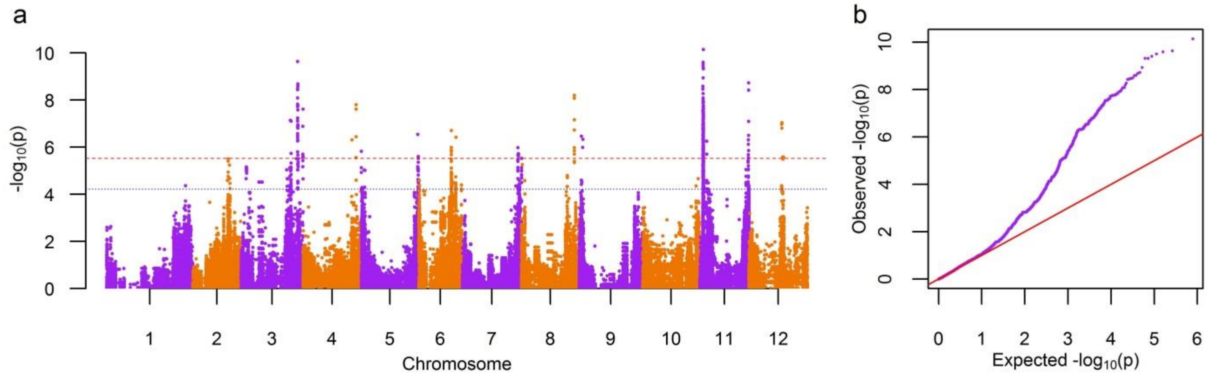

Supplementary Figure 50. Meta-analysis of genome-wide association of aspartate. (**a**) Manhattan plot of meta-analysis. (**b**) Quantile-quantile plot of meta-analysis.

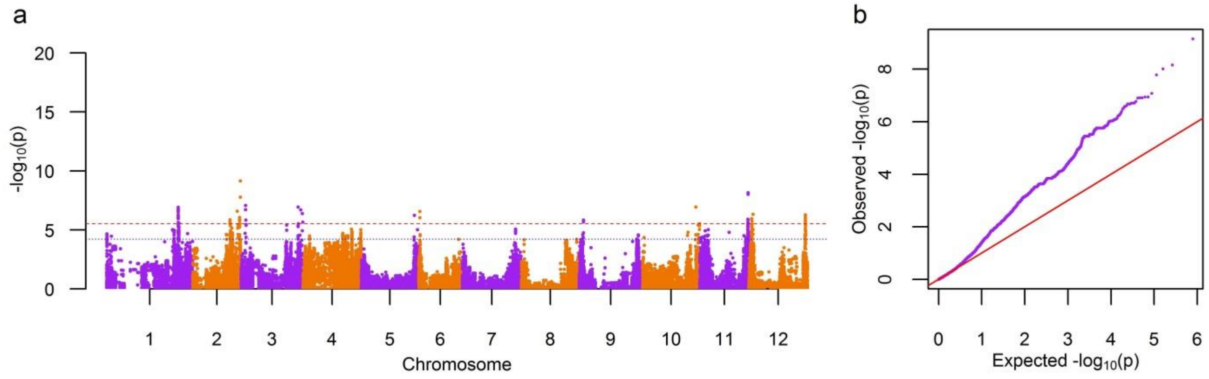

Supplementary Figure 51. Meta-analysis of genome-wide association of GABA. (**a**) Manhattan plot of meta-analysis. (**b**) Quantile-quantile plot of meta-analysis.

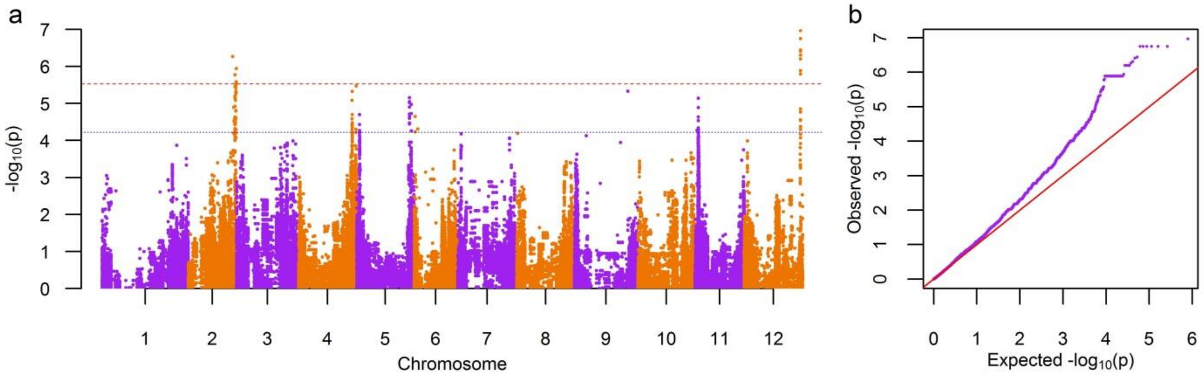

Supplementary Figure 52. Meta-analysis of genome-wide association of glutamine. (**a**) Manhattan plot of meta-analysis. (**b**) Quantile-quantile plot of meta-analysis.

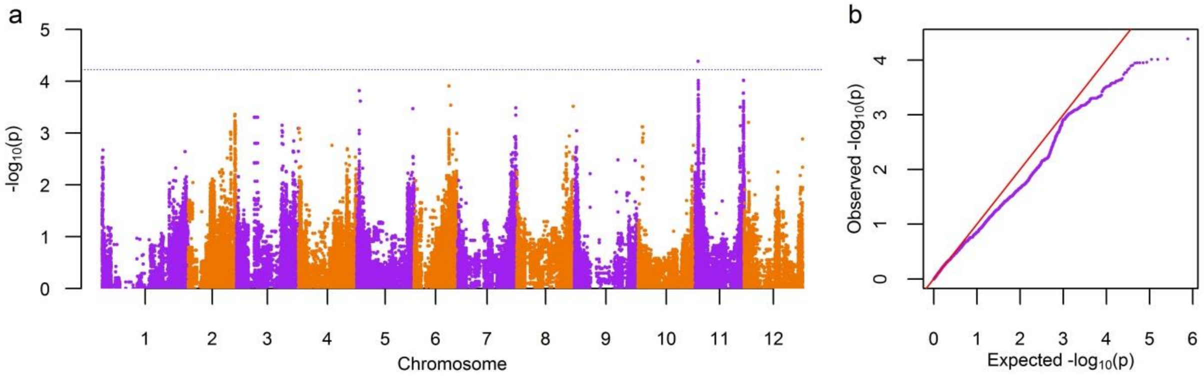

Supplementary Figure 53. Meta-analysis of genome-wide association of lysine. (**a**) Manhattan plot of meta-analysis. (**b**) Quantile-quantile plot of meta-analysis.

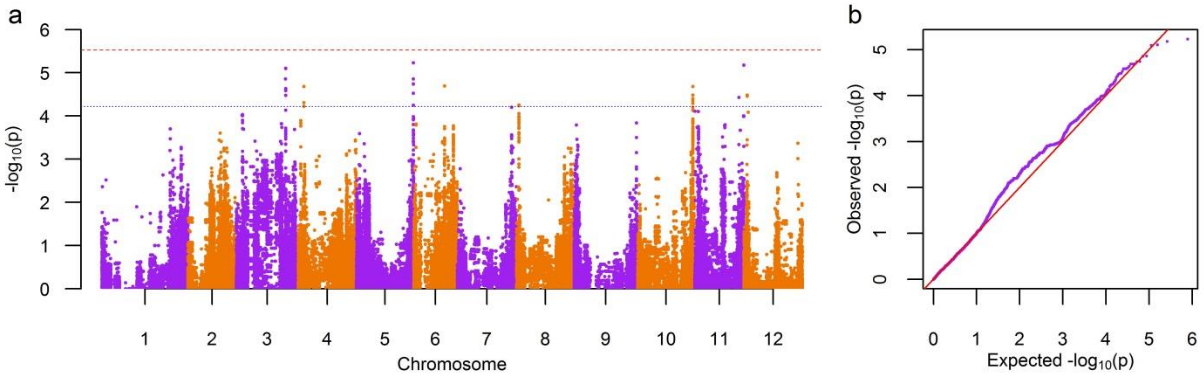

Supplementary Figure 54. Meta-analysis of genome-wide association of methionine. (**a**) Manhattan plot of meta-analysis. (**b**) Quantile-quantile plot of meta-analysis.

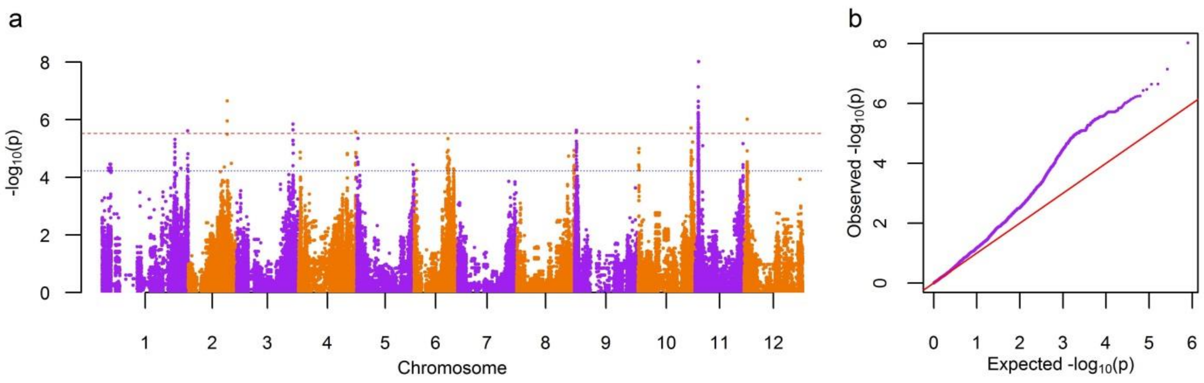

Supplementary Figure 55. Meta-analysis of genome-wide association of phenylalanine. (**a**) Manhattan plot of meta-analysis. (**b**) Quantile-quantile plot of meta-analysis.

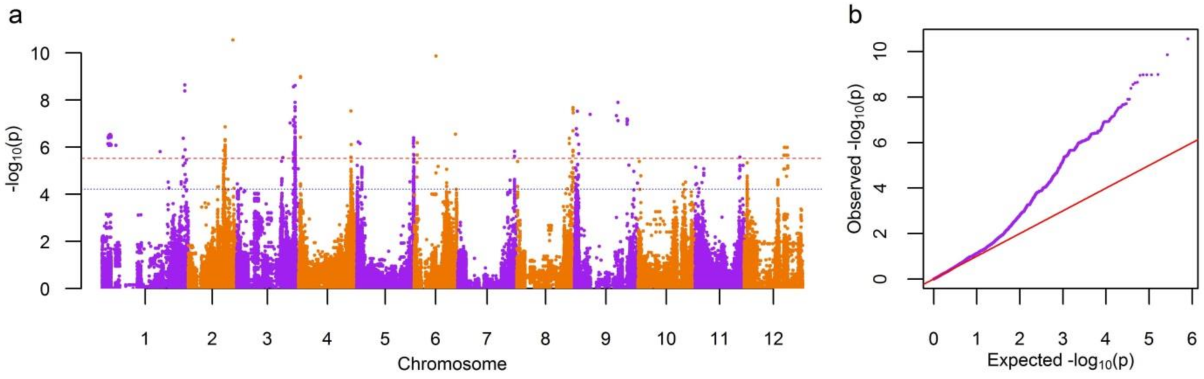

Supplementary Figure 56. Meta-analysis of genome-wide association of proline. (**a**) Manhattan plot of meta-analysis. (**b**) Quantile-quantile plot of meta-analysis.

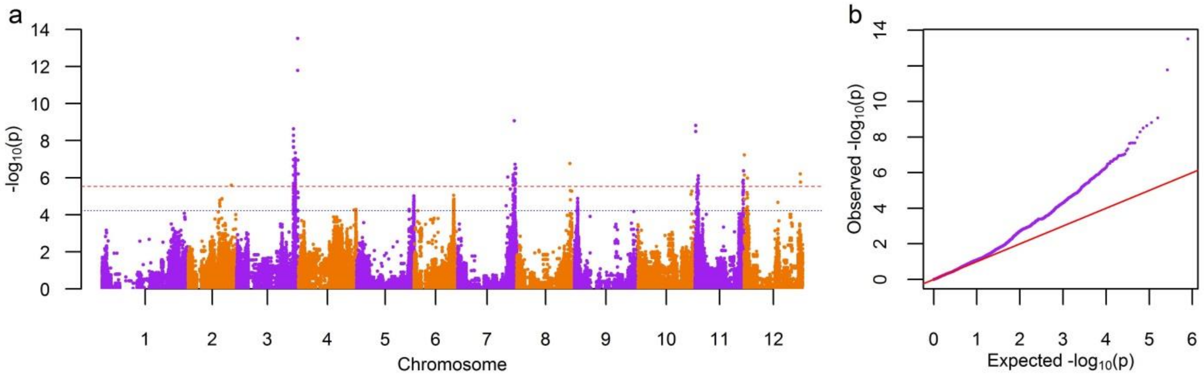

Supplementary Figure 57. Meta-analysis of genome-wide association of serine. (**a**) Manhattan plot of meta-analysis. (**b**) Quantile-quantile plot of meta-analysis.

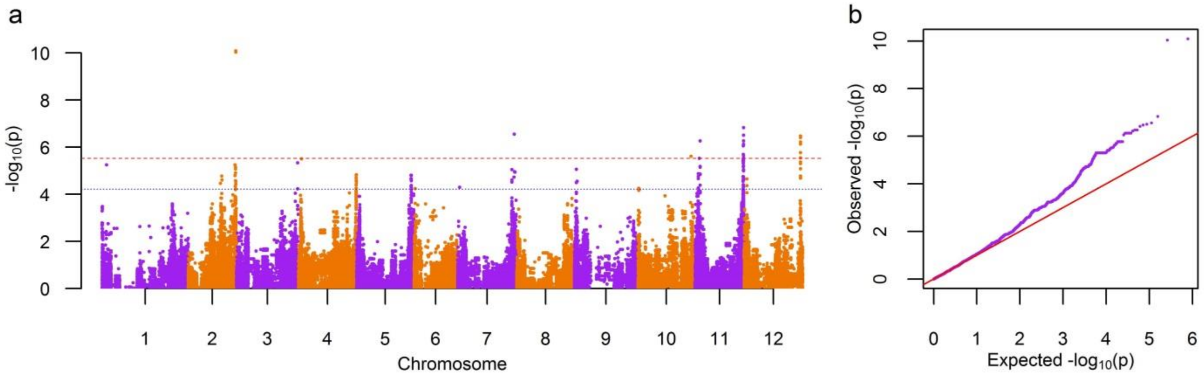

Supplementary Figure 58. Meta-analysis of genome-wide association of threonine. (**a**) Manhattan plot of meta-analysis. (**b**) Quantile-quantile plot of meta-analysis.

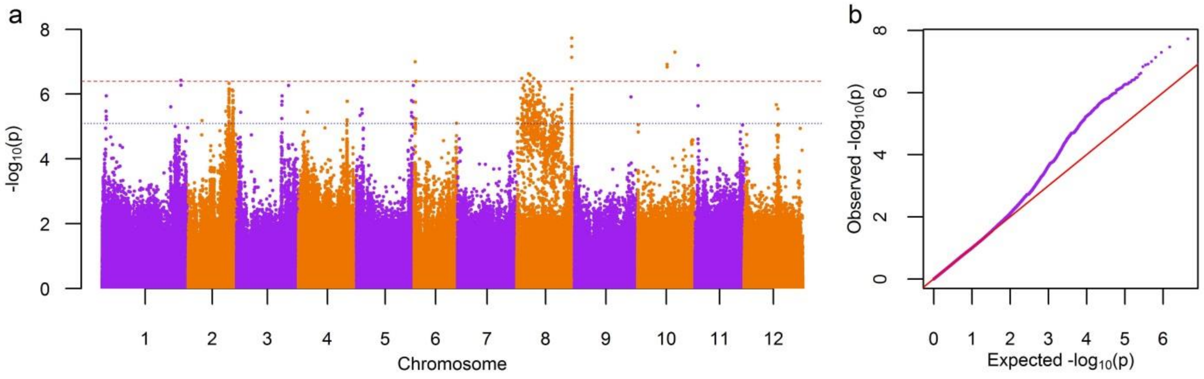

Supplementary Figure 59. Meta-analysis of genome-wide association of beta-ionone. (**a**) Manhattan plot of meta-analysis. (**b**) Quantile-quantile plot of meta-analysis.

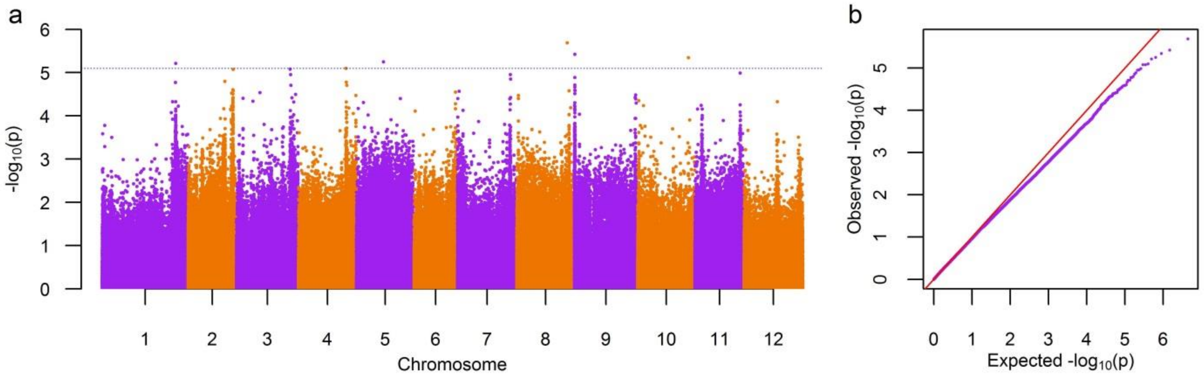

Supplementary Figure 60. Meta-analysis of genome-wide association of (E)-2-heptenal. (**a**) Manhattan plot of meta-analysis. (**b**) Quantile-quantile plot of meta-analysis.

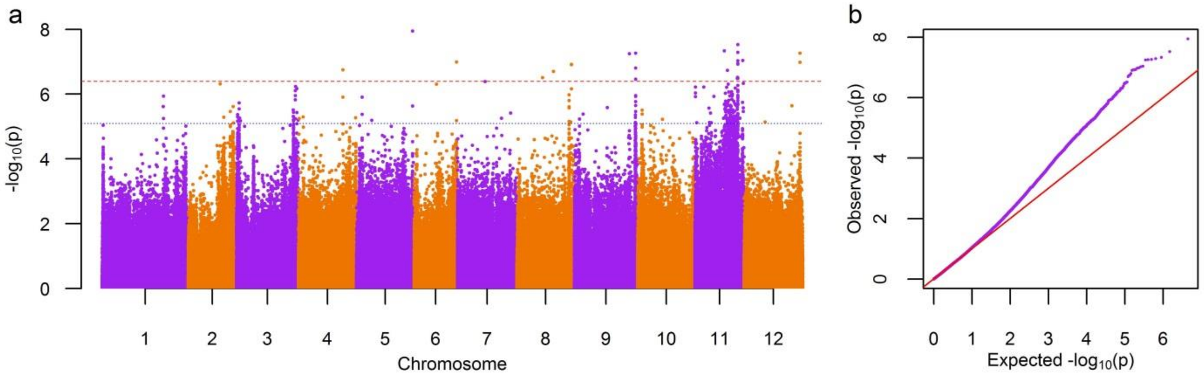

Supplementary Figure 61. Meta-analysis of genome-wide association of (E)-hexenal. (**a**) Manhattan plot of meta-analysis. (**b**) Quantile-quantile plot of meta-analysis.

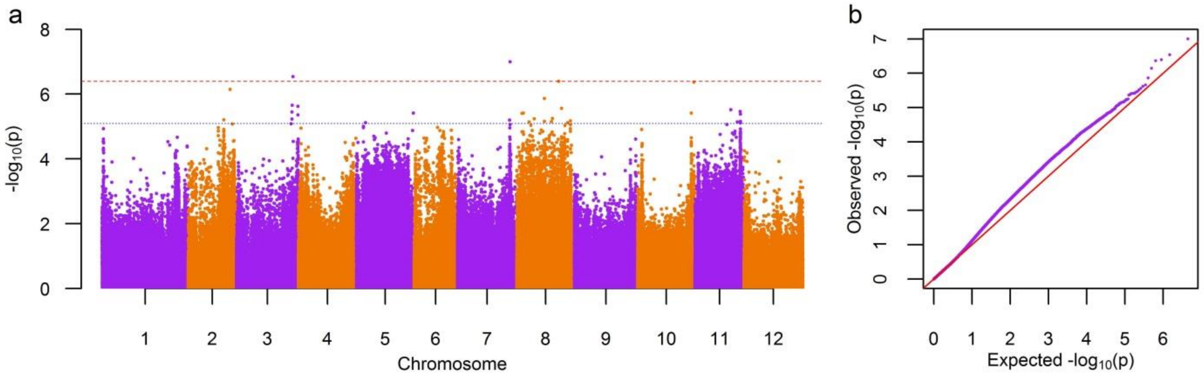

Supplementary Figure 62. Meta-analysis of genome-wide association of (E,E)-2,4-decadienal. (**a**) Manhattan plot of meta-analysis. (**b**) Quantile-quantile plot of meta-analysis.

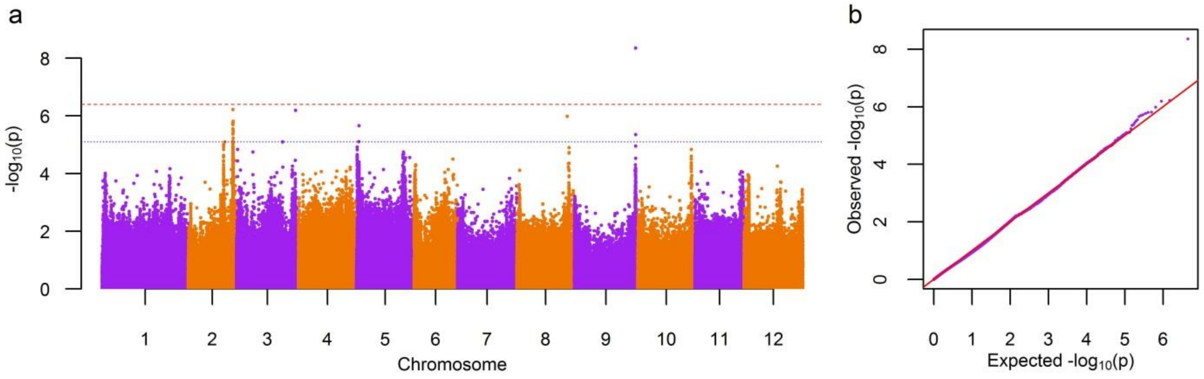

Supplementary Figure 63. Meta-analysis of genome-wide association of (E)-pentenal. (**a**) Manhattan plot of meta-analysis. (**b**) Quantile-quantile plot of meta-analysis.

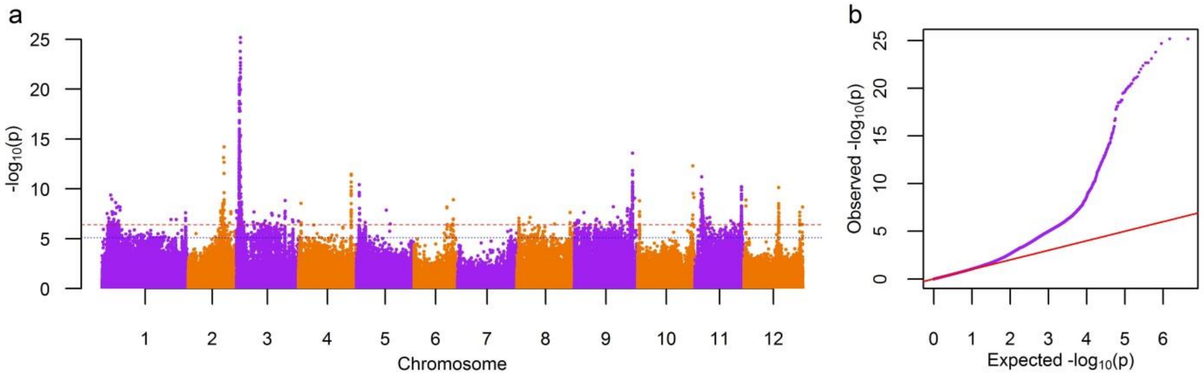

Supplementary Figure 64. Meta-analysis of genome-wide association of geranyl acetone. (**a**) Manhattan plot of meta-analysis. (**b**) Quantile-quantile plot of meta-analysis.

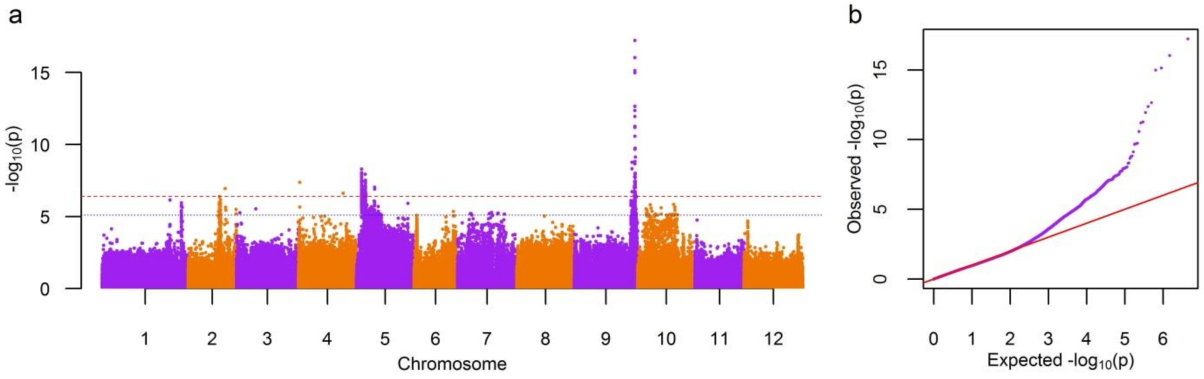

Supplementary Figure 65. Meta-analysis of genome-wide association of guaiacol. (**a**) Manhattan plot of meta-analysis. (**b**) Quantile-quantile plot of meta-analysis.

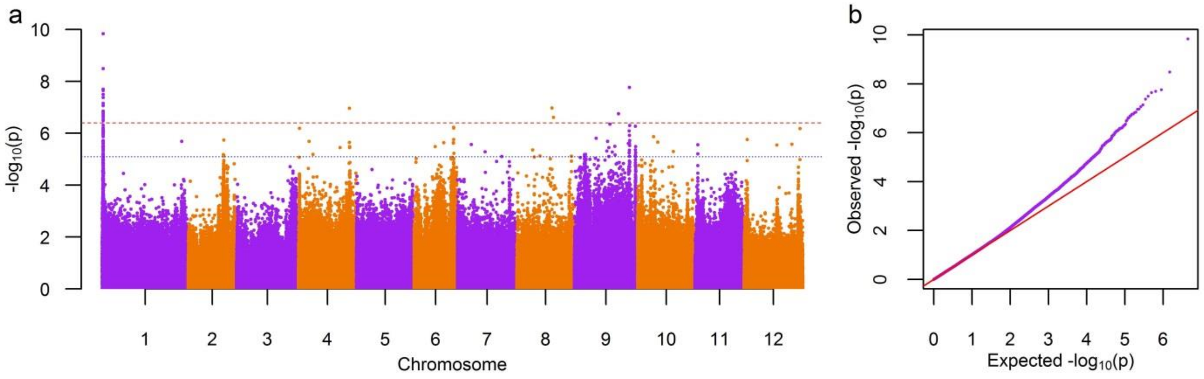

Supplementary Figure 66. Meta-analysis of genome-wide association of hexanal. (**a**) Manhattan plot of meta-analysis. (**b**) Quantile-quantile plot of meta-analysis.

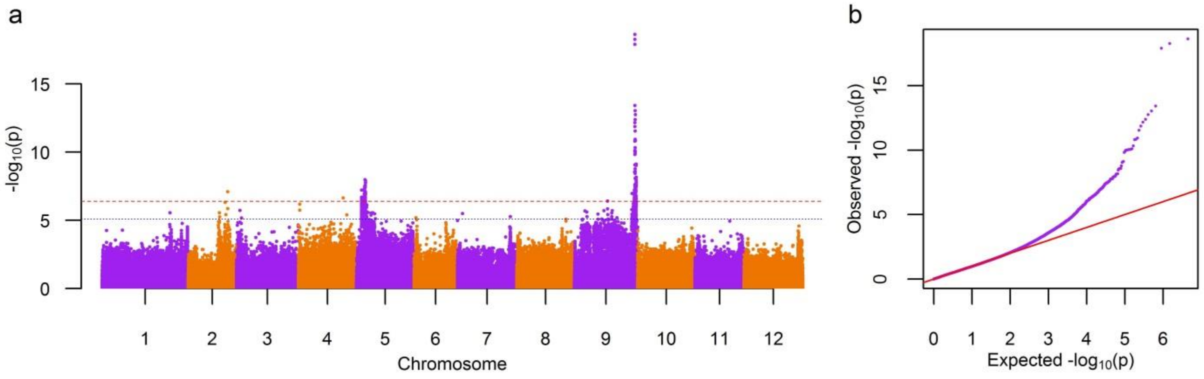

Supplementary Figure 67. Meta-analysis of genome-wide association of methyl salicylate. (**a**) Manhattan plot of meta-analysis. (**b**) Quantile-quantile plot of meta-analysis.

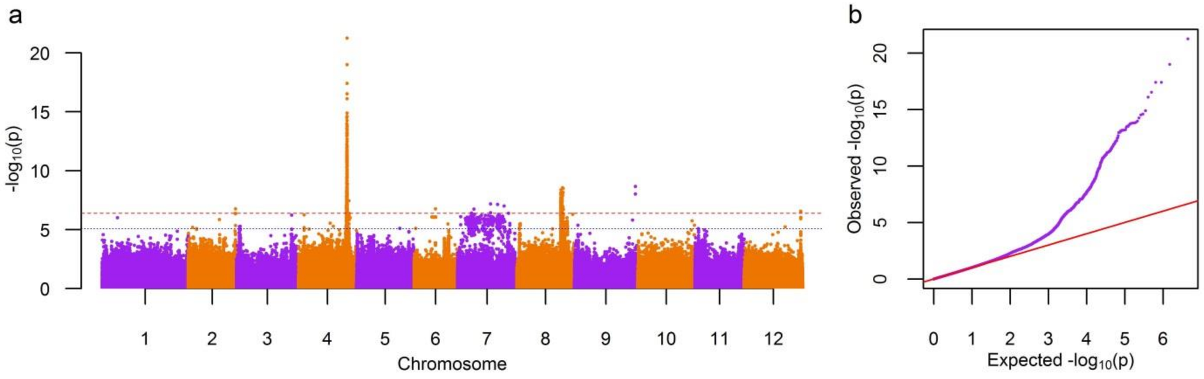

Supplementary Figure 68. Meta-analysis of genome-wide association of phenylacetaldehyde. (**a**) Manhattan plot of meta-analysis. (**b**) Quantile-quantile plot of meta-analysis.

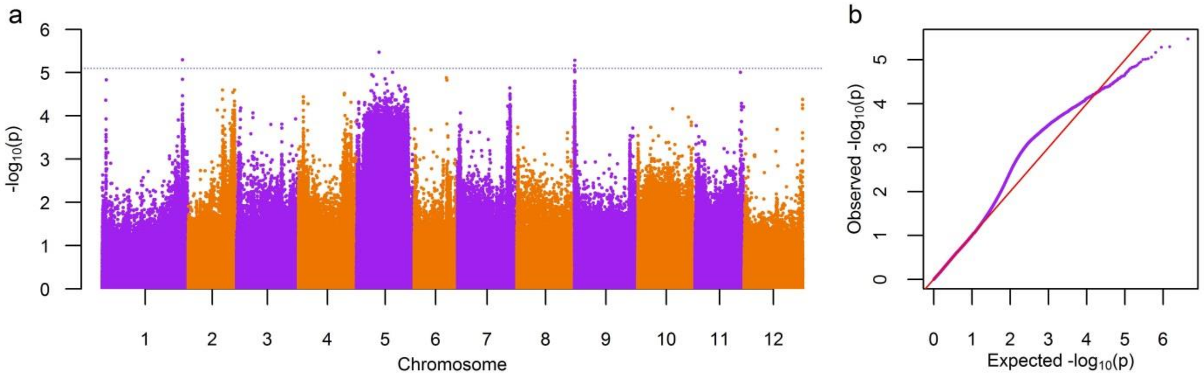

Supplementary Figure 69. Meta-analysis of genome-wide association of 1-octen-3-one. (**a**) Manhattan plot of meta-analysis. (**b**) Quantile-quantile plot of meta-analysis.

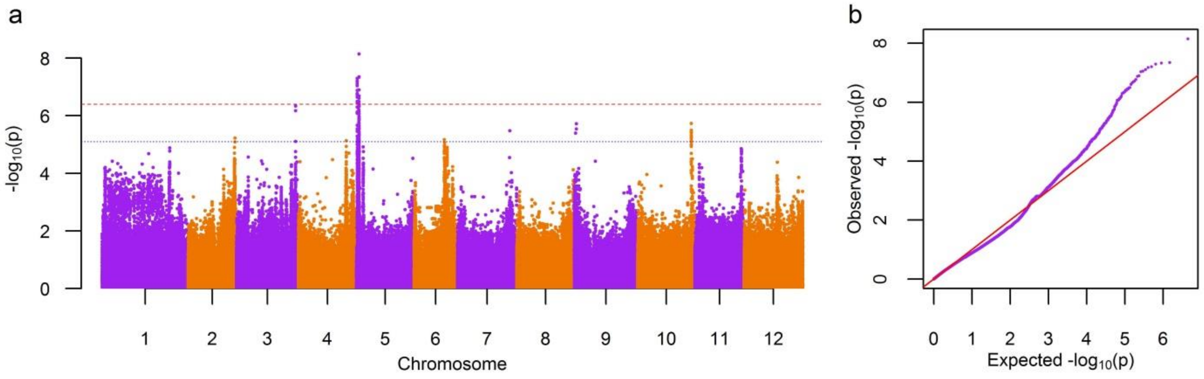

Supplementary Figure 70. Meta-analysis of genome-wide association of 1-penten-3-one. (**a**) Manhattan plot of meta-analysis. (**b**) Quantile-quantile plot of meta-analysis.

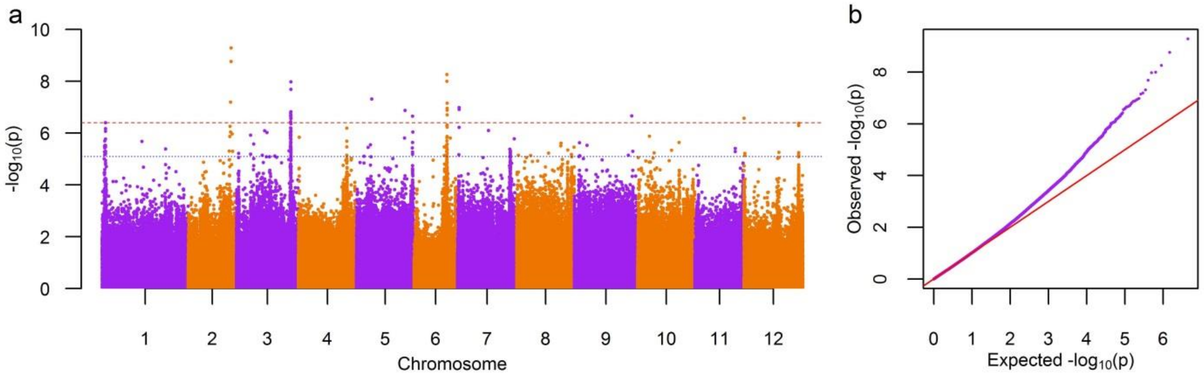

Supplementary Figure 71. Meta-analysis of genome-wide association of 2-methyl-1-butanol. (**a**) Manhattan plot of meta-analysis. (**b**) Quantile-quantile plot of meta-analysis.

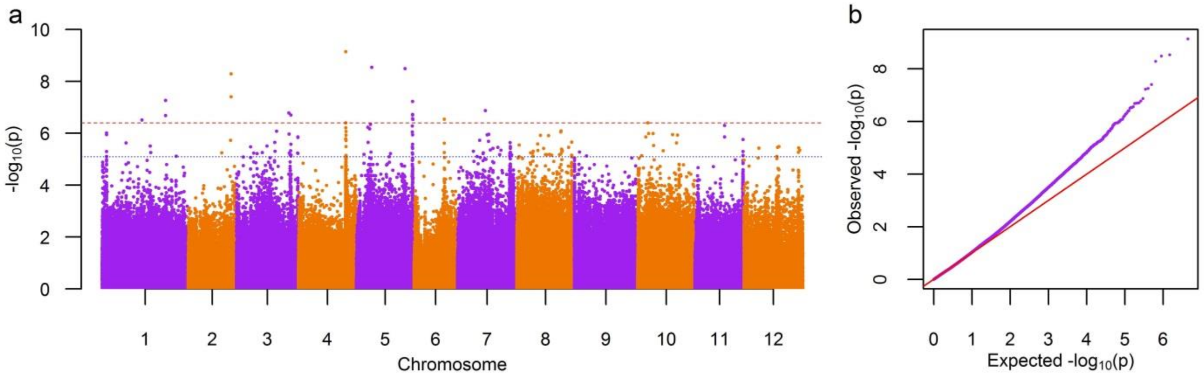

Supplementary Figure 72. Meta-analysis of genome-wide association of 3-methyl-1-butanol. (**a**) Manhattan plot of meta-analysis. (**b**) Quantile-quantile plot of meta-analysis.

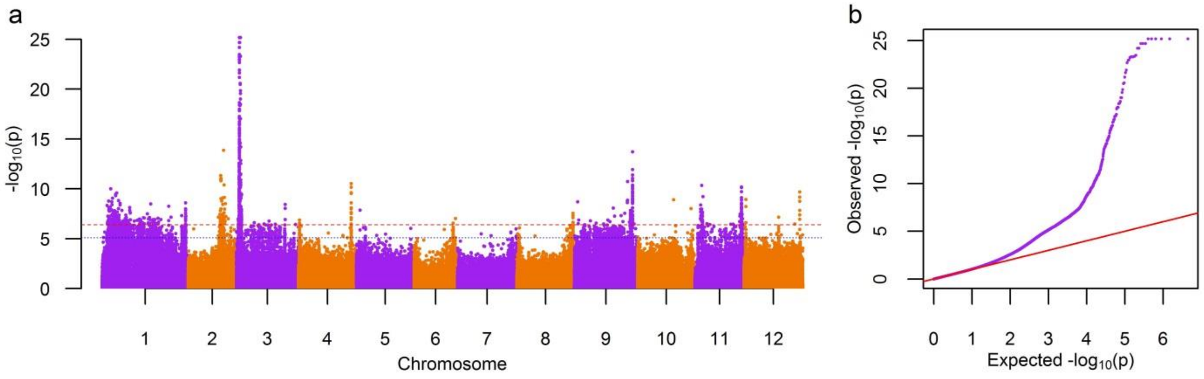

Supplementary Figure 73. Meta-analysis of genome-wide association of 6-methyl-5-hepten-2-one. (**a**) Manhattan plot of meta-analysis. (**b**) Quantile-quantile plot of meta-analysis.

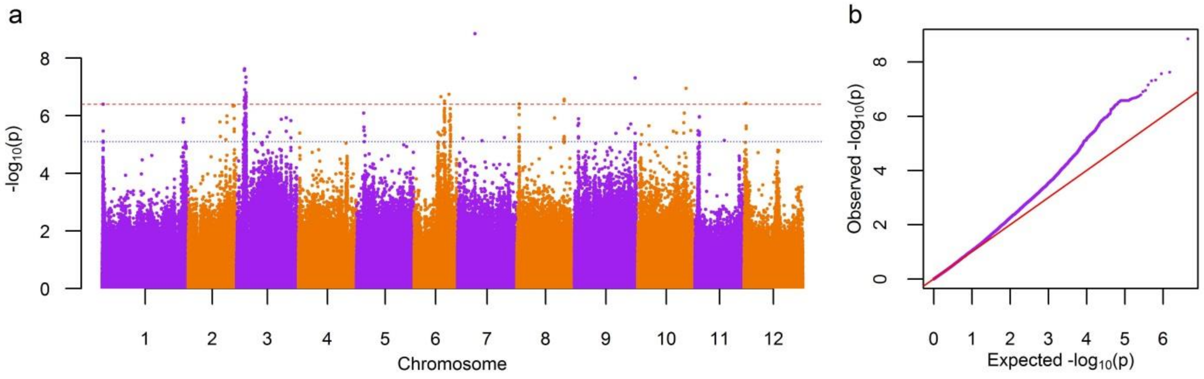

Supplementary Figure 74. Meta-analysis of genome-wide association of (Z)-3-hexen-1-ol. (**a**) Manhattan plot of meta-analysis. (**b**) Quantile-quantile plot of meta-analysis.

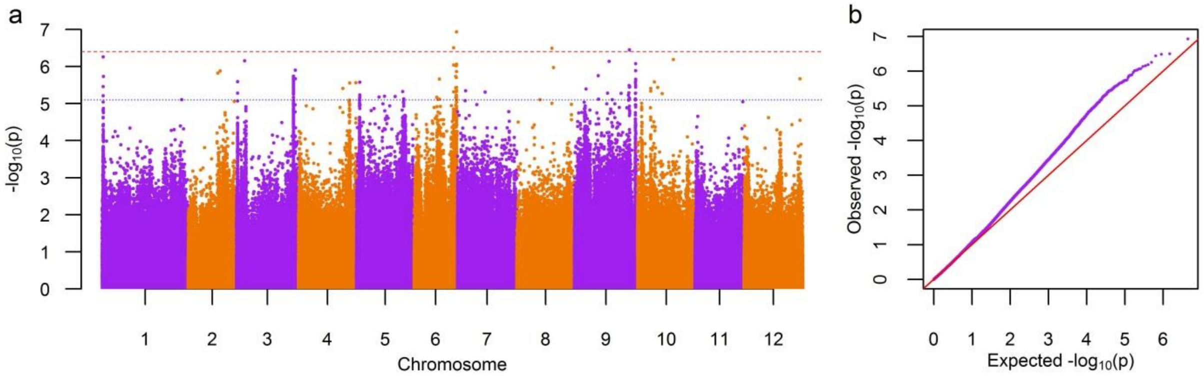

Supplementary Figure 75. Meta-analysis of genome-wide association of (Z)-3-hexenal. (**a**) Manhattan plot of meta-analysis. (**b**) Quantile-quantile plot of meta-analysis.

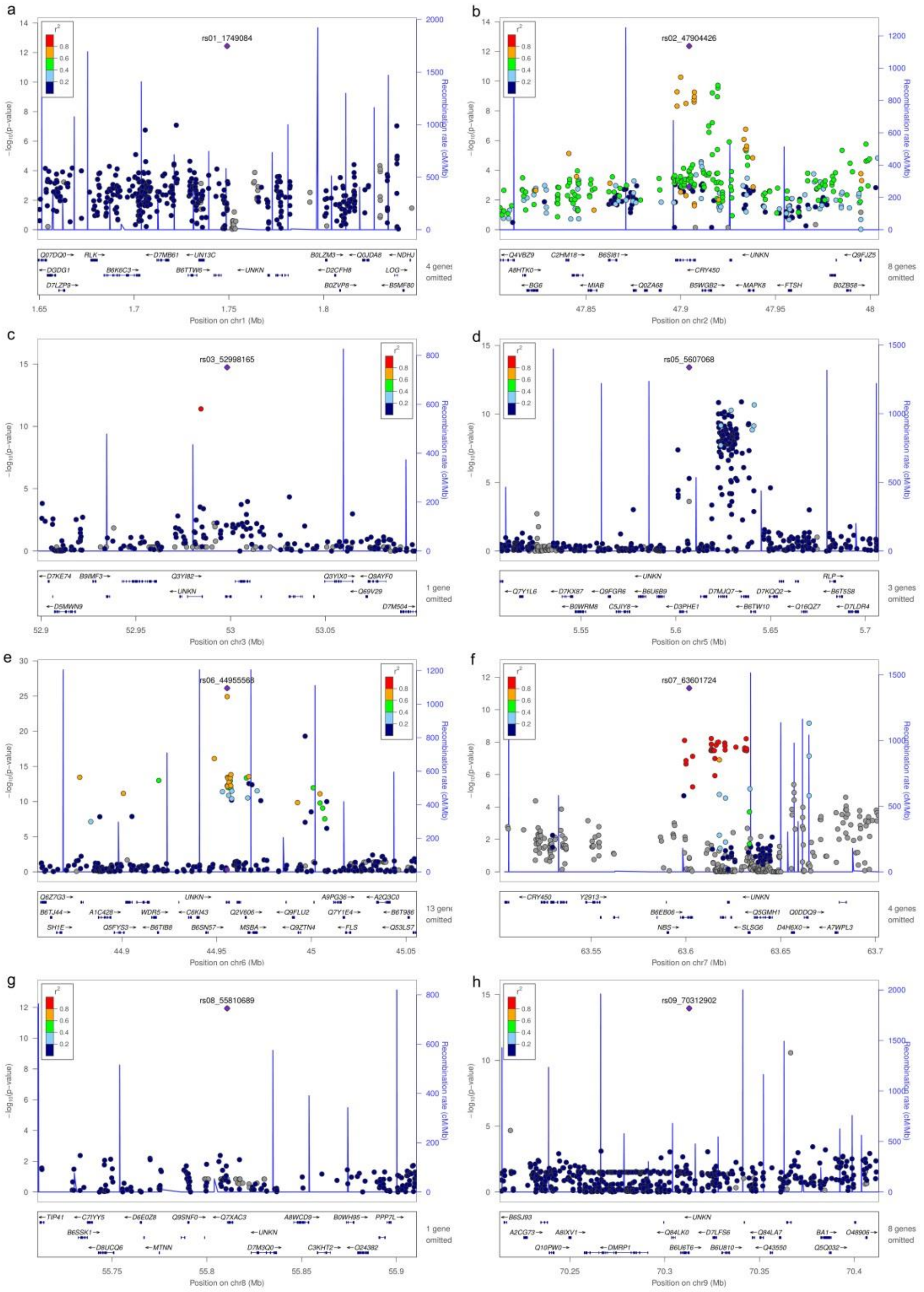

Supplementary Figure 76. LocusZoom plots of the previously identified significant associated loci in the meta-analysis of genome-wide associations of citrate. Associations are expressed as –$\log_{10}$(*P* value). The top SNPs are shown as purple squares within the window of 100 kb. Color legend represents the linkage disequilibrium between the top SNPs and SNPs appeared in the 1 Mb window size and was calculated based on the 221 reference panel. If the peak SNP was not in the reference panel, the genotype from panel T was used for the LD calculation. The blue line represents the recombination rate from the inferred recombination maps. **a-h** stands for different LocusZoom plot for each significant association.

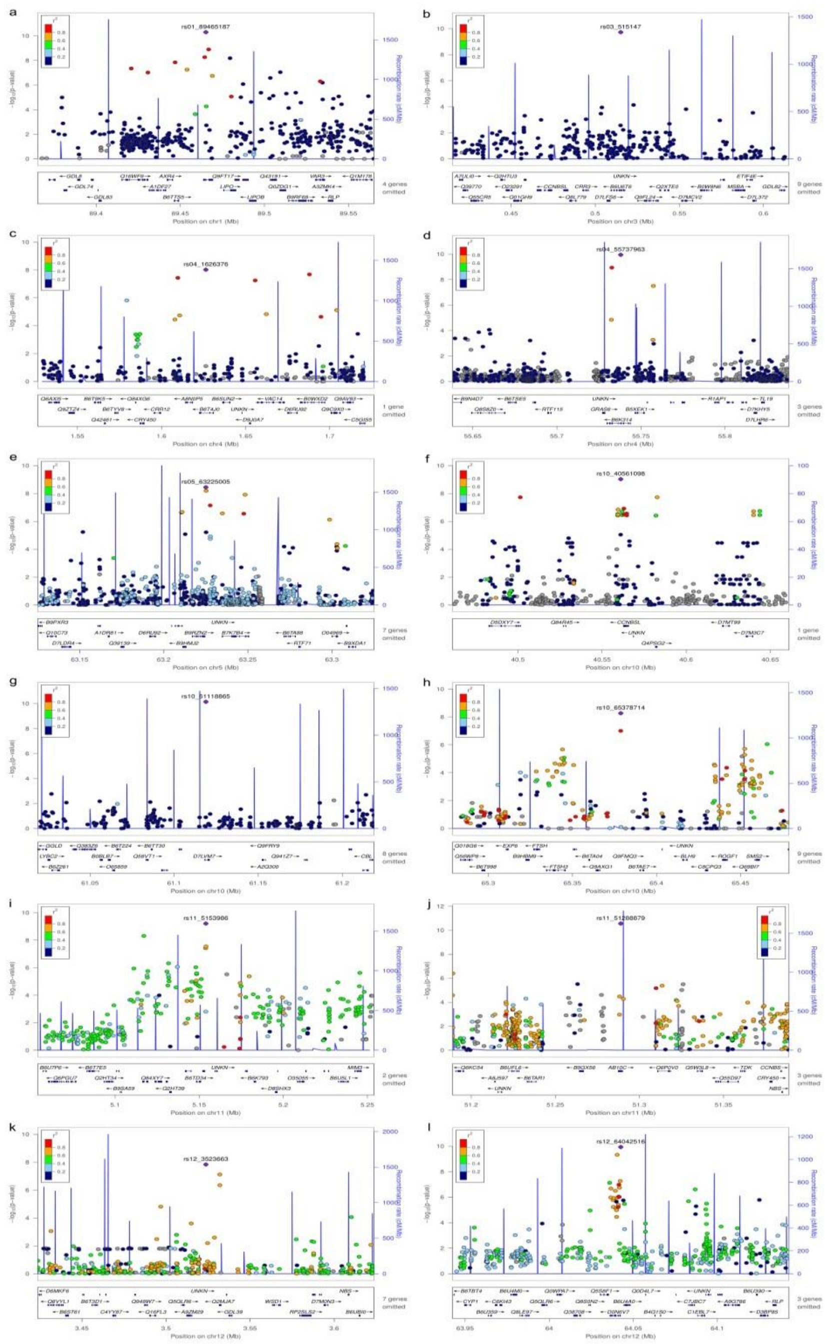

Supplementary Figure 77. LocusZoom plots of the newly identified significant associated loci in the meta-analysis of genome-wide associations of citrate. Associations are expressed as $-\log_{10}$(P value). The top SNPs are shown as purple squares within the window of 100 kb. Color legend represents the linkage disequilibrium between the top SNPs and SNPs appeared in the 1 Mb window size and was calculated based on the 221 reference panel. If the peak SNP was not in the reference panel, the genotype from panel T was used for the LD calculation. The blue line represents the recombination rate from the inferred recombination maps. **a-i** stands for different LocusZoom plot for each significant association.

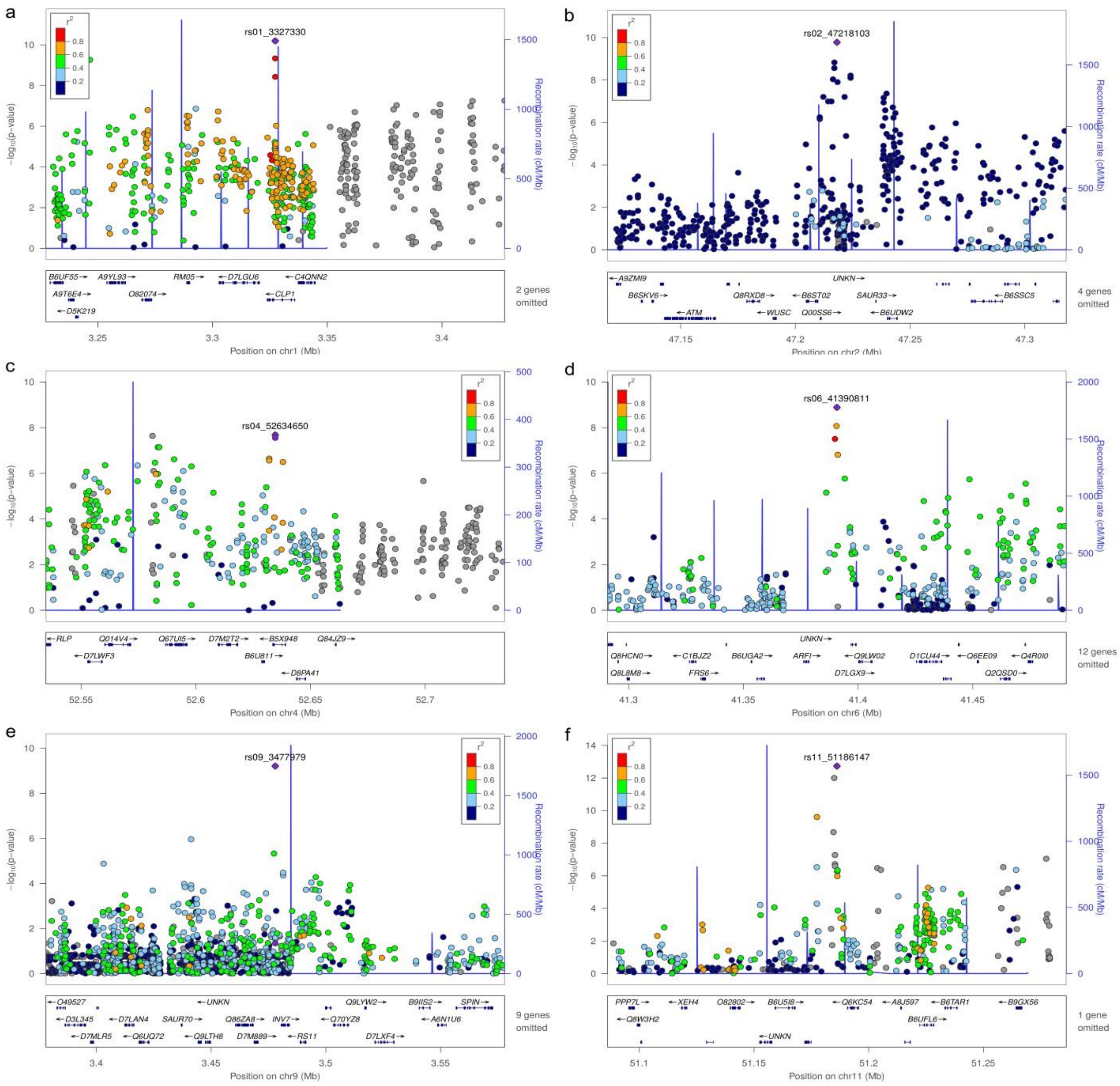

Supplementary Figure 78. LocusZoom plots of the previously identified significant associated loci in the meta-analysis of genome-wide associations of fructose. Associations are expressed as –$\log_{10}$(P value). The top SNPs are shown as purple squares within the window of 100 kb. Color legend represents the linkage disequilibrium between the top SNPs and SNPs appeared in the 1 Mb window size and was calculated based on the 221 reference panel. If the peak SNP was not in the reference panel, the genotype from panel T was used for the LD calculation. The blue line represents the recombination rate from the inferred recombination maps. **a-f** stands for different LocusZoom plot for each significant association.

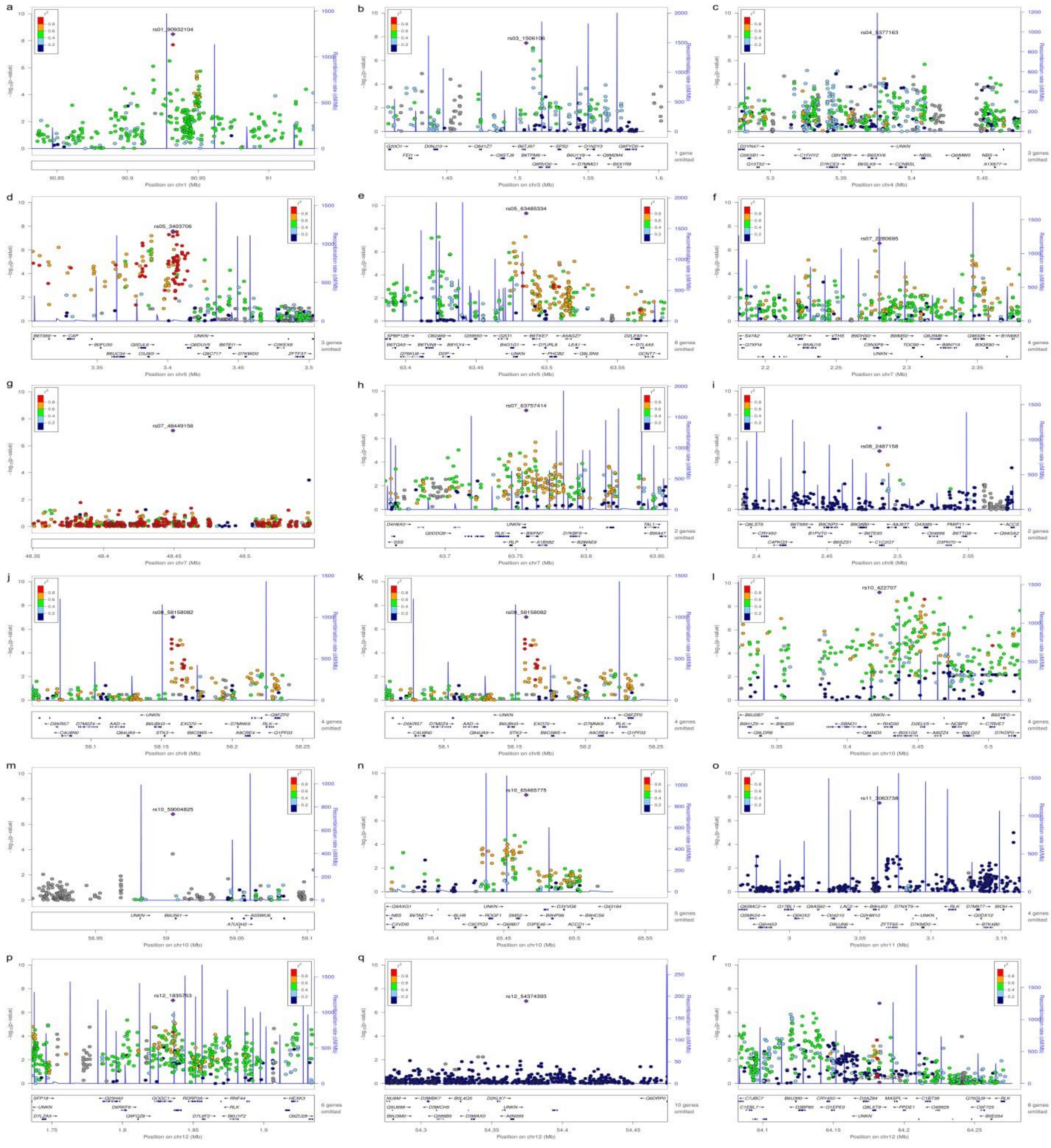

Supplementary Figure 79. LocusZoom plots of the newly identified significant associated loci in the meta-analysis of genome-wide associations of fructose. Associations are expressed as $-\log_{10}$(P value). The top SNPs are shown as purple squares within the window of 100 kb. Color legend represents the linkage disequilibrium between the top SNPs and SNPs appeared in the 1 Mb window size and was calculated based on the 221 reference panel. If the peak SNP was not in the reference panel, the genotype from panel T was used for the LD calculation. The blue line represents the recombination rate from the inferred recombination maps. **a-r** stands for different LocusZoom plot for each significant association.

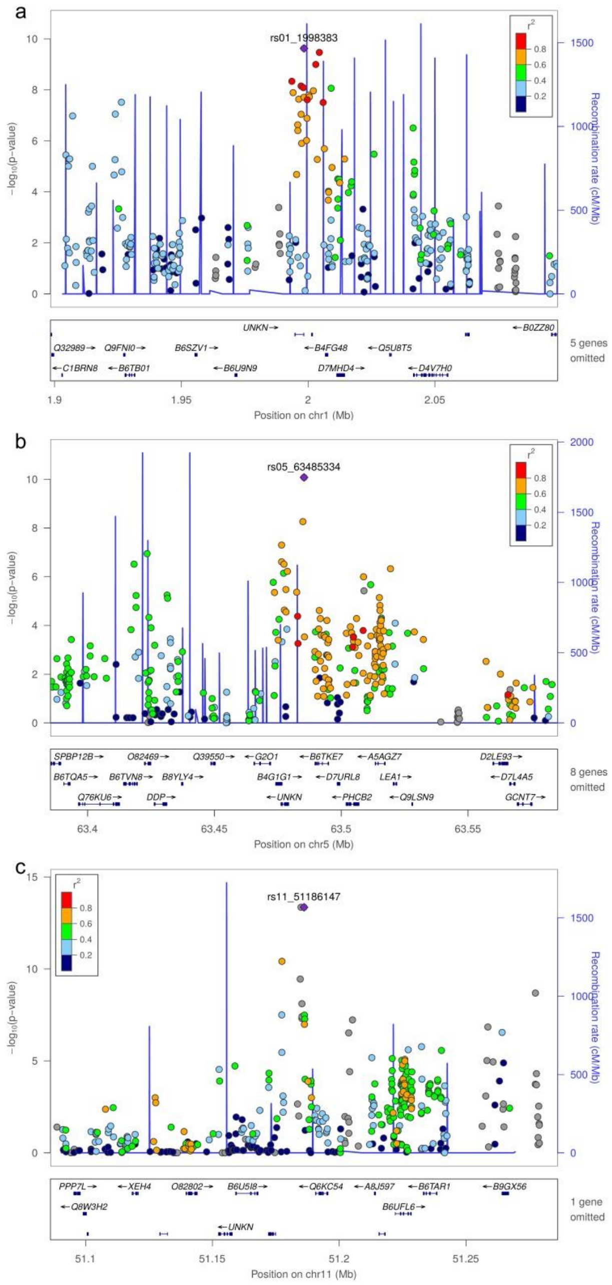

Supplementary Figure 80. LocusZoom plots of the previously identified significant associated loci in the meta-analysis of genome-wide associations of glucose. Associations are expressed as $-\log_{10}(P \text{ value})$. The top SNPs are shown as purple squares within the window of 100 kb. Color legend represents the linkage disequilibrium between the top SNPs and SNPs appeared in the 1 Mb window size and was calculated based on the 221 reference panel. If the peak SNP was not in the reference panel, the genotype from panel T was used for the LD calculation. The blue line represents the recombination rate from the inferred recombination maps. **a-c** stands for different LocusZoom plot for each significant association.

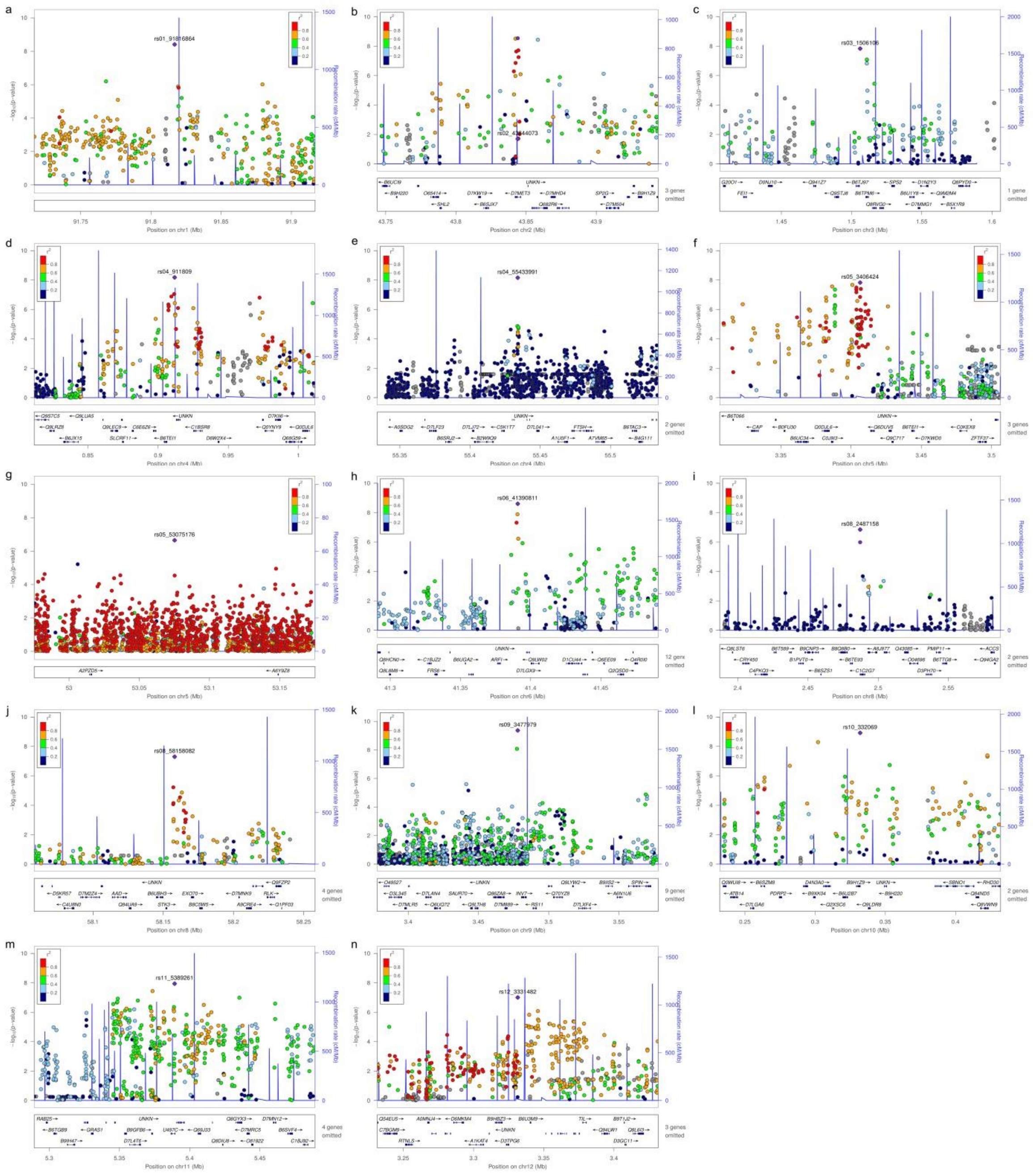

Supplementary Figure 81. LocusZoom plots of the newly identified significant associated loci in the meta-analysis of genome-wide associations of glucose. Associations are expressed as $-\log_{10}(P \text{ value})$. The top SNPs are shown as purple squares within the window of 100 kb. Color legend represents the linkage disequilibrium between the top SNPs and SNPs appeared in the 1 Mb window size and was calculated based on the 221 reference panel. If the peak SNP was not in the reference panel, the genotype from panel T was used for the LD calculation. The blue line represents the recombination rate from the inferred recombination maps. **a-n** stands for different LocusZoom plot for each significant association.

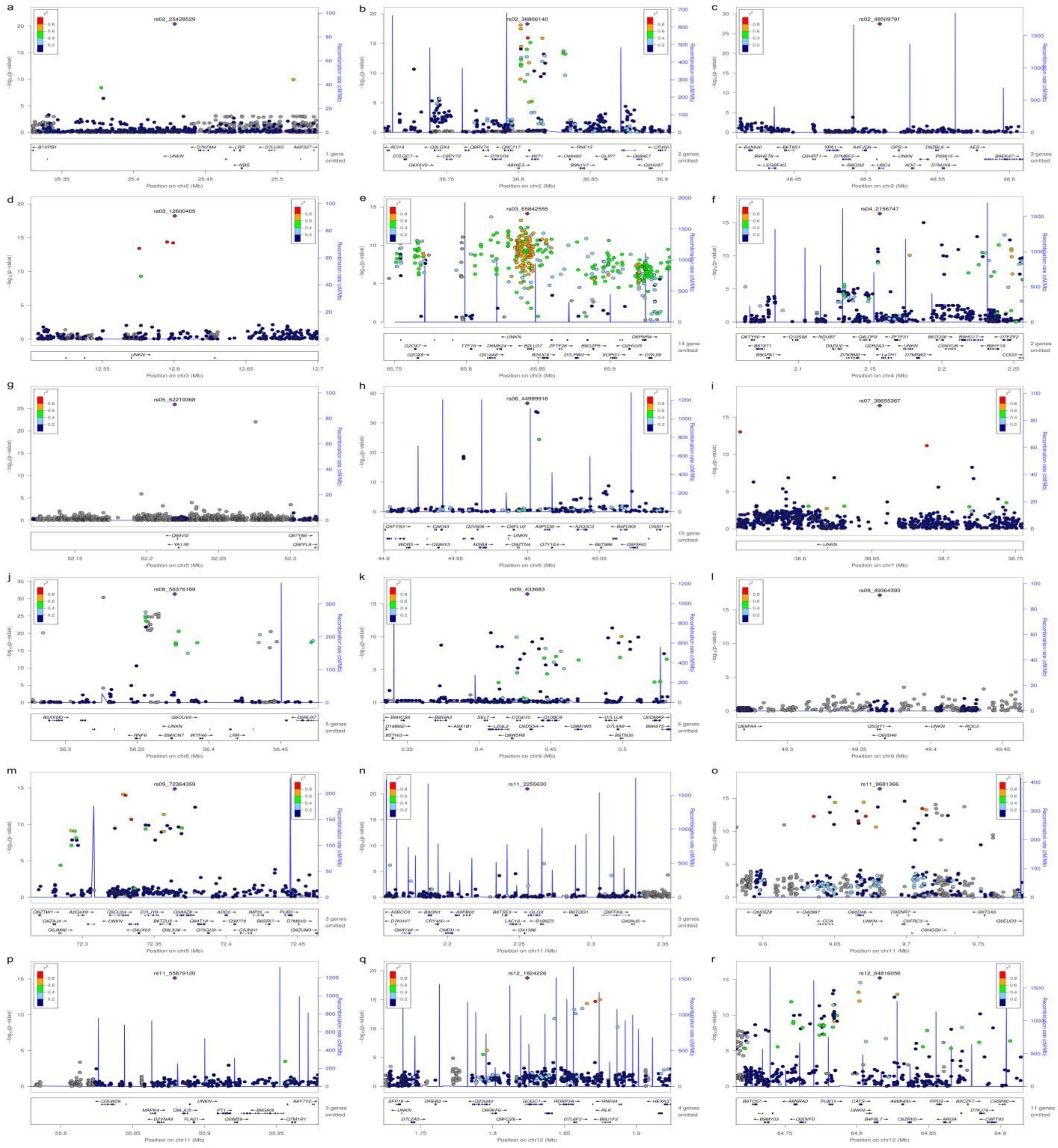

Supplementary Figure 82. LocusZoom plots of the previously identified significant associated loci in the meta-analysis of genome-wide associations of malate. Associations are expressed as –$\log_{10}$(P value). The top SNPs are shown as purple squares within the window of 100 kb. Color legend represents the linkage disequilibrium between the top SNPs and SNPs appeared in the 1 Mb window size and was calculated based on the 221 reference panel. If the peak SNP was not in the reference panel, the genotype from panel T was used for the LD calculation. The blue line represents the recombination rate from the inferred recombination maps. **a-r** stands for different LocusZoom plot for each significant association.

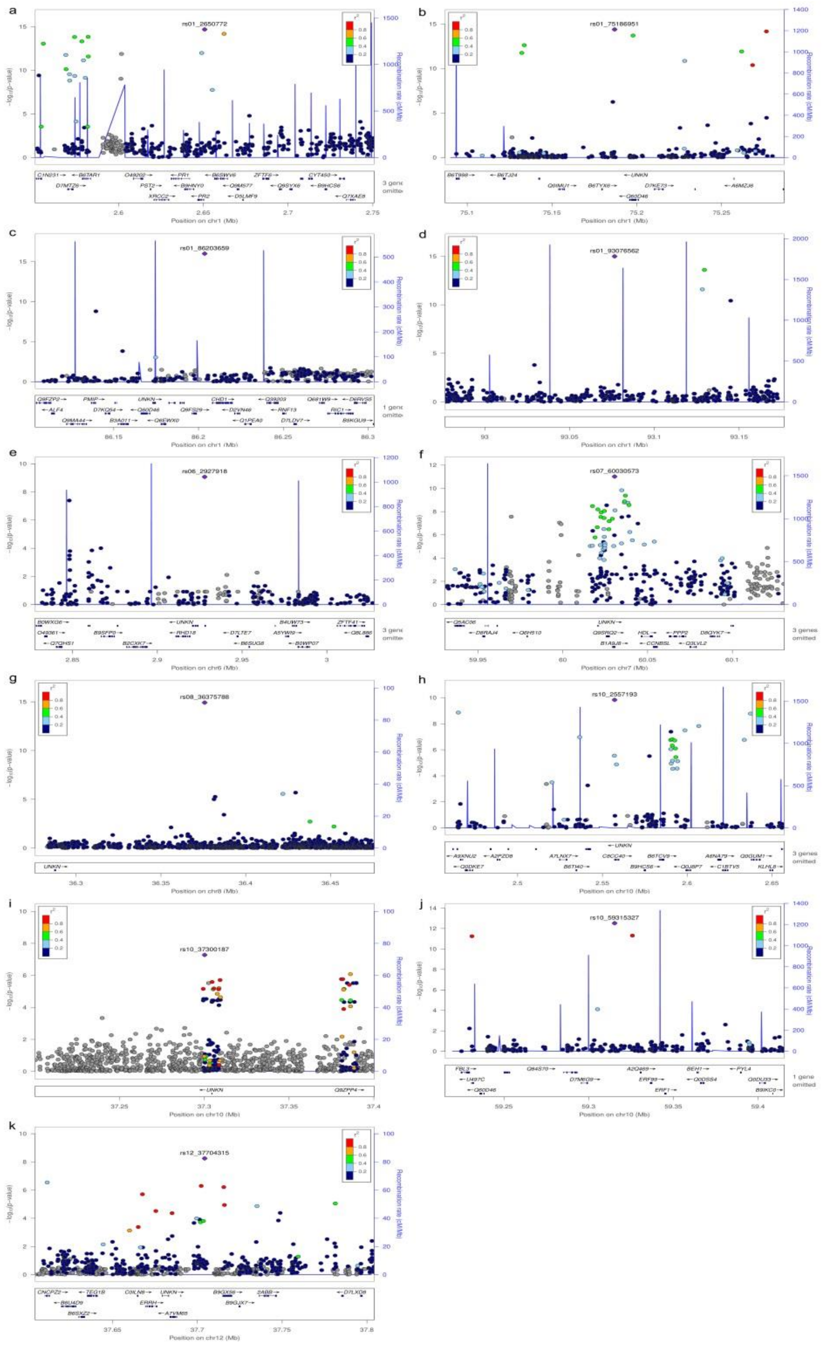

Supplementary Figure 83. LocusZoom plots of the newly identified significant associated loci in the meta-analysis of genome-wide associations of malate. Associations are expressed as $-\log_{10}(P$ value). The top SNPs are shown as purple squares within the window of 100 kb. Color legend represents the linkage disequilibrium between the top SNPs and SNPs appeared in the 1 Mb window size and was calculated based on the 221 reference panel. If the peak SNP was not in the reference panel, the genotype from panel T was used for the LD calculation. The blue line represents the recombination rate from the inferred recombination maps. **a-k** stands for different LocusZoom plot for each significant association.

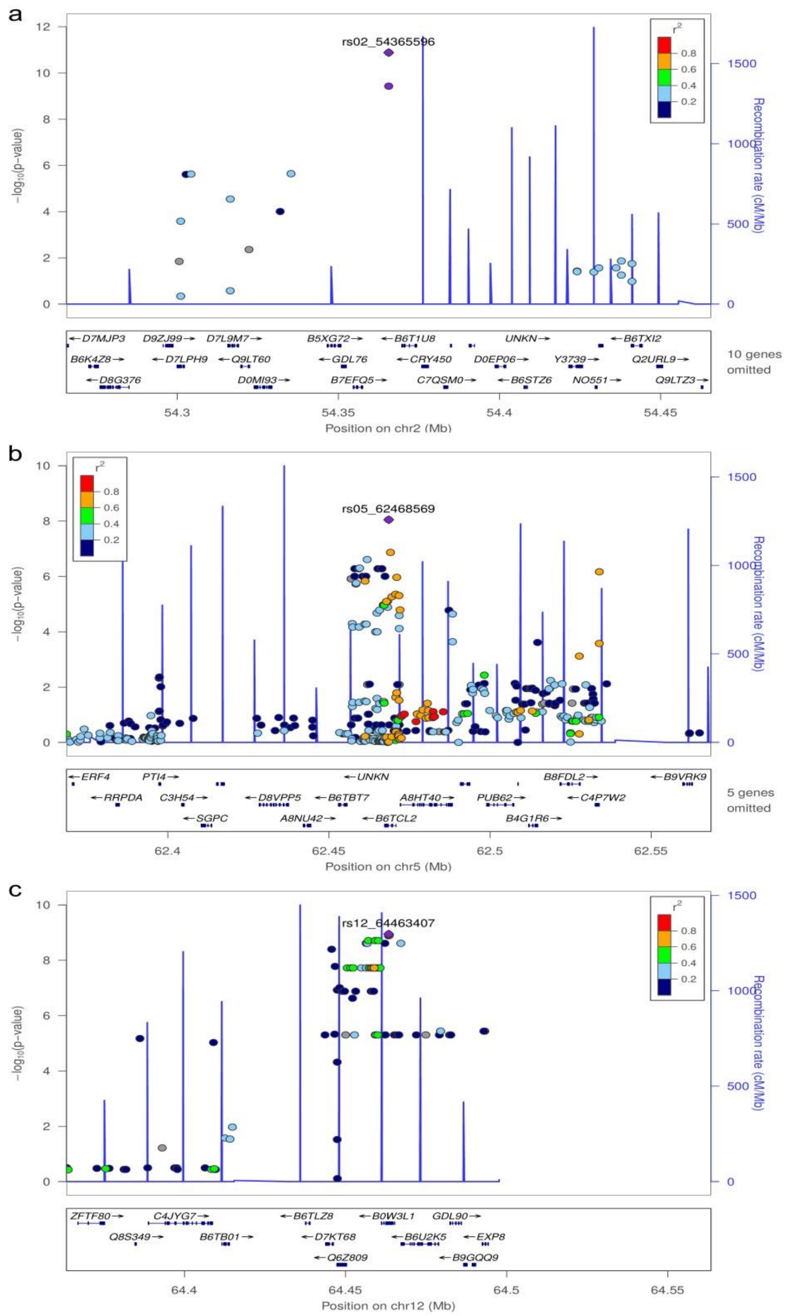

Supplementary Figure 84. LocusZoom plots of the previously identified significant associated loci in the meta-analysis of genome-wide associations of asparagine. Associations are expressed as $-\log_{10}$(*P* value). The top SNPs are shown as purple squares within the window of 100 kb. Color legend represents the linkage disequilibrium between the top SNPs and SNPs appeared in the 1 Mb window size and was calculated based on the 221 reference panel. If the peak SNP was not in the reference panel, the genotype from panel T was used for the LD calculation. The blue line represents the recombination rate from the inferred recombination maps. **a-c** stands for different LocusZoom plot for each significant association.

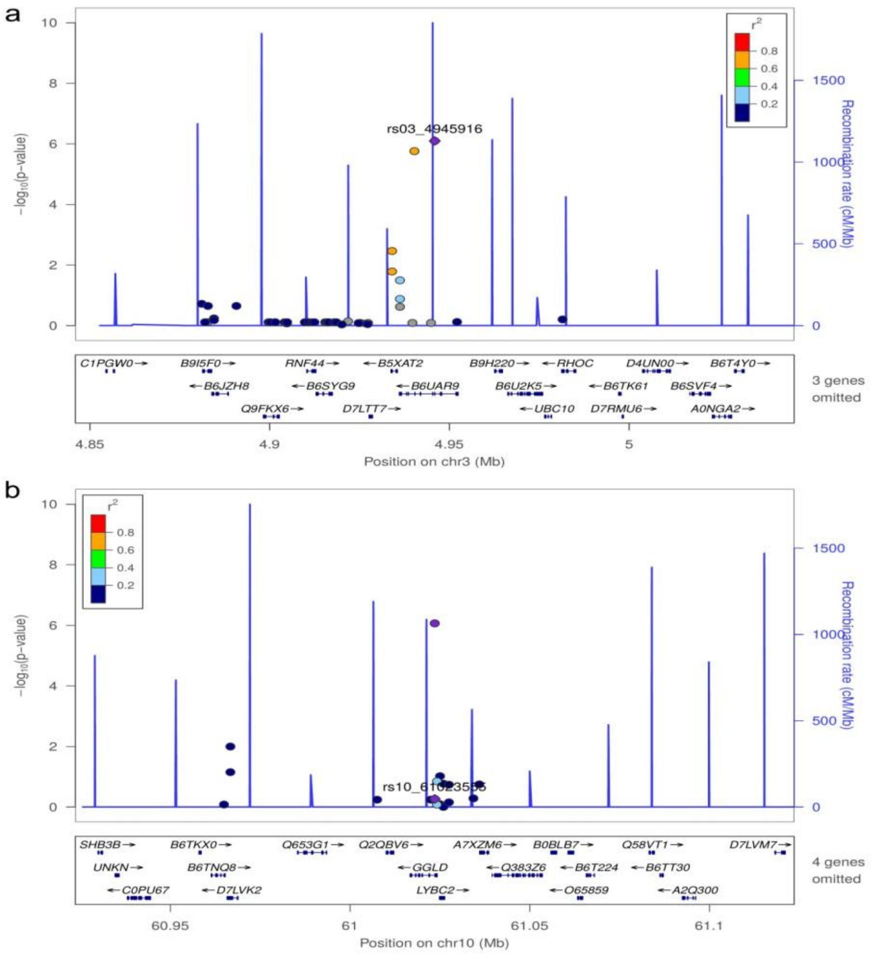

Supplementary Figure 85. LocusZoom plots of the newly identified significant associated loci in the meta-analysis of genome-wide associations of asparagine. Associations are expressed as $-\log_{10}(P \text{ value})$. The top SNPs are shown as purple squares within the window of 100 kb. Color legend represents the linkage disequilibrium between the top SNPs and SNPs appeared in the 1 Mb window size and was calculated based on the 221 reference panel. If the peak SNP was not in the reference panel, the genotype from panel T was used for the LD calculation. The blue line represents the recombination rate from the inferred recombination maps. **a-b** stands for different LocusZoom plot for each significant association.

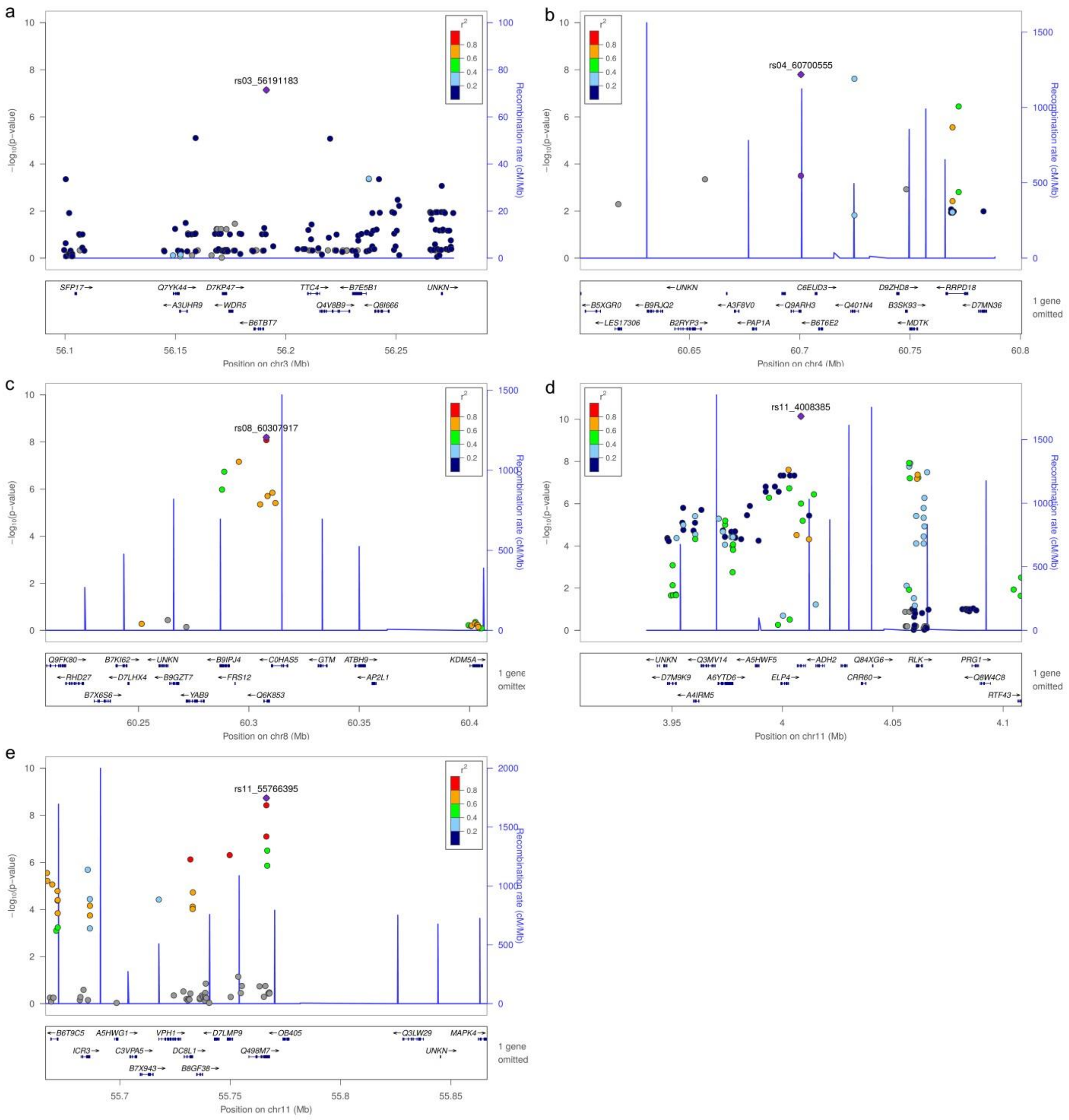

Supplementary Figure 86. LocusZoom plots of the previously identified significant associated loci in the meta-analysis of genome-wide associations of aspartate. Associations are expressed as –$\log_{10}$(P value). The top SNPs are shown as purple squares within the window of 100 kb. Color legend represents the linkage disequilibrium between the top SNPs and SNPs appeared in the 1 Mb window size and was calculated based on the 221 reference panel. If the peak SNP was not in the reference panel, the genotype from panel T was used for the LD calculation. The blue line represents the recombination rate from the inferred recombination maps. **a-e** stands for different LocusZoom plot for each significant association.

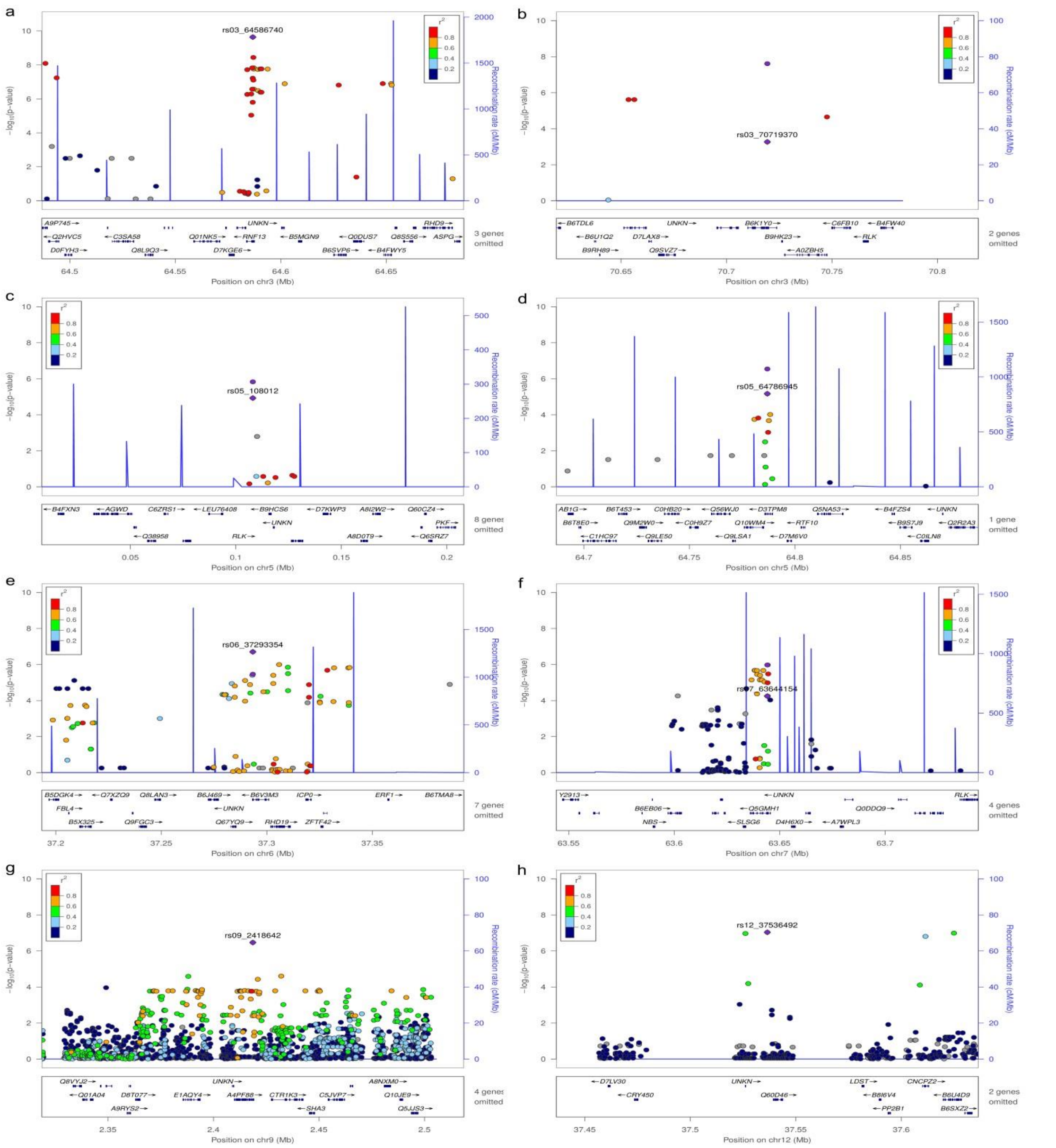

Supplementary Figure 87. LocusZoom plots of the newly identified significant associated loci in the meta-analysis of genome-wide associations of aspartate. Associations are expressed as –$\log_{10}$(P value). The top SNPs are shown as purple squares within the window of 100 kb. Color legend represents the linkage disequilibrium between the top SNPs and SNPs appeared in the 1 Mb window size and was calculated based on the 221 reference panel. If the peak SNP was not in the reference panel, the genotype from panel T was used for the LD calculation. The blue line represents the recombination rate from the inferred recombination maps. **a-h** stands for different LocusZoom plot for each significant association.

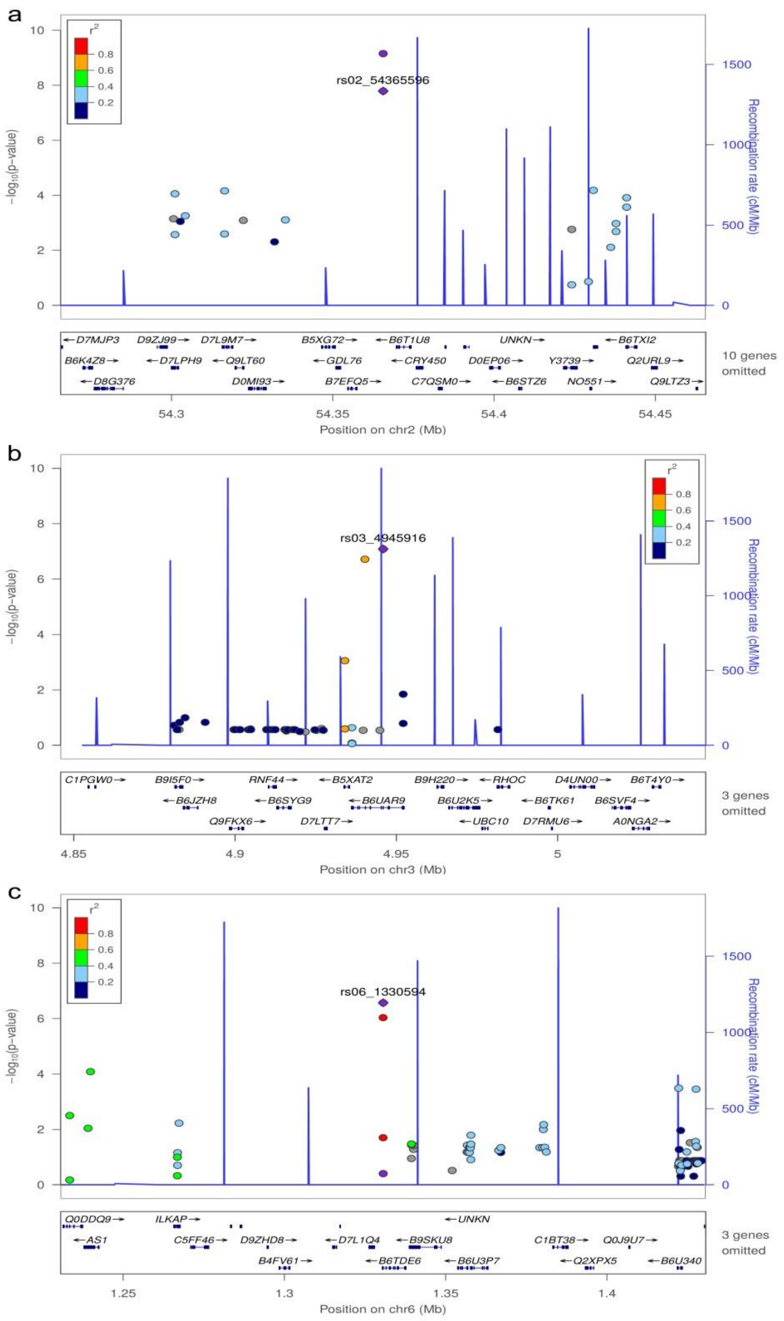

Supplementary Figure 88. LocusZoom plots of the previously identified significant associated loci in the meta-analysis of genome-wide associations of GABA. Associations are expressed as –log$_{10}$(P value). The top SNPs are shown as purple squares within the window of 100 kb. Color legend represents the linkage disequilibrium between the top SNPs and SNPs appeared in the 1 Mb window size and was calculated based on the 221 reference panel. If the peak SNP was not in the reference panel, the genotype from panel T was used for the LD calculation. The blue line represents the recombination rate from the inferred recombination maps. **a-c** stands for different LocusZoom plot for each significant association.

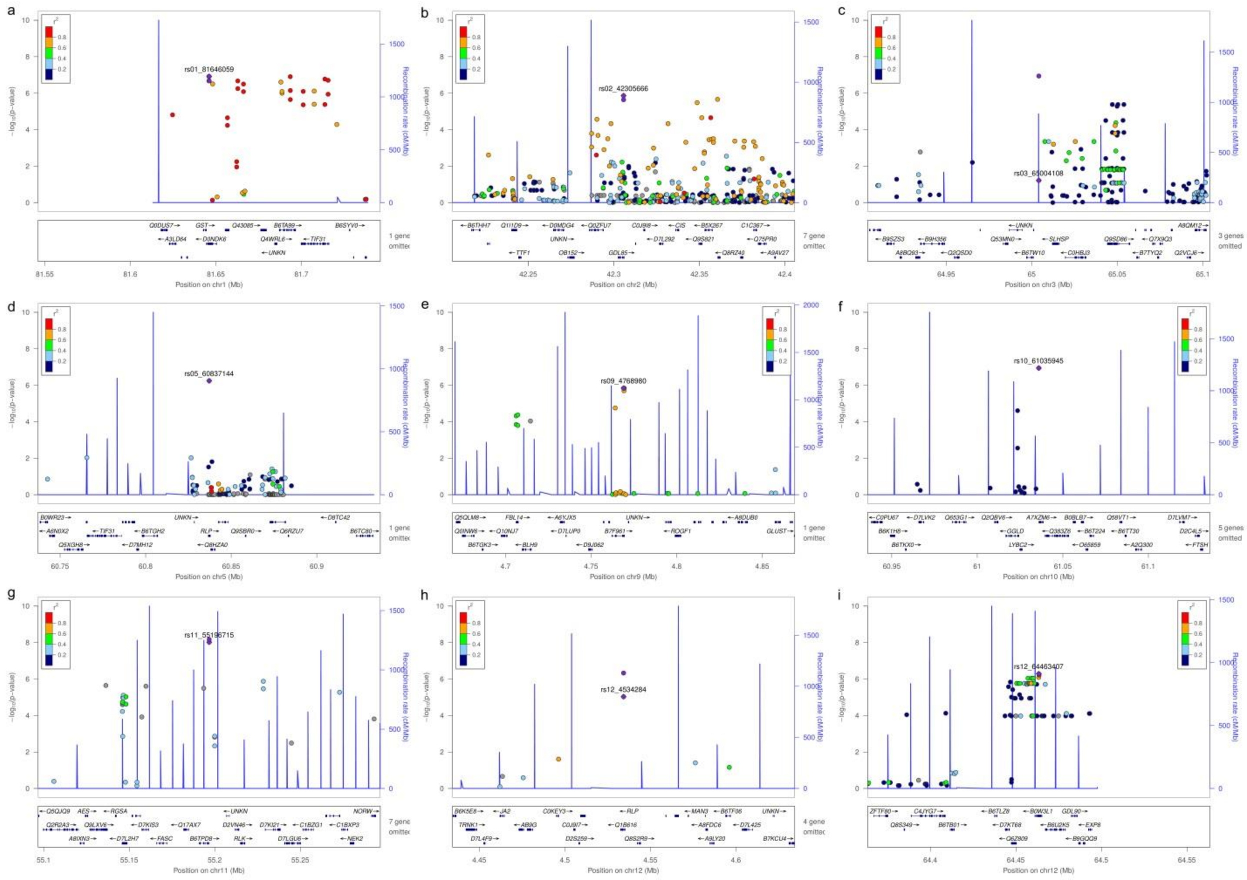

Supplementary Figure 89. LocusZoom plots of the newly identified significant associated loci in the meta-analysis of genome-wide associations of GABA. Associations are expressed as $-\log_{10}$(*P* value). The top SNPs are shown as purple squares within the window of 100 kb. Color legend represents the linkage disequilibrium between the top SNPs and SNPs appeared in the 1 Mb window size and was calculated based on the 221 reference panel. If the peak SNP was not in the reference panel, the genotype from panel T was used for the LD calculation. The blue line represents the recombination rate from the inferred recombination maps. **a-i** stands for different LocusZoom plot for each significant association.

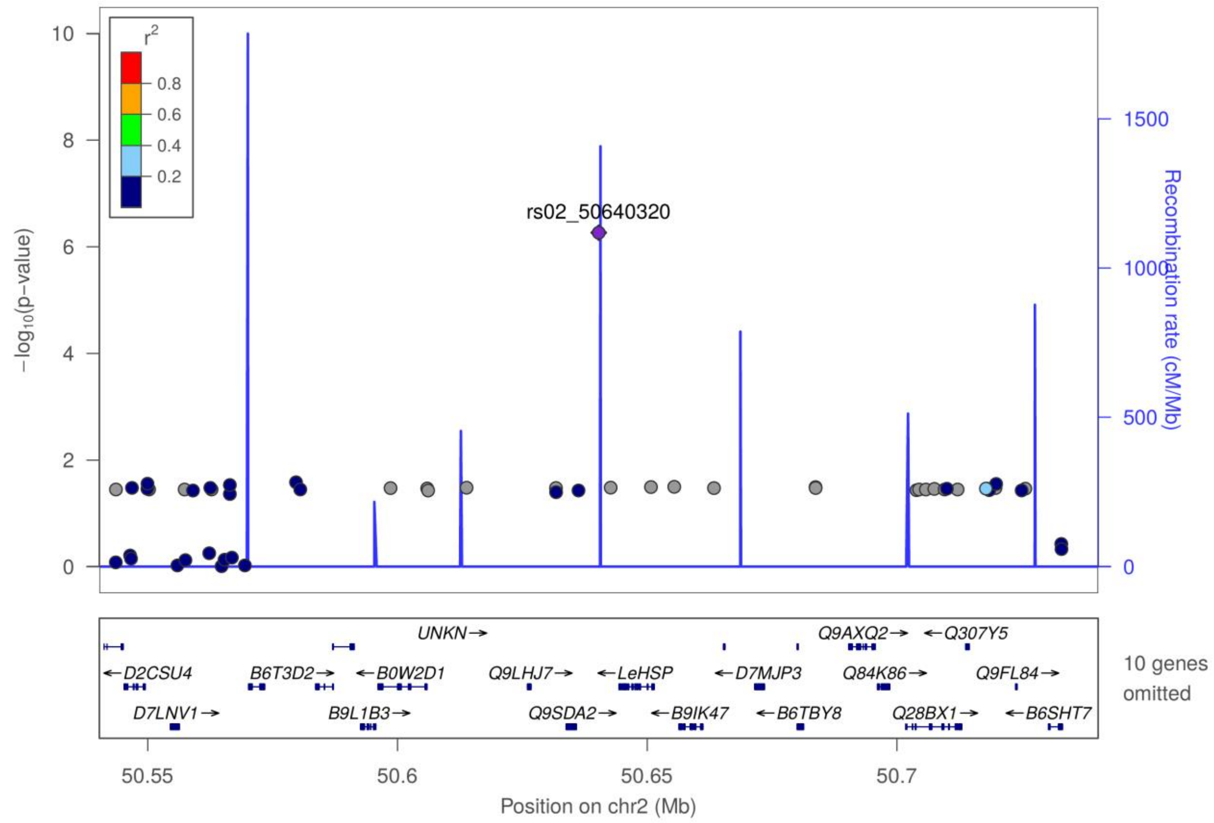

Supplementary Figure 90. LocusZoom plots of the previously identified significant associated loci in the meta-analysis of genome-wide associations of glutamine. Associations are expressed as –$\log_{10}$(*P* value). The top SNPs are shown as purple squares within the window of 100 kb. Color legend represents the linkage disequilibrium between the top SNPs and SNPs appeared in the 1 Mb window size and was calculated based on the 221 reference panel. If the peak SNP was not in the reference panel, the genotype from panel T was used for the LD calculation. The blue line represents the recombination rate from the inferred recombination maps.

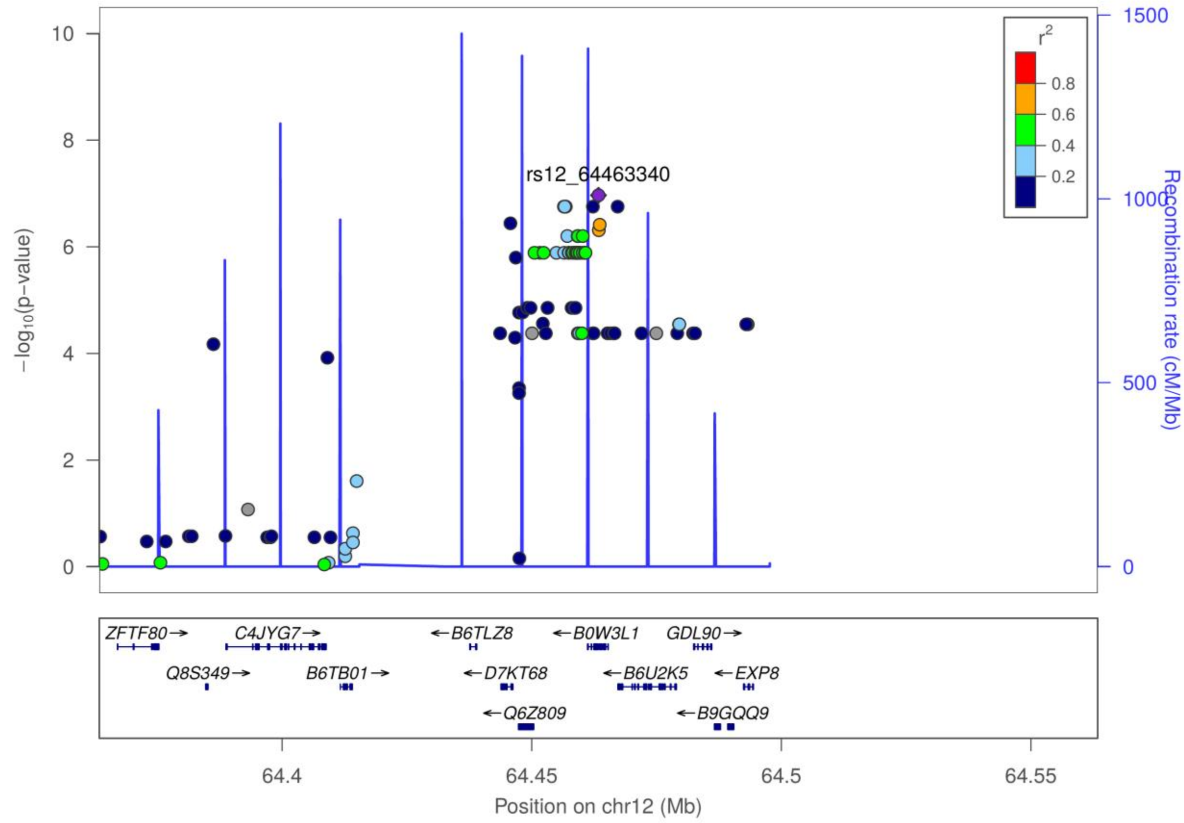

Supplementary Figure 91. LocusZoom plots of the newly identified significant associated loci in the meta-analysis of genome-wide associations of glutamine. Associations are expressed as –$\log_{10}$(*P* value). The top SNPs are shown as purple squares within the window of 100 kb. Color legend represents the linkage disequilibrium between the top SNPs and SNPs appeared in the 1 Mb window size and was calculated based on the 221 reference panel. If the peak SNP was not in the reference panel, the genotype from panel T was used for the LD calculation. The blue line represents the recombination rate from the inferred recombination maps.

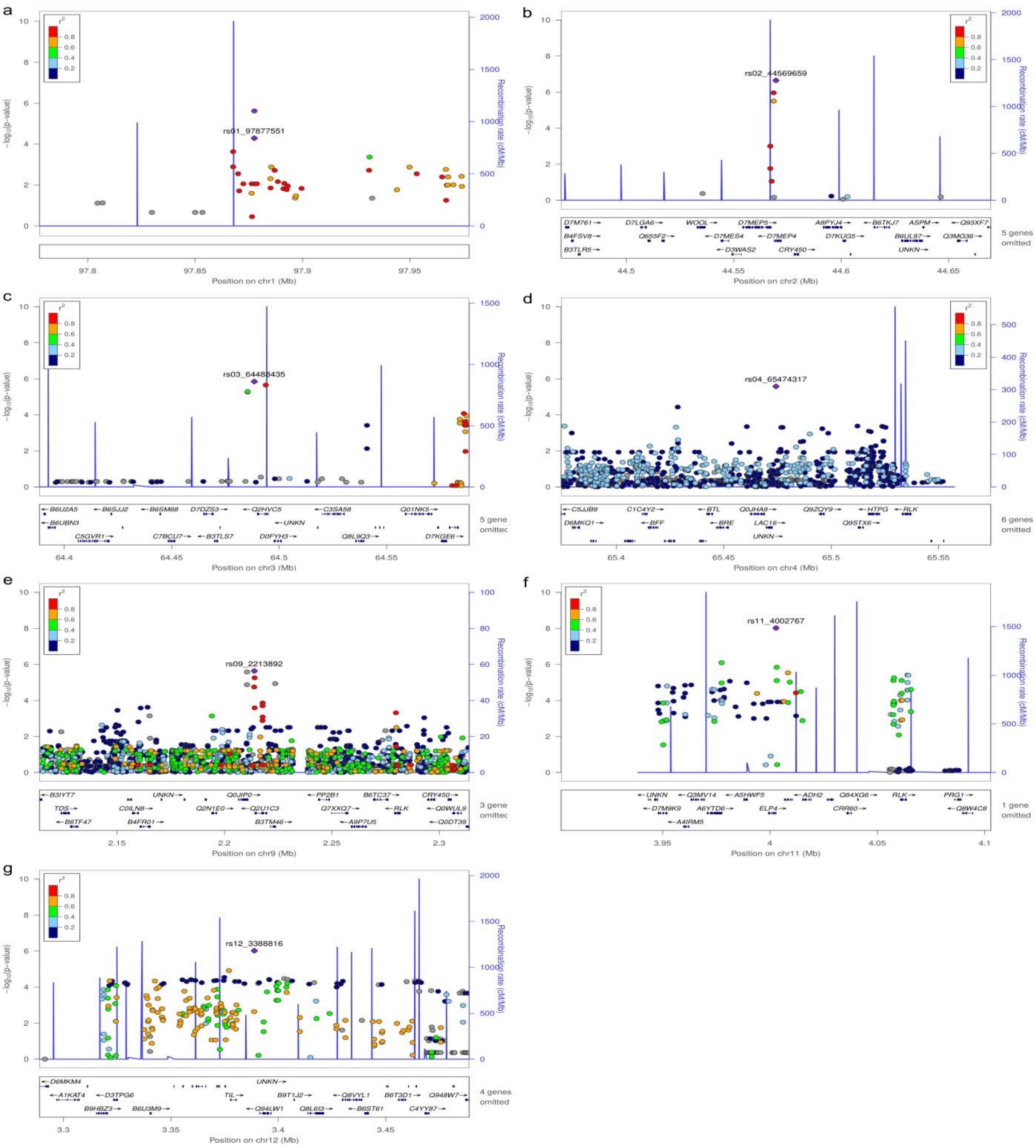

Supplementary Figure 92. LocusZoom plots of the newly identified significant associated loci in the meta-analysis of genome-wide associations of phenylalanine. Associations are expressed as –$\log_{10}$(P value). The top SNPs are shown as purple squares within the window of 100 kb. Color legend represents the linkage disequilibrium between the top SNPs and SNPs appeared in the 1 Mb window size and was calculated based on the 221 reference panel. If the peak SNP was not in the reference panel, the genotype from panel T was used for the LD calculation. The blue line represents the recombination rate from the inferred recombination maps. **a-g** stands for different LocusZoom plot for each significant association.

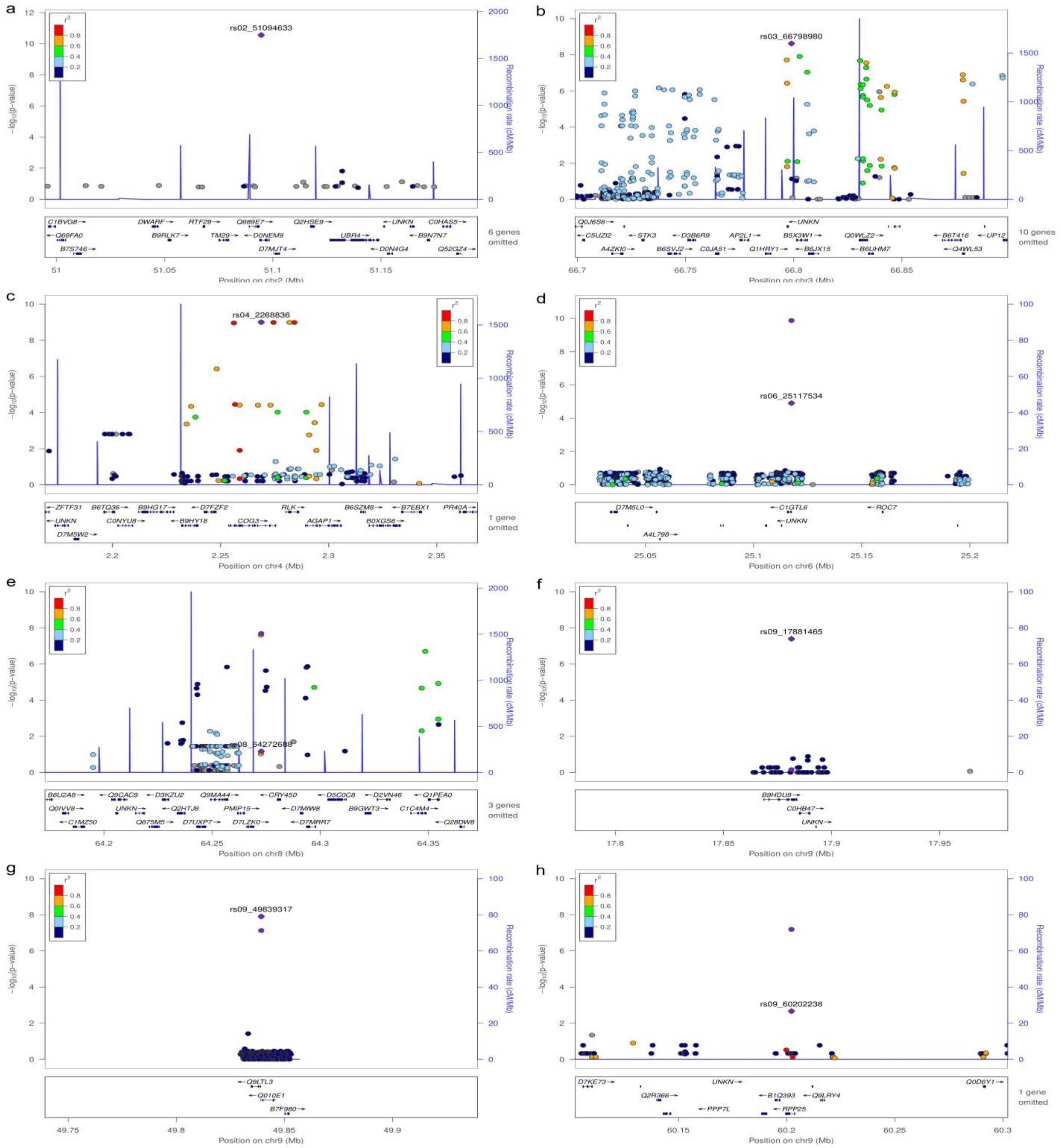

Supplementary Figure 93. LocusZoom plots of the previously identified significant associated loci in the meta-analysis of genome-wide associations of proline. Associations are expressed as –log$_{10}$(P value). The top SNPs are shown as purple squares within the window of 100 kb. Color legend represents the linkage disequilibrium between the top SNPs and SNPs appeared in the 1 Mb window size and was calculated based on the 221 reference panel. If the peak SNP was not in the reference panel, the genotype from panel T was used for the LD calculation. The blue line represents the recombination rate from the inferred recombination maps. **a-h** stands for different LocusZoom plot for each significant association.

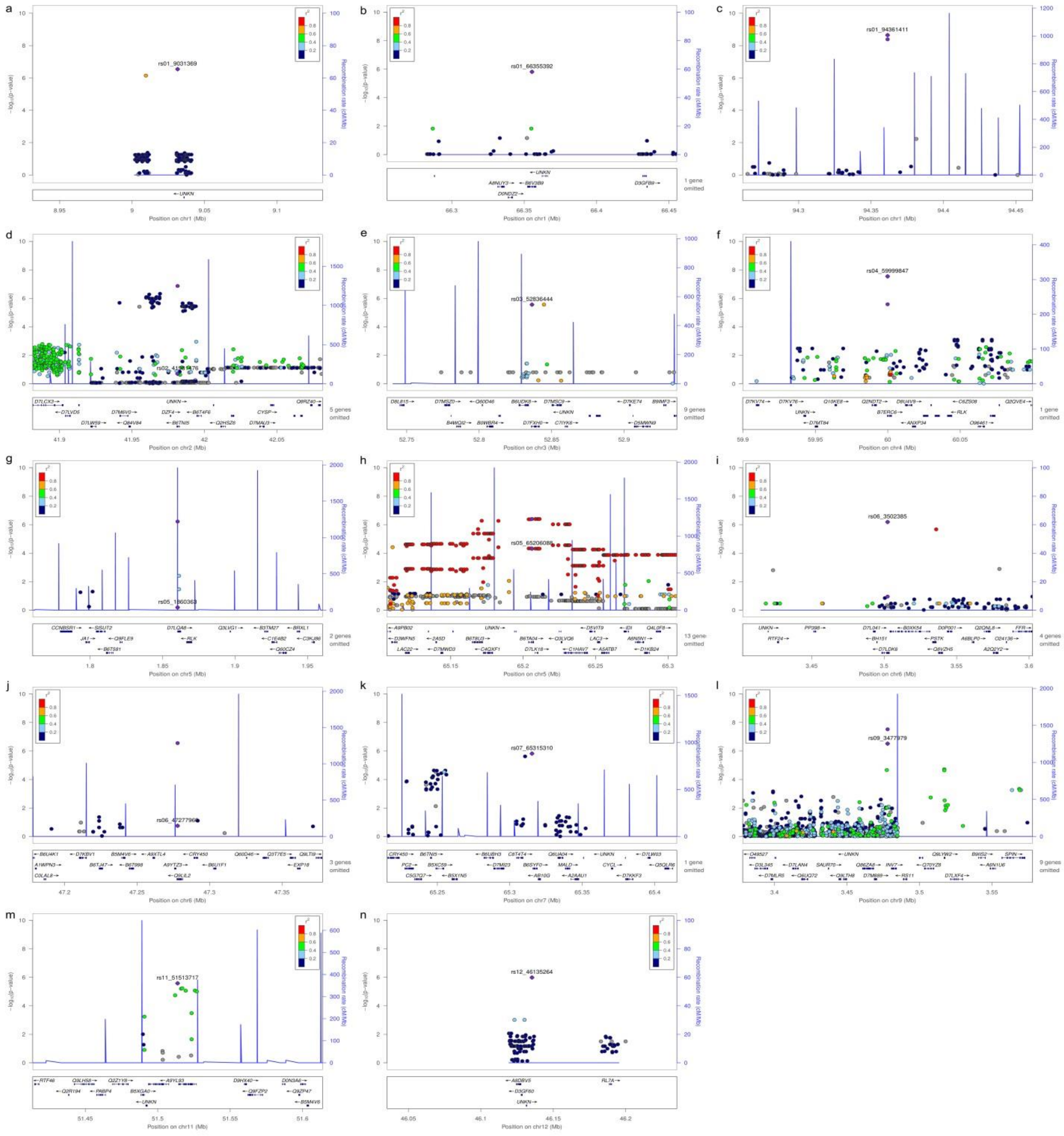

Supplementary Figure 94. LocusZoom plots of the newly identified significant associated loci in the meta-analysis of genome-wide associations of proline. Associations are expressed as –$\log_{10}$(P value). The top SNPs are shown as purple squares within the window of 100 kb. Color legend represents the linkage disequilibrium between the top SNPs and SNPs appeared in the 1 Mb window size and was calculated based on the 221 reference panel. If the peak SNP was not in the reference panel, the genotype from panel T was used for the LD calculation. The blue line represents the recombination rate from the inferred recombination maps. **a-n** stands for different LocusZoom plot for each significant association.

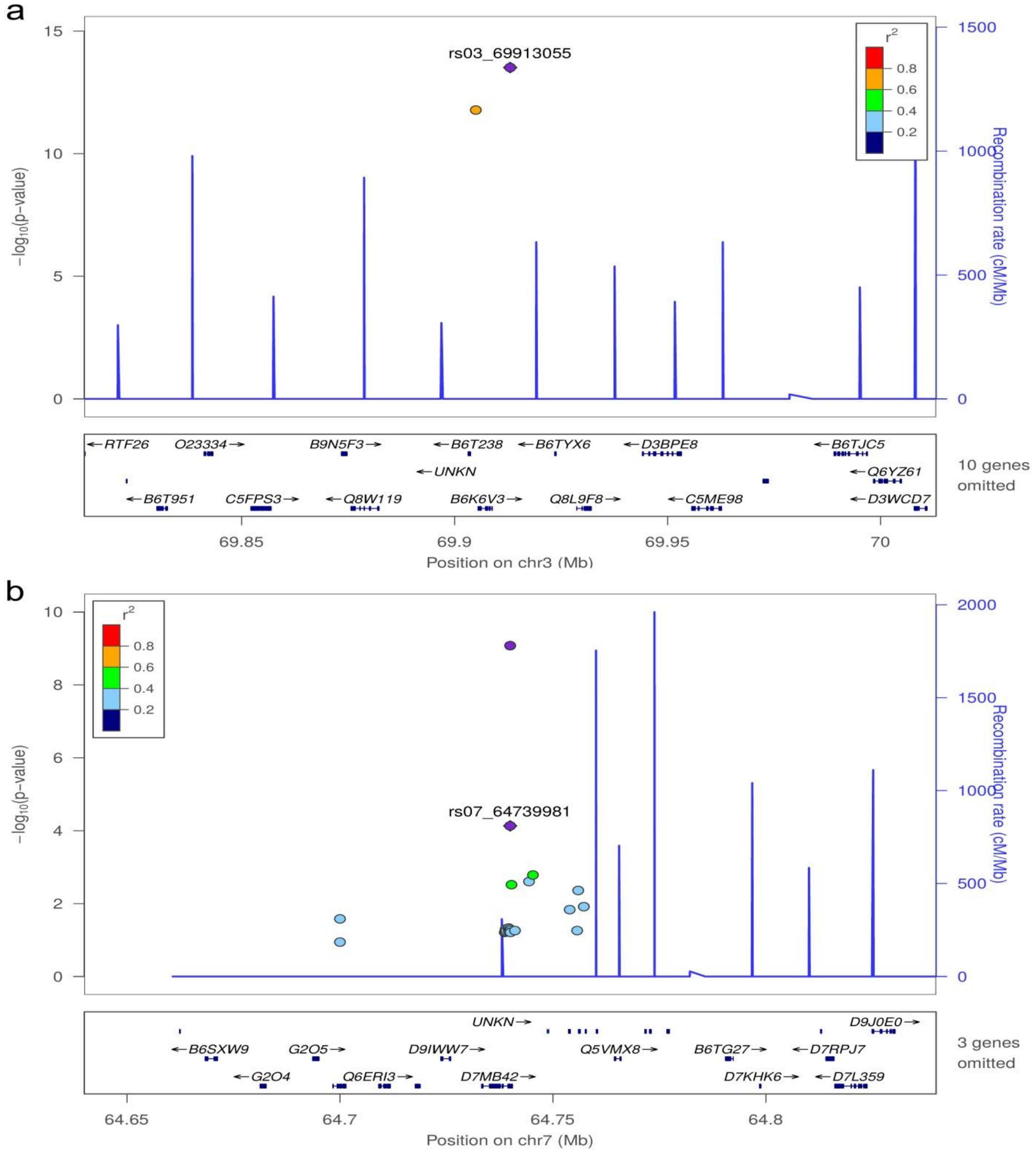

Supplementary Figure 95. LocusZoom plots of the previously identified significant associated loci in the meta-analysis of genome-wide associations of serine. Associations are expressed as –$\log_{10}$(*P* value). The top SNPs are shown as purple squares within the window of 100 kb. Color legend represents the linkage disequilibrium between the top SNPs and SNPs appeared in the 1 Mb window size and was calculated based on the 221 reference panel. If the peak SNP was not in the reference panel, the genotype from panel T was used for the LD calculation. The blue line represents the recombination rate from the inferred recombination maps. **a-b** stands for different LocusZoom plot for each significant association.

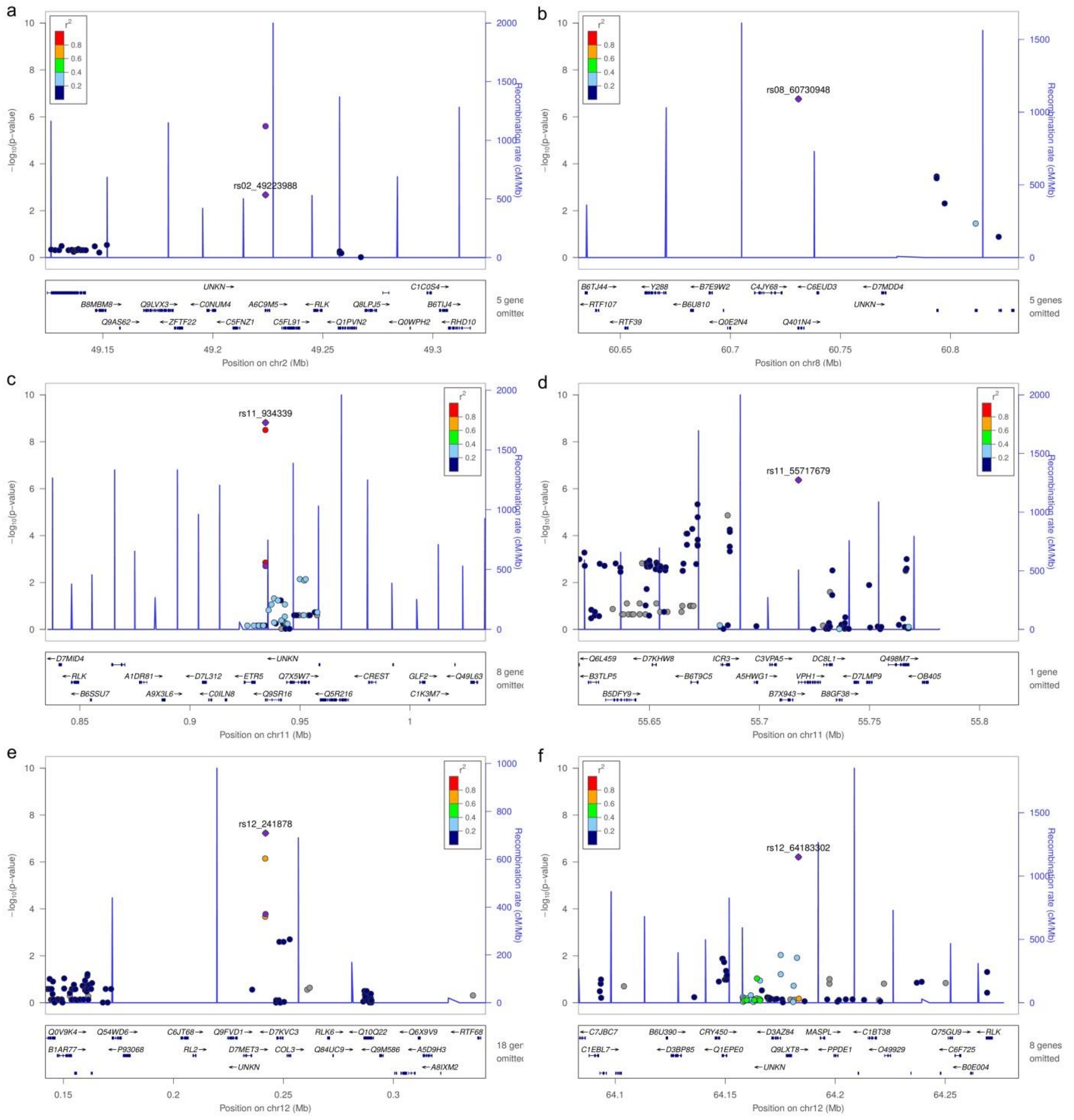

Supplementary Figure 96. LocusZoom plots of the newly identified significant associated loci in the meta-analysis of genome-wide associations of serine. Associations are expressed as $-\log_{10}$(P value). The top SNPs are shown as purple squares within the window of 100 kb. Color legend represents the linkage disequilibrium between the top SNPs and SNPs appeared in the 1 Mb window size and was calculated based on the 221 reference panel. If the peak SNP was not in the reference panel, the genotype from panel T was used for the LD calculation. The blue line represents the recombination rate from the inferred recombination maps. **a-f** stands for different LocusZoom plot for each significant association.

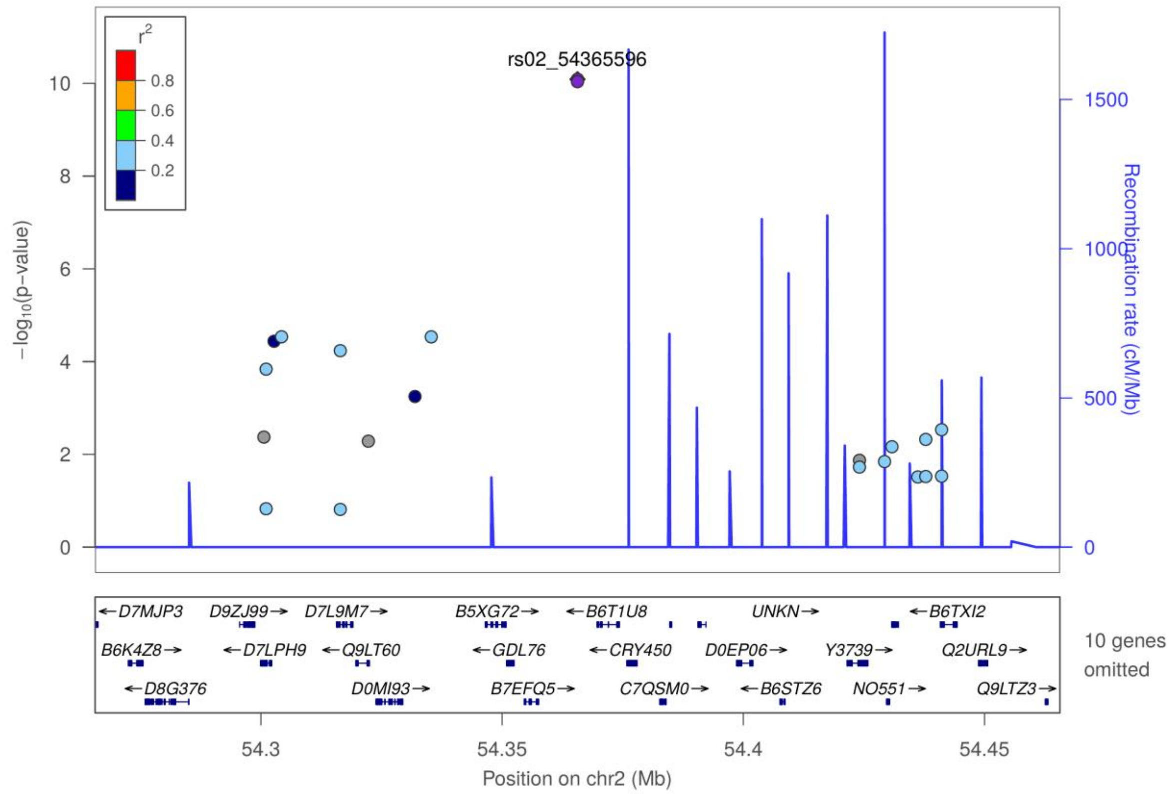

Supplementary Figure 97. LocusZoom plots of the previously identified significant associated loci in the meta-analysis of genome-wide associations of threonine. Associations are expressed as $-\log_{10}(P \text{ value})$. The top SNPs are shown as purple squares within the window of 100 kb. Color legend represents the linkage disequilibrium between the top SNPs and SNPs appeared in the 1 Mb window size and was calculated based on the 221 reference panel. If the peak SNP was not in the reference panel, the genotype from panel T was used for the LD calculation. The blue line represents the recombination rate from the inferred recombination maps.

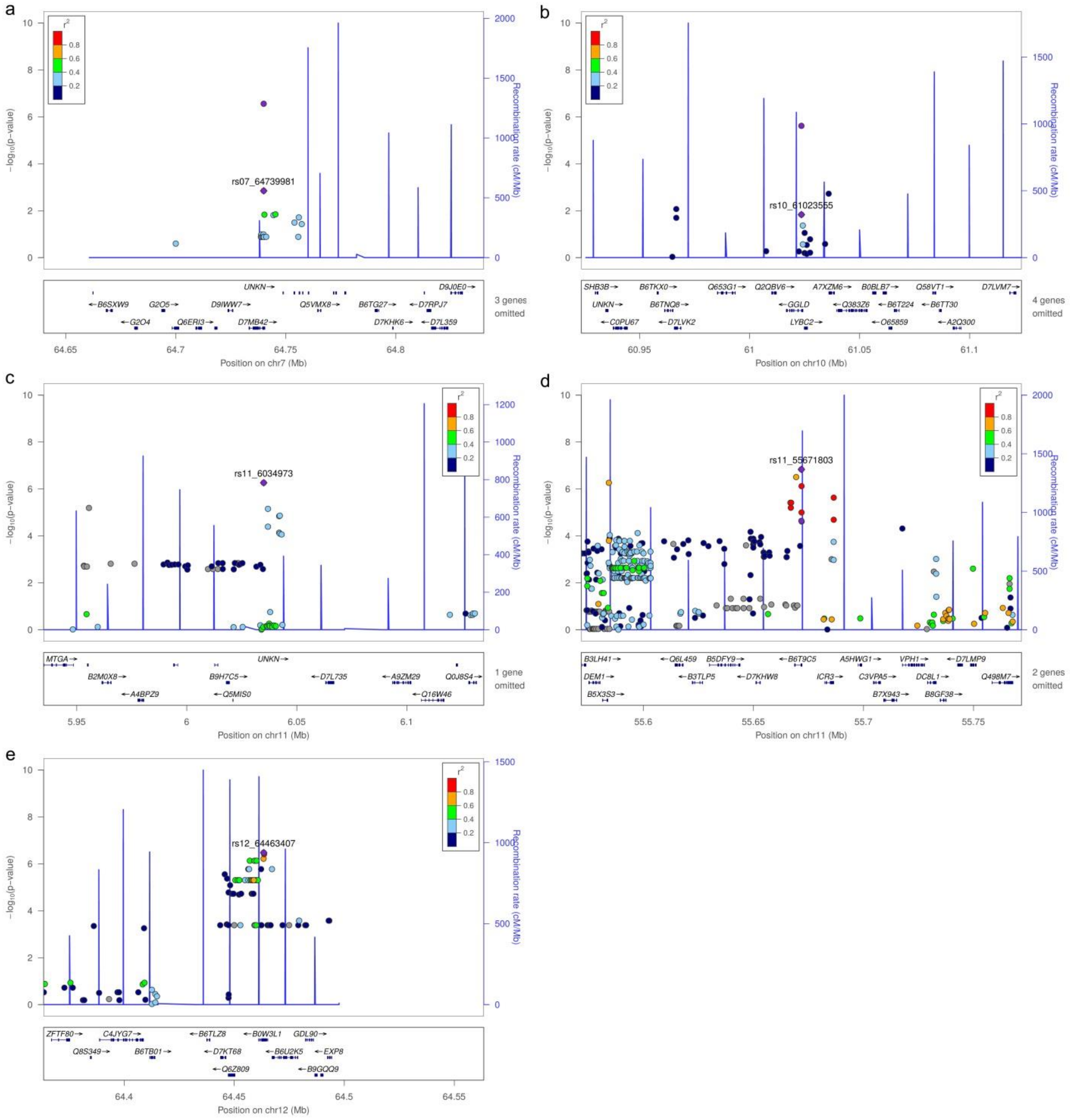

Supplementary Figure 98. LocusZoom plots of the newly identified significant associated loci in the meta-analysis of genome-wide associations of threonine. Associations are expressed as –$\log_{10}$(P value). The top SNPs are shown as purple squares within the window of 100 kb. Color legend represents the linkage disequilibrium between the top SNPs and SNPs appeared in the 1 Mb window size and was calculated based on the 221 reference panel. If the peak SNP was not in the reference panel, the genotype from panel T was used for the LD calculation. The blue line represents the recombination rate from the inferred recombination maps. **a-e** stands for different LocusZoom plot for each significant association.

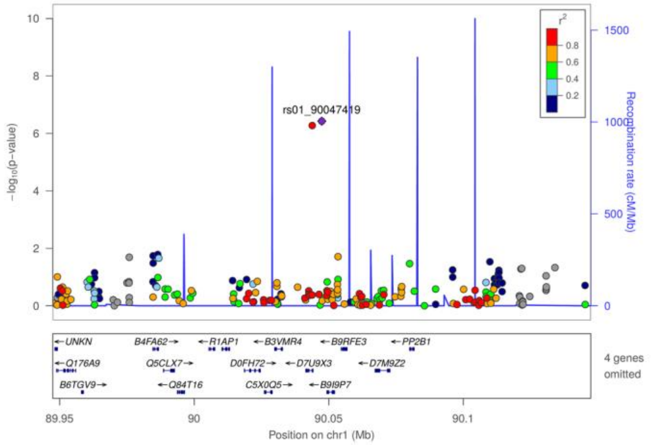

Supplementary Figure 99. LocusZoom plots of the previously identified significant associated loci in the meta-analysis of genome-wide associations of beta-ionone. Associations are expressed as –$\log_{10}$(P value). The top SNPs are shown as purple squares within the window of 100 kb. Color legend represents the linkage disequilibrium between the top SNPs and SNPs appeared in the 1 Mb window size and was calculated based on the 221 reference panel. If the peak SNP was not in the reference panel, the genotype from panel T was used for the LD calculation. The blue line represents the recombination rate from the inferred recombination maps.

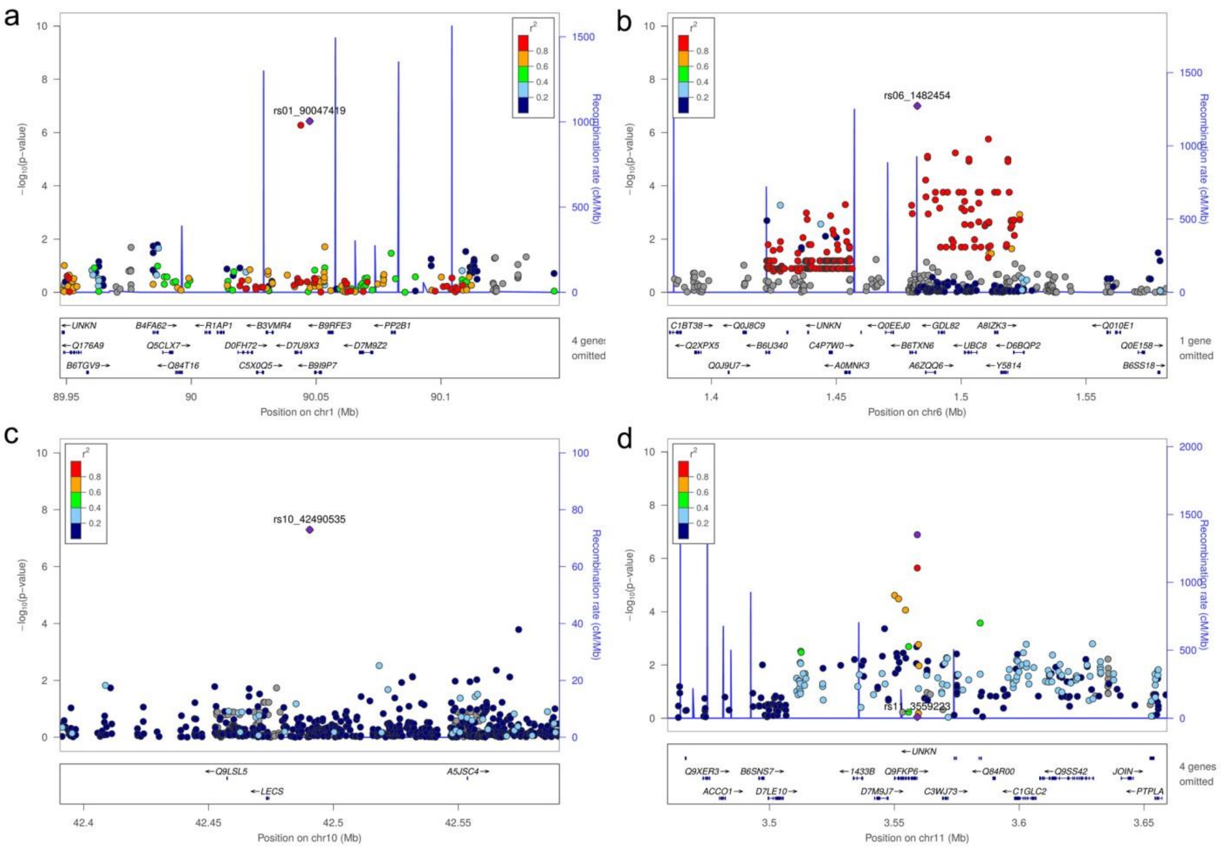

Supplementary Figure 100. LocusZoom plots of the newly identified significant associated loci in the meta-analysis of genome-wide associations of beta-ionone. Associations are expressed as –log$_{10}$(P value). The top SNPs are shown as purple squares within the window of 100 kb. Color legend represents the linkage disequilibrium between the top SNPs and SNPs appeared in the 1 Mb window size and was calculated based on the 221 reference panel. If the peak SNP was not in the reference panel, the genotype from panel T was used for the LD calculation. The blue line represents the recombination rate from the inferred recombination maps. **a-d** stands for different LocusZoom plot for each significant association.

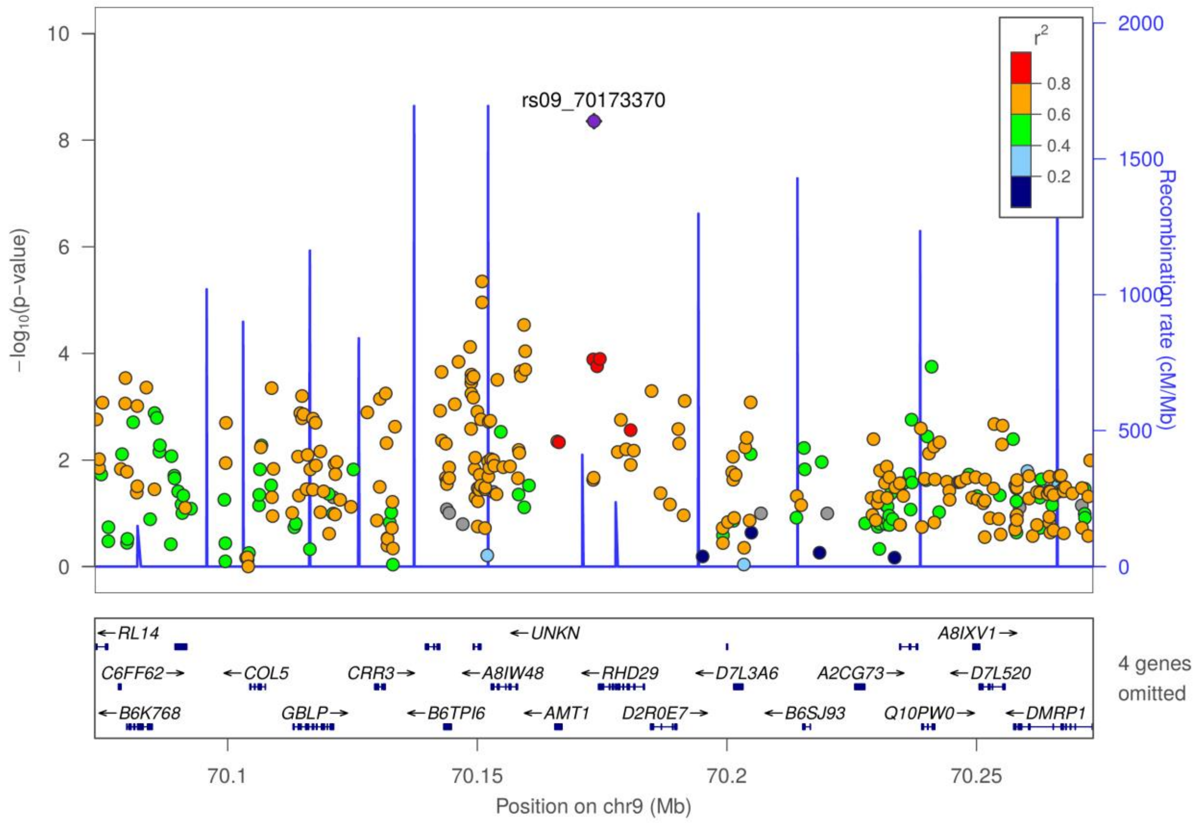

Supplementary Figure 101. LocusZoom plots of the previously identified significant associated loci in the meta-analysis of genome-wide associations of (E)-2-pentenal. Associations are expressed as $–\log_{10}$(P value). The top SNPs are shown as purple squares within the window of 100 kb. Color legend represents the linkage disequilibrium between the top SNPs and SNPs appeared in the 1 Mb window size and was calculated based on the 221 reference panel. If the peak SNP was not in the reference panel, the genotype from panel T was used for the LD calculation. The blue line represents the recombination rate from the inferred recombination maps.

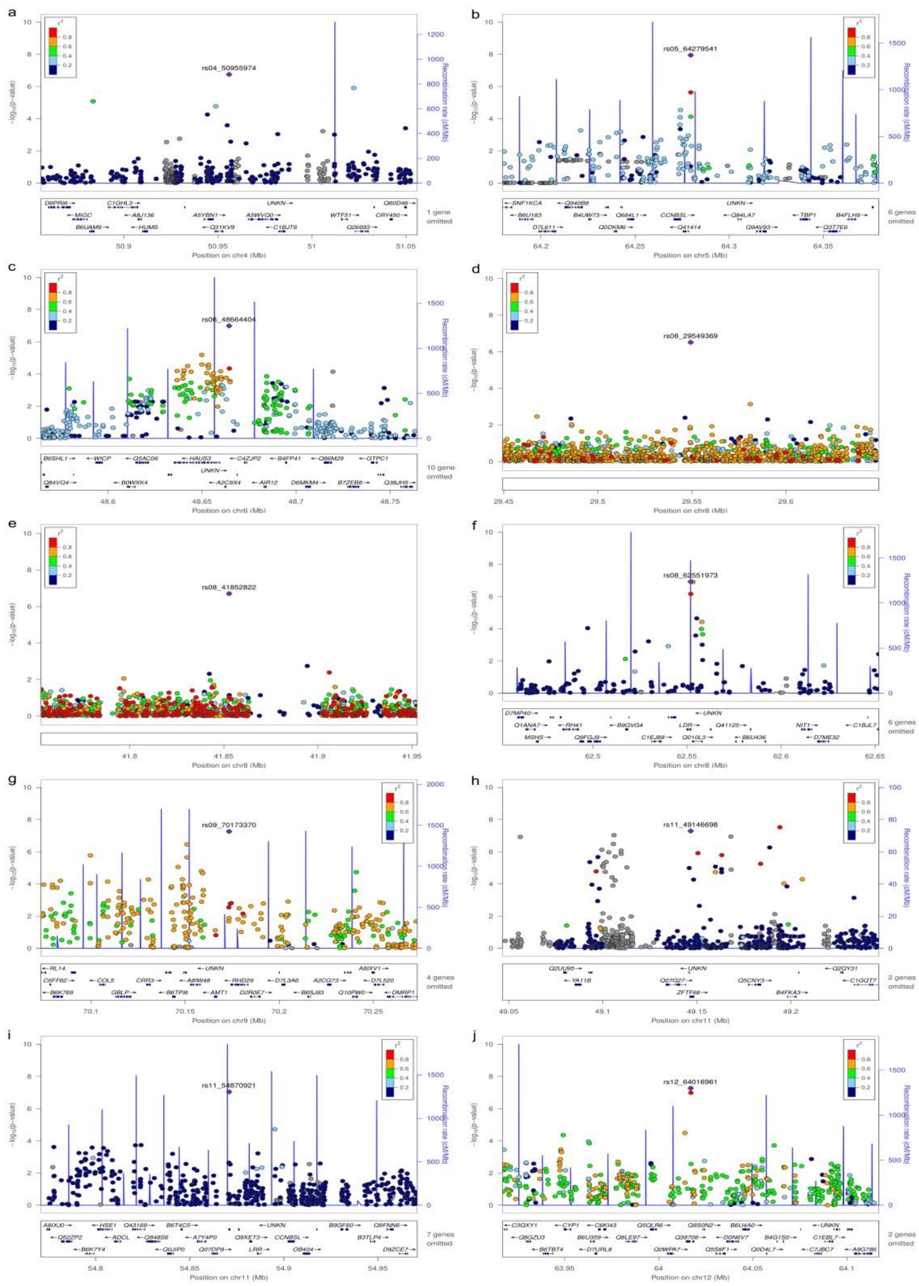

Supplementary Figure 102. LocusZoom plots of the newly identified significant associated loci in the meta-analysis of genome-wide associations of (E)-2-hexenal. Associations are expressed as $-\log_{10}(P$ value). The top SNPs are shown as purple squares within the window of 100 kb. Color legend represents the linkage disequilibrium between the top SNPs and SNPs appeared in the 1 Mb window size and was calculated based on the 221 reference panel. If the peak SNP was not in the reference panel, the genotype from panel T was used for the LD calculation. The blue line represents the recombination rate from the inferred recombination maps. **a-j** stands for different LocusZoom plot for each significant association.

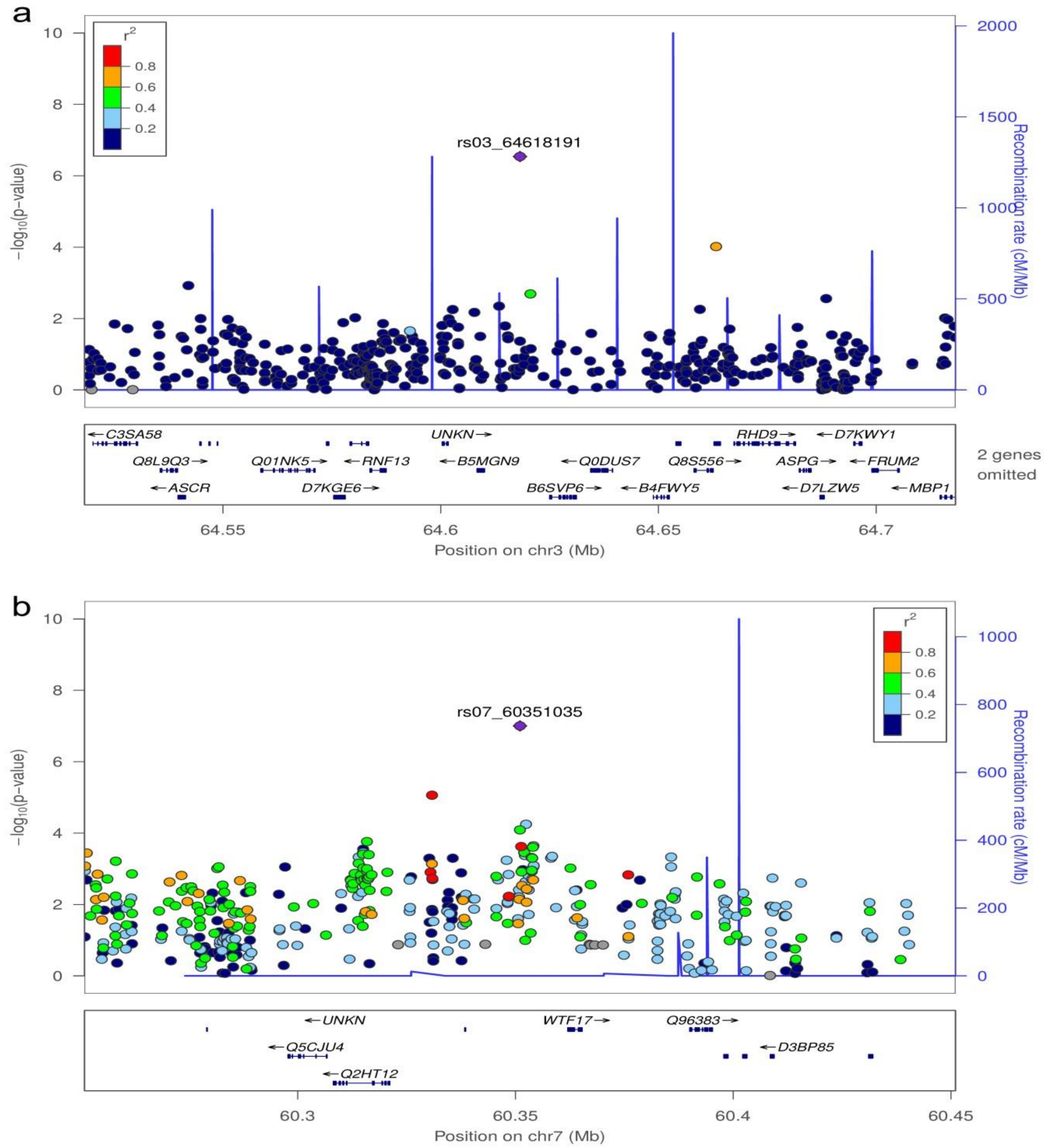

Supplementary Figure 103. LocusZoom plots of the newly identified significant associated loci in the meta-analysis of genome-wide associations of (E,E)-2,4-decadienal. Associations are expressed as –log$_{10}$(P value). The top SNPs are shown as purple squares within the window of 100 kb. Color legend represents the linkage disequilibrium between the top SNPs and SNPs appeared in the 1 Mb window size and was calculated based on the 221 reference panel. If the peak SNP was not in the reference panel, the genotype from panel T was used for the LD calculation. The blue line represents the recombination rate from the inferred recombination maps. **a-b** stands for different LocusZoom plot for each significant association.

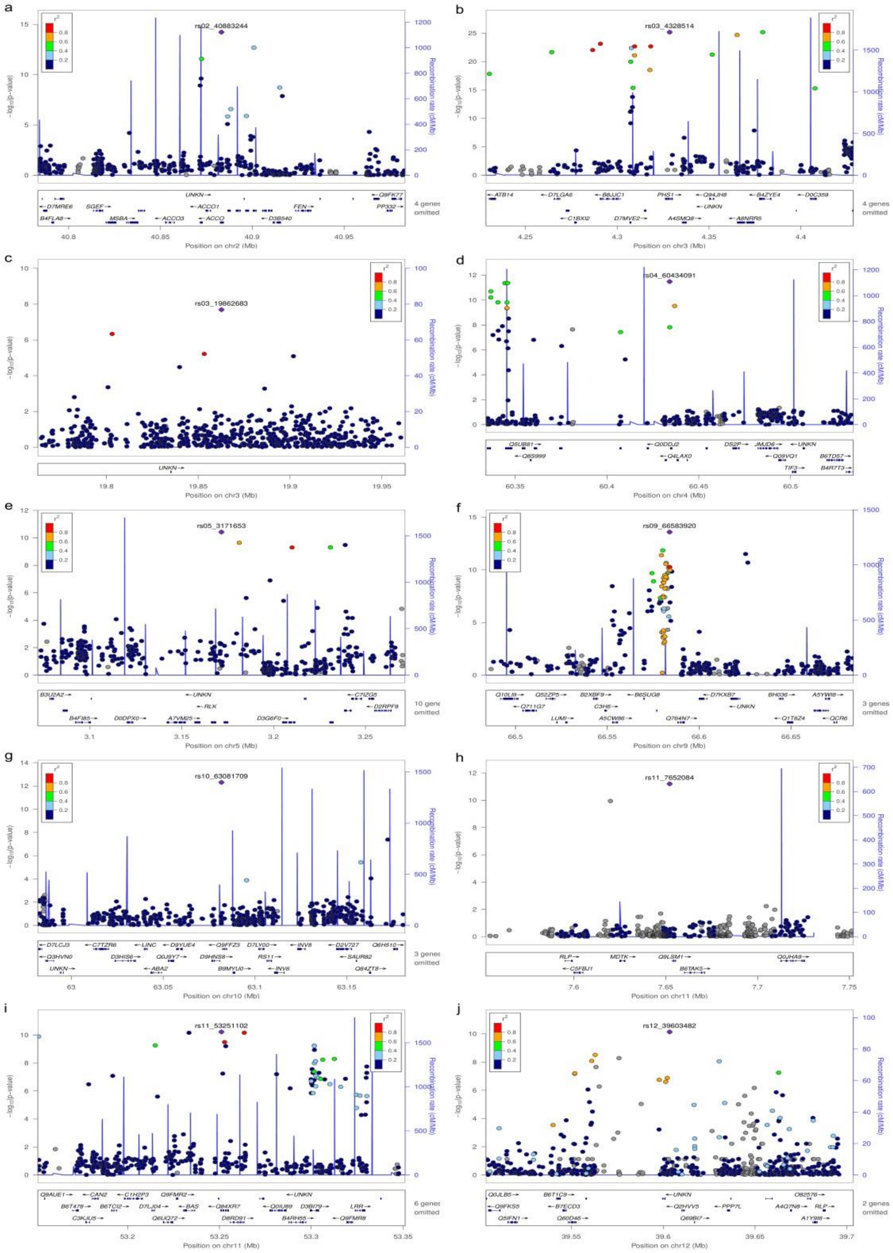

Supplementary Figure 104. LocusZoom plots of the previously identified significant associated loci in the meta-analysis of genome-wide associations of geranyl acetone. Associations are expressed as $-\log_{10}$(P value). The top SNPs are shown as purple squares within the window of 100 kb. Color legend represents the linkage disequilibrium between the top SNPs and SNPs appeared in the 1 Mb window size and was calculated based on the 221 reference panel. If the peak SNP was not in the reference panel, the genotype from panel T was used for the LD calculation. The blue line represents the recombination rate from the inferred recombination maps. **a-j** stands for different LocusZoom plot for each significant association.

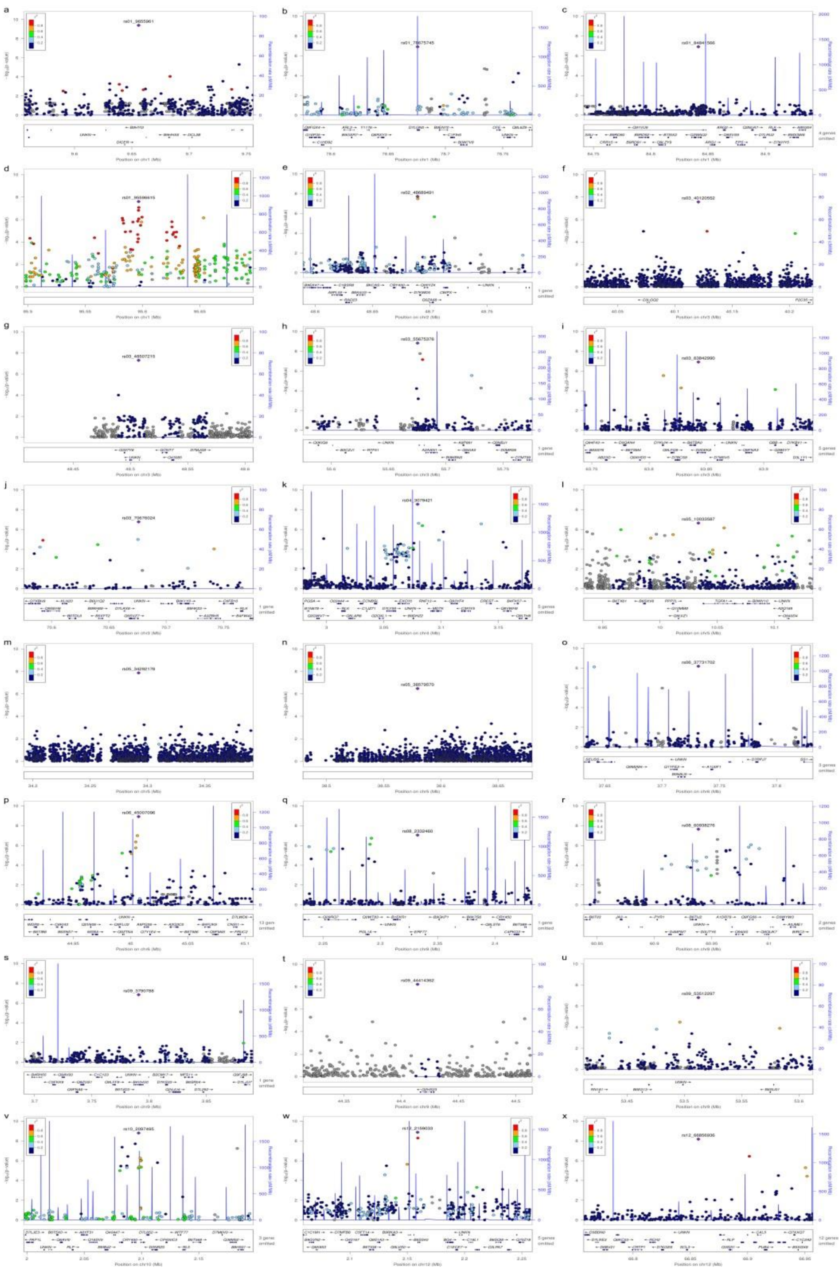

Supplementary Figure 105. LocusZoom plots of the newly identified significant associated loci in the meta-analysis of genome-wide associations of geranyl acetone. Associations are expressed as –log$_{10}$(P value). The top SNPs are shown as purple squares within the window of 100 kb. Color legend represents the linkage disequilibrium between the top SNPs and SNPs appeared in the 1 Mb window size and was calculated based on the 221 reference panel. If the peak SNP was not in the reference panel, the genotype from panel T was used for the LD calculation. The blue line represents the recombination rate from the inferred recombination maps. **a-x** stands for different LocusZoom plot for each significant association.

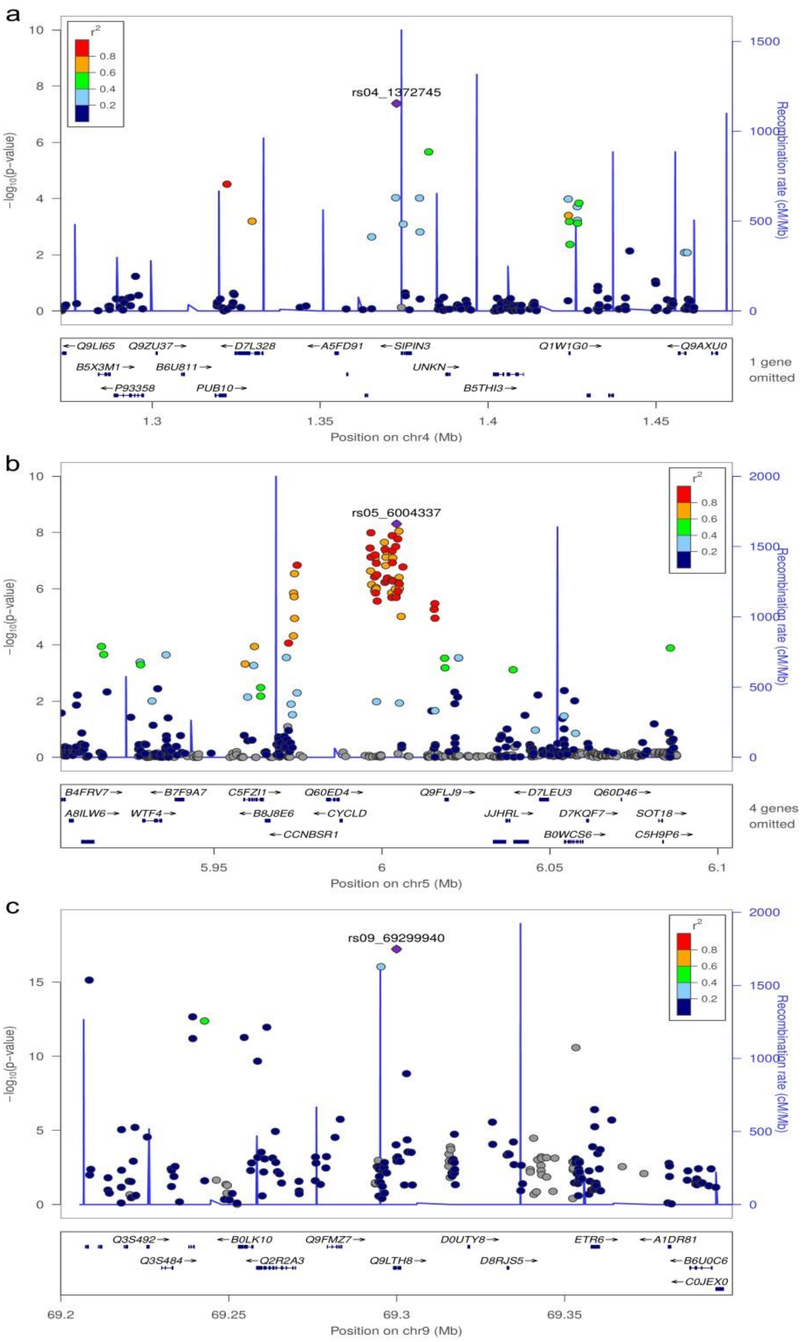

Supplementary Figure 106. LocusZoom plots of the previously identified significant associated loci in the meta-analysis of genome-wide associations of guaiacol. Associations are expressed as $–\log_{10}(P \text{ value})$. The top SNPs are shown as purple squares within the window of 100 kb. Color legend represents the linkage disequilibrium between the top SNPs and SNPs appeared in the 1 Mb window size and was calculated based on the 221 reference panel. If the peak SNP was not in the reference panel, the genotype from panel T was used for the LD calculation. The blue line represents the recombination rate from the inferred recombination maps. **a-c** stands for different LocusZoom plot for each significant association.

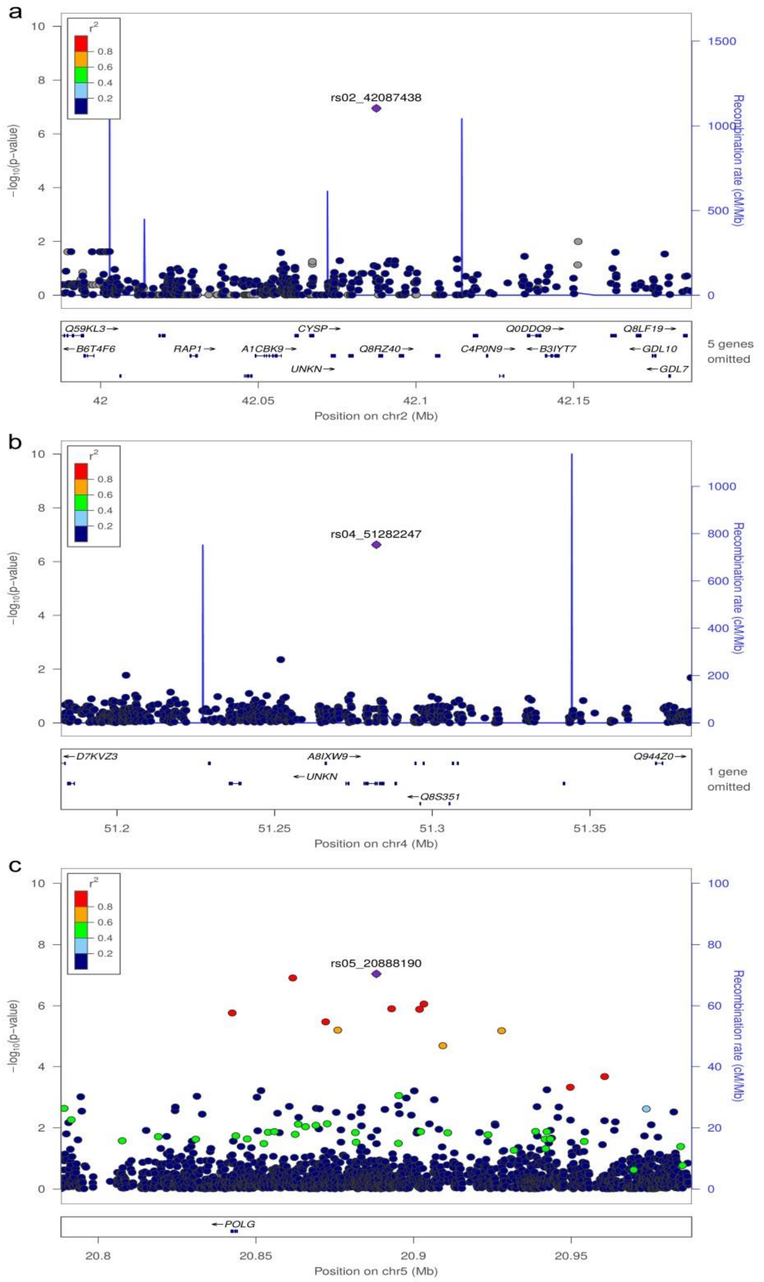

Supplementary Figure 107. LocusZoom plots of the newly identified significant associated loci in the meta-analysis of genome-wide associations of guaiacol. Associations are expressed as –log$_{10}$(P value). The top SNPs are shown as purple squares within the window of 100 kb. Color legend represents the linkage disequilibrium between the top SNPs and SNPs appeared in the 1 Mb window size and was calculated based on the 221 reference panel. If the peak SNP was not in the reference panel, the genotype from panel T was used for the LD calculation. The blue line represents the recombination rate from the inferred recombination maps. **a-c** stands for different LocusZoom plot for each significant association.

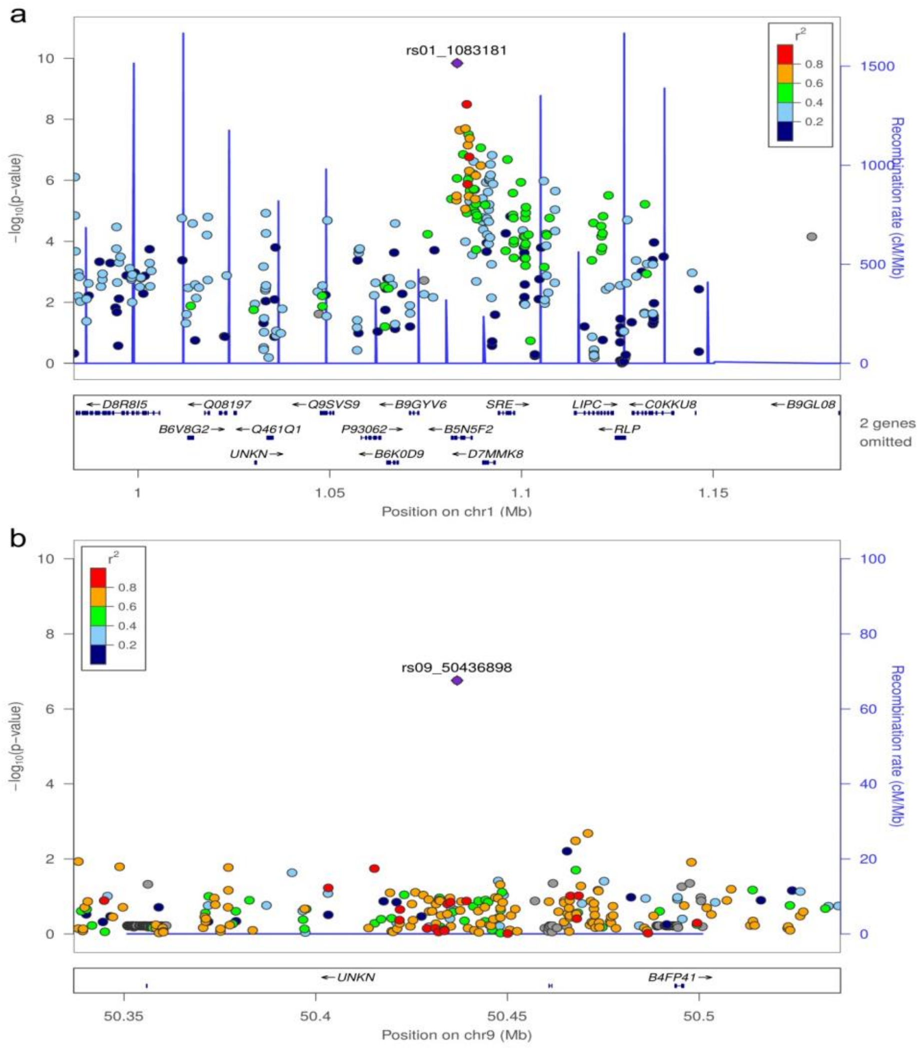

Supplementary Figure 108. LocusZoom plots of the previously identified significant associated loci in the meta-analysis of genome-wide associations of hexanal. Associations are expressed as $-\log_{10}$(P value). The top SNPs are shown as purple squares within the window of 100 kb. Color legend represents the linkage disequilibrium between the top SNPs and SNPs appeared in the 1 Mb window size and was calculated based on the 221 reference panel. If the peak SNP was not in the reference panel, the genotype from panel T was used for the LD calculation. The blue line represents the recombination rate from the inferred recombination maps. **a-b** stands for different LocusZoom plot for each significant association.

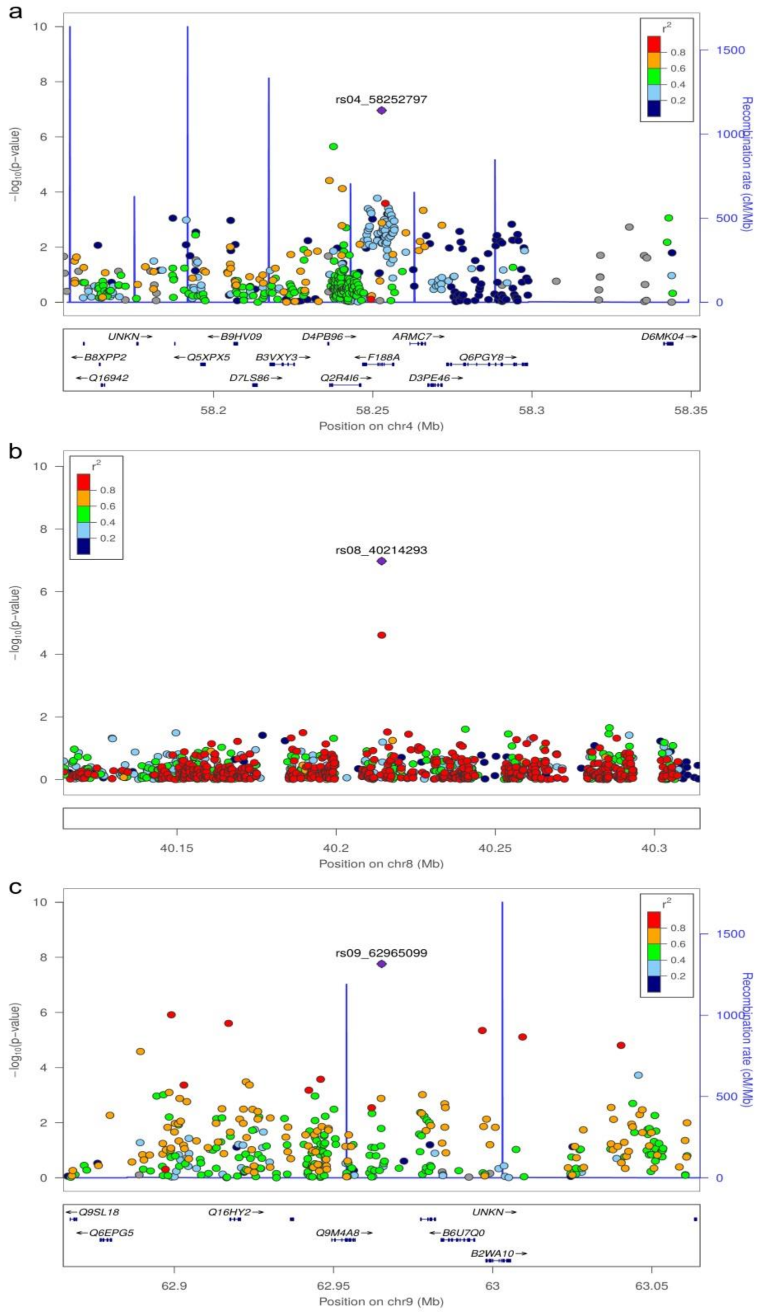

Supplementary Figure 109. LocusZoom plots of the newly identified significant associated loci in the meta-analysis of genome-wide associations of hexanal. Associations are expressed as $-\log_{10}$(*P* value). The top SNPs are shown as purple squares within the window of 100 kb. Color legend represents the linkage disequilibrium between the top SNPs and SNPs appeared in the 1 Mb window size and was calculated based on the 221 reference panel. If the peak SNP was not in the reference panel, the genotype from panel T was used for the LD calculation. The blue line represents the recombination rate from the inferred recombination maps. **a-c** stands for different LocusZoom plot for each significant association.

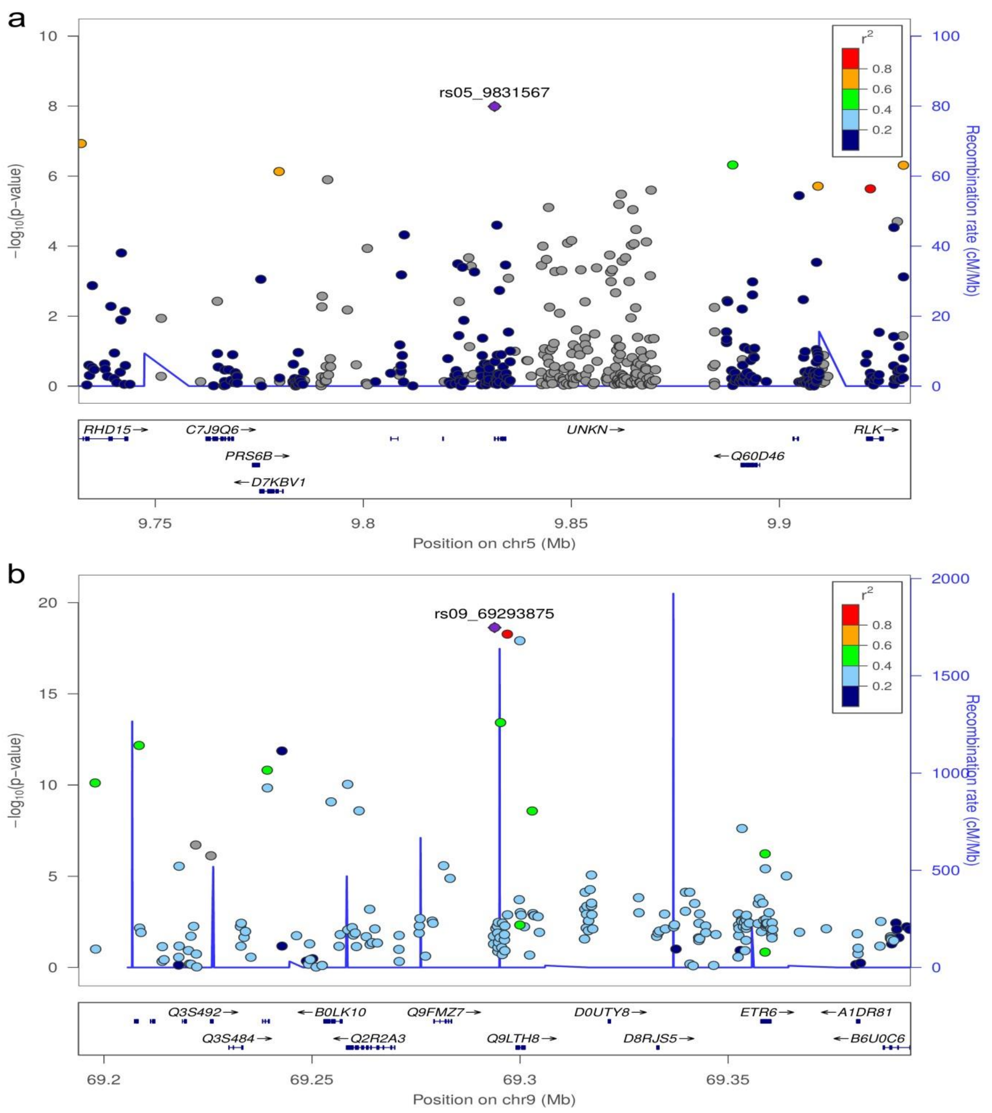

Supplementary Figure 110. LocusZoom plots of the previously identified significant associated loci in the meta-analysis of genome-wide associations of methyl salicylate. Associations are expressed as –$\log_{10}$(P value). The top SNPs are shown as purple squares within the window of 100 kb. Color legend represents the linkage disequilibrium between the top SNPs and SNPs appeared in the 1 Mb window size and was calculated based on the 221 reference panel. If the peak SNP was not in the reference panel, the genotype from panel T was used for the LD calculation. The blue line represents the recombination rate from the inferred recombination maps. **a-b** stands for different LocusZoom plot for each significant association.

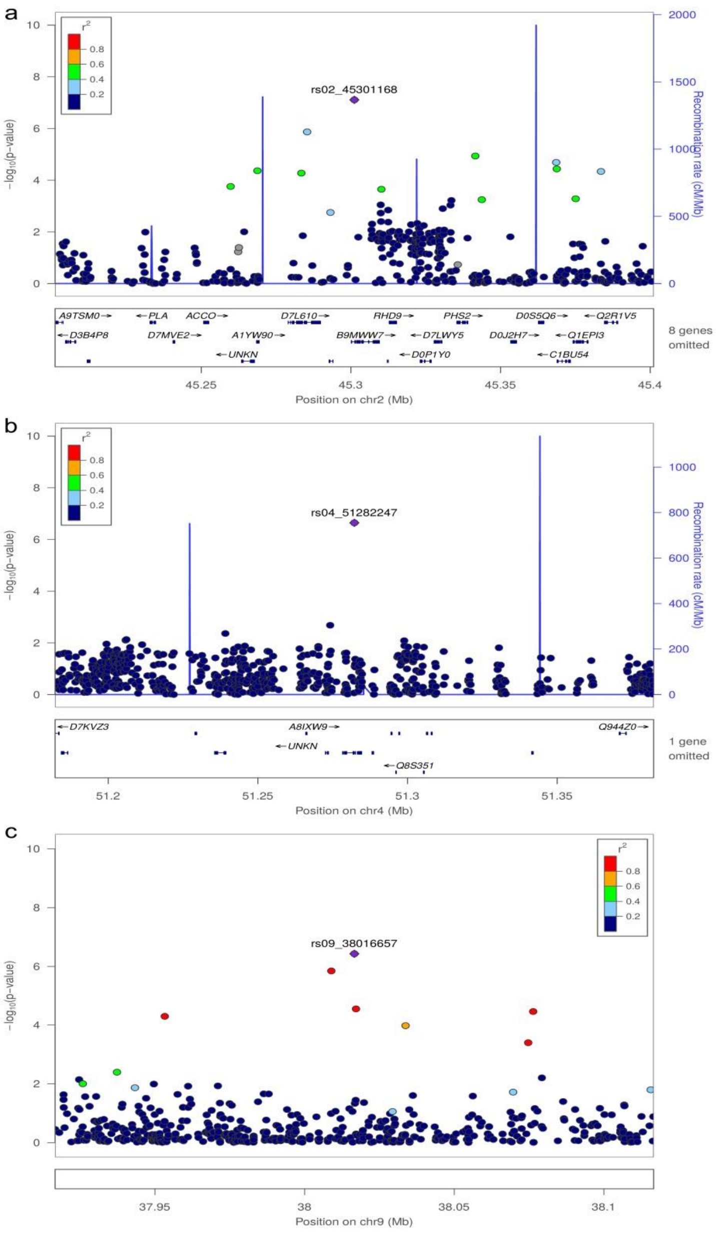

Supplementary Figure 111. LocusZoom plots of the newly identified significant associated loci in the meta-analysis of genome-wide associations of methyl salicylate. Associations are expressed as $–\log_{10}$(*P* value). The top SNPs are shown as purple squares within the window of 100 kb. Color legend represents the linkage disequilibrium between the top SNPs and SNPs appeared in the 1 Mb window size and was calculated based on the 221 reference panel. If the peak SNP was not in the reference panel, the genotype from panel T was used for the LD calculation. The blue line represents the recombination rate from the inferred recombination maps. **a-c** stands for different LocusZoom plot for each significant association.

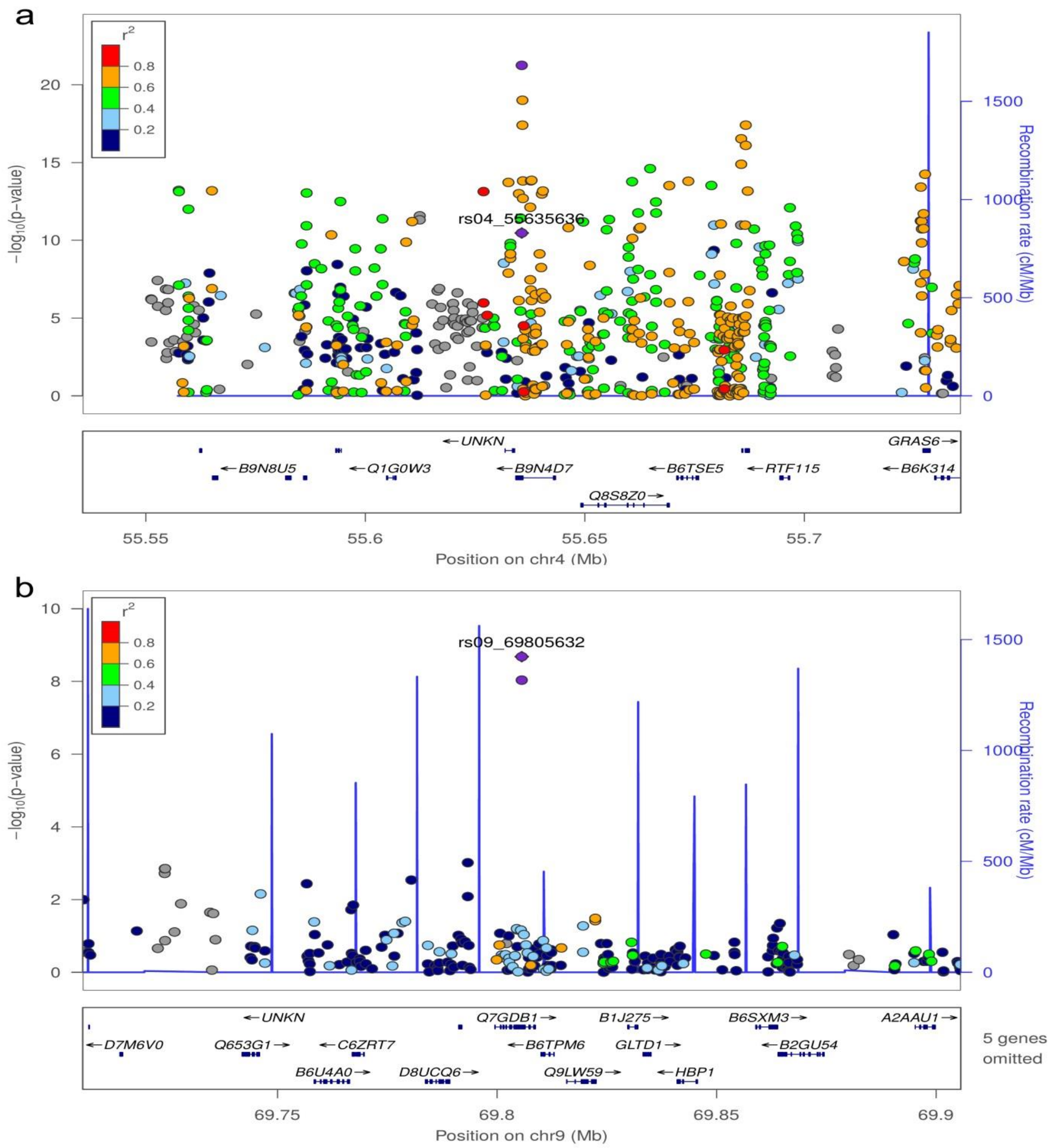

Supplementary Figure 112. LocusZoom plots of the previously identified significant associated loci in the meta-analysis of genome-wide associations of phenylacetaldehyde. Associations are expressed as –log$_{10}$(P value). The top SNPs are shown as purple squares within the window of 100 kb. Color legend represents the linkage disequilibrium between the top SNPs and SNPs appeared in the 1 Mb window size and was calculated based on the 221 reference panel. If the peak SNP was not in the reference panel, the genotype from panel T was used for the LD calculation. The blue line represents the recombination rate from the inferred recombination maps. **a-b** stands for different LocusZoom plot for each significant association.

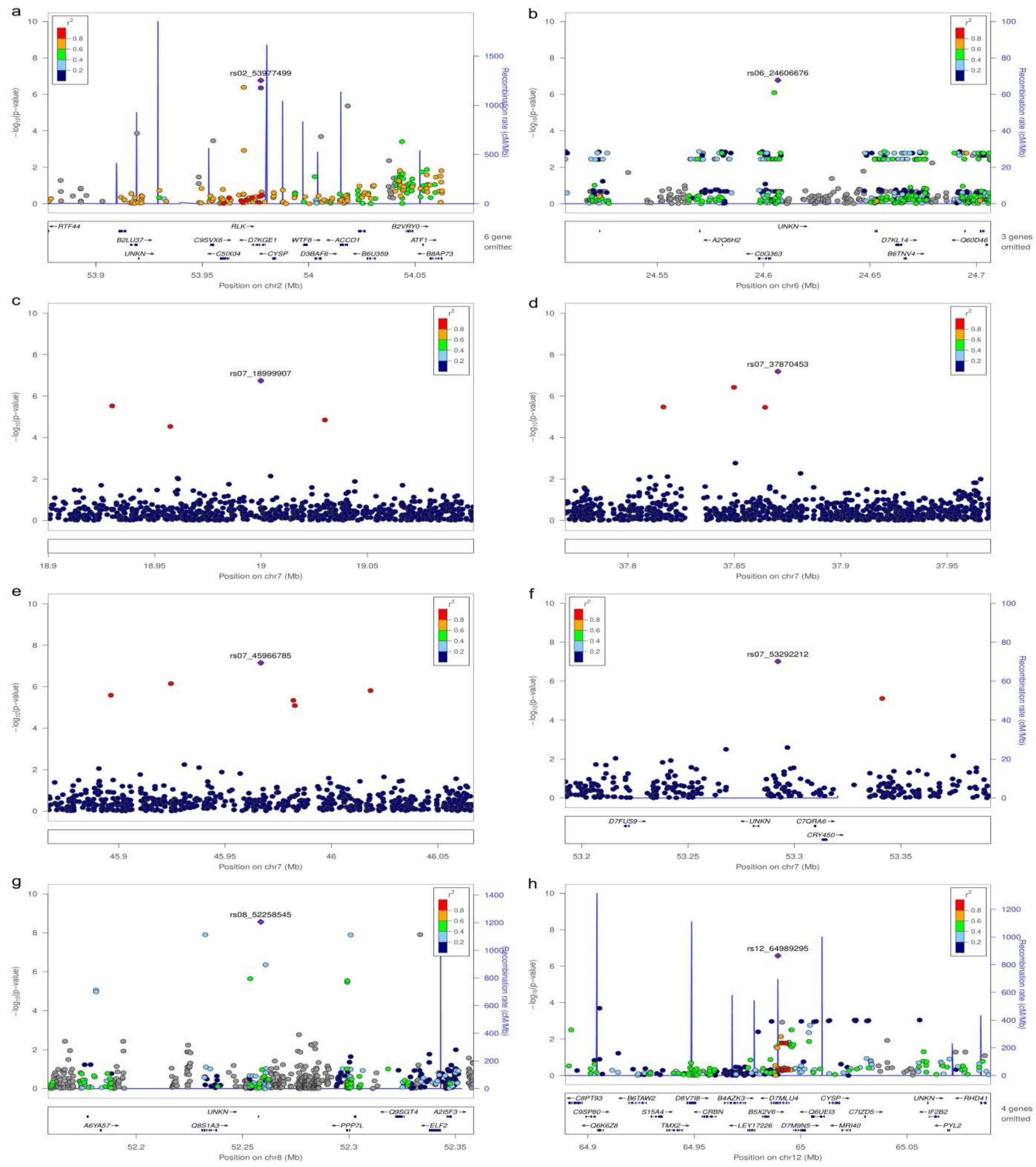

Supplementary Figure 113. LocusZoom plots of the newly identified significant associated loci in the meta-analysis of genome-wide associations of phenylacetalehyde. Associations are expressed as $–\log_{10}(P$ value). The top SNPs are shown as purple squares within the window of 100 kb. Color legend represents the linkage disequilibrium between the top SNPs and SNPs appeared in the 1 Mb window size and was calculated based on the 221 reference panel. If the peak SNP was not in the reference panel, the genotype from panel T was used for the LD calculation. The blue line represents the recombination rate from the inferred recombination maps. **a-h** stands for different LocusZoom plot for each significant association.

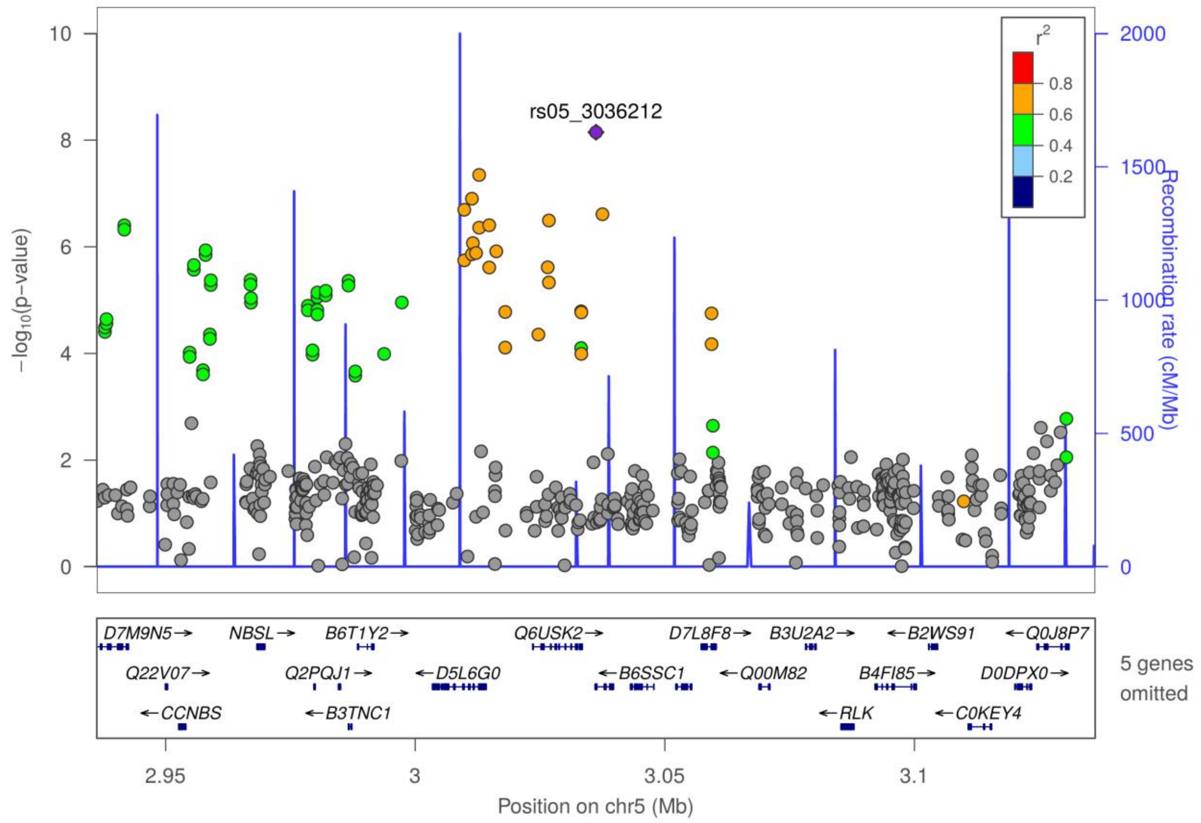

Supplementary Figure 114. LocusZoom plots of the previously identified significant associated loci in the meta-analysis of genome-wide associations of 1-penten-3-one. Associations are expressed as –$\log_{10}$(P value). The top SNPs are shown as purple squares within the window of 100 kb. Color legend represents the linkage disequilibrium between the top SNPs and SNPs appeared in the 1 Mb window size and was calculated based on the 221 reference panel. If the peak SNP was not in the reference panel, the genotype from panel T was used for the LD calculation. The blue line represents the recombination rate from the inferred recombination maps.

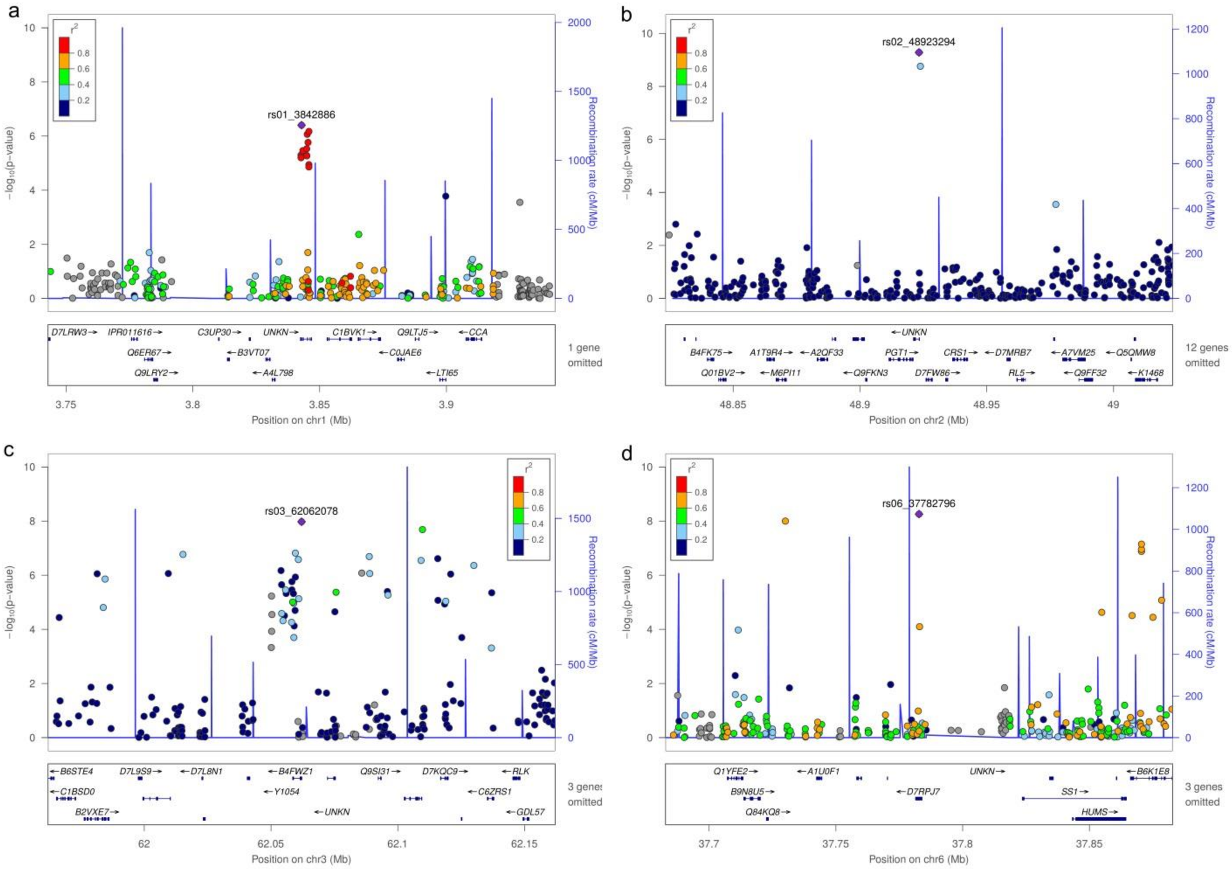

Supplementary Figure 115. LocusZoom plots of the previously identified significant associated loci in the meta-analysis of genome-wide associations of 2-methyl-1-butanol. Associations are expressed as –$\log_{10}$(P value). The top SNPs are shown as purple squares within the window of 100 kb. Color legend represents the linkage disequilibrium between the top SNPs and SNPs appeared in the 1 Mb window size and was calculated based on the 221 reference panel. If the peak SNP was not in the reference panel, the genotype from panel T was used for the LD calculation. The blue line represents the recombination rate from the inferred recombination maps. **a-d** stands for different LocusZoom plot for each significant association.

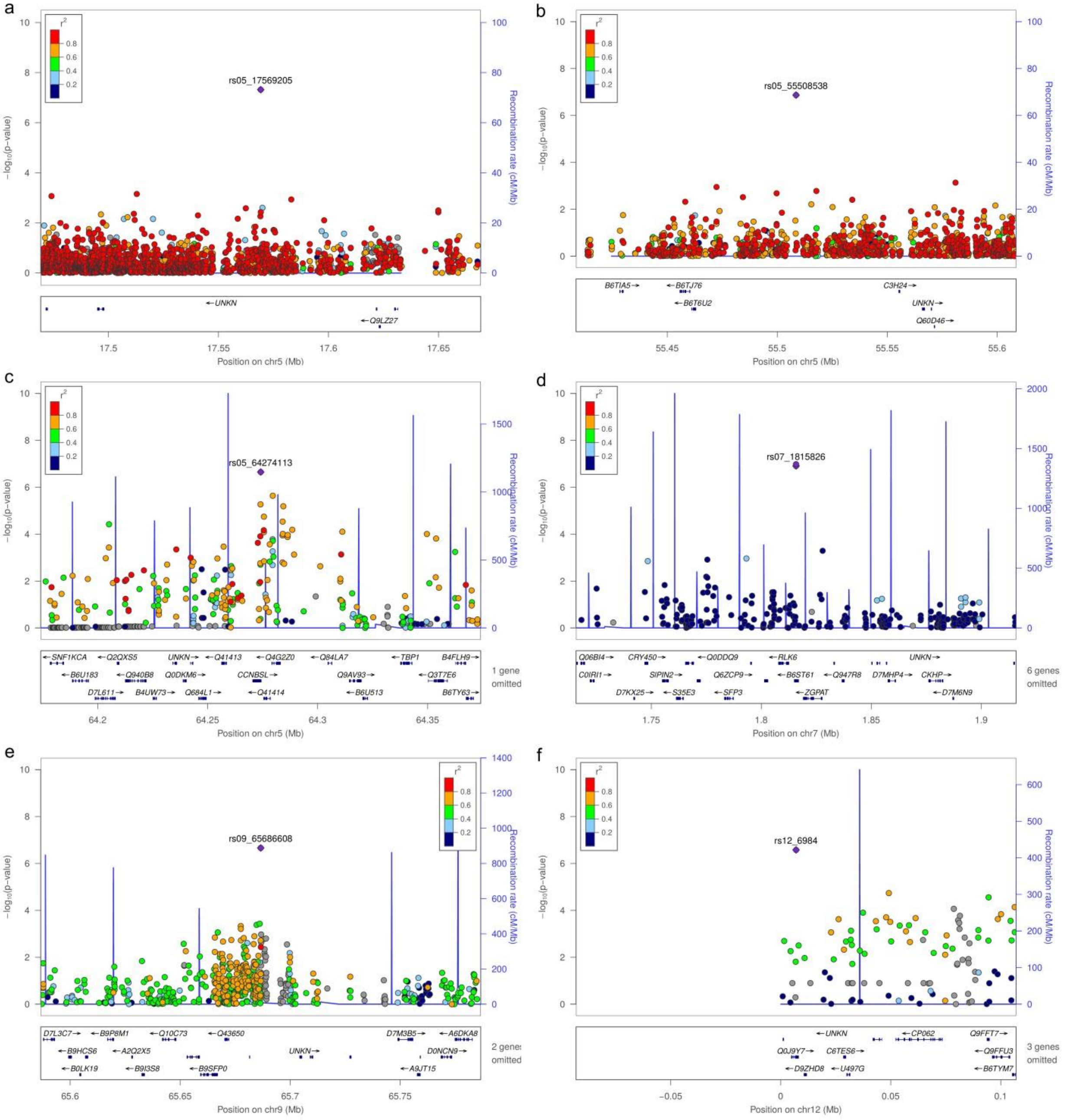

Supplementary Figure 116. LocusZoom plots of the newly identified significant associated loci in the meta-analysis of genome-wide associations of 2-methyl-1-butanol. Associations are expressed as –$\log_{10}$(P value). The top SNPs are shown as purple squares within the window of 100 kb. Color legend represents the linkage disequilibrium between the top SNPs and SNPs appeared in the 1 Mb window size and was calculated based on the 221 reference panel. If the peak SNP was not in the reference panel, the genotype from panel T was used for the LD calculation. The blue line represents the recombination rate from the inferred recombination maps. **a-f** stands for different LocusZoom plot for each significant association.

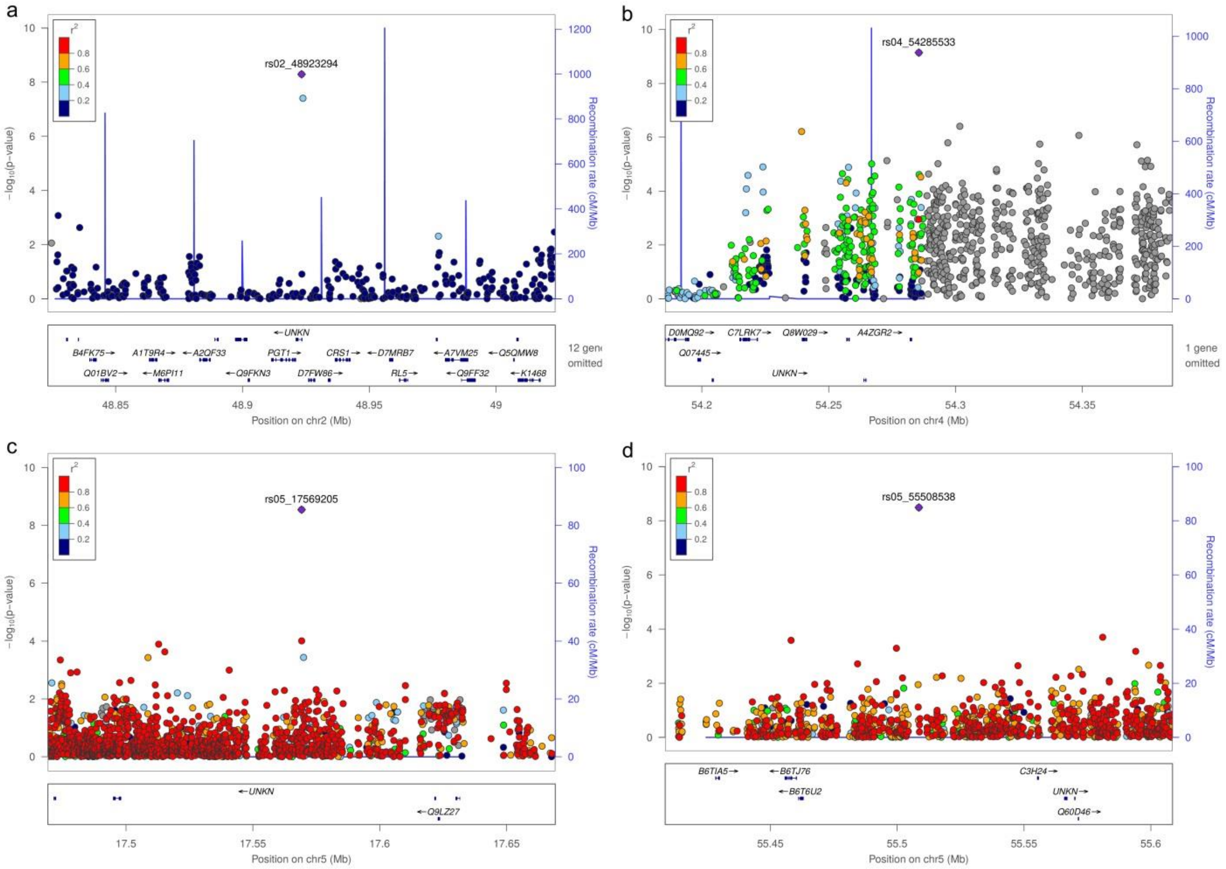

Supplementary Figure 117. LocusZoom plots of the previously identified significant associated loci in the meta-analysis of genome-wide associations of 3-methyl-1-butanol. Associations are expressed as $-\log_{10}(P$ value). The top SNPs are shown as purple squares within the window of 100 kb. Color legend represents the linkage disequilibrium between the top SNPs and SNPs appeared in the 1 Mb window size and was calculated based on the 221 reference panel. If the peak SNP was not in the reference panel, the genotype from panel T was used for the LD calculation. The blue line represents the recombination rate from the inferred recombination maps. **a-d** stands for different LocusZoom plot for each significant association.

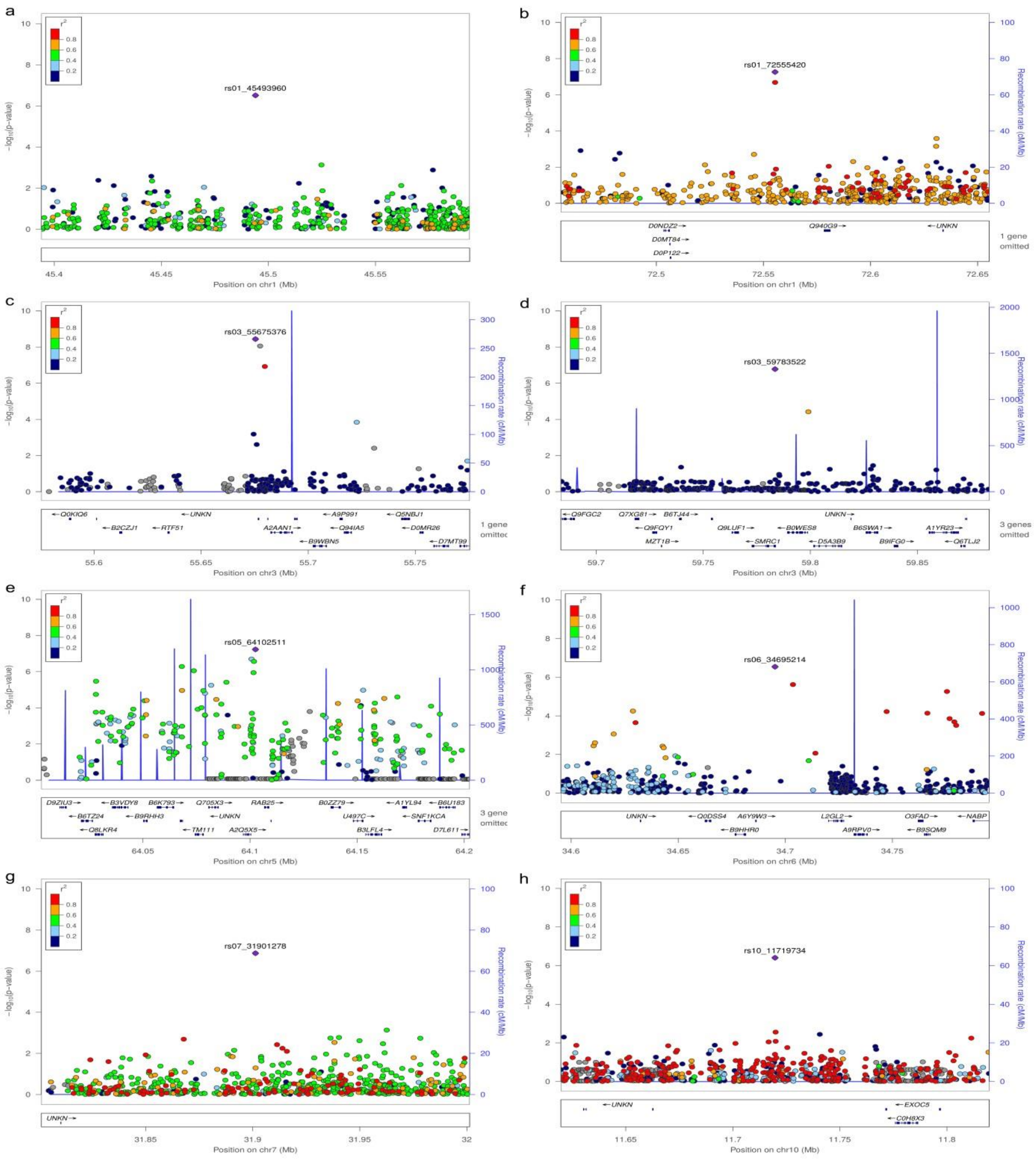

Supplementary Figure 118. LocusZoom plots of the newly identified significant associated loci in the meta-analysis of genome-wide associations of 3-methyl-1-butanol. Associations are expressed as –$\log_{10}$(*P* value). The top SNPs are shown as purple squares within the window of 100 kb. Color legend represents the linkage disequilibrium between the top SNPs and SNPs appeared in the 1 Mb window size and was calculated based on the 221 reference panel. If the peak SNP was not in the reference panel, the genotype from panel T was used for the LD calculation. The blue line represents the recombination rate from the inferred recombination maps. **a-h** stands for different LocusZoom plot for each significant association.

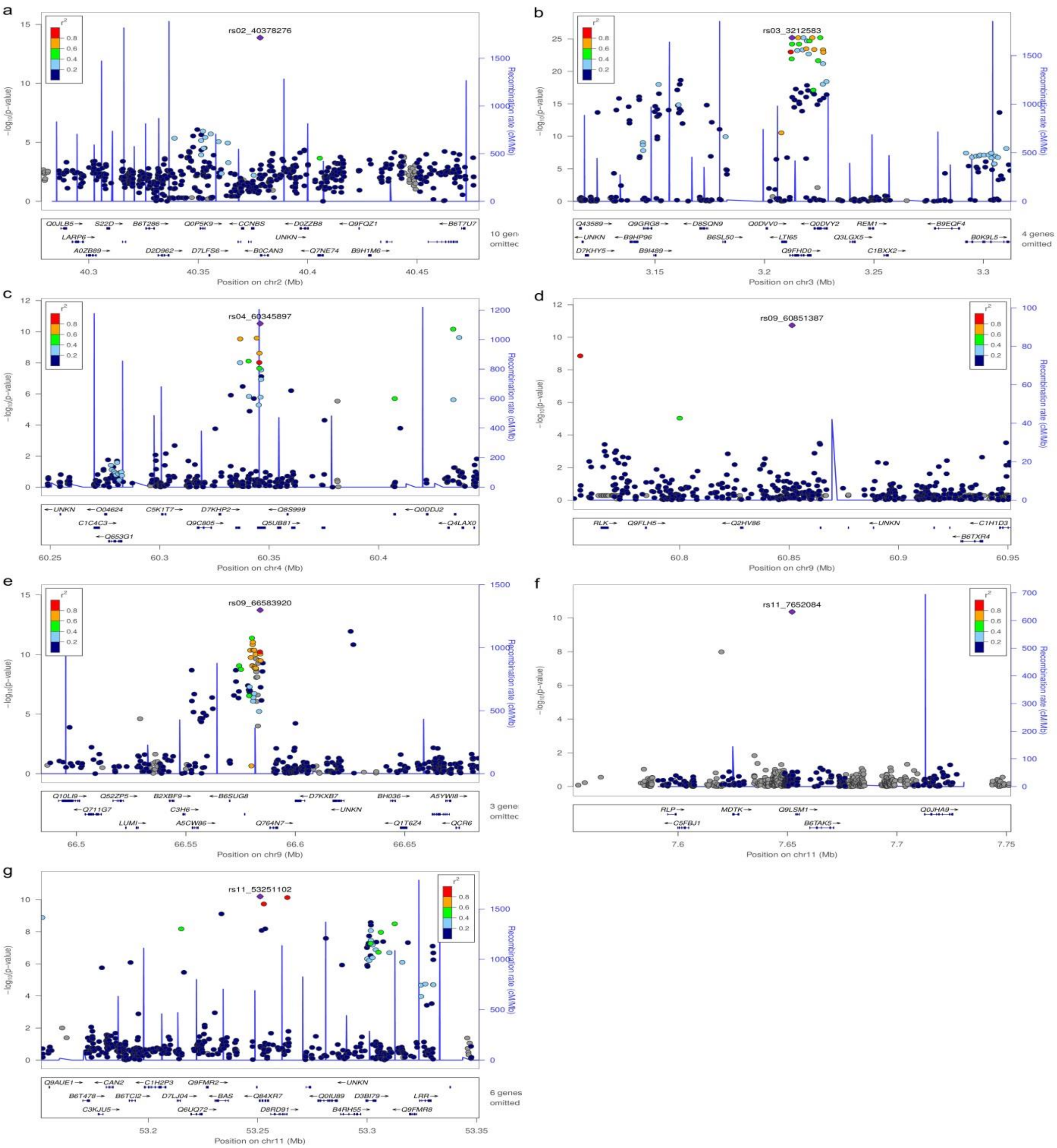

Supplementary Figure 119. LocusZoom plots of the previously identified significant associated loci in the meta-analysis of genome-wide associations of 6-methyl-5-hepten-2-one. Associations are expressed as $–\log_{10}(P$ value). The top SNPs are shown as purple squares within the window of 100 kb. Color legend represents the linkage disequilibrium between the top SNPs and SNPs appeared in the 1 Mb window size and was calculated based on the 221 reference panel. If the peak SNP was not in the reference panel, the genotype from panel T was used for the LD calculation. The blue line represents the recombination rate from the inferred recombination maps. **a-g** stands for different LocusZoom plot for each significant association.

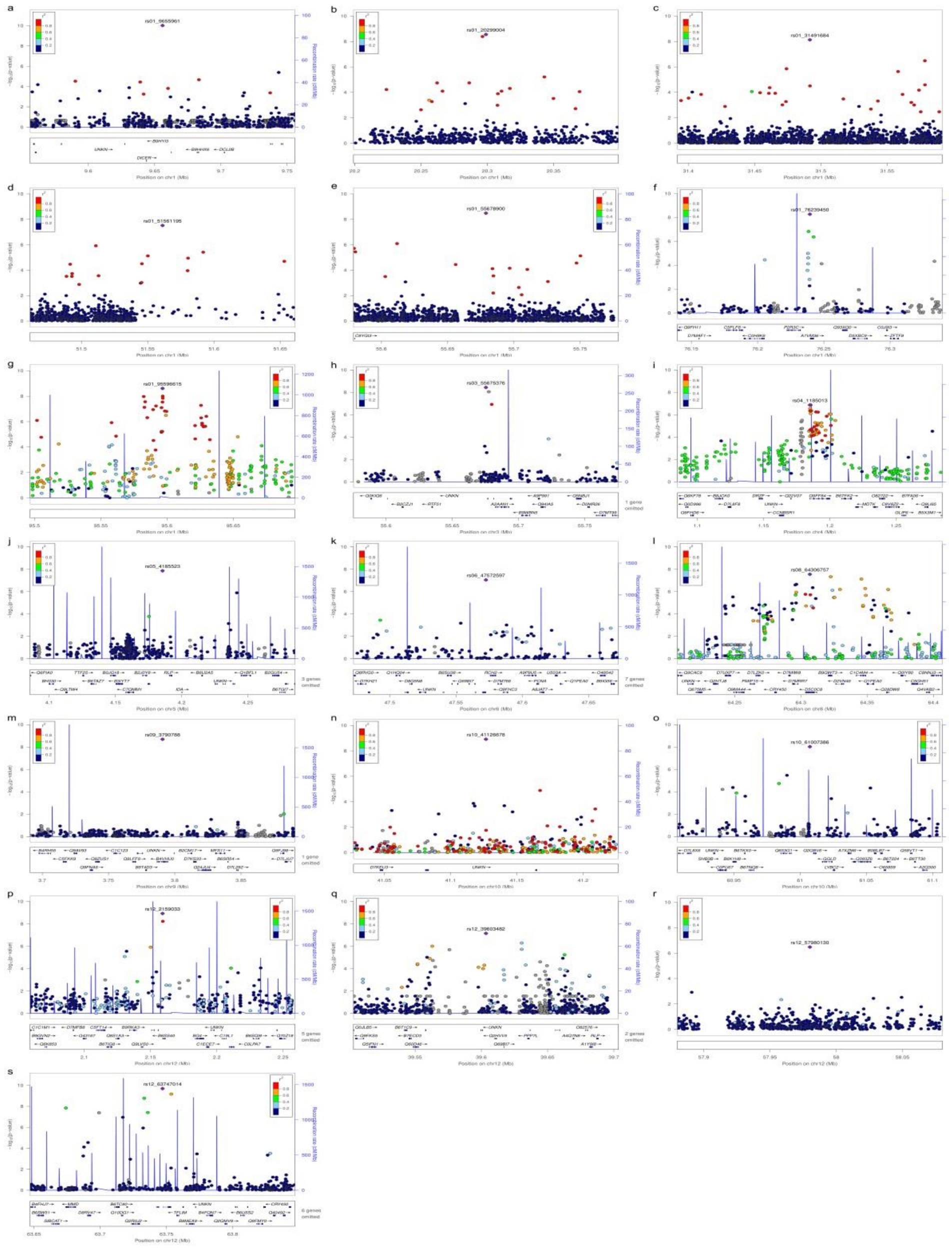

Supplementary Figure 120. LocusZoom plots of the newly identified significant associated loci in the meta-analysis of genome-wide associations of 6-methyl-5-hepten-2-one. Associations are expressed as $-\log_{10}$(P value). The top SNPs are shown as purple squares within the window of 100 kb. Color legend represents the linkage disequilibrium between the top SNPs and SNPs appeared in the 1 Mb window size and was calculated based on the 221 reference panel. If the peak SNP was not in the reference panel, the genotype from panel T was used for the LD calculation. The blue line represents the recombination rate from the inferred recombination maps. **a-s** stands for different LocusZoom plot for each significant association.

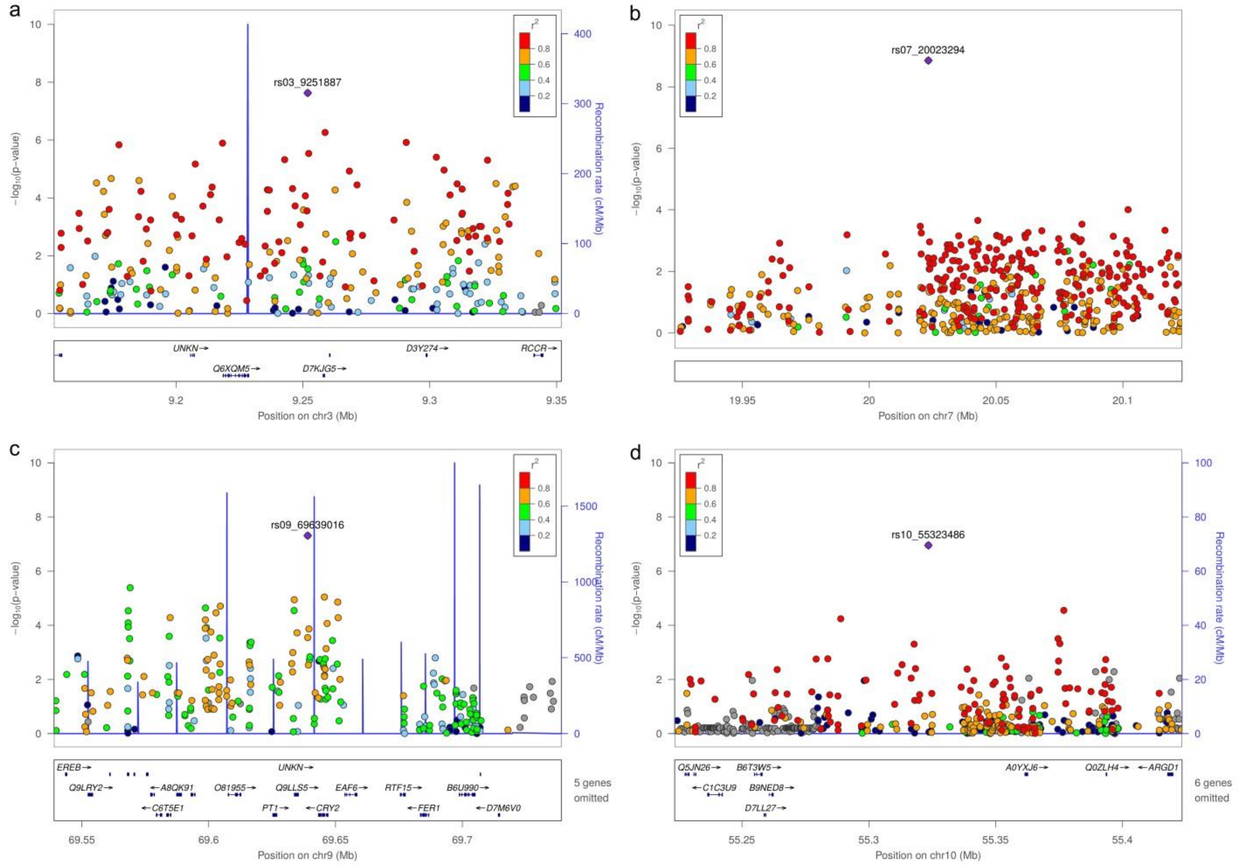

Supplementary Figure 121. LocusZoom plots of the previously identified significant associated loci in the meta-analysis of genome-wide associations of (Z)-3-hexen-1-ol. Associations are expressed as $-\log_{10}(P$ value). The top SNPs are shown as purple squares within the window of 100 kb. Color legend represents the linkage disequilibrium between the top SNPs and SNPs appeared in the 1 Mb window size and was calculated based on the 221 reference panel. If the peak SNP was not in the reference panel, the genotype from panel T was used for the LD calculation. The blue line represents the recombination rate from the inferred recombination maps. **a-d** stands for different LocusZoom plot for each significant association.

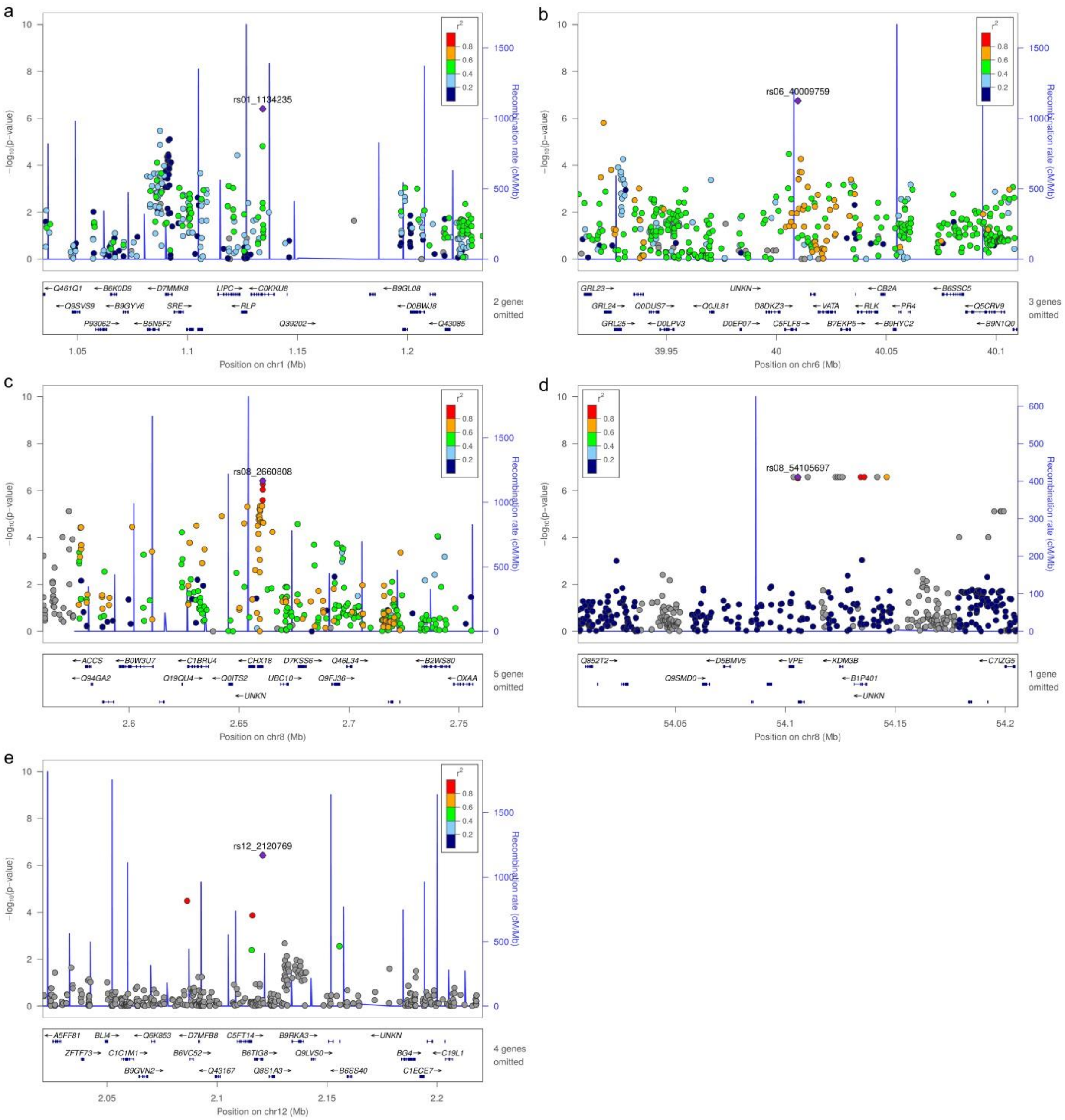

Supplementary Figure 122. LocusZoom plots of the newly identified significant associated loci in the meta-analysis of genome-wide associations of (Z)-3-hexen-1-ol. Associations are expressed as –$\log_{10}$(*P* value). The top SNPs are shown as purple squares within the window of 100 kb. Color legend represents the linkage disequilibrium between the top SNPs and SNPs appeared in the 1 Mb window size and was calculated based on the 221 reference panel. If the peak SNP was not in the reference panel, the genotype from panel T was used for the LD calculation. The blue line represents the recombination rate from the inferred recombination maps. **a-e** stands for different LocusZoom plot for each significant association.

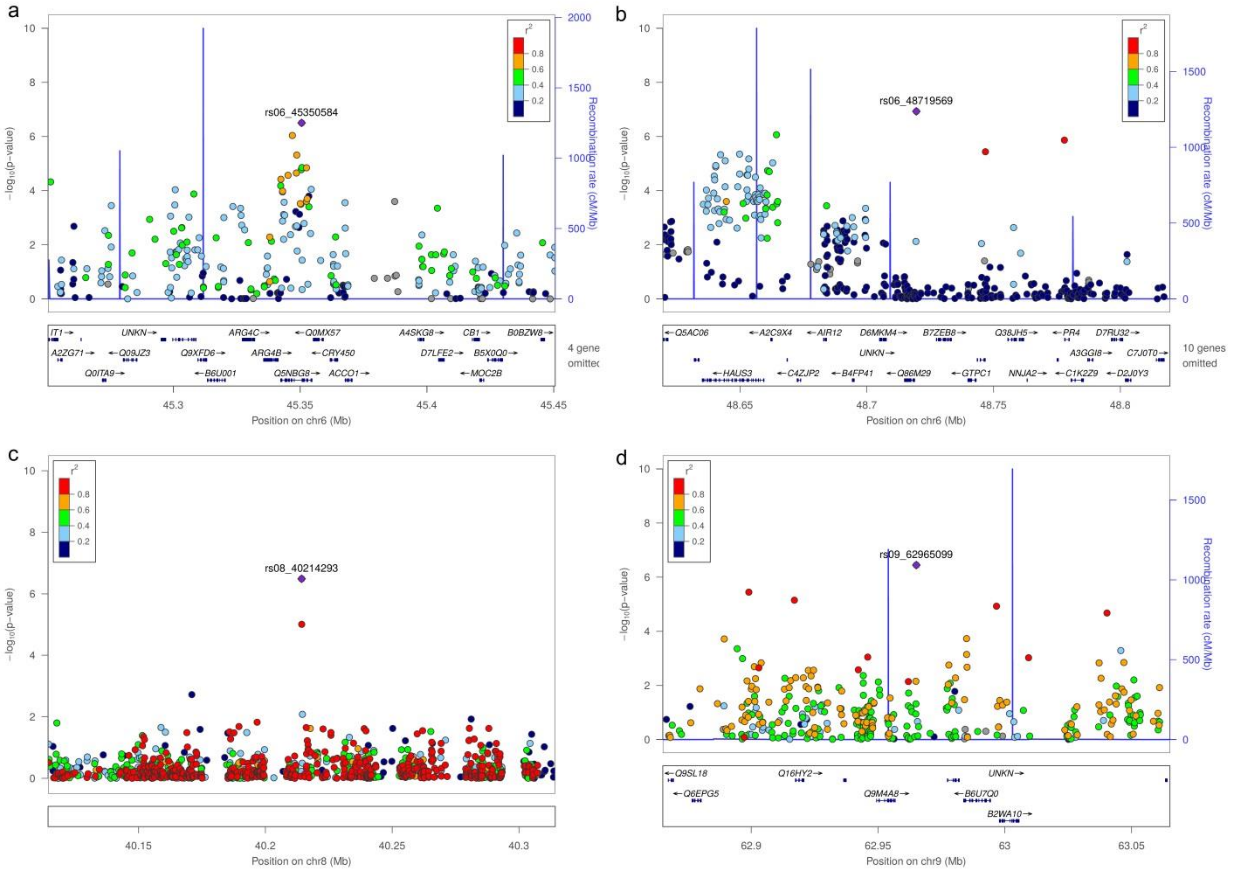

Supplementary Figure 123. LocusZoom plots of the newly identified significant associated loci in the meta-analysis of genome-wide associations of (Z)-3-hexenal. Associations are expressed as –log$_{10}$(P value). The top SNPs are shown as purple squares within the window of 100 kb. Color legend represents the linkage disequilibrium between the top SNPs and SNPs appeared in the 1 Mb window size and was calculated based on the 221 reference panel. If the peak SNP was not in the reference panel, the genotype from panel T was used for the LD calculation. The blue line represents the recombination rate from the inferred recombination maps. **a-d** stands for different LocusZoom plot for each significant association.

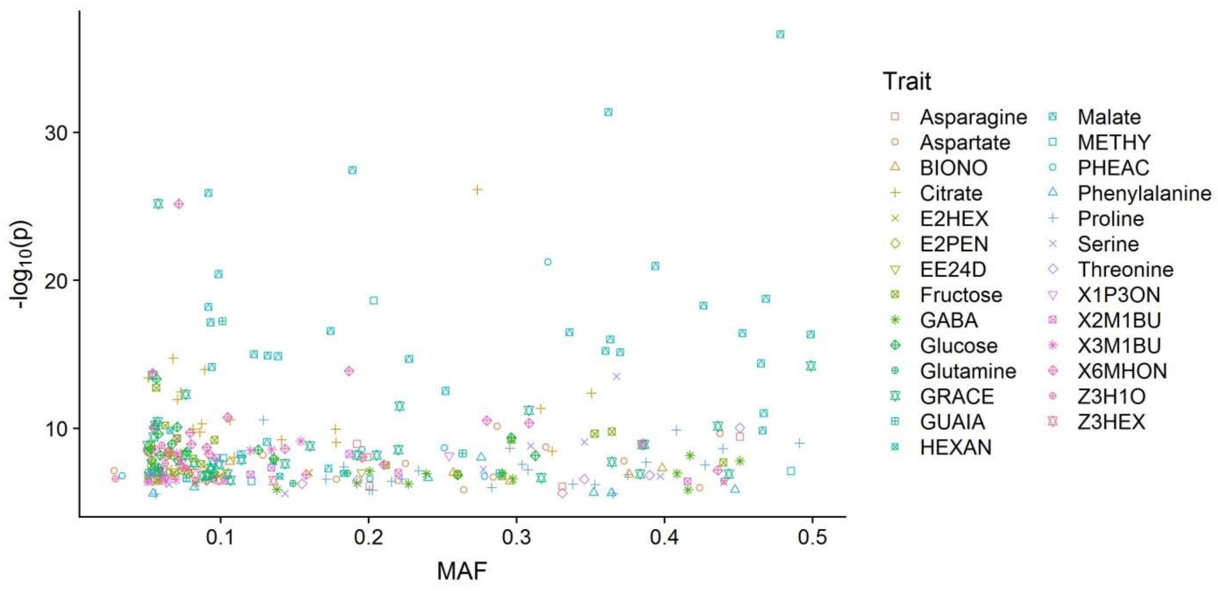

Supplementary Figure 124. Relationship between minor allele frequency and all significant association *P*-values for different metabolites. Source data are provided as a Source Data file.

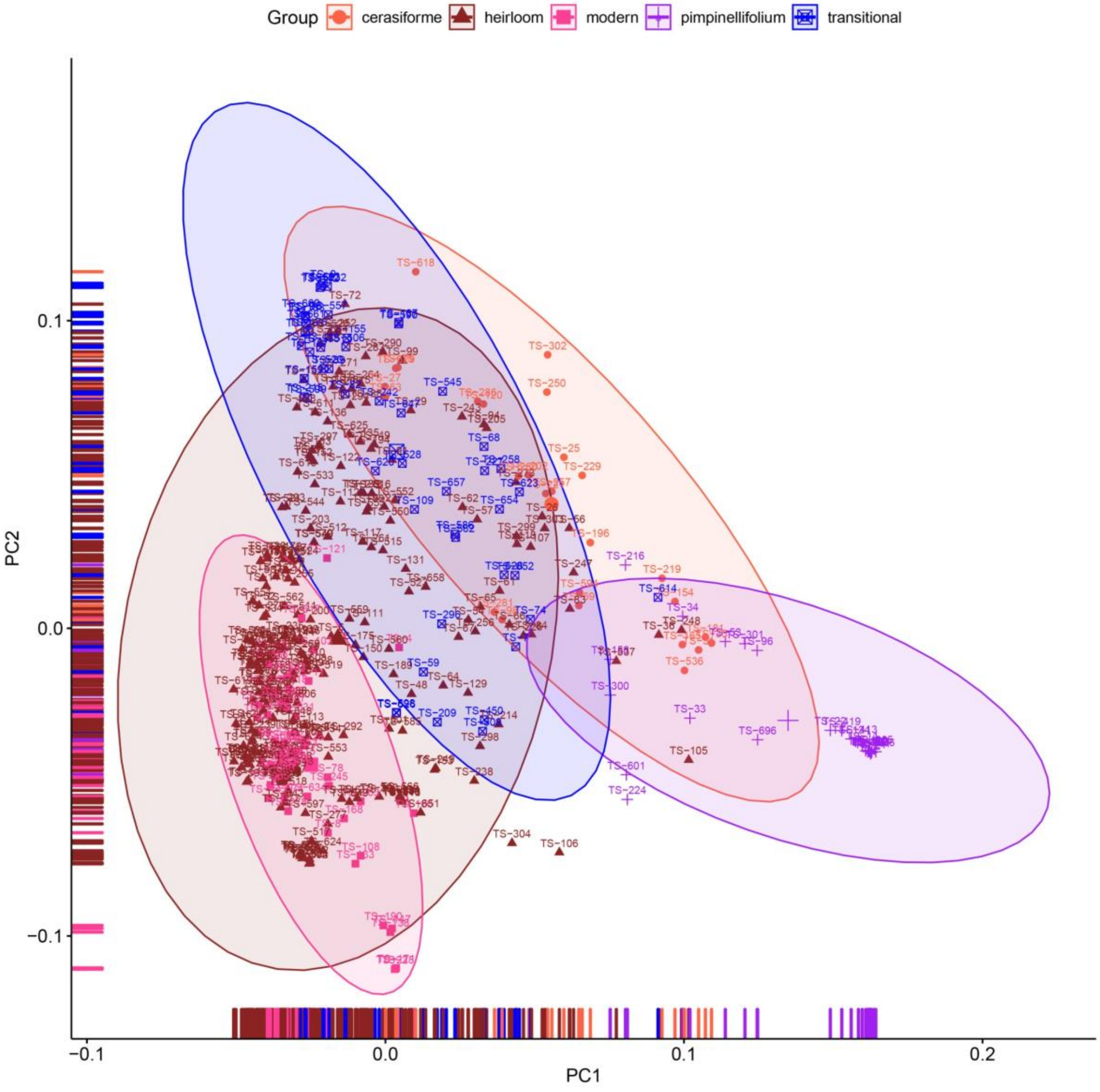

Supplementary Figure 125. Principal component analysis of panel T based on independent SNPs. Accessions were previously defined as five clusters based on DAPC analysis. Source data are provided as a Source Data file.

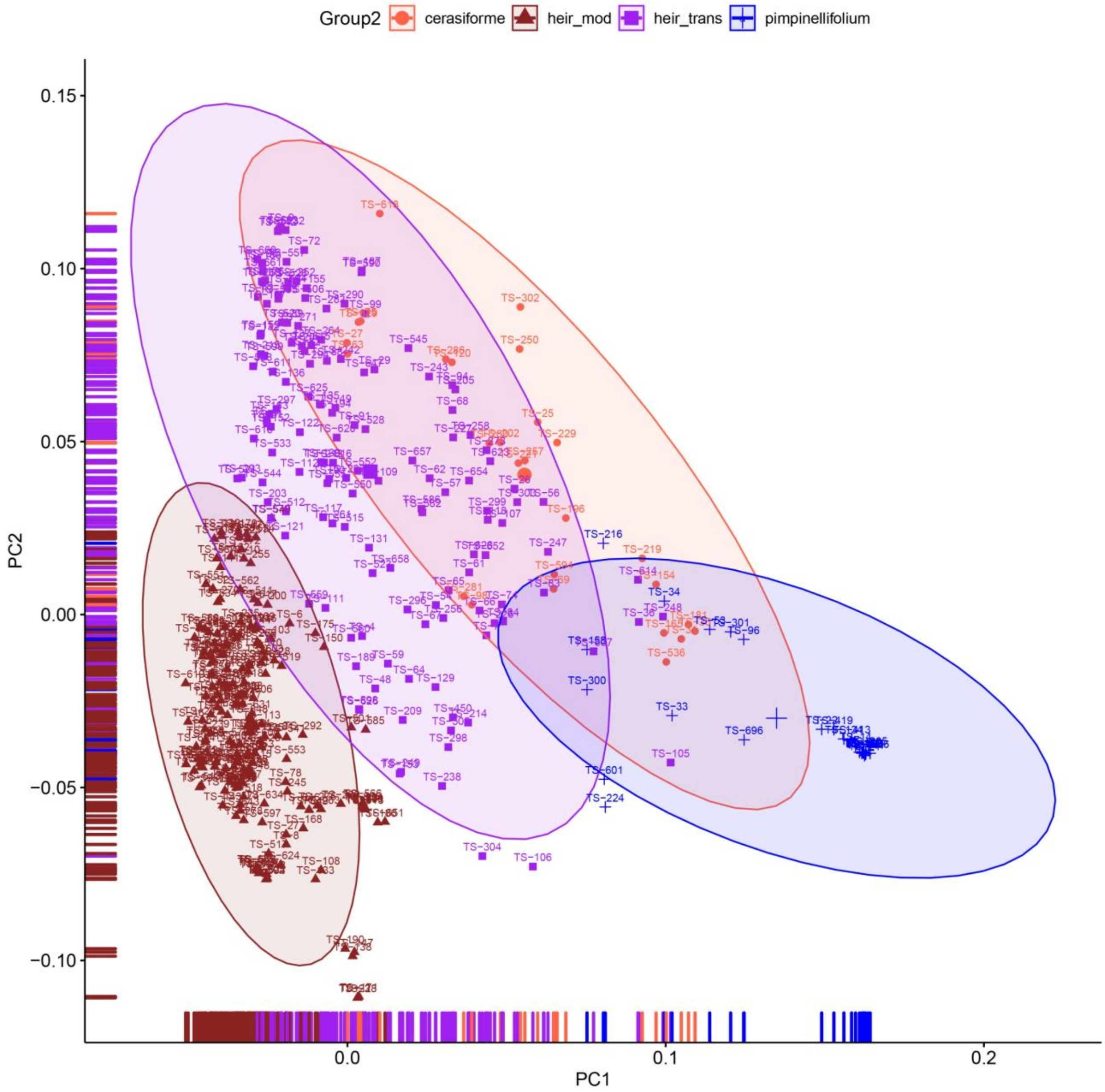

Supplementary Figure 126. Principal component analysis of panel T based on independent SNPs. Accessions were redefined as four main groups as indicated in different colored shapes. Source data are provided as a Source Data file.
