# Supplementary material for: Meta-analysis of genome-wide association studies provides insights into genetic control of tomato flavor
Source: Nat Commun. 2019 Apr 4;10:1534. doi: 10.1038/s41467-019-09462-w (PMC6449550; doi:10.1038/s41467-019-09462-w)
Supplement: Supplementary file 4 — Description of Additional Supplementary Files [file 41467_2019_9462_MOESM4_ESM.docx]

**Description of Additional Supplementary Files**

File Name: Supplementary Data 1

Description: Detailed information of the 221 reference accessions. Only accessions with genome coverage ≥ 90% and mean depth ≥ 4.0 were kept. Wild species (namely crop wild relatives) were also removed. The 9 accessions that have been both sequened and genotyped in panel S were indicated in bold red.

File Name: Supplementary Data 2

Description: Comparison of the number of correctly imputed SNPs at different imputation quality info. This comparison was done on the nine accessions that were both in the reference and panel S. Imputed SNPs were first filtered with MAF ≥ 0.037, HWE ≥ 0.000001, missing ≤ 0.1 and missing_call ≤ 0.1.

File Name: Supplementary Data 3

Description: Comparison of the number of correctly imputed SNPs at different MAF. This comparison was based on the nine accessions that were both in the reference and panel S. Imputed SNPs were first filtered with MAF ≥ 0.037, HWE ≥ 0.000001, missing ≤ 0.1 and missing_call ≤ 0.1 before counting the number.

File Name: Supplementary Data 4

Description: Detailed information of membership of each cluster revealed by all independent SNPs in DAPC for panel S.

File Name: Supplementary Data 5

Description: Detailed information of membership of each cluster revealed by all independent SNPs in DAPC for panel B.

File Name: Supplementary Data 6

Description: Genomic inflation factors for all traits from three GWAS panels before and after meta-analysis.

File Name: Supplementary Data 7

Description: Summary of all identified significant associations via meta-analysis for main flavor-related traits in tomato fruit. For each association, associated traits, SNP, chromosome (CHR), position (bp), reference allele (Ref), alternative allele (Alt), minor allele frequency (MAF), beta value of meta,analysis (Beta), standard error of meta analysis (SE), oddor ratio and 95% confidence interval of meta-analysis (OR (95% CI)), meta-analysis P value (Meta_P_value), cis-eQTL P value of the annoated candidate gene (cis-eQTL P; ns, not significant), Study direction (Direction), heterogeneity (I2), heterogeneity P value (HetPVal), start position and end position between the peak SNP and farest SNPs with linkage disequilibrium higher than 0.5 and the numer of candidate genes with this region (No. of genes), locus name of the annotated gene (Soly name) and annotated gene (Candidate gene) were provided.

File Name: Supplementary Data 8

Description: Detail information for singular enrichment analysis. Significant enriched processes and groups were indicated in bold.

File Name: Supplementary Data 9

Description: Significant tests of the Mean (± SE) malate content in the indicated allele combinations as determined by pairwise Student's t test.

File Name: Supplementary Data 10

Description: Cross-checked individual IDs of panel T. Duplicated lines are indicated in red and were removed.

File Name: Supplementary Data 11

Description: Redefined clusters of the accessions in panel T based on principal component analysis.
