# Supplementary material for: Meta-analysis of genome-wide association studies provides insights into genetic control of tomato flavor
Source: Nat Commun. 2019 Apr 4;10:1534. doi: 10.1038/s41467-019-09462-w (PMC6449550; doi:10.1038/s41467-019-09462-w)
Supplement: Supplementary file 2 — Peer Review [file 41467_2019_9462_MOESM2_ESM.pdf]

Reviewers' comments:

Reviewer #1 (Remarks to the Author):

The authors present an interesting story on meta-genome analysis of tomato fruit quality loci. Data from at least three different published experiments were combined and several hundred new loci identified and mapped as a result. They further defined putative candidate genes under a number of those loci. The story adds to our understanding of tomato fruit quality and provides an example of a meta analysis to identify QTLs. Candidate genes are presented though none are confirmed. The authors use standard analytical pipelines so I suspect they are valid but note that I am not expertise in this regard. The manuscript provide new data of value to tomato breeders and others interested in tomato fruit and flavour quality and a long list of genes to be validated by those interested in the genes and biochemistry of flavor chemistry.

The manuscript could be improved for a broader audience I believe without too much additional effort from the authors. In present form, the manuscript is mainly of value to a narrow audience interested in tomato and flavour. They suggest the value of meta analysis in identifying additional QTLs but I would argue this is obvious in cases where sufficient additional variation and/or recombination is present and monitored in the additional genotypes as compared to each prior analysis. While a number of additional QTLs are identified, the reader learns little about why or how the combined data aided QTL identification. How many of the new loci are strong versus weak QTLs? Could they have been identified in individual populations by lowering thresholds? Can the authors make inferences about LD, numbers of accessions, diversity or other general metrics that could be used to guide meta analysis or primary GWAS assessment in other systems? A brief review of prior publications by these authors indicates strong genetic and genomics backgrounds and I believe they could present their data in a way where it had more general relevance to the broader genetics and breeding communities.

Line 47 – RF: Rothan in press……this needs to be updated. There is nothing in the reference list.

Lines 182 – 184: I am confused by the use of the term "suffered" as this implies a negative connotation. I believe the authors are simply referring to possible causes of genetic diversity for citrate and their possibly underlying genes. I do not believe they intend to associate this diversity with content "suffering" in terms of low or high levels. Perhaps "was influenced" is better terminology?

Lines 258-260. This sentence is confusing. Are the authors saying cherry (small), large fruited and wild species are the "most promising candidates for high-flavor breeding purposes"? They seem to be suggesting as much, though what is left? I suspect the intended meaning here is different than conveyed. Please rewrite.

Overall the manuscript could benefit from editing for grammar, word usage, clarity and assurance of intended meaning.

Reviewer #2 wrote in the Remarks to Editor section.

(S)he thinks the using IMPUTE and filter after imputation by MAF, HWE, and INFO are appropriate. Meta-analysis strategy, i.e., using METAL as default and METASOFT under the presence of at least modearate heterogeneity, is also appropriate.

Reviewer #3 (Remarks to the Author):

The present manuscript "Meta-analysis of genome-wide association studies provide new insights into

genetic control of tomato flavor” definitely presents a new chapter in the quest to understand and apply genetic control of complex crop traits such as flavour. It combines different association studies in order to produce more information about genetic loci that possibly control tomato fruits flavour and will serve as a useful resource for researchers and breeders working in this area.

I believe it would help understanding the results of this study if a few issues listed below are addressed.

Meta-analyses in human studies commonly detect genetic variation that may explain very little proportion of phenotypic variation. In this study about 2/3 of the associations detected were new compared to those had been found previously. This is a considerable amount of new information, but what quantitative value these new loci would have in addition to already known loci? Are we not looking here at e.g. environmentally vulnerable small effects which now popped up just because the sample size and therefore the statistical power increased? Can this be estimated and discussed?
The genetic structure of the combined data was described well and was dealt appropriately in the GWAS analysis. However, very little was said about phenotypic structure – the data came from different studies with different plant growth conditions, different harvesting and sampling procedures, different metabolic analysis protocols etc., which are all non-genetic sources of variation which could lead to significant phenotypic batch effects. Could the authors show the phenotypic variation just as they did for the genetic variation, e.g. using PCA, and to explain how they dealt with the batch effects?

It has been found by the authors that the flavour related chemical composition tends to improve with an increase of alternative alleles at the associated genetic loci, e.g. sugar content positively correlates with the number of alternative alleles of the associated genes. However, most of the alternative alleles in this case come from the berry-sized wild accessions, whose overall genetic background and therefore physiological background is quite different. It is, therefore, quite possible that all these alternative alleles and their combinations may lead to the chemical phenotype only when the overall background is “suitable”. As it looks in Fig.2d, the sugar content mainly depends on fruits size. And indeed most of the loci mentioned are in close proximity to known loci for fruit size. Thus, is this then possible that we are simply looking at the fruit size effect?

Do Beta values in Supplemental Table S9 indicate size and directionality of the effects of alternative alleles? If so then counting the effects up of the alleles of the selected sugar genes for the two most extreme allele combinations in Fig.2e RAARA allele combination should give a higher sugar content than ARAAA combination, but in Fig. 2E they are the other way around. Also in the Discussion (p.12,l.247) it is stated that that the sugar content can be significantly improved almost all alternative alleles are selected, also combining the effects of these alleles does not really support this. How can these contradiction be explained? Or the data should be interpreted in a different way? Can the authors then provide more extensive guidance how to interpret different parameters in terms of their biological meaning?

P9L174 The authors refer to cherry tomato on Fig 3c,d and also use this later in the text (Fig.4h) in comparison with “modern” tomato. Cherry tomato varieties has been introduced to the market since 1990th and therefore also should be considered as modern tomato. Since many wild cerasiforme accessions were used in this study, it would be better if the authors come up with an alternative term for what they call cherry tomato in order to avoid any confusion.

Reviewers' comments:

Reviewer #1 (Remarks to the Author):

The manuscript could be improved for a broader audience I believe without too much additional effort from the authors. In present form, the manuscript is mainly of value to a narrow audience interested in tomato and flavour. They suggest the value of meta analysis in identifying additional QTLs but I would argue this is obvious in cases where sufficient additional variation and/or recombination is present and monitored in the additional genotypes as compared to each prior analysis. While a number of additional QTLs are identified, the reader learns little about why or how the combined data aided QTL identification. How many of the new loci are strong versus weak QTLs? Could they have been identified in individual populations by lowering thresholds? Can the authors make inferences about LD, numbers of accessions, diversity or other general metrics that could be used to guide meta analysis or primary GWAS assessment in other systems? A brief review of prior publications by these authors indicates strong genetic and genomics backgrounds and I believe they could present their data in a way where it had more general relevance to the broader genetics and breeding communities.

Reply: Many thanks for this comment. We would like to provide a point-to-point reply.

Q1: While a number of additional QTLs are identified, the reader learns little about why or how the combined data aided QTL identification. How many of the new loci are strong versus weak QTLs? Could they have been identified in individual populations by lowering thresholds?

Reply 1: We added a new paragraph in the discussion section to show readers the main advantages of meta-analysis of GWASs (**Line 229-245**). Briefly, we demonstrated the great potentials of meta-analysis of GWASs in other major crops. We discussed the main differences between mega-analysis (re-analysis of pooled data) and meta-analysis of GWASs. We then discussed the main benefits of meta-analysis. As for strong versus weak QTLs, some parameters derived from the meta-analysis should be detailed. For example, if we use $P = 1.00\times10^{-09}$ as the genome-wide threshold of strong QTLs, then a total of 87 strong meta-QTLs were identified (**Supplementary Table 9; Line 90-91**). Among all the newly identified loci, most of them passed the suggestive thresholds ($P = 10^{-04}$) in at least one panel (**Figure S13-75; Line 91-92**).

Q2: Can the authors make inferences about LD, numbers of accessions, diversity or other general metrics that could be used to guide meta analysis or primary GWAS assessment in other systems?

Reply 2: We cited two publications which detailed protocols to perform meta-analysis of GWASs (**Line 230-232**). Most of the steps can be directly applied for other systems. METAL[1] (fixed-effect model) and METASOFT[2] (random-effect model) are the two most commonly used meta-analysis software[3] (**Line 570-571**). We explained in details about these two methods in the online methods (**Line 571-577**). We also provided a detailed script to perform meta-analysis along with all the raw meta-analysis data (See data availability **Line 623-625**; also see link at: https://data.inra.fr/privateurl.xhtml?token=a1b23652-4d2f-4a49-9b83-88a6236f9151).

Q3: Line 47 – RF: Rothan in press……this needs to be updated. There is nothing in the reference list.

Reply 3: The paper of Rothan was recently online and we have updated this (**Line 48**).

Q4: Lines 182 – 184: I am confused by the use of the term "suffered" as this implies a negative connotation. I believe the authors are simply referring to possible causes of genetic diversity for citrate and their possibly underlying genes. I do not believe they intend to associate this diversity with content "suffering" in terms of low or high levels. Perhaps "was influenced" is better terminology?

Reply 4: Thanks for this concern. Following your suggestions, we replaced the term "suffered" to "was influenced" (**See Line 189-190; 270; 281-282**).

Q5: Lines 258-260. This sentence is confusing. Are the authors saying cherry (small), large fruited and wild species are the "most promising candidates for high-flavor breeding purposes"? They seem to be suggesting as much, though what is left? I suspect the intended meaning here is different than conveyed. Please rewrite.

Reply 5: Sorry for this misleading. In fact, the cherry tomato genomes are mosaic of big-fruited tomatoes and wild species genomes. We have modified this sentence in the revised manuscript (**See Line 287-291**).

Q6: Overall the manuscript could benefit from editing for grammar, word usage, clarity and assurance of intended meaning.

Reply 6: Many thanks. The two co-authors Harry Klee and Denise Tieman, from University of Florida, USA (both of them are native English speakers), have checked for these minor issues and a new careful reading was performed.

Reviewer #2 wrote in the Remarks to Editor section.

(S)he thinks the using IMPUTE and filter after imputation by MAF, HWE, and INFO are appropriate. Meta-analysis strategy, i.e., using METAL as default and METASOFT under the presence of at least modearate heterogeneity, is also appropriate.

Reply: Many thanks for your positive feedbacks.

Reviewer #3 (Remarks to the Author):

The present manuscript "Meta-analysis of genome-wide association studies provide new insights into genetic control of tomato flavor" definitely presents a new chapter in the quest to understand and apply genetic control of complex crop traits such as flavour. It combines different association studies in order to produce more information about genetic loci that possibly control tomato fruits flavour and will serve as a useful resource for researchers and breeders working in this area.

I believe it would help understanding the results of this study if a few issues listed below are addressed.

Q1: Meta-analyses in human studies commonly detect genetic variation that may explain very little proportion of phenotypic variation. In this study about 2/3 of the associations detected were new compared to those had been found previously. This is a considerable amount of new information, but what quantitative value these new loci would have in addition to already known loci? Are we not looking here at e.g. environmentally vulnerable small effects which now popped up just because the sample size and therefore the statistical power increased? Can this be estimated and discussed?

Reply 1: Many thanks for these comments. In fact, we have tried to analyze how much new heritability or percentage of phenotypic variation can be explained based on summary statistics of these new identified loci. We have tried both the LD score regression approach (LDSC)[4] and Heritability Estimation from Summary Statistics (HESS)[5]. However, there are at least two crucial

problems: substantially large population size (in humans, around or above 50,000 samples) and large high sequencing-depth reference panels. Based on the available data we have, the heritability estimated was not that trust-worthy. We agree that a large part of the new associations may result from the power of increased sample size and SNP imputation as stated earlier that among all the newly identified loci, most of them passed the suggestive thresholds ($P = 10^{-04}$) in at least one panel (**Figure S13-75; Line 91-92**).

For your concern about environmentally vulnerable small effects, in meta-analysis of GWASs, this has already been handled properly. In fact, environmental effect is just one cause of cross-study heterogeneity. Together with other causes, such as population structure, LD, phenotyping platforms, G×E interactions, were managed by using the heterogeneity I^2. If I^2 is lower than 25%, it means the heterogeneity is quite small. If I^2 is higher than 25%, it means that is strong heterogeneity, which can be caused by many unknown factors, including environmentally vulnerable small effects. We properly handled this using the random-effect model as stated at **Line 574-577.**

Q2: The genetic structure of the combined data was described well and was dealt appropriately in the GWAS analysis. However, very little was said about phenotypic structure – the data came from different studies with different plant growth conditions, different harvesting and sampling procedures, different metabolic analysis protocols etc., which are all non-genetic sources of variation which could lead to significant phenotypic batch effects. Could the authors show the phenotypic variation just as they did for the genetic variation, e.g. using PCA, and to explain how they dealt with the batch effects?

Reply 2: Thanks for this comment. For the phenotype data of each individual panel, they have been properly managed and normalized before GWASs[6–8] (**see Materials and Methods of the 3 GWAS articles**). All your concerns are crucial when you want to combine the phenotypes and genotypes of all panels and run a single GWAS using the combined data, which is technically referred as mega-analysis of GWASs. For mega-analysis, genotypes and phenotypes from all panels should be first combined and then analyzed, which requires proper management of the phenotypic structure. In contrast, meta-analysis has great benefits when pooling raw data of individual panels (mega-analysis) are impossible. In this study, we only had the summary data from each GWAS. This is discussed at **Lines 229-245** in the discussion.

Q3: It has been found by the authors that the flavour related chemical composition tends to improve with an increase of alternative alleles at the associated genetic loci, e.g. sugar content positively correlates with the number of alternative alleles of the associated genes. However, most of the alternative alleles in this case come from the berry-sized wild accessions, whose overall genetic background and therefore physiological background is quite different. It is, therefore, quite possible that all these alternative alleles and their combinations may lead to the chemical phenotype only when the overall background is “suitable”. As it looks in Fig.2d, the

sugar content mainly depends on fruits size. And indeed most of the loci mentioned are in close proximity to known loci for fruit size. Thus, is this then possible that we are simply looking at the fruit size effect?

Reply 3: Fruit weight was influenced by strong domestication and improvement sweeps, which have been detailed in Lin et al. (2014)[9]. In their study, they provided a summary table of fruit weight loci selected during domestication and improvement (**Supplementary Table 11 of Lin's paper**; can also be downloaded at: **https://www.nature.com/articles/ng.3117#supplementary-information**). However, for all the 6 loci we analyzed in Figure 2, only the locus on chromosome 11 was influenced by a domestication sweep (DW149). In Lin's study, all the three main QTLs (*fw11.1*, *fw11.2*, *fw11.3*) were influenced by improvement sweeps, instead of domestication sweeps. For the remaining 5 loci, they were not influenced by any domestication and improvement sweeps. So, we conclude that though sugar content has a strong negative correlation with fruit weight, it is more likely for the 6 loci associated with both fructose and glucose, were not strongly selected or were loosely linked to fruit weight QTLs (**Line 269-272**). By the way, we identified more loci for these two sugars, some of which being close to fruit weight QTLs but we did not analyze them.

Q4: Do Beta values in Supplemental Table S9 indicate size and directionality of the effects of alternative alleles? If so then counting the effects up of the alleles of the selected sugar genes for the two most extreme allele combinations in Fig.2e RAARA allele combination should give a higher sugar content than ARAAA combination, but in Fig. 2E they are the other way around. Also in the Discussion (p.12,l.247) it is stated that the sugar content can be significantly improved almost all alternative alleles are selected, also combining the effects of these alleles does not really support this. How can these contradiction be explained? Or the data should be interpreted in a different way? Can the authors then provide more extensive guidance how to interpret different parameters in terms of their biological meaning?

Reply 4: If the genotypes of each individual panel were aligned with the reference genome and the first allele in the genotype files were always the alternative allele, then the beta values indeed would indicate the size and directionality of the effects of alternative alleles. However, we did not do this and the directions in Supplementary Table S9 also indicated that the first allele in the genotype files of different panels was not always the reference genome. As for your main concerns about the allele combinations, we would like to provide more detailed explanations. 1) The allele combination analysis was only based on panel T as explained in Online Materials **Lines 604-621**). We did not combine three panels because the sugar content had strong cross-study heterogeneity and we could not simply combine the panels. Panel T had the largest population size among the three panels and we therefore only focused on panel T. 2) As panel T was genotyped by resequencing, we did not perform genotype imputation for this panel. Before we analyze the relationship between sugar contents and allele combinations, we had to remove accessions with too much missing genotype calls. Thus for some allele combinations, the number of individuals was quite small, 4) For some allele combinations, including RAARA, RRRAR,

ARRRR and AARRR, for which there was only one observation and we could not perform statistical tests as explained in Online Materials **Line 620-621**.

Q5: P9L174 The authors refer to cherry tomato on Fig 3c,d and also use this later in the text (Fig.4h) in comparison with "modern" tomato. Cherry tomato varieties has been introduced to the market since 1990th and therefore also should be considered as modern tomato. Since many wild cerasiforme accessions were used in this study, it would be better if the authors come up with an alternative term for what they call cherry tomato in order to avoid any confusion.

Reply 5: Though cherry tomato varieties have been introduced to the market since 1990s, they are admixture of modern big-fruited tomatoes and wild species[9,10]. So, cherry tomatoes are not really modern tomatoes. We have clarified this at **Lines 287-291.**

Related References

1. Willer, C. J., Li, Y. & Abecasis, G. R. METAL: Fast and efficient meta-analysis of genomewide association scans. *Bioinformatics* **26,** 2190–2191 (2010).

2. Han, B. & Eskin, E. Random-effects model aimed at discovering associations in meta-analysis of genome-wide association studies. *Am. J. Hum. Genet.* **88,** 586–598 (2011).

3. Evangelou, E. & Ioannidis, J. P. A. Meta-analysis methods for genome-wide association studies and beyond. *Nat. Rev. Genet.* **14,** 379–389 (2013).

4. Bulik-Sullivan, B. K. *et al.* LD Score regression distinguishes confounding from polygenicity in genome-wide association studies. *Nat. Genet.* **47,** 291–295 (2015).

5. Shi, H., Kichaev, G. & Pasaniuc, B. Contrasting the Genetic Architecture of 30 Complex Traits from Summary Association Data. *Am. J. Hum. Genet.* **99,** 139–153 (2016).

6. Tieman, D. *et al.* A chemical genetic roadmap to improved tomato flavor. *Science (80-.).* **355,** 391–394 (2017).

7. Sauvage, C. *et al.* Genome-Wide Association in Tomato Reveals 44 Candidate Loci for Fruit Metabolic Traits. *Plant Physiol.* **165,** 1120–1132 (2014).

8. Bauchet, G. *et al.* Identification of major loci and genomic regions controlling acid and volatile content in tomato fruit: implications for flavor improvement. *New Phytol.* **215,** 624–641 (2017).

9. Lin, T. *et al.* Genomic analyses provide insights into the history of tomato breeding. *Nat. Genet.* **46,** 1220–1226 (2014).

10. Blanca, J. *et al.* Genomic variation in tomato, from wild ancestors to contemporary breeding accessions. *BMC Genomics* **16,** 257 (2015).

REVIEWERS' COMMENTS:

Reviewer #1: unavailable.

Reviewer #3 (Remarks to the Author):

I think most of my comments were addressed in the revised version and in the replies sufficiently. I have no further comments.

I was also asked by the editor to comment whether authors have adequately addresses Reviewer #1's previous concerns. I think authors provided sufficient explanations or/and additional information.
